# ﻿Revision of the *Austrelatuspapuensis* group with descriptions of 42 new species from New Guinea (Coleoptera, Dytiscidae, Copelatinae)

**DOI:** 10.3897/zookeys.1201.116131

**Published:** 2024-05-10

**Authors:** Helena Shaverdo, Lars Hendrich, Suriani Surbakti, Rawati Panjaitan, Michael Balke

**Affiliations:** 1 Naturhistorisches Museum Wien, Burgring 7, 1010 Vienna, Austria Naturhistorisches Museum Wien Vienna Austria; 2 SNSB-Zoologische Staatssammlung München, Münchhausenstraße 21, D-81247, Munich, Germany SNSB-Zoologische Staatssammlung München Munich Germany; 3 GeoBioCenter, Ludwig-Maximilians-University, Munich, Germany Ludwig-Maximilians-University Munich Germany; 4 Department of Biology, Universitas Cendrawasih, Jayapura, Papua, Indonesia Universitas Cendrawasih Jayapura Indonesia; 5 Department of Biology, Faculty of Sciences and Mathematics, State University of Papua (UNIPA), Jalan Gunung Salju Amban, Manokwari, 98314, West Papua, Indonesia State University of Papua Manokwari Indonesia

**Keywords:** Australasia, Dytiscidae, New Guinea, new species, species group, taxonomy

## Abstract

The *Austrelatuspapuensis* group is the second species group of the New Guinean representatives of the recently described genus *Austrelatus*Shaverdo et al., 2023. The group is mainly defined by distinct scale- and/or spinula-like surface structures of the dorsal sclerite of the median lobe. The species group already contains four described species and 42 new species and one subspecies treated here: *Austrelatusaiyurensis***sp. nov.**, *A.asteios***sp. nov.**, *A.bewaniensis***sp. nov.**, *A.bosaviensis***sp. nov.**, *A.bundunensis***sp. nov.**, *A.centralensis***sp. nov.**, *A.craterensis***sp. nov.**, *A.decoris***sp. nov.**, *A.dekai***sp. nov.**, *A.epicharis***sp. nov.**, *A.flavocapitatus***sp. nov.**, *A.fuscus***sp. nov.**, *A.herzogensis***sp. nov.**, *A.inconstans***sp. nov.**, *A.iriatoi***sp. nov.**, *A.kalibumi***sp. nov.**, *A.kebarensis***sp. nov.**, *A.kokodensis***sp. nov.**, *A.leptos***sp. nov.**, *A.loloki***sp. nov.**, *A.lopintolensis***sp. nov.**, *A.madangensis***sp. nov.**, *A.maindai***sp. nov.**, *A.mamberamo***sp. nov.**, *A.mianminensis***sp. nov.**, *A.miltokarenos***sp. nov.**, *A.noiadi***sp. nov.**, *A.normanbyensis***sp. nov.**, *A.ohu***sp. nov.**, *A.posmani***sp. nov.**, *A.procerus***sp. nov.**, *A.pseudogestroi***sp. nov.**, *A.pseudomianminensis***sp. nov.**, *A.robustus***sp. nov.**, *A.sararti***sp. nov.**, *A.sumokedi***sp. nov.**, *A.wanangensis***sp. nov.**, *A.wasiorensis***sp. nov.**, *A.wasurensis***sp. nov.**, *A.weigeli***sp. nov.**, *A.yamurensis***sp. nov.**, *A.yeretuar***sp. nov.**, *A.xanthocephalusnabirensis***ssp. nov.** A checklist and identification key to New Guinean species of the group are provided and important diagnostic characters are illustrated. Data on the species distributions and habiat preferences are given.

## ﻿﻿Introduction

The genus *Austrelatus*Shaverdo et al., 2023 has been recently described for 62 Australasian species, 37 of which are known from New Guinea. The New Guinean *Austrelatus* species include *A.clarki* (Sharp, 1882), *A.fumato*Shaverdo et al., 2023, and *A.setiphallus*Shaverdo et al., 2023 with rather distinct morphology of the male genitalia, as well as 32 species of the *A.neoguineensis* group (Shaverdo et al. 2023). The remained four species of New Guinean *Austrelatus* are *A.luteomaculatus* (Guignot, 1956), *A.gestroi* (Régimbart, 1892), *A.papuensis* (J. Balfour-Browne, 1939), and *A.xanthocephalus* (Régimbart, 1899) that have distinct scale- and/or spinula-like surface structures of the dorsal sclerite of the median lobe. Therefore, they were placed into the *A.papuensis* group introduced by Shaverdo et al. (2023) and defined here in detail. Based on this special character of the male genitalia, the Solomon Islands species *A.baranensis* (Hájek, Shaverdo, Hendrich & Balke, 2021), *A.bougainvillensis* (Hájek, Shaverdo, Hendrich & Balke, 2021), and *A.kietensis* (Hájek, Shaverdo, Hendrich & Balke, 2021) and the Moluccan species *A.sibelaemontis* (Hájek, Hendrich, Hawlitschek & Balke, 2010), *A.ternatensis* (Régimbart, 1899), and *A.wallacei* (J. Balfour-Browne, 1939) are also considered to belong to the *A.papuensis* group (Shaverdo et al. 2023). At present, the *A.papuensis* group is the largest species group of *Austrelatus*, because 42 new species from New Guinea described here also belong to it, which increases its species number to 52.

The present work is aimed to define in detail the *A.papuensis* group, describe its new representatives, and provide a key to their identification as well as information on their distributions and habitats.

## ﻿﻿Materials and methods

The present work is based on material from the following collections:

**BMNH** The Natural History Museum, London, UK

**CAS** Collection of Anders Skale, Gera, Germany

**CLH** Collection of Lars Hendrich, Munich, Germany (property of NHMW)

**HNHM** Hungarian Natural History Museum, Budapest, Hungary

**IRSNB** Institut Royal des Sciences Naturelles de Belgique, Brussels, Belgium

**KSP** Kelompok Serranga Papua Collection, UNCEN, Jayapura, Papua, Indonesia

**MNHN** Muséum national d‘Histoire naturelle, Paris, France

**MTD** Museum für Tierkunde, Dresden, Germany

**MZB** Museum Zoologicum Bogoriense, Cibinong, West Java, Indonesia

**NHMB** Naturhistorisches Museum, Basel, Switzerland

**NHMW** Naturhistorisches Museum Wien, Vienna, Austria

**NMPC** Národní muzeum, Prague, Czech Republic

**RMNH** Naturalis Biodiversity Center

**SMNS** Staatliches Museum für Naturkunde, Stuttgart, Germany

**ZSM** SNSB-Zoologische Staatssammlung München, Munich, Germany

All specimen data are quoted as they appear on the labels attached to the specimens. Label text is cited using quotation marks; comments in square brackets are ours, including hw (handwritten). All holotypes and paratypes are provided with corresponding red printed labels. Administrative divisions of Indonesia and Papua New Guinea follow information from Wikipedia (2021a, b, c).

The methods used to study and illustrate morphology follow those of Shaverdo et al. (2023). In female descriptions, as “matt forms” we indicate females with the pronotum and elytron very densely covered with fine longitudinal strioles. For most of the species, the median lobe should be dissected to study structure of its ventral sclerite in detail. Terminology of the median lobe sclerites follows that of Shaverdo et al. (2023) where the illustrations (fig. 4 on p. 15 and fig. 5 on p. 16) can be consulted. In the female descriptions, “as male” is with an exclusion of the male characters of the pro- and mesoleg, i.e., protibiae, pro- and mesotarsi, and proclaws are not modified in females. The species descriptions are given in the alphabetic order. Arrangement of the figures follows the species order in the key to simplify use of the key and affinity notes. The morphological illustrations are followed by distributional maps and habitats photos.

The following abbreviations were used in the descriptions: **DBE** – minimum distance between eyes; **PL** – pronotal length (along midline from anterior to posterior margins); **PW** – pronotal width at level of posterior margin; **TL** – total length, measured from front of head to apex of elytra; **TL-H** – total length minus head length, measured from anterior margin of pronotum to apex of elytra; **MW** – maximum width of body measured at right angle to TL.

## ﻿﻿Results

### ﻿﻿*Austrelatuspapuensis* group

The group is represented in New Guinea by 46 species (Table 1), 42 of which are new and described below.

The diagnostic characters of the group are mainly those of the median lobe:

- median lobe of aedeagus with dorsal and ventral sclerites not separated medially, pressed together;
- dorsal and ventral sclerites each divided into two lobes in apical half;
- dorsal sclerite more strongly sclerotised than partly membranous ventral one, ventral lobes (especially left one) each with a differently developed lateral sclerotised area;
- apexes of lobes of dorsal and ventral sclerites without strong modification, elongate, more or less pressed together;
- surface of left and right lobes of dorsal sclerite in apical half with numerous scale- and/or spinula-like structures distinctly visible in lateral views;
- elytron with number of striae and degree of their development very variable among species and within one species: (0–11)+(0–1) meaning (dorsal striae)+(submarginal stria); usual patterns 11+1, (10–11)+(0–1), and 6+(0–1).

The other morphological characters of the group representatives are in correspondence with the diagnosis and morphological description of the genus (Shaverdo et al. 2023) that can be supplemented with some general characters of the surface sculpture of the head and pronotum and more detailed description of the ventral surface sculpture, which is similar in all studied New Guinean species:

**Table 1.** Checklist, body size, number of elytral striae, and distribution of the *Austrelatuspapuensis* group species. IN: Indonesia, PNG: Papua New Guinea, EHL: Eastern Highlands, NCD: National Capital District, SHL: Southern Highlands.

	Species	TL, mm	Elytral striae	Distribution
1	*A.aiyurensis* sp. nov.	6.5–7.6	11+0	PNG: EHL
2	*A.asteios* sp. nov.	5.7–6.6	11+1	IN: West Papua: Manokwari
3	*A.bewaniensis* sp. nov.	6.9–7.8	11+1	IN: Papua: Biak Numfor, Sarmi, Jayapura; PNG: Sandaun
4	*A.bosaviensis* sp. nov.	7.25–8.3	11+1	PNG: SHL
5	*A.bundunensis* sp. nov.	7.1–7.9	(0–10)+(0–1)	PNG: Morobe
6	*A.centralensis* sp. nov.	7–8	(6–10)+1	PNG: Central
7	*A.craterensis* sp. nov.	6.4	11+0	PNG: Simbu
8	*A.decoris* sp. nov.	5.9	11+1	IN: Papua: Mimika
9	*A.dekai* sp. nov.	6.45–7.7	(10–11)+(0–1)	IN: Papua: Mimika, Pegunungan Bintang, Yahukimo; PNG: SHL
10	*A.epicharis* sp. nov.	5.1–6.35	11+1	IN: Papua: Aru Islands, Mimika, Nabire, Yahukimo; West Papua: Kaimana, Raja Ampat; PNG: SHL, Central
11	*A.flavocapitatus* sp. nov.	5.4–6.1	(10–11)+1	PNG: East Sepik
12	*A.fuscus* sp. nov.	6.4	10+1	PNG: Central
13	*A.gestroi* (Régimbart, 1892)	5.8–6.8	(10–11)+1	PNG: Central, Morobe, NCD, ?East New Britain
14	*A.herzogensis* sp. nov.	5.65–6.7	(10–11)+0	?IN: West Papua: Yapen Islands; PNG: East Sepik, Madang, Morobe
15	*A.inconstans* sp. nov.	6.7–8.2	(0–10)+(0–1)	IN: Papua: Nabire, Puncak; West Papua: Kaimana, Teluk Wondama
16	*A.iriatoi* sp. nov.	4.95–5.55	11+1	IN: Papua: Puncak
17	*A.kalibumi* sp. nov.	6.1–6.7	(10–11)+0	IN: Papua: Nabire
18	*A.kebarensis* sp. nov.	5.1–5.7	6+1	IN: West Papua: Manokwari
19	*A.kokodensis* sp. nov.	5.6–5.65	11+(0–1)	PNG: Central
20	*A.leptos* sp. nov.	4.4–4.5	0+0	PNG: Sandaun
21	*A.loloki* sp. nov.	5	11+1	PNG: NCD
22	*A.lopintolensis* sp. nov.	4.7	6+1	IN: West Papua: Raja Ampat (Waigeo)
23	*A.luteomaculatus* (Guignot, 1956)	5.55–6.6	6+(0–1)	PNG: Gulf, Madang, Morobe
24	*A.madangensis* sp. nov.	6.8–8.15	(10–11)+1	PNG: EHL, East Sepik, Madang
25	*A.maindai* sp. nov.	5.4–5.7	11+1	IN: Papua, Sarmi
26	*A.mamberamo* sp. nov.	7.5–7.9	11+1	IN: Papua: Mamberamo Raya
27	*A.mianminensis* sp. nov.	5.6–6.4	11+1	PNG: Sandaun, ?Madang
28	*A.miltokarenos* sp. nov.	7.3–8.2	(6–11)+1	PNG: Morobe
29	*A.noiadi* sp. nov.	7.9	11+1	IN: Papua: Mamberamo Raya
30	*A.normanbyensis* sp. nov.	6.5–7	6+1	PNG: Milne Bay Province (Normanby Island)
31	*A.ohu* sp. nov.	4.35–5	6+(0–1)	PNG: Madang
32	*A.papuensis* (Balfour-Browne, 1939)	6.9–7.8	(10–11)+1	PNG: Central, EHL, Morobe
33	*A.posmani* sp. nov.	7–7.7	11+1	PNG: Central
34	*A.procerus* sp. nov.	4.5–4.55	(0–6)+0	IN: West Papua: Sorong
35	*A.pseudogestroi* sp. nov.	6.9	11+1	PNG: NCD
36	*A.pseudomianminensis* sp. nov.	5.3–5.5	11+1	IN: Papua: Puncak
37	*A.robustus* sp. nov.	7.45–7.9	11+1	PNG: Central, Madang
38	*A.sararti* sp. nov.	5.7	11+1	IN: West Papua: Teluk Wondama (Wandammen)
39	*A.sumokedi* sp. nov.	4.85	11+1	IN: Papua: Mimika
40	*A.wanangensis* sp. nov.	5.3	11+1	PNG: Madang, Sandaun
41	*A.wasiorensis* sp. nov.	5.95–6.4	11+1	IN: West Papua: Teluk Wondama (Wandammen)
42	*A.wasurensis* sp. nov.	5.4–6.3	11+1	IN: Papua: Merauke
43	*A.weigeli* sp. nov.	6.4–6.6	11+1	PNG: East New Britain
44	*A.yamurensis* sp. nov.	5.6–5.8	6+0	IN: West Papua: Kaimana
45	*A.yeretuar* sp. nov.	4.8–5.55	11+1	IN: Papua: Nabire
46	*A.xanthocephalus* (Régimbart, 1899)	4.7–7.1	6+(0–1)	IN: West Papua: Fak-Fak, Manokwari, Raja Ampat, Sorong, South Manokwari, South Sorong, Tambrauw
46a	*A.xanthocephalusnabirensis* ssp. nov.	6.1–7.1	(0–6)+0	IN: Papua: Nabire

Head with a row of coarse setiferous punctures along inner margin of each eye and a short row or just a few punctures at frontal angle of each eye; a longer punctural row forms a more or less strongly impressed fronto-clypeal depression at each head side. Pronotum with a row of setiferous punctures along pronotal margins, absent in posterior middle, generally less distinct when strioles present; disc of pronotum with distinct or indistinct, short or long longitudinal median scratch.

Ventral side with very fine punctation, almost invisible on metaventrite and metacoxae and more distinct but sparse on abdominal ventrites; abdominal ventrite 6 with distinct punctation, sparse medially and forming denser area at each lateral side; prosternum without microreticulation, smooth medially, with very sparse punctation; metaventrite and metacoxae with distinct microreticulation; on abdominal ventrites microreticulation slightly finer; metacoxal plates with numerous, distinctly impressed, longitudinal strioles, abdominal ventrites 1 and 2 with numerous, long, longitudinal strioles; on abdominal ventrites 3 and 4 strioles turn to middle, sometimes almost horizontal, sparser or absent medially and more distinct laterally; abdominal ventrites 5 and 6 usually without strioles, abdominal ventrite 5 sometimes with oblique strioles at its anterior margin, abdominal ventrite 6 very seldom with a few small strioles at its sides, e.g., for the *A.papuensis* group, it was observed in *A.decoris* sp. nov., *A.inconstans* sp. nov., and *A.procerus* sp. nov.

### ﻿﻿Species descriptions

#### ﻿﻿1. Austrelatus aiyurensis sp. nov.

Taxon classificationAnimaliaColeoptera Dytiscidae

604F18ED-56B7-5644-B4F9-926CA0C0A2CF

https://zoobank.org/EF4E1E21-62E2-4B81-98F0-801AC03DBD1B

Figs 29 , 80 , 107

##### Type locality.

Papua New Guinea: Eastern Highlands Province, Aiyura, 06°21.131'S, 145°54.398'E, 1670 m a.s.l.

##### Type material.

***Holotype***: male “3189” [green label], “Papua New Guinea: Eastern Highlands, Aiyura, 1670m, 5.iv.2006, 06.21.131S 145.54.398E, Balke & Sagata (PNG 32)” (ZSM). ***Paratypes***: 2 males, 3 females with the same label as the holotype (NHMW, ZSM). 2 males “Papua New Guinea: Eastern Highlands, Aiyura creek, 1670m, 20.iv.2006, 06.21.131S 145.54.398E, John & Balke (PNG 70)” (ZSM).

##### Description.

***Body size and form***: Beetle large, oblong-oval to elongate (Fig. 29).

***Measurements***: TL 6.5–7.6 mm, TL-H 5.9–6.95 mm, MW 2.9–3.6 mm, TL/MW 2.11–2.24; PL 0.9–1.1 mm, PW 2.6–3.05 mm, PL/PW 0.35–0.36; DBE 1.15–1.3 mm, DBE/PW 0.43–0.44. ***Holotype***: TL 7.6 mm, TL-H 6.95 mm, MW 3.6 mm, TL/MW 2.11; PL 1.1 mm, PW 3.05 mm, PL/PW 0.36; DBE 1.3 mm, DBE/PW 0.43.

***Colouration***: Dorsally piceous, usually with yellowish red head, pronotal sides and at elytral apex between striae (Fig. 29).

Head yellowish red to brown, narrowly darker behind eyes. Pronotum brown to piceous on disc and reddish to yellowish red on sides, especially at anterior angles; in teneral species, gradually paler (to yellowish red) to sides. Elytron brown to piceous, without basal band, sometimes with small, faint, reddish spot at shoulder, but broadly yellow between striae in apical 1/2, sometimes even to shoulder spot laterally, and reddish along suture. Scutellum reddish to brown. Antennae, other head appendages, and pro- and mesolegs yellowish red, metalegs darker, all legs darker distally. Ventral side brown, yellowish red to reddish on head, prosternum, lower margin of metacoxae and medially and laterally on abdominal ventrites 1–4. ***Note***: the holotype is a sequenced specimen; such specimens are usually darker.

***Surface sculpture***: Elytron with 11 complete striae, submarginal stria absent: 11+0 (Fig. 29).

Head usually without or with single strioles between eyes, with more or less regular, dense punctation (spaces between punctures 1–3× size of punctures); punctures relatively coarse (usually diameter of punctures equal to diameter of microreticulation cells); microreticulation distinct. Pronotum with numerous strioles, with punctation finer than on head and microreticulation fine. Elytron with 11 complete dorsal striae; submarginal stria absent. Elytron with fine, sparse punctation and fine microreticulation.

***Structures***: Head relatively broad. Pronotum trapezoid, its lateral margins convergent anteriorly. Base of prosternum narrowly rounded anteriorly, convex medially; blade of prosternal process relatively broad.

***Male***: Protibia straight, not modified. Proclaws long, subequal in length. Median lobe of aedeagus robust, with two lobes of dorsal sclerite subequal in length, with pointed apexes and covered with scale-like structures; left dorsal lobe distinctly concave subapically, with long, narrow apical crest and apex slightly thickened at its tip, also with lateral side concave medially, above this concavity with small convex area covered with spinula-like structures; right dorsal lobe with large, elongate membranous area medially; lobes of ventral sclerite pressed together between lobes of dorsal sclerite, but partly visible in left lateral view; left ventral lobe with relatively broad, elongate sclerotised area, with pointed apex, situated in left part of lobe and mostly visible in left lateral view; membranous right part of left ventral lobe conjoined with sclerotised area and has apical tuft of long setae; right ventral lobe membranous, long and relatively broad. Parameres with dense, long setae occupying slightly more than half of dorsal margin (Fig. 80).

***Female***: Some females with more numerous strioles on head and pronotum than in males.

##### Variability.

There is a variation in body shape, dorsal colouration and striolation described above.

##### Affinities.

The species belongs to the large species with robust median lobe, covered mainly with scale-like structures, e.g., *A.centralensis* sp. nov. and *A.normanbyensis* sp. nov. However, it is well-recognisable by its elytral colouration without basal band, 11+0 elytral striation, relatively narrow body shape, and median lobe with left dorsal lobe distinctly concave subapically and having convex area with spinula-like structures medially.

##### Etymology.

The species is named after Aiyura creek. The name is an adjective in the nominative singular.

##### Distribution.

Papua New Guinea: Eastern Highlands Province. The species is known only from the type locality (Fig. 107).

##### Habitat.

The species was collected in rest pools of a temporary stream.

#### ﻿﻿2. Austrelatus asteios sp. nov.

Taxon classificationAnimaliaColeoptera Dytiscidae

B1473A18-D168-50B2-97A9-8F1D9DF84E2B

https://zoobank.org/D024C45D-C054-4087-B4FD-C97B7CF02FEF

Figs 57 , 105 , 108

##### Type locality.

Indonesia: West Papua Province: Manokwari Regency, Kebar, 0°48'27.9"S, 133°03'33.3"E, 584 m a.s.l.

##### Type material.

***Holotype***: male “Indonesia: Papua Barat, Kebar, shaded deep sandy irritation roadside ditches, 584m, 6.xi.2013,”, “-0.80775253 133.05923529, UNIPA Team (BH031)” (MZB). ***Paratypes***: 102 males, 78 females with the same label as the holotype, one male with an additional green text label “6249” (MZB, NHMW, ZSM). 1 male, 1 female “Indonesia: Papua Barat, Kebar Valley, 596m, 6.v.2015, -0,8406 133,2682, UNIPA Team (BH059)” (ZSM). 1 female “IN: West Papua: Manokwari Reg., on road Manokwari-Kebar, near Munbrani vill., ca. 600 m, 8.V., 00°46'21"S 133°22'53"E, roadside ditch (2015-WP36)” (NHMW). 5 males, 4 females “Indonesia: Papua, Manokwari, 140m, 8.ii.2006, 00.55.752S 133.54.448E, UNIPA Team (BH 09)”, one male with an additional green label “4248” (ZSM).

##### Description.

***Body size and form***: Beetle medium-sized, oblong-oval (Fig. 57).

***Measurements***: TL 5.7–6.6 mm, TL-H 5.1–5.95 mm, MW 2.7–3.2 mm, TL/MW 2.06–2.12; PL 0.8–0.95 mm, PW 2.3–2.7 mm, PL/PW 0.35–0.36; DBE 0.85–1 mm, DBE/PW 0.37–0.38. ***Holotype***: TL 6.15 mm, TL-H 5.55 mm, MW 2.9 mm, TL/MW 2.12; PL 0.9 mm, PW 2.5 mm, PL/PW 0.36; DBE 0.95 mm, DBE/PW 0.38.

***Colouration***: With piceous elytra and pronotum, yellowish red head, pronotal sides, and elytral basal band and yellowish between striae laterally (Fig. 57).

Head yellowish red medially, narrowly piceous behind eyes and brownish anteriorly. Pronotum piceous on disc and broadly yellowish red on sides, especially at anterior angles. Elytron piceous, with rather broad yellowish red basal band, usually extending between striae, especially laterally and yellowish between striae laterally and sometimes additionally apically, seldom whole elytron slightly yellowish, with distinct basal band. Scutellum reddish to piceous. Antennae, other head appendages, and pro- and mesolegs yellow, metalegs slightly darker proximally and distinctly distally. Ventral side yellowish red, darker on metaventrite, metacoxal plates and two last abdominal ventrites.

***Surface sculpture***: Elytron with 11 complete dorsal striae, submarginal stria present, long: 11+1 (Fig. 57).

Head without strioles, with relatively dense punctation (spaces between punctures 1–3× size of punctures); punctures relatively fine (diameter of punctures usually equal to or smaller than diameter of microreticulation cells); microreticulation distinct. Pronotum with numerous, sparse strioles on lateral parts, only middle of disc without strioles, with punctation finer than on head and microreticulation fine. Elytron with 11 complete dorsal striae, striae 1 and 2, seldom 1–3, less strongly impressed dorsally; submarginal stria present, long, often reaching ½ of elytral length. Elytron with fine punctation and microreticulation.

***Structures***: Head relatively broad. Pronotum trapezoid, its lateral margins convergent anteriorly. Base of prosternum rounded anteriorly, strongly convex medially; blade of prosternal process narrow.

***Male***: Protibia straight, not modified. Proclaws relatively long, simple, subequal in length. Median lobe of aedeagus rather robust, apically thick, with two lobes of dorsal sclerite with broadly pointed apexes, more or less straight and subequal in length; left dorsal lobe with lateral margin not beaded, rather strongly concave at its apical 1/4; lateral side of left dorsal lobe completely covered with tiny spinulae situated in groups on scale-like structures; right dorsal lobe covered with large scale-like structures, with distinct median membranous area; lobes of ventral sclerite partly sclerotised, long, slightly visible in left lateral view; left ventral lobe with elongate, strong sclerotised area, with curved apex and slightly sclerotised part distinctly shorter, with dense setae apically; right ventral lobe longer than left one, partly sclerotised, with apex curved left and covered with setae sometimes distinctly sticking out in left lateral view. Parameres with dense, relatively long setae occupying slightly more than half of dorsal margin (Fig. 105).

***Female***: As males.

##### Affinities.

The species belong to the medium-sized species with median lobe of the aedeagus that has scale- and spinula-like structures on the left dorsal lobe and among them, to the species with surface of left dorsal lobe completely covered by scale- and spinula-like structures in left lateral and ventral views. Based on body shape and size and general shape of the median lobe, it is similar and probably closely related to *A.epicharis* sp. nov. but differs from it in paler elytral colouration, more robust median lobe, and left dorsal lobe of the median lobe with apex thicker and lateral margin more strongly concave apically and therefore ventral lobes more visible in left lateral view. See also under *A.yeretuar* sp. nov.

##### Etymology.

The species name is a Greek adjective meaning “nice, pretty” and refers to the nice yellowish red dorsal pattern of the species.

##### Distribution.

Indonesia: West Papua Province: Manokwari Regency (Fig. 108).

##### Habitat.

The species was collected in shaded, deep, sandy irritation roadside ditches.

#### ﻿﻿3. Austrelatus bewaniensis sp. nov.

Taxon classificationAnimaliaColeoptera Dytiscidae

FD403403-C0CC-593B-92C5-99E67A2AA140

https://zoobank.org/7D8151FC-9022-4367-89A1-B72A6096CFA5

Figs 36 , 86 , 108

##### Type locality.

Papua New Guinea: Sandaun Province, Bewani Mts, 03°05.130'S, 141°10.227'E, 200–300 m a.s.l.

##### Type material.

***Holotype***: male “Papua New Guinea: Sandaun, Bewani Stn., stream@base of Bewani Mts., 200–300m, 12.iv.2006, nr. 03.05.130S 141.10.227E, Balke & Sagata (PNG 37)” (MZB). ***Paratypes*: *PNG*: *Sandaun***: 4 males, 2 females with the same labels as the holotype (MZB, NHMW, ZSM). 2 females “Papua New Guinea: Sandaun, Bewani Stn., forest puddles @ base of Bewani Mts., 300 m, 12.iv.2006, nr. 03.05.130S 141.10.227E, Balke & Sagata (PNG 38)”, one with an additional green label “3215” (ZSM). ***IN*: *Papua*: *Jayapura Regency***: 1 male “Dutch New Guinea: Humbolt Bay [Yos Sudarso Bay] Dist. Pukusam Dist. West of Tami River. Vi.1937.” (BMNH). ***Sarmi Regency***: 1 male, 1 female “Indonesia: Papua, Sarmi, Waaf, N Foja Mts, waterfall in forest, 120m, 23.ix.2014, -2,3317 138,7500, Tim UNCEN: Balke & Menufandu (Pap031)”, male with “6465” [green text] (ZSM). 2 males, 4 females “Indonesia: Papua, Foja Mountains N foot, N Waaf vill., pondok, 150m, 4.-7.vi.2016,”, “-2.06142 138.743949, Sumoked (Pap061)” (NHMW, ZSM). ***Biak Numfor Regency***: 1 male “Indonesia: Papua, Biak, Nimbotong Nimbokramp, 110m 21.iv.2006, Tindige leg.”, “3157” [green label] (ZSM). 1 male “Indonesia: Papua, Biak, Nimbotong Nimbokramp, 110m 21.iv.2006, Tindige”, “1325” [green label] (ZSM). 3 males, 4 females “Indonesia: Papua, Biak, Nimbotong Nimbokramp, 110m 21.iv.2006, Tindige” (NHMW, ZSM).

##### Additional material.

***IN*: *Papua*: *Pegunungan Bintang Regency***: 1 male “Indonesia: Papua, Dekai, upper Brazza, 273m, 2./3.vi.2015, -4,741084724 139,654211075976, Sumoked (Pap044)” (ZSM). ***Note***: a teneral specimen.

##### Description.

***Body size and form***: Beetle large, oblong-oval (Fig. 36).

***Measurements***: TL 6.9–7.8 mm, TL-H 6.35–7.1 mm, MW 3.25–3.6 mm, TL/MW 2.12–2.23; PL 0.95–1.15 mm, PW 2.85–3.2 mm, PL/PW 0.33–0.37; DBE 1.2–1.3 mm, DBE/PW 0.4–0.42. ***Holotype***: TL 7.5 mm, TL-H 6.8 mm, MW 3.4 mm, TL/MW 2.21; PL 1.15 mm, PW 3.1 mm, PL/PW 0.37; DBE 1.3 mm, DBE/PW 0.42.

***Colouration***: Dorsally piceous, with yellowish red to reddish head, pronotal sides, and elytron with narrow, often faint yellowish red to reddish basal band and yellow apex (Fig. 36).

Head yellowish red to reddish, narrowly darker behind eyes. Pronotum piceous on disc and gradually paler (to yellowish red) to sides. Elytron piceous, with narrow yellowish red to reddish basal band, sometimes broadened between striae making base of elytron yellowish red or sometimes basal band faint, very narrow or even absent; elytral apex broadly or narrowly yellowish red, seldom broadened between striae making elytron yellowish red laterally or even in apical 1/2, striae brown to piceous. Scutellum yellowish red to brown. Antennae, other head appendages, and pro- and mesolegs yellowish red to reddish, metalegs darker, all legs darker distally. Ventral side reddish brown, paler on prosternum.

***Surface sculpture***: Elytron with 11 dorsal striae, stria 1 shortly reduced basally, submarginal stria present: 11+1 (Fig. 36).

Head without strioles, with dense punctation (spaces between punctures 1–3× size of punctures); punctures relatively fine (usually diameter of punctures equal to or smaller than diameter of microreticulation cells); microreticulation distinct. Pronotum with few to several strioles posterolaterally, more numerous in females, with punctation finer than on head and microreticulation fine. Elytron with 11 dorsal striae: stria 1 shortly reduced basally, seldom striae 1–3 reduced basally; submarginal stria present. Elytron with fine, sparse punctation and fine microreticulation.

***Structures***: Head relatively broad. Pronotum trapezoid, its lateral margins convergent anteriorly. Base of prosternum rounded anteriorly, convex medially; blade of prosternal process relatively narrow.

***Male***: Protibia more or less straight: its ventral margin slightly curved proximally. Proclaws long, subequal in length, anterior claw thicker subapically and more strongly curved downwards than posterior one and due to this with a median incision on its inner margin. Median lobe of aedeagus robust, with two lobes of dorsal sclerite covered with scale-like structures; left dorsal lobe without distinct median concavity and its whole apical 1/2 broad, more or less evenly tapering to small, curved downwards, slightly truncate apex bearing crest; scale-like structures of left dorsal lobe large in its apical 1/2; right dorsal lobe shorter than left one, broad, with pointed apex and membranous area medially; lobes of ventral sclerite partly sclerotised, pressed together between lobes of dorsal sclerite, not visible in left lateral view due to broad, not concave left dorsal lobe; left ventral lobe consists of two parts: left sclerotised area and less sclerotised right part; sclerotised area with elongate and broad basal 1/2 and long, thread-like apical 1/2; less sclerotised right part long and broad, with its apical part densely covered with long setae; right ventral lobe partly sclerotised, long and broad. Parameres with dense, long setae occupying slightly more than half of dorsal margin (Fig. 86).

***Female***: Trimorphic: 1) as male but with much more numerous pronotal strioles, 2) matt, with pronotum and elytron very densely covered with fine longitudinal strioles, and 3) less matt with strioles partly absent on pronotal and elytral discs, sometimes completely absent on elytron, but with strong dorsal punctation and microreticulation, especially on elytron.

##### Variability.

There is a variation in dorsal colouration and striolation as described above. The male from Dekai (see Additional material) is teneral but most likely belongs to *A.bewaniensis* sp. nov. It is not *A.dekai* sp. nov. according to shape of the male proclaws and median lobe though the latter is difficult clearly to interpret due to weak sclerotisation. However, the specimen differs from all other specimens of *A.bewaniensis* sp. nov. in reduced elytral striation: dorsal stria 1 almost completely reduced, present only as single striole in apical 1/2, striae 3, 5, 7, 9 absent in basal 1/2 and present in apical one as striae or strioles. More material from the area is necessary to clarify specimen affiliation and range of elytral striation variability of the species.

##### Affinities.

The species belongs to the large species with robust median lobe, covered mainly with scale-like structures. Among them, it is distinguished by rather dark dorsal colouration and 11+1 elytral striation, and, especially, left dorsal lobe of the median lobe without median concavity and its apical 1/2 covered with large scale-like structures, very broad, evenly tapering to small, curved downwards, slightly truncate apex bearing crest.

##### Etymology.

The species is named after Bewani Mountains. The name is an adjective in the nominative singular.

##### Distribution.

Indonesia: Papua Province and Papua New Guinea: Sandaun Province (Fig. 108).

##### Habitat.

The species was collected in the different forest water bodies: a stream, puddles, and near a waterfall.

#### ﻿﻿4. Austrelatus bosaviensis sp. nov.

Taxon classificationAnimaliaColeoptera Dytiscidae

CD1FCC63-D3A2-54F7-9FAD-8B5EF619655B

https://zoobank.org/66A2C27D-5AE8-434A-8F22-5CC9FD46A9F0

Figs 31 , 82 , 107

##### Type locality.

Papua New Guinea: Southern Highlands Province, Bosavi Mt, 06°28'S, 142°50'E, 700 m a.s.l.

##### Type material.

***Holotype***: male “Collection Naturhistorisches Museum Basel”, “Papua New Guinea S. Highlands Prov. L.Cizek lgt.”, “Mt Bosavi, 700m 142°50'E 6°28'S 20–27.VI.1999” (NHMB). ***Paratypes***: 18 males, 16 females with the same labels as the holotype (NHMB, NHMW, ZSM).

##### Description.

***Body size and form***: Beetle large, oblong-oval (Fig. 31).

***Measurements***: TL 7.25–8.3 mm, TL-H 6.65–7.5 mm, MW 3.5–4.1 mm, TL/MW 2.02–2.07; PL 1.05–1.3 mm, PW 3.05–3.6 mm, PL/PW 0.34–0.36; DBE 1.2–1.4 mm, DBE/PW 0.39. ***Holotype***: TL 8.2 mm, TL-H 7.4 mm, MW 3.95 mm, TL/MW 2.08; PL 1.25 mm, PW 3.45 mm, PL/PW 0.36; DBE 1.35 mm, DBE/PW 0.39.

***Colouration***: Dorsally piceous, with reddish head, pronotal sides and elytral basal band (Fig. 31).

Head dark yellowish red to reddish, narrowly darker behind eyes. Pronotum dark brown to piceous on disc and sometimes gradually paler (to dark yellowish red) to sides or sides broadly reddish, in contrast to piceous disc. Elytron dark brown to piceous, with narrow, dark yellowish red to reddish basal band differently developed: short, present in shoulder area, faint or distinct at whole base length. Scutellum reddish brown to piceous. Antennae, other head appendages, and pro- and mesolegs reddish, metalegs darker, all legs darker distally. Ventral side reddish brown, darker on posterior margins of abdominal ventrites 1–5 and on abdominal ventrite 6.

***Surface sculpture***: Elytron with 11 complete striae, submarginal stria present: 11+1 (Fig. 31).

Head without strioles, with dense punctation (spaces between punctures 1–3× size of punctures); punctures coarse (usually diameter of punctures larger than diameter of microreticulation cells); microreticulation distinct. Pronotum with strioles posterolaterally, with punctation finer than on head and microreticulation fine. Elytron with 11 dorsal striae, stria 1 shortly or distinctly reduced basally, sometimes absent in basal 1/2; submarginal stria present. Elytron with fine, sparse punctation and fine microreticulation.

***Structures***: Head relatively broad. Pronotum trapezoid, its lateral margins convergent anteriorly. Base of prosternum rounded anteriorly, convex medially; blade of prosternal process relatively narrow.

***Male***: Protibia slightly modified: its ventral margin slightly curved proximally. Proclaws long and straight, subequal in length, anterior claw medially thicker than posterior one and due to this with a shallow subproximal incision on its inner margin. Median lobe of aedeagus very robust, with two lobes of dorsal sclerite covered with scale-like structures; left dorsal lobe very strongly concave medially, with apical 1/2 very broad and apex slightly rounded and bearing small but rather distinct crest; right dorsal lobe shorter, narrower, more or less evenly tapering to pointed apex, with small membranous area medially; lobes of ventral sclerite mostly visible in left lateral view; left ventral lobe consists of two parts: left sclerotised area and less sclerotised right part; sclerotised area with elongate and broad basal 1/2 (visible in left lateral view) and long, thread-like apical 1/2 (not visible); less sclerotised right part long and broad, with its apical part densely covered with long setae (partly visible in left lateral view); right ventral lobe mostly sclerotised, long and broad (partly visible in left lateral view). Parameres with dense, long setae occupying slightly more than half of dorsal margin (Fig. 82).

***Female***: As male.

##### Variability.

There is an insignificant variation in dorsal colouration described above.

##### Affinities.

The species belongs to the large species with robust median lobe, covered mainly with scale-like structures. Among them, it is very similar to *A.robustus* sp. nov. and differs from it in narrower, straighter male anterior claw with inner margin incision situated more proximally and distinctly broader apical 1/2 of the left dorsal lobe of the median lobe. See also under *A.robustus* sp. nov.

##### Etymology.

The species is named after Bosavi Mountain. The name is an adjective in the nominative singular.

##### Distribution.

Papua New Guinea: Southern Highlands Province, Bosavi Mt area (Fig. 107).

##### Habitat.

Unknown.

#### ﻿﻿5. Austrelatus bundunensis sp. nov.

Taxon classificationAnimaliaColeoptera Dytiscidae

322B9FB0-26F8-5CBE-BBC2-75F4125D00FA

https://zoobank.org/5E16A2A5-B1ED-44FF-A81F-306748A53480

Figs 11 , 12 , 64 , 107

##### Type locality.

Papua New Guinea: Morobe Province, Herzog Mts, Bundun, 06°51.598'S, 146°37.07'E, 700–800 m a.s.l.

##### Type material.

***Holotype***: male “Papua New Guinea: Morobe, Herzog Mts., Bundun, 700–800m, 2.iv.2006, 06.51.598S 146.37.07E, Balke & Sagata (PNG 27)” (ZSM). ***Paratypes***: 16 males, 10 females with the same label as the holotype, three males additionally with green labels “3198”, “3199”, and “3228” (NHMW, ZSM). 1 male “3237” [green label], “Papua New Guinea: Morobe, Huon Pen., rd to Kwapsanek, 250m, 31.iii.2006, 06.30.270S 146.59.581E, Balke & Sagata (PNG 24)” (ZSM). 1 female “3208” [green label], “Papua New Guinea: Morobe, Huon Pen., rd to Kwapsanek, 460m, 31.iii.2006, 06.32.736S 146.59.616E, Balke & Sagata (PNG 26)” (ZSM).

##### Description.

***Body size and form***: Beetle large, with oblong-oval habitus (Figs 11, 12).

***Measurements***: TL 7.1–7.9 mm, TL-H 6.5–7.1 mm, MW 3.4–3.7 mm, TL/MW 2.08–2.14; PL 1–1.15 mm, PW 2.9–3.25 mm, PL/PW 0.34–0.35; DBE 1.2–1.3 mm, DBE/PW 0.4–0.41. ***Holotype***: TL 7.7 mm, TL-H 7 mm, MW 3.7 mm, TL/MW 2.08; PL 1.1 mm, PW 3.2 mm, PL/PW 0.34; DBE 1.3 mm, DBE/PW 0.41.

***Colouration***: With piceous elytra and pronotum and yellowish red head, pronotal sides, and basal band on elytron (Figs 11, 12).

Head yellow to yellowish red, narrowly dark brown behind eyes. Pronotum dark brown to piceous on disc and gradually paler (to yellow) on sides. Elytron dark brown to piceous, with narrow yellow to yellowish red basal band, sometimes with yellowish red apex, seldom yellowish red line along suture too. Scutellum yellowish red to brown. Antennae, other head appendages, and pro- and mesolegs yellow to yellowish red, metalegs darker, all legs darker distally. Ventral side reddish brown.

***Surface sculpture***: Dorsal elytral striation very variable, submarginal stria usually absent or present as a short stria or few short strioles apically: (0–10)+(0–1) (Figs 11, 12).

Head without strioles, with relatively dense punctation (spaces between punctures 1–3× size of punctures); punctures relatively coarse (diameter of punctures larger than or equal to diameter of microreticulation cells); microreticulation distinct. Pronotum with strioles laterally, with punctation and microreticulation slightly finer than on head. Elytron with striation very variable: from without striae, with three puncture lines, with up to ten dorsal striae (as in holotype), of which striae 3, 5–10 usually complete, striae 1 and 2 always strongly reduced basally; submarginal stria usually absent or present as a short stria or few short strioles apically. Elytron with punctation distinctly finer than on pronotum and fine microreticulation.

***Structures***: Head relatively broad. Pronotum trapezoid, its lateral margins distinctly convergent anteriorly. Base of prosternum narrowly rounded anteriorly, distinctly convex medially; blade of prosternal process relatively broad.

***Male***: Protibia more or less straight, not modified. Proclaws long, slightly curved, subequal in size and form. Median lobe of aedeagus robust, with two lobes of dorsal sclerite unequal in length and shape, both covered with scale-like structures; left dorsal lobe broader apically and shorter than right dorsal lobe, with a distinct median concavity and its whole apical 1/2 narrowed, its proximal part very large, and its apex more or less straight, with a distinct crest; right dorsal lobe with apex pointed and membranous area medially; lobes of ventral sclerite sclerotised, pressed together between lobes of dorsal sclerite, but partly visible in left lateral view; left ventral lobe consists of two parts: left sclerotised area (visible as a triangular in left lateral view) and less sclerotised right part (visible in left lateral view); sclerotised area with elongate and broad basal 1/2 and long, thread-like apical 1/2; less sclerotised right part long and broad, with its apical part densely covered with long setae; right ventral lobe long and broad. Parameres with dense, long setae occupying slightly more than half of dorsal margin (Fig. 64).

***Female***: Probably dimorphic: all females from the type locality (PNG27) are matt. The only female without this dense striolation is from the locality PNG26, where it was the only specimen collected. It is characterised by the reduced elytral striae (3 more or less complete dorsal striae, with some strioles between them) and presence of numerous strioles on the pronotal sides.

##### Variability.

The species is very variable in the elytral striation, as described above, within and among populations. The holotype is with 10+1 elytral striae. Most specimens from the type locality (PNG27) have such striation but some of them have stria reduction, one specimen up to 5 dorsal striae. Only male from the locality PNG24 is without elytral striae. The female from the locality PNG26 is 3 more or less complete dorsal striae, with some strioles between them.

##### Affinities.

The species belongs to the large species with robust median lobe, covered mainly with scale-like structures. Among them, it is similar to *A.inconstans* sp. nov. in size, body form, and dorsal colouration, but distinctly differs from it in shape of the median lobe and simple male proclaws. Also, the species is very similar to *A.miltokarenos* sp. nov. in median lobe shape but has some differences: apex of left dorsal lobe straighter, with stronger apical crest (in left lateral view) and right dorsal lobe with longer, more curved apex and ventral opening broader (in right lateral view). Additionally, *A.bundunensis* sp. nov. distinctly differs from *A.miltokarenos* sp. nov. by presence of a yellowish red basal band on elytron and its smaller, more oval body.

##### Etymology.

The species is named after its type locality, Bundun. The name is an adjective in the nominative singular.

##### Distribution.

Papua New Guinea: Morobe Province, Herzog Mts (Fig. 107).

##### Habitat.

The species was collected in small forest pools.

#### ﻿﻿6. Austrelatus centralensis sp. nov.

Taxon classificationAnimaliaColeoptera Dytiscidae

06C6D287-4FC5-5970-BF93-2BE2236519C4

https://zoobank.org/0083CB16-F998-465B-AB5D-F16D690BAC5F

Figs 20 , 71 , 107

##### Type locality.

Papua New Guinea: Central Province, 09°25'47.5"S, 147°32'59.1"E, 755 m a.s.l.

##### Type material.

***Holotype***: male “Papua New Guinea: Central, 755 m 28.x.2009, S9 25 47.5 E147 32 59.1, Sagata (PNG229)” (ZSM). ***Paratypes*: *PNG*: *Central***: 10 males, 6 females with the same label as holotype (NHMW, ZSM). 1 male, 4 females “Papua New Guinea: Central, Moroka area, Kailaki Wareaga ridge, 768 m, 27.x.2009, S9 25 42.4 E147 31 06.8, Sagata (PNG227)” (ZSM). 2 males “Papua New Guinea: Central, Moreguina [10°00'57"S, 148°28'27"E] 16.viii.2008 Posman (PNG183) (ZSM). 1 male “Papua New Guinea: Central, Moreguina 18.viii.2008 Posman (PNG184)” (ZSM). 1 male, 1 female “Papua New Guinea: Central, Kokoda Trek, 320m, i.2008, [09°]19.236S 147 31.791E, Posman, (PNG 168)” (ZSM). 2 females “Papua New Guinea: Central, Kokoda Trek, 980m, i.2008, [09°]15.933S 147 36.590E, Posman, (PNG 169)” (ZSM). ***NCD***: 2 males “Papua New Guinea: National Capital District, Varirata NP, 600m, 16.xii.2007, 09.26.13S 147.22.09E, Balke & Sagata (PNG 159)”, one male with an additional green label “2847” (ZSM).

##### Description.

***Body size and form***: Beetle large, with oblong-oval habitus (Fig. 20).

***Measurements***: TL 7–8 mm, TL-H 6.3–7.2 mm, MW 3.3–3.8 mm, TL/MW 2.11–2.12; PL 1–1.2 mm, PW 2.9–3.4 mm, PL/PW 0.35–0.36; DBE 1.15–1.35 mm, DBE/PW 0.4. ***Holotype***: TL 7.6 mm, TL-H 6.8 mm, MW 3.6 mm, TL/MW 2.11; PL 1.1 mm, PW 3.1 mm, PL/PW 0.36; DBE 1.25 mm, DBE/PW 0.4.

***Colouration***: With piceous elytra and pronotum and yellowish red head, pronotal sides, and basal band on elytron (Fig. 20).

Head yellowish red to red, narrowly piceous behind eyes. Pronotum piceous on disc and gradually paler (to yellowish red) on sides, especially at anterior angles. Elytron piceous, with yellowish red to red basal band, which can be distinct or faint, narrower or broader, especially between striae, sometimes due to this elytron reddish laterally; often with yellowish brown apex. Scutellum piceous. Antennae, other head appendages, and pro- and mesolegs yellowish red, metalegs darker, all legs darker distally. Ventral side reddish brown medially and yellowish red on pro- and metaventrite and abdominal ventrites.

***Surface sculpture***: Dorsal elytral striation variable, usually with six dorsal striae and stria 1 reduced in basal 1/2, submarginal stria present: (6–10)+1 (Fig. 20).

Head without strioles, with relatively dense punctation (spaces between punctures 1–3× size of punctures); punctures relatively coarse (diameter of punctures larger than or equal to diameter of microreticulation cells); microreticulation distinct. Pronotum with strioles laterally (few at posterolateral angles to rather numerous but sparse on sides), with punctation slightly denser and finer than on head and microreticulation fine. Elytron usually with six dorsal striae and some strioles between them, sometimes developed into complete and/or partly complete striae: up to ten dorsal striae; stria 1 always absent in basal 1/2; submarginal stria present, sometimes reduced to short apical strioles. Elytron with very fine, sparse punctation and fine microreticulation.

***Structures***: Head relatively broad. Pronotum trapezoid, its lateral margins distinctly convergent anteriorly. Base of prosternum broadly rounded anteriorly, distinctly convex medially; blade of prosternal process relatively narrow.

***Male***: Protibia more or less straight, not modified; its ventral margin can be slightly curved proximally. Proclaws relatively short, subequal in length, anterior claw slightly thicker and more strongly curved downwards than posterior one and due to this with a median incision on its inner margin. Median lobe of aedeagus robust, with two lobes of dorsal sclerite distinctly narrowed to pointed apexes and covered with scale-like structures; left dorsal lobe longer than right lobe, slightly concave subapically and medially, with apex slightly curved downwards; right dorsal lobe with small membranous area medially; lobes of ventral sclerite sclerotised, pressed together between lobes of dorsal sclerite, but partly visible in left lateral view; left ventral lobe consists of two parts: left sclerotised area (not visible in left lateral view) and less sclerotised right part (visible in left lateral view); sclerotised area with elongate and broad basal 1/2 and long, hair-like apical 1/2; less sclerotised right part long and broad, with its apical part with a tuft of long setae; right ventral lobe long and broad. Parameres with dense, long setae occupying approximately half of dorsal margin; more distally situated setae longer than more proximal ones (Fig. 71).

***Female***: As male.

##### Variability.

There is a variation in the elytral striation and colouration described above.

##### Affinities.

The species belongs to the large species with robust median lobe, covered mainly with scale-like structures. Among them, it is similar to *A.inconstans* sp. nov. and *A.bundunensis* sp. nov. in size, body form, and dorsal colouration, but distinctly differs from them in median lobe with dorsal sclerite lobes distinctly narrowed to pointed apex and elytral striae always present.

##### Etymology.

The species is named after Central Province. The name is an adjective in the nominative singular.

##### Distribution.

Papua New Guinea: Central Province (Fig. 107).

##### Habitat.

Unknown.

#### ﻿﻿7. Austrelatus craterensis sp. nov.

Taxon classificationAnimaliaColeoptera Dytiscidae

24C308F6-6240-5516-8EE5-6026E450942C

https://zoobank.org/3AC4CFFC-3749-4CA1-B01D-E0DD4431E6F0

Figs 28 , 79 , 107

##### Type locality.

Papua New Guinea: Simbu Province, Crater Mts, ca. 06°34'53.5"S, 145°05'26.7"E.

##### Type material.

***Holotype***: male “386” [green label], “PNG: Crater Mts ii.2003, Sagata, MB 386” (ZSM).

##### Description.

***Body size and form***: Beetle medium-sized, oblong-oval (Fig. 28).

***Measurements*: *Holotype***: TL 6.4 mm, TL-H 5.75 mm, MW 3.1 mm, TL/MW 2.07; PL 1 mm, PW 2.8 mm, PL/PW 0.36; DBE 1.2 mm, DBE/PW 0.43.

***Colouration***: Dorsally yellowish-reddish brown, with darker head and pronotum (Fig. 28).

Head brown, gradually paler (to reddish) anteriorly, narrowly darker behind eyes. Pronotum dark brown on disc and gradually paler (to reddish) to sides. Elytron uneven yellowish-reddish brown due to strongly developed yellowish red colouration between striae. Scutellum brown. Antennae, other head appendages, and pro- and mesolegs reddish, metalegs darker, all legs darker distally. Ventral side brown.

***Surface sculpture***: Elytron with 11 complete striae, submarginal stria absent: 11+0 (Fig. 28).

Head without strioles, with dense punctation (spaces between punctures 1–3× size of punctures); punctures coarse (diameter of punctures larger than or equal to diameter of microreticulation cells); microreticulation distinct. Pronotum with strioles on sides, with punctation finer than on head and microreticulation fine. Elytron with 11 complete dorsal striae, stria 1 shortly reduced basally; submarginal stria absent. Elytron with fine, relatively dense punctation and fine microreticulation.

***Structures***: Head relatively broad. Pronotum trapezoid, its lateral margins convergent anteriorly. Base of prosternum rounded anteriorly, convex medially; blade of prosternal process relatively broad.

***Male***: Protibia straight, not modified. Proclaws long, slightly curved, subequal in length. Median lobe of aedeagus robust, with two lobes of dorsal sclerite unequal in length and shape, both covered with scale-like structures; left dorsal lobe more or less evenly tapering to apex, its apex straight, with small but distinct incision and crest; right dorsal lobe with apex pointed and membranous area medially; lobes of ventral sclerite, pressed together between lobes of dorsal sclerite, but partly visible in left lateral view, with sclerotised areas (since there is only the holotype, we could not dissect the median lobe to study structure of the ventral sclerite lobes in detail). Parameres with dense, long setae occupying distinctly more than half of dorsal margin (Fig. 79).

***Female***: Unknown.

##### Affinities.

The species is well distinguishable by its uniform, yellowish-reddish brown elytral colouration, 11+0 elytral striation and smaller body size from species with similar structures of the median lobe: usually, these species have TL > 6.5 mm, e.g., *A.inconstans* sp. nov.; additionally, the left dorsal lobe of the median lobe of *A.craterensis* sp. nov. has a very characteristic apex with small but distinct incision and crest.

##### Etymology.

The species is named after Crater Mountains. The name is an adjective in the nominative singular.

##### Distribution.

Papua New Guinea: Simbu Province. The species is known only from the type locality (Fig. 107).

##### Habitat.

Unknown.

#### ﻿﻿8. Austrelatus decoris sp. nov.

Taxon classificationAnimaliaColeoptera Dytiscidae

8CFA3B29-970B-5353-9E26-B8B83148ECB4

https://zoobank.org/52BB9A5B-7448-40A1-8784-FCA429991CC4

Figs 27 , 78 , 107

##### Type locality.

Indonesia: Papua Province: Mimika Regency, 04°15'07.3"S, 136°38'36.2"E, 149 m a.s.l.

##### Type material.

***Holotype***: male “7889” [green text], “Indonesia: Papua, Kabupaten Mimika, Timika, 149m, 25–30.v.2017,”, “-4.252020° 136.643384°, B.Sumoked (Pap68-Bob06)” (MZB).

##### Description.

***Body size and form***: Beetle medium-sized, elongate (Fig. 27).

***Measurements*: *Holotype***: TL 5.9 mm, TL-H 5.3 mm, MW 2.72 mm, TL/MW 2.17; PL 0.95 mm, PW 2.4 mm, PL/PW 0.4; DBE 1.05 mm, DBE/PW 0.53.

***Colouration***: Dorsally dark brown, with paler head, pronotal sides and elytral base and apex (Fig. 27).

Head reddish, narrowly piceous behind eyes and darker posteromedially. Pronotum dark brown on disc and gradually paler (reddish) on sides. Elytron dark brown, reddish basally, laterally and apically, also with faint reddish colouration between striae so that elytron not looking uniformly dark brown. Scutellum reddish brown. Antennae, other head appendages, and legs reddish, darker distally. Ventral side brown.

***Surface sculpture***: Elytron with 11 complete, relatively strongly impressed striae, submarginal stria present: 11+1 (Fig. 27).

Head without strioles, with distinct, dense punctation (spaces between punctures 1–3× size of punctures); punctures relatively coarse (diameter of punctures slightly larger than or equal to diameter of microreticulation cells); microreticulation distinct. Pronotum without strioles, with punctation finer than on head and fine microreticulation. Elytron with 11 complete, relatively strongly impressed striae, submarginal stria present. Elytron with fine punctation and distinct microreticulation. Abdominal ventrite 6 with distinct punctation, sparse medially and forming denser area at each lateral side and with some strioles medially of this area.

***Structures***: Head large and broad. Pronotum relatively long, its lateral margins rounded, very slightly convergent anteriorly. Base of pronotum slightly narrower than base of elytron, therefore, habitus outline discontinuous. Base of prosternum rounded anteriorly, convex medially; blade of prosternal process relatively broad.

***Male***: Protibia slightly modified; its ventral margin curved proximally. Proclaws relatively long, subequal in length, anterior claw slightly thicker and more strongly curved downwards than posterior one and due to this with a median incision on its inner margin. Median lobe of aedeagus with two lobes of dorsal sclerite unequal in length, covered with scale-like structures; left dorsal lobe with lateral margin slightly concave medially, with apex truncate and slightly curved downwards, having distinct crest; right dorsal lobe broad, shorter than left lobe, with pointed apex and membranous area medially; lobes of ventral sclerite mostly sclerotised, pressed together between lobes of dorsal sclerite, almost invisible in lateral view: tip of setation of less membranous part of left ventral lobe distinctly sticking out (since there is only the holotype, we could not dissect the median lobe to study structure of the ventral sclerite lobes in detail, see e.g., *A.inconstans* sp. nov.). Parameres with long and dense setae occupying approximately half of dorsal margin; more distally situated setae longer than more proximal ones (Fig. 78).

***Female***: Unknown.

##### Affinities.

The species is well-recognisable by its elongate, slightly discontinuous habitus, almost uniformly reddish brown dorsal colouration, strong elytral striation, and characteristic shape of the median lobe.

##### Etymology.

The species name is a Latin adjective meaning elegant and refers its elongate, slightly discontinuous habitus, with rounded pronotal sides.

##### Distribution.

Indonesia: Papua Province: Mimika Regency. The species is known only from the type locality (Fig. 107).

##### Habitat.

Unknown.

#### ﻿﻿9. Austrelatus dekai sp. nov.

Taxon classificationAnimaliaColeoptera Dytiscidae

1D0FD45C-5C80-5B41-B8F7-C109933AEDC2

https://zoobank.org/1DE615B8-46C6-4985-929B-4DF5AD73B16D

Figs 32 , 83 , 107

##### Type locality.

Indonesia: Papua Province: Yahukimo Regency, Dekai, upper Brazza, 04°44'27.9"S, 139°39'15.2"E, 273 m a.s.l.

##### Type material.

***Holotype***: male “Indonesia: Papua, Dekai, upper Brazza, 273m, 2./3.vi.2015, -4,741084724 139,654211075976, Sumoked (Pap044)” (MZB). ***Paratypes*: *IN***: ***Papua*: *Yahukimo Regency***: 11 males, 13 females with the same label as the holotype, 2 males and 2 females with additional green text labels “7214”, “7215”, “7216” and “7217”, respectively (MZB, NHMW, ZSM). ***Pegunungan Bintang Regency***: 4 males, 4 females “Indonesia: Papua, S Ok Sibil, tributary of Digul Riv, 359m, 9.vi.2015, -5,05718389526009 140,722535848617, Sumoked (Pap051)” (MZB, NHMW, ZSM). ***Mimika Regency***: 1 male “Indonesia: Papua, Kabupaten Mimika, Timika, 149m, 25–30.v.2017, 04°15.092'S 136°38.597'E, B. Sumoked (Pap68-Bob06)” (ZSM). 1 male, 1 female “Indonesia: Papua, Kabupaten Mimika, Timika, 149m, 25–30.v.2017,”, “-4.252020° 136.643384°, B.Sumoked (Pap68-Bob06)”, with additional green text labels “7887” and “7888”, respectively (ZSM). ***PNG*: *SHL***: 1 male, 1 female “Collection Naturhistorisches Museum Basel”, “Papua New Guinea S. Highlands Prov. L.Cizek lgt.”, “Mt Bosavi, 700m 142°50'E 6°28'S 20–27.VI.1999” (NHMB).

##### Additional material.

7 males “New Guinea XII-1942 W.G.Bodenstein” (ZSM). Note: teneral specimens.

##### Description.

***Body size and form***: Beetle large, oblong-oval (Fig. 32).

***Measurements***: TL 6.45–7.7 mm, TL-H 5.8–6.95 mm, MW 3.1–3.7 mm, TL/MW 2–2.08; PL 1–1.2 mm, PW 2.8–3.4 mm, PL/PW 0.35–0.36; DBE 1.15–1.35 mm, DBE/PW 0.39–0.41. ***Holotype***: TL 7.2 mm, TL-H 6.5 mm, MW 3.6 mm, TL/MW 2; PL 1.15 mm, PW 3.2 mm, PL/PW 0.36; DBE 1.25 mm, DBE/PW 0.39.

***Colouration***: Dorsally piceous, with yellowish red to reddish head, pronotal sides and usually elytral basal band (Fig. 32).

Head yellowish red to reddish, narrowly darker behind eyes. Pronotum dark brown to piceous on disc and gradually paler (to yellowish red) to sides. Elytron brown to piceous, without basal band or with narrow, yellowish red to reddish basal band, often short, present in shoulder area, faint. Scutellum brown. Antennae, other head appendages, and pro- and mesolegs yellowish red to reddish, metalegs darker, all legs darker distally. Ventral side reddish brown.

***Surface sculpture***: Elytron with 10 or 11 differently reduced striae, submarginal stria present or absent: (10–11)+(0–1) (Fig. 32).

Head without strioles, with dense punctation (spaces between punctures 1–3× size of punctures); punctures relatively fine (usually diameter of punctures equal to or smaller than diameter of microreticulation cells); microreticulation distinct. Pronotum without strioles, with punctation finer than on head and microreticulation fine. Elytron with 10 or 11 differently reduced dorsal striae: stria 1 usually completely reduced, sometimes more or less complete or present as strioles in apical 1/2, striae 2 and 3 usually reduced basally, striae 5, 7, and 10 usually interrupted in basal 1/2; submarginal stria absent or present, seldom as complete stria, usually as apical strioles. Elytron with fine, sparse punctation and fine microreticulation.

***Structures***: Head relatively broad. Pronotum trapezoid, its lateral margins convergent anteriorly. Base of prosternum rounded anteriorly, convex medially; blade of prosternal process broad, weakly convex.

***Male***: Protibia slightly modified: its ventral margin slightly curved proximally. Proclaws long and straight, subequal in length, anterior claw thicker medially than posterior one. Median lobe of aedeagus robust, with two lobes of dorsal sclerite covered with scale-like structures; left dorsal lobe distinctly broader and longer than right dorsal lobe; left dorsal lobe with a distinct median concavity and its whole apical 1/2 narrowed, its apex straight, without distinct crest, sometimes with a weak crest due to small, very shallow tip concavity, more or less evenly rounded; right dorsal lobe with apex pointed and membranous area medially; lobes of ventral sclerite sclerotised, pressed together between lobes of dorsal sclerite, but visible in left lateral view; left ventral lobe consists of two parts (partly visible in left lateral view): left sclerotised area and less sclerotised right part; sclerotised area with elongate and broad basal 1/2 and long, thread-like apical 1/2; less sclerotised right part long and broad, with its apical part densely covered with long setae; right ventral lobe partly sclerotised, long and broad, curved at apex. Parameres with long and dense setae occupying approximately half of dorsal margin; more distally situated setae longer and denser than more proximal ones (Fig. 83).

***Female***: Dimorphic: as male, shiny, and matt, with pronotum and elytron very densely covered with fine longitudinal strioles. Matt forms present in two populations, more numerous than shiny ones: e.g., ratio shiny to with strioles is 11:2 in the type locality. Only matt forms are known from the locality “Pap051”.

##### Variability.

There is a variation in dorsal colouration and striolation described above.

##### Affinities.

The species belongs to the large species with robust median lobe, covered mainly with scale-like structures. Among them, it is similar to *A.inconstans* sp. nov., *A.bundunensis* sp. nov. and *A.miltokarenos* sp. nov. but differs from them in smaller body size, male anterior proclaw long, straight but thicker medially than posterior one and narrower apical 1/2 of the left dorsal lobe of the median lobe.

##### Etymology.

The species is named after its type locality, Dekai Village. The name is a noun in the nominative singular standing in apposition.

##### Distribution.

Indonesia: Papua Province and Papua New Guinea: Southern Highlands Province (Fig. 107).

##### Habitat.

The species was collected side pools of a forest stream.

#### ﻿﻿10. Austrelatus epicharis sp. nov.

Taxon classificationAnimaliaColeoptera Dytiscidae

23C700D8-5D85-593C-83B2-AEE350FD073B

https://zoobank.org/13F8C1E4-59CB-428B-BB42-0C5FCBF963BF

Figs 56 , 106 , 108 , 109 , 110 , 115

##### Type locality.

Indonesia: Papua Province: Nabire Regency, Nabire, Wanggar, Kali Bumi.

##### Type material.

***Holotype***: male “W.-Neuguinea /Paniai Prov./ Wanggar- Kali Bumi / IR 14 30.9 & 1.10.90 leg: Balke & Hendrich” (ZSM). ***Paratypes*: *IN*: *Papua*: *Nabire Regency***: 14 males, 20 females with the same label as the holotype (CLH, ZSM). 3 males, 8 females “IR 90#14: West New Guinea, Nabire→Wanggar, 100m, 30.ix.1990, Balke” (ZSM). 2 males, 8 females “Irian: Nabire – Ikaga [sic! Ilaga], “KM 35”, Seitenstr. nach K. Cemara, 1991, leg. M. Balke” (ZSM). 8 males, 8 females “West New Guinea/Paniai Prov./IR 23 track Nabire-Ilaga km 34 near Topo, 120m, 23.7.1991 leg: Balke & Hendrich” (ZSM). 4 males, 1 female “IRIAN JAYA: Nabire Prov. rd. Nabire – Ilaga, Km 35 Kali Cemara, 100m, 27.9.1997 (IR97#6)” (NHMW). 7 males, 6 females “IRIAN JAYA: Nabire Prov. rd. Nabire – Ilaga, Km 35 Kali Cemara, 100m, 23.10.1997 (IR97#14)” (NHMW). ***Mimika Regency***: 1 female “Indonesia: Papua, Kabupaten Mimika, Timika, 149m, 25–30.v.2017, 04°15.092'S 136°38.597'E, B. Sumoked (Pap68-Bob06)” (ZSM). 9 males, 4 females “Indonesia: Papua, Kabupaten Mimika, 24m, 25–30.v.2017, 04°30.330’”, “E 136°46.53’, B. Sumoked (Pap69-Bob07)” (ZSM). ***Yahukimo Regency***: 2 males, 4 females “Indonesia: Papua, Dekai, upper Brazza, 273m, 2./3.vi.2015, -4,741084724 139,654211075976, Sumoked (Pap044)”, one male with an additional green text label “7221” (ZSM). ***Aru Islands Regency***: 2 males “INDONESIA: Aru Islands, Trangan, 1km E of Ngalgull, 6°48'S 134°4'E, 29.vii.1994, 90m, A.H. Kirk-Spriggs.”, “A.H.Kirk-Spriggs Maluku Tenggara Coll. NMW.Z.1994.061”, “Temporary/Sago pools, Primary mixed forest.” (CLH, ZSM). 1 female “INDONESIA: Aru Islands, Trangan, 1km E of Ngalgull, 6°48'S 134°4'E, 23–28.vii.1994, 90m, A.H. Kirk-Spriggs.”, “A.H.Kirk-Spriggs Maluku Tenggara Coll. NMW.Z.1994.061”, “Temporary/Sago pools, Primary mixed forest.” (CLH, ZSM). 1 male “INDONESIA, SE Moluccas Aru Isls, Wokam Isl. 17 km NE Wakua vill. 1.-7.ii.2022, S. Jákl leg.”, “coll. Jiří Hájek National Museum Prague, Czech Republic” (NMPC). 1 male “INDONESIA, SE Moluccas ARU ISLS, WOKAM I. 17 km NE Wakua vill. 1–7.II.2022, St. Jakl leg.”, “coll. Jiří Hájek National Museum Prague, Czech Republic” (NMPC). ***West Papua***: ***Kaimana Regency***: 1 male, 3 females “INDONESIA W-PAPUA 50km SE Kaimana, Triton bay, vic. Kamaka vill. S3°49'50”/E134°11'27”, 10–50m, 02.-05.II.2011 A. Skale (006)” (ZSM). 1 male “INDONESIA: W-PAPUA vic. Kaimana, road 17 km NE, S3°31'41”/E133°40'51”, 50m, 31.I.2011 A Skale (003)”, “4431” [green label] (ZSM). 47 males, 60 females “IRIAN JAYA: Fak Fak dist. Lake Yamur area, IV.1998 ca. 50 – 100m, Waldtümpel” (NHMW). ***Raja Ampat Regency*: *Waigeo***: 2 males, 3 females “W-PAPUA Raja Ampat Prov. Waigeo Isl., Lopintol 0°07'54"S, 130°53'45"E 11.i.2004 leg. A.Skale UWP” (CAS). 1 male “760” [green label], “W Papua: Raja Ampat, Waigeo, Lopintol, 11/2005, A. Skale, MB760” (ZSM). 1 female “762” [green label], “W Papua: Raja Ampat, Waigeo, Lopintol, 11/2005, A. Skale, MB 762” (ZSM). ***Batanta***: 1 male “W-PAPUA Raja Ampat Pr. Yensawai Batanta, 9 km W Ross-River 0°49'23"S 130°35'52"E 17.I.2004 leg. A.Weigel UWP KL” (CAS). 1 male, 1 female “Indonesia: Papua, Batanta Selatan, Wailebet, 100m, 17.ii.2006, inland 00.53.957S 130.39.951E, Tindige & Prativi (BH 15)”, with additional green labels “4252” and “4253” (ZSM). 1 male “Indonesia: Papua, Batanta Selatan, Wailebet, 20m, 16.ii.2006, 00.54.003S 130.39.296E (BH 14)” (ZSM). 6 males, 2 females “Indonesia: Papua, Batanta Utara, 20m, 14.ii.2006, 00.50.125S 130.42.856E (BH 12)” (ZSM). 1 male, 1 female “W-PAPUA, Raja Ampat Pr. Waywesar/Batanta 2 km E, 0°45'17"S 130°48'06"E 18.I.2004, leg. A. Weigel” (CAS). 11 males, 21 females “W-PAPUA Raja Ampat Prov. Batanta Isl. bor., 9 km W Yensawai, Ross-River 0°49'23"S, 130°35'52"E 17.I.2004 leg. A. Skale” (CAS, NHMW). 4 males “W-PAPUA Raja Ampat Prov. Batanta Isl. bor., Yensawai, 0°48'05"S, 130°40'36"E 15.-18.I.2004 leg. A.Skale” (CAS, NHMW). 1 female “W-PAPUA Raja Ampat Prov. Batanta Isl. bor., Arefl, 0°47'24,5"S, 130°42'10"E 16.I.2004 leg. A.Skale” (CAS). 7 males, 2 females “W-PAPUA Raja Ampat Prov. Batanta Isl. bor., Waywesar, 0°45'26"S, 130°46'55"E 12.-15.I.2004 leg. A.Skale UWP” (CAS, NHMW). ***Salawati***: 2 males “W-PAPUA Raja Ampat Prov. Salawati Isl. Or., 2–4 km N Kalobo 01°00'56"S, 131°04'58"E 28.I.2004 leg. A.Skale” (CAS). 1 male, 1 female “W-PAPUA Raja Ampat Prov. 1 km E Kalobo, Wajir Island 01°00'S, 131°04'E 26.I.2004 leg. A.Skale UWP/UWS” (CAS). ***Misool***: 23 males, 19 females “Indonesia: West Papua, Raja Ampat, Misool, v.2015, -1.799575° 129.950446, Prativi” (NHMW, ZSM). ***PNG*: *SHL***: 4 males, 1 female “Collection Naturhistorisches Museum Basel”, “Papua New Guinea S. Highlands Prov. L.Cizek lgt.”, “Mt Bosavi, 700m 142°50'E 6°28'S 20–27.VI.1999” (NHMB, NHMW, ZSM). ***Central***: 6 males, 5 females “Papua New Guinea: Central, Tapini, 870m, 29.x.2007, 08.20.511S 146.59.824E Kinibel (PNG 161)”, one male and one female additionally with green labels “2854” and “2853”, respectively (NHMW, ZSM).

##### Additional material.

2 males (teneral) “Papua New Guinea: Southern Highlands Province, Tari to Komo, Hides Gas basecamp, 1200m, 13.v.2006, 05.55.223'S 142.46.090'E, Balke (PNG 60)”, one male additionally with a green label “3231” (ZSM).

##### Description.

***Body size and form***: Beetle small to medium-sized, oblong-oval (Fig. 56).

***Measurements***: TL 5.1–6.35 mm, TL-H 4.55–5.8 mm, MW 2.45–3.1 mm, TL/MW 2.04–2.8; PL 0.8–0.95 mm, PW 2.1–2.65 mm, PL/PW 0.35–0.38; DBE 0.85–1.1 mm, DBE/PW 0.4–0.42. ***Holotype***: TL 5.6 mm, TL-H 5.1 mm, MW 2.75 mm, TL/MW 2.04; PL 0.85 mm, PW 2.4 mm, PL/PW 0.35; DBE 0.95 mm, DBE/PW 0.4.

***Colouration***: With piceous elytra and pronotum and yellowish red head, pronotal sides, and elytral basal band and apical spot (Fig. 56).

Head yellowish red medially, narrowly piceous behind eyes and brownish anteriorly. Pronotum piceous on disc and broadly yellowish red on sides, especially at anterior angles. Elytron piceous, with distinct yellowish red basal band, usually not reaching shoulders and suture; elytron usually with yellow spot apically sometimes slightly extending laterally. Scutellum reddish to piceous. Antennae, other head appendages, and pro- and mesolegs yellow, metalegs slightly darker proximally and distinctly distally. Ventral side reddish brown, darker on metaventrite, metacoxal plates and two last abdominal ventrites.

***Surface sculpture***: Elytron with 11 complete dorsal striae, submarginal stria present, long: 11+1 (Fig. 56).

Head without strioles, with relatively dense punctation (spaces between punctures 1–3× size of punctures); punctures relatively fine (diameter of punctures usually equal to and smaller than diameter of microreticulation cells); microreticulation distinct. Pronotum with several or rather numerous, sparse strioles on lateral parts, sometimes only middle of disc without strioles, with punctation finer than on head and microreticulation fine. Elytron with 11 complete dorsal striae, sometimes strongly impressed, sometimes less, sometimes stria 1 or striae 1 and 2, seldom 1–3, less strongly impressed dorsally; submarginal stria present, long, often reaching ½ of elytral length. Elytron with fine punctation and microreticulation.

***Structures***: Head relatively broad. Pronotum trapezoid, its lateral margins convergent anteriorly. Base of prosternum rounded anteriorly, strongly convex medially; blade of prosternal process narrow.

***Male***: Protibia straight, not modified. Proclaws relatively long, simple, subequal in length. Median lobe of aedeagus with two lobes of dorsal sclerite with broadly pointed apexes, more or less straight and subequal in length; left dorsal lobe with lateral margin not beaded, slightly concave its apical 1/2; lateral side of left dorsal lobe completely covered with tiny spinulae situated in groups on scale-like structures; right dorsal lobe covered with large scale-like structures, with distinct median membranous area; lobes of ventral sclerite partly sclerotised, long, slightly visible in left lateral view; left ventral lobe with elongate, strong sclerotised area, with curved apex and slightly sclerotised part distinctly shorter, with dense setae apically; right ventral lobe longer than left one, partly sclerotised, with apex curved left and covered with setae sometimes distinctly sticking out in left lateral view. Parameres with long and dense setae occupying approximately half of dorsal margin; more distally situated setae longer and denser than more proximal ones (Fig. 106).

***Female***: As males.

##### Affinities.

The species belong to the medium-sized species with median lobe of the aedeagus that has scale- and spinula-like structures on the left dorsal lobe and among them, to the species with surface of left dorsal lobe completely covered by scale- and spinula-like structures in left lateral and ventral views. Based on body shape and size and general shape of the median lobe, it is similar and probably closely related to *A.asteios* sp. nov. but differs from it in paler elytral colouration, more robust median lobe, and left dorsal lobe of the median lobe with apex thicker and lateral margin more strongly concave apically and therefore ventral lobes more visible in left lateral view. See also under *A.yeretuar* sp. nov.

##### Notes on Copelatinae of the Aru Islands.

*Austrelatusepicharis* sp. nov. is the second copelatine species described from the Aru Islands. The first one is *Copelatushaemorrhoidalis* Régimbart, 1883, which was described based on a single female. We have studied habitus photos of the holotype (RMNH.INS.1487394), which is deposited in the Naturalis Biodiversity Center (former Rijksmuseum van Natuurlijke Historie), Leiden, Netherlands (RMNH), and compared them with images and specimens of *A.epicharis* sp. nov. We believe that they are two different species since 1) thought *C.haemorrhoidalis* has 11+1 elytral striae, striae 2, 4, 6, 8 are reduced in the apical 1/2, and *A.epicharis* sp. nov. never demonstrates elytral stria reduction; 2) *C.haemorrhoidalis* has less prominent yellowish red dorsal colouration and a V-shaped brown spot on head, but head of *A.epicharis* sp. nov. is always uniformly yellowish red medially (Figs 56, 58). Most likely, *C.haemorrhoidalis* belongs to the genus *Austrelatus* but it will be possible to determine with certainty when males of the species are found. Besides these two species, there are two more copelatine species found in the material recently collected from the Aru Islands. They are represented by three females, two of which belong to the *A.neoguineensis* group and one is most likely a *Copelatus* species. Thus, Copelatinae fauna of the Aru Islands includes four different species with the certain record of *A.epicharis* sp. nov. For three other species, more material is necessary to clarify their taxonomic position.

##### Etymology.

The species name is a Greek adjective meaning “beautiful, graceful” and refers to striation and nice yellowish red dorsal pattern of the species.

##### Distribution.

The species is widely distributed in Indonesian part of New Guinea including the small islands; it is also known from Central and SHL provinces of PNG (Fig. 108).

##### Habitat.

All specimens on the track Nabire to Ilaga were collected in shallow (up to 20 cm water depth), shaded or at least partly shaded forest pools and road-side ditches, rich in rotten leaves and twigs (Figs 109, 110). The type specimens were collected in shallow, exposed and flooded meadow puddles and oxbows along Kali (= River) Bumi at Wanggar (Fig. 115). Few specimens were also found in water-filled track hollows. On the Aru Islands, the species was collected in temporary Sago swamp pools, surrounded by primary mixed forest.

#### ﻿﻿11. Austrelatus flavocapitatus sp. nov.

Taxon classificationAnimaliaColeoptera Dytiscidae

03EC2CBE-33BA-53CC-BF00-A3D96946EDEE

https://zoobank.org/95679550-737D-4E55-8AD5-80A6C66AA0B2

Figs 46 , 95 , 108

##### Type locality.

Papua New Guinea: East Sepik Province, Lembena, 04°56.859'S, 143°57.379'E, 335 m a.s.l.

##### Type material.

***Holotype***: male “Papua New Guinea: East Sepik, Lembena, 335m, 10.ix.2009, 04 56.859S 143 57.379E, Ibalim & Pius (PNG251)” (ZSM). ***Paratypes***: 3 males, 2 females with the same label as the holotype (NHMW, ZSM). 3 males, 3 females “Papua New Guinea: East Sepik, Lembena, 198m, 3.ix.2009, 04.56.974S 143.56.995E, Ibalim & Pius (PNG241)” (NHMW, ZSM). 3 females “Papua New Guinea: East Sepik, Lembena, 198m, 3.ix.2009, 04 46 [!] 974S 143.56.995E, Ibalim & Pius (PNG243)” (ZSM). 2 males “Papua New Guinea: East Sepik, Lembena, 136m, 3.ix.2009, 04 56.911S 143.56.870E, Ibalim & Pius (PNG244)” (ZSM). 1 male, 6 females “Papua New Guinea: East Sepik, Lembena, 117m, 8.ix.2009, 04.57.513S 14357.296E, Ibalim & Pius (PNG248)”, one male and four females with additional green text labels “6001”, “5999”, “6000”, “6002”, and “6003”, respectively (ZSM). 1 male, 2 females “Papua New Guinea: East Sepik, Lembena, 110m, 10.ix.2009, 04.57.512S 143.57.366E, Ibalim & Pius (PNG249)”, male with a green text label “6033” (ZSM). 2 females “Papua New Guinea: East Sepik, Lembena, 335m, 10.ix.2009, 04.56.859S 143.59.375E, Ibalim & Pius (PNG250)” (ZSM).

##### Description.

***Body size and form***: Beetle medium-sized, oblong-oval (Fig. 46).

***Measurements***: TL 5.4–6.1 mm, TL-H 5–5.4 mm, MW 2.65–2.9 mm, TL/MW 2.04–2.1; PL 0.85–0.9 mm, PW 2.3–2.5 mm, PL/PW 0.36–0.37; DBE 0.9–0.95 mm, DBE/PW 0.38–0.39. ***Holotype***: TL 5.5 mm, TL-H 5 mm, MW 2.7 mm, TL/MW 2.04; PL 0.85 mm, PW 2.35 mm, PL/PW 0.36; DBE 0.9 mm, DBE/PW 0.38.

***Colouration***: With piceous elytra and pronotum and yellowish red head, pronotal sides, and basal band and large apical spot on elytron (Fig. 46).

Head yellowish red, narrowly piceous behind eyes. Pronotum piceous on disc and yellowish red on sides, especially at anterior angles. Elytron piceous, with distinct, rather broad, yellowish red basal band and large, often laterally extending yellow spot on apex. Scutellum reddish to piceous. Antennae, other head appendages, and pro- and mesolegs yellowish red, metalegs distinctly darker, especially distally. Ventral side reddish brown to brown, paler on pro- and metaventrite and abdominal ventrites 1–3.

***Surface sculpture***: Elytron with (10–11)+1 striae, sometimes striae differently reduced or interrupted (Fig. 46).

Head without strioles, with rather dense punctation (spaces between punctures 1–3× size of punctures); punctures rather coarse (diameter of punctures usually equal to diameter of microreticulation cells); microreticulation distinct. Pronotum without or with few strioles posterolaterally, with punctation finer than on head and microreticulation fine. Elytron with 11 dorsal striae, stria 1 differently reduced, often absent in basal 1/2 and present as few strioles in apical 1/2, seldom completely absent, striae 2 and 3 reduced or interrupted basally, striae 5–7, 9 and 10 sometimes shortly reduced or interrupted basally; submarginal stria present, sometimes reduced to few strioles apically. Elytron with fine punctation and microreticulation.

***Structures***: Head relatively broad. Pronotum trapezoid, its lateral margins convergent anteriorly. Base of prosternum broadly rounded anteriorly, convex medially; blade of prosternal process relatively narrow.

***Male***: Protibia straight, not modified. Proclaws relatively long, slightly curved, subequal in length, anterior claw thicker subapically than posterior one and due to this with a median incision on its inner margin. Median lobe of aedeagus with two lobes of dorsal sclerite subequal in length, more or less straight, with very broadly pointed apexes, apex of left dorsal lobe slightly curved downwards; left dorsal lobe with lateral side covered with numerous, distinct, strong spinula-like structures; lateral margin apically without surface structures, smooth; right dorsal lobe covered with scale-like structures, with distinct median membranous area; lobes of ventral sclerite partly sclerotised, long, subequal in length, partly visible in left lateral view; left ventral lobe with elongate, strong sclerotised area, concave subapically, with apex pointed and slightly curved to right and membranous right part long, thin, apically covered with setae sometimes visible in left lateral view; right ventral lobe with narrow sclerotised area on right margin. Parameres with long and dense setae occupying approximately half of dorsal margin; more distally situated setae longer and denser than more proximal ones (Fig. 95).

***Female***: As males but with stronger dorsal punctation and microreticulation and usually more numerous pronotal strioles.

##### Affinities.

The species belong to the medium-sized species with median lobe of the aedeagus that has scale- and spinula-like structures on the left dorsal lobe and among them, to the species with surface of left dorsal lobe not completely covered by spinula-like structures, upper lateral margin without spinulae, smooth in left lateral and ventral views. Based on shape of the median lobe, it is similar to *A.mianminensis* sp. nov. and especially to *A.pseudomianminensis* sp. nov. (in having lateral margin of left dorsal lobe with edge, below which spinula-like structures situated, more sharp, prominent) but differs from them in more bright dorsal colouration: yellowish red head and lateral sides of pronotum, elytron with broad yellowish red basal band and large apical spot, as well as in less striolated elytron: (10–11)+1 striae, stria 1 strongly reduced basally (visible in traces in apical 1/2), sometimes completely absent, the other striae can be differently reduced. See also under *A.maindai* sp. nov. and *A.kokodensis* sp. nov.

##### Etymology.

The species name is a combination of the Latin words *flavus* (yellow) and *capitatus* (heaving a head) and refers to the distinct yellowish head colouration of the species. The species name is an adjective in the nominative singular.

##### Distribution.

Papua New Guinea: East Sepik Province. The species is known only from Lembena area (Fig. 108).

##### Habitat.

Unknown.

#### ﻿﻿12. Austrelatus fuscus sp. nov.

Taxon classificationAnimaliaColeoptera Dytiscidae

E09D835C-76DD-52C7-85EE-658E51F4220C

https://zoobank.org/C206C98D-7EBE-4FFE-9797-BFA24340800B

Figs 50 , 99 , 108

##### Type locality.

Papua New Guinea: Central Province, Kokoda, 08°53.481'S, 147°43.648'E, 410 m a.s.l.

##### Type material.

***Holotype***: male “Papua New Guinea: Northern, Kokoda, 410m, i.2008, 53.481S 147.43.648E, Posman, (PNG 174)” (ZSM).

##### Description.

***Body size and form***: Beetle medium-sized, oblong-oval (Fig. 50).

***Measurements*: *Holotype***: TL 6.4 mm, TL-H 5.8 mm, MW 3.1 mm, TL/MW 2.07; PL 0.9 mm, PW 2.7 mm, PL/PW 0.33; DBE 0.95 mm, DBE/PW 0.35.

***Colouration***: Dorsally almost uniformly piceous (Fig. 50).

Head dark brown, reddish posteriorly between eyes and anteromedially, narrowly piceous behind eyes. Pronotum piceous, dark brown on sides, reddish at anterior angles. Elytron piceous, yellowish red apically, slightly extending laterally. Scutellum piceous. Antennae, other head appendages, and pro- and mesolegs reddish, metalegs distinctly darker, especially distally. Ventral side piceous, with paler prosternum.

***Surface sculpture***: Elytron with 10+1 striae; submarginal stria present as weak apical strioles (Fig. 50).

Head without strioles, with rather dense punctation (spaces between punctures 1–3× size of punctures); punctures rather coarse (diameter of punctures equal to diameter of microreticulation cells); microreticulation distinct. Pronotum with single strioles at posterior angles, with punctation slightly finer than on head and microreticulation fine. Elytron with ten dorsal striae, striae 1 and 2 absent in basal 1/3, striae 4 and 5 can be interrupted basally, stria 10 reduced and interrupted basally; submarginal stria present only as very weak apical strioles. Elytron with fine punctation and microreticulation.

***Structures***: Head relatively broad. Pronotum trapezoid, its lateral margins convergent anteriorly. Base of prosternum rounded anteriorly, convex medially; blade of prosternal process relatively narrow.

***Male***: Protibia straight, not modified. Proclaws relatively short, subequal in length, anterior claw thicker and more curved subapically than posterior one and due to this with a subapical incision on its inner margin. Median lobe of aedeagus with two lobes of dorsal sclerite subequal in length, with broadly pointed apexes; whole lateral side of left dorsal lobe with large but shallow concavity, covered with numerous large spinula-like structures getting longer and denser towards lateral margin and shorter towards dorsal side where they replaced by scale-like structures; lateral margin slightly concave apically so that apex of left dorsal lobe slightly curved upwards; right dorsal lobe covered with large scale-like structures, with small, indistinct median membranous area; lobes of ventral sclerite partly sclerotised, long, subequal in length, partly visible in left lateral view; left ventral lobe with elongate, strong sclerotised area, concave subapically, with apex pointed and slightly curved to right and membranous right part long, thin, apically covered with setae visible in left lateral view; right ventral lobe with narrow sclerotised area on right margin. Parameres with dense, rather short setae occupying more than half of dorsal margin (Fig. 99).

***Female***: Unknown.

##### Affinities.

The species belong to the medium-sized species with median lobe of the aedeagus that has scale- and spinula-like structures on the left dorsal lobe and among them, to the species with surface of left dorsal lobe completely covered by scale- and spinula-like structures in left lateral and ventral views. Based on body size and dark dorsal colouration, it is similar to *A.kalibumi* sp. nov., *A.herzogensis* sp. nov., *A.mianminensis* sp. nov., and *A.pseudomianminensis* sp. nov. but differs from them in left dorsal lobe of the median lobe with large, shallow concavity and surface of left dorsal lobe completely covered by surface structures. It is also similar to *A.sararti* sp. nov. but differs from it in larger body size, elytron with 10, not 11, dorsal striae, and left dorsal lobe of the median lobe with concavity and large, long spinula-like structures. See also under *A.wasiorensis* sp. nov.

##### Etymology.

The species name is a Latin adjective meaning “dark, dusky” and indicates the dark elytral colouration of the species.

##### Distribution.

Papua New Guinea: Central Province. The species is known only from the type locality (Fig. 108).

##### Habitat.

Unknown.

#### ﻿﻿13. Austrelatus gestroi

Taxon classificationAnimaliaColeoptera Dytiscidae

(Régimbart, 1892)

5D838384-4D92-52A6-81E7-B0CB0A354932

Figs 52 , 101 , 108

Copelatus gestroi Régimbart 1892: 991 (orig. descr.); Régimbart (1899: 303); Zimmermann (1919: 202, 1920: 139); Guignot (1956: 55); Guéorguiev (1968: 14); Guéorguiev and Rocchi (1993: 159); as objective synonym of Copelatusneogestroi Balke in Shaverdo et al. (2008: 50).
Copelatus neogestroi Balke in Shaverdo et al. (2008: 50) as replacement name for C.gestroi Régimbart, 1892; Nilsson and Hájek (2023: 53).
Austrelatus gestroi (Régimbart, 1892): Shaverdo et al. (2023: 6).

##### Type locality.

Papua New Guinea: Central Province, Rigo District, ca. 09°46′51.6″S, 147°50′24″E.

##### Type material.

***Lectotype***: male “N. GUINEA MER. RIGO *Luglio 1889* L.Loria”, “Neu Guinea Mus. Genua”, “Copelatus Gestroi Reg.” [hw], “Coll. Mus. Vindob.” (NHMW). ***Paralectotypes***: 2 males, 6 females “N. GUINEA MER. RIGO *Luglio 1889* L.Loria”, “Neu Guinea Mus. Genua” (NHMW). 1 male “N. GUINEA MER. RIGO *Luglio 1889* L.Loria”, “Neu Guinea Mus. Genua”, “Copelatus Gestroi Reg.” [hw] (NHMW). 1 male “N. Guinea Rigo Luglio 1889 L.Loria” [hw], “Neu Guinea Mus. Genua” (NHMW). 2 males “N. GUINEA MER. RIGO *Luglio 1889* L.Loria”, “Cotype” [red], “Regimbart det., 1891: COPELATUS (s. str.) Gestroi – Reg.” [partly hw] (ZSM). 2 males “N. GUINEA MER. RIGO *Luglio 1889* L.Loria”, “Neu Guinea 7407” [partly hw], “Copelatus Gestroi Rg det. Gschwendt.” [hw], “Staatl. Museum für Tierkunde, Dresden” (MTD). 2 females “N. GUINEA MER. RIGO *Luglio 1889* L.Loria”, “Neu Guinea 7407” [partly hw], “Staatl. Museum für Tierkunde, Dresden” (MTD). 3 males, 2 females “N. GUINEA MER. RIGO *Luglio 1889* L.Loria”, “Neu Guinea 8947” [partly hw], “Staatl. Museum für Tierkunde, Dresden” (MTD). 1 male “N. GUINEA MER. RIGO *Luglio 1889* L.LORIA”, “Regimbart det., 1891: COPELATUS (s. str.) Gestroi – Reg.” [partly hw], “EX TYPUS” (IRSNB). 21 exs. “N. GUINEA MER. RIGO *Luglio 1889* L.Loria” (MNHN); these paralectotypes are from the collections of Régimbart (1 ex.), Legros (1 ex.), Guignot (2 ex.), Oberthür (17 exs.), some of them with the hand written labels indicating *C.gestroi*; median lobes of the specimens were not studied. 1 male “N. GUINEA MER. KELESI Nov. Dic. 1890 L.LORIA”, “MUSEUM PARIS COLL MAURICE REGIMBART 1908”, “MNHN, Paris EC29239 [with QR code]” (MNHN).

##### Additional material.

1 male “Neu Guinea” [hw by ?Zimmermann], “Samml. A. Zimmermann” (ZSM). 1 male [only aedeagus glued on pined triangular, beetle probably lost] “Penis C.gestroi” [hw by ?Zimmermann], “Samml. A. Zimmermann” (ZSM). ***PNG*: *NCD***: 1 male “2846”, “Papua New Guinea: National Capital District, Varirata NP, 600m, 16.xii.2007, 09.26.13S 147.22.09E, Balke & Sagata (PNG 159)” (ZSM). 4 males, 5 females “Stn. No. 196”, “Papua: Pt. Moresby Brown R. Rd., 15.III.1965”, “M.E. Bacchus. B.M. 1965-120” (BMNH, NHMW). 1 male, 4 females “Stn. No. 195”, “Papua: Pt. Moresby Brown R. Rd., 15.III.1965”, “M.E.Bacchus, B.M. 1965-120” (BMNH). 2 males, 3 females “Stn. No. 206”, “Papua: Loloki c. 10 m. N. of Pt. Moresby, 19.III.1965.”, “M.E. Bacchus. B.M. 1965-120” (BMNH). 4 males, 10 females “Stn. No. 208”, “Papua: Loloki, c. 10 m. N. of Pt. Moresby 19.III.1965.”, “M.E. Bacchus. B.M. 1965-120” (BMNH, NHMW). 5 males, 3 females “Stn. No. 207a”, “Papua: Moitaka, c. 7 m. N. of Pt. Moresby. 17.III.1965”, “M.E. Bacchus. B.M. 1965-120” (BMNH, NHMW). 1 male, 1 female “4881 New Guinea Port Moresby Dist 24.iii.1956 E.S. Brown” [partly hw], “Pres. By Com.Inst.Ent. B.M. 1958-79” (BMNH). ***Morobe***: 2 males, 1 female “Stn. No. 103”, “New Guinea: Morobe Dist., Finisterre Mts., Mt. Abilala, c.9,000 ft. 19–22.xi.1964.”, “M.E. Bacchus. B.M. 1965-120” (BMNH).

##### Description.

***Body size and form***: Beetle medium-sized, oblong-oval (Fig. 52).

***Measurements***: TL 5.8–6.8 mm, TL-H 5.45–6.2 mm, MW 2.9–3.3 mm, TL/MW 2–2.06; PL 0.85–0.95 mm, PW 2.45–2.8 mm, PL/PW 0.34–0.35; DBE 0.9–1 mm, DBE/PW 0.35–0.37. ***Lectotype***: TL 6.4 mm, TL-H 5.8 mm, MW 3.15 mm, TL/MW 2.03; PL 0.9 mm, PW 2.6 mm, PL/PW 0.35; DBE 0.9 mm, DBE/PW 0.35.

***Colouration***: With piceous elytra and pronotum and yellowish red posterior margin of head, pronotal sides, and elytral basal band and apex (Fig. 52).

Head almost completely brownish or piceous, often paler, to yellowish red posteriorly and anteriorly and darker medially. Pronotum dark brown to piceous on disc and broadly yellowish red on sides, especially at anterior angles. Elytron dark brown to piceous, with yellowish red basal band differently developed: distinct, relatively broad or rather narrow, often notched at posterior margin (expending between striae), seldom reduced near suture and distinct at shoulders; elytron broadly yellow apically, this yellow colouration usually extending between striae, especially laterally (up to elytral 1/2). Scutellum reddish to piceous. Antennae, other head appendages, and pro- and mesolegs yellowish red, metalegs darker, especially distally. Ventral side reddish brown to brown, darker on coxal plates and abdominal ventrites 4–6.

***Surface sculpture***: Elytron with (10–11)+1 striae (Fig. 52).

Head without strioles, with rather dense punctation (spaces between punctures 1–3× size of punctures); punctures rather coarse (diameter of punctures usually equal to and larger than diameter of microreticulation cells); microreticulation distinct. Pronotum with few to several strioles at posterior angles or more numerous laterally (in females), with punctation finer than on head and microreticulation fine. Elytron with 11 dorsal striae, stria 1 usually reduced to strioles in apical 1/2, sometimes completely absent; striae 2 and 3 usually absent in basal 1/4 and additionally interrupted, sometimes uneven dorsal striae interrupted basally, especially striae 5 and 7; submarginal stria present, well-developed. Elytron with fine punctation and microreticulation.

***Structures***: Head relatively broad. Pronotum trapezoid, its lateral margins convergent anteriorly. Base of prosternum rounded anteriorly, convex medially; blade of prosternal process relatively narrow.

***Male***: Protibia straight, not modified. Proclaws relatively long, subequal in length, anterior claw thicker and more curved at its apex than posterior one and due to this with a subapical incision on its inner margin. Median lobe of aedeagus with two lobes of dorsal sclerite with broadly pointed apexes, left straight, right slightly curved to left; left dorsal lobe slightly longer than right dorsal lobe, distinctly narrowed to apex in its apical 1/2; lateral side of left dorsal lobe in whole apical 1/2 narrowly and shallowly concave under lateral margin that looks like a bead, covered with large scale-like structures towards dorsal side and smaller scale-like structures towards apex and on lateral margin, with numerous small spinula-like structures in concavity and less numerous large, short spinula-like structures medially; right dorsal lobe covered with large scale-like structures, with small, indistinct median membranous area; lobes of ventral sclerite sclerotised, long, broad, partly visible in left lateral view; left ventral lobe with elongate, strong sclerotised area, with slightly curved apex and broad slightly sclerotised part rounded apically; right ventral lobe longer than left one, slightly sclerotised, with long curved apex visible in left lateral view. Parameres with dense, relatively long setae occupying approximately half of dorsal margin, with single the most proximal setae standing separately (Fig. 101).

***Female***: As males but with stronger dorsal punctation and microreticulation and more numerous pronotal strioles.

##### Affinities.

The species belong to the medium-sized species with median lobe of the aedeagus that has scale- and spinula-like structures on the left dorsal lobe and among them, to the species with surface of left dorsal lobe completely covered by scale- and spinula-like structures in left lateral and ventral views. Based on dorsal colouration and general shape of the median lobe, it is similar to *A.asteios* sp. nov., *A.epicharis* sp. nov., *A.wasurensis* sp. nov., and *A.yeretuar* sp. nov. but differs from them in larger body size and left dorsal lobe of the median lobe with narrow, shallow, concavity under the lateral margin and different organisation of the surface structures. It is also very similar to *A.pseudogestroi* sp. nov., see its affinities.

##### Distribution.

Papua New Guinea: Central (Rigo, Kapakapa, Hula and Kelesi in Régimbart (1892: 991; 1899: 303), Angabanga River as Paumomu River in Régimbart (1899: 303)), Morobe, National Capital District, East New Britain (Kokopo as Herbertshöhe in Zimmermann (1919: 202)) provinces (Fig. 108). The East New Britain record needs to be confirmed.

##### Habitat.

The species was collected in Varirata NP in small, shallow and shaded forest pools rich in rotten leaves.

#### ﻿﻿14. Austrelatus herzogensis sp. nov.

Taxon classificationAnimaliaColeoptera Dytiscidae

87779FC0-2939-5031-9602-E5AF03F49B56

https://zoobank.org/A70BB204-8306-43EA-8A9A-1B5E336F3E9A

Figs 43 , 92 , 108

##### Type locality.

Papua New Guinea: Morobe Province, Herzog Mts, backroad to Wagau, 06°46.717'S, 146°38.953'E, 100 m a.s.l.

##### Type material.

***Holotype***: male “Papua New Guinea: Morobe, Herzog Mts., backroad to Wagau, 100m, 3.iv.2006, 6 46.717S 146 38.953E, Balke & Sagata (PNG 29)” (ZSM). ***Paratypes*: *PNG*: *Morobe***: 1 male, 7 females with the same label as the holotype (NHMW, ZSM). 1 male “Papua New Guinea: Morobe, Herzog Mts., backroad to Wagau, 100m, 3.iv.2006, Balke & Sagata (PNG 29)”, “3216” [green label] (ZSM). 5 males, 7 females “Stn. No. 158”, “New Guinea: Morobe Distr., Gusap, Markham, Valley c.90 m W. of Lae. 1,000ft. 27–30.i.1965”, “M.E. Bacchus B.M. 1965-120” (BMNH, NHMW). 1 male, 1 female “Stn. No. 162”, “New Guinea: Morobe Distr., Gusap, Markham, Valley c.90 m W. of Lae. 1,000ft. 27–30.i.1965”, “M.E. Bacchus B.M. 1965-120” (BMNH). 1 female “Stn. No. 161”, “New Guinea: Morobe Distr., Gusap, Markham, Valley c.90 m W.of Lae. 1,000ft. 27–30.i.1965”, “M.E. Bacchus B.M. 1965-120” (BMNH). 1 male, 6 females “Stn. No. 114”, “New Guinea: Morobe Distr., Markham R. Valley. Gusap, c.90 m. W. of Lae. 1000ft. 3.xii.1964”, “M.E. Bacchus B.M. 1965-120” (BMNH). ***Madang***: 3 males, 7 females “Papua New Guinea: Madang, middle ramu [Ramu River], Akaraski, 50m, 12.iii.2007, 05.04.787S 144.43.137E, Kinibel (PNG 157)”, two males with additional green labels “2881” and “2882” (NHMW, ZSM). ***East Sepik***: 1 male, 2 females “Papua New Guinea East Sepik Province Amboin Patrol Post Karawari Lodge 7 Jan. 1983, A.C.Messer” (ZSM). ***IN*: *West Papua*: *Yapen Islands Regency***: 1 male “Irian Jaya: Japen Isl. W Serui, Panduamin ca. 100m, 19.2.1999 leg. Riedel” (NHMW).

##### Description.

***Body size and form***: Beetle medium-sized, oblong-oval (Fig. 43).

***Measurements***: TL 5.65–6.7 mm, TL-H 5.75–6.1 mm, MW 2.8–3.2 mm, TL/MW 2.02–2.09; PL 0.8–0.95 mm, PW 2.3–2.7 mm, PL/PW 0.33–0.37; DBE 0.85–1 mm, DBE/PW 0.37. ***Holotype***: TL 6.3 mm, TL-H 5.75 mm, MW 3.05 mm, TL/MW 2.07; PL 0.95 mm, PW 2.6 mm, PL/PW 0.37; DBE 0.95 mm, DBE/PW 0.37.

***Colouration***: Dorsally piceous, with yellowish red posterior part of head, pronotal sides, especially anterolaterally, broad, basal band and large, apical spot on elytron (Fig. 43).

Head piceous, yellowish red posteriorly, between eyes; sometimes yellowish red to brown, piceous behind eyes and two spots in middle. Pronotum piceous, with yellowish red sides, often only in anterolateral half. Elytron piceous, with broad, distinct, yellowish red basal band and large, yellow spot apically that usually slightly extending laterally. Scutellum piceous. Antennae, other head appendages, and pro- and mesolegs yellowish red, metalegs slightly darker, especially distally. Ventral side brown, with paler prosternum and abdominal ventrites.

***Surface sculpture***: Elytron with (10–11)+0 striae; stria 1 usually strongly reduced, striae 2 and 3 sometimes reduced basally (Fig. 43).

Head without strioles, with rather dense punctation (spaces between punctures 1–3× size of punctures); punctures coarse (diameter of punctures usually larger than or equal to diameter of microreticulation cells); microreticulation distinct. Pronotum without strioles or with few strioles posterolaterally or in females, with numerous strioles laterally; with punctation finer than on head and microreticulation fine. Elytron with 11 dorsal striae, stria 1 often present as strioles in apical 1/2 or sometimes completely absent, striae 2 and 3 usually absent in basal 1/4 or present as strioles, stria 10 reduced at shoulder; submarginal stria absent. Elytron with fine punctation and microreticulation.

***Structures***: Head relatively broad. Pronotum trapezoid, its lateral margins convergent anteriorly. Base of prosternum rounded anteriorly, convex medially; blade of prosternal process relatively narrow.

***Male***: Protibia straight, not modified. Proclaws relatively long, simple, subequal in length. Median lobe of aedeagus with two lobes of dorsal sclerite subequal in length, more or less straight and evenly tapering to broadly pointed apexes, tip of left dorsal lobe apex with kind of small incision, not completely pointed; left dorsal lobe with lateral side modified: with distinct, median concavity of of triangular shape, covered with numerous, very distinct, strong and long spinula-like structures; lateral margin apically without surface structures, smooth; right dorsal lobe covered with scale-like structures, with median membranous area; lobes of ventral sclerite partly sclerotised, long, partly visible in left lateral view; left ventral lobe with elongate, strong sclerotised area, concave apically, with apex pointed and slightly curved to right and membranous right part with long, thin, upwardly curved apex sometimes sticking out in left lateral view; right ventral lobe with narrow sclerotised area on right margin. Parameres with dense, long setae occupying approximately half of dorsal margin, with single the most proximal setae standing separately (Fig. 92).

***Female***: As males but usually with stronger elytral punctation and microreticulation and pronotum with numerous strioles occupying sides completely so that only disc without strioles. Interestingly, some females have pronotum with lateral margin slightly to distinctly concave at anterior angles.

##### Variability.

There is a variation in dorsal colouration and striolation as described above.

##### Affinities.

The species belong to the medium-sized species with median lobe of the aedeagus that has scale- and spinula-like structures on the left dorsal lobe. Based on body size and shape and shape of the median lobe with the characteristic median, triangle concavity of the left dorsal lobe, it is very similar to *A.kalibumi* sp. nov. but differs from it in elytron with broad and distinct, yellowish red basal band and slightly differently shaped median lobe, with its apical part distinctly shorter and apex of the left dorsal lobe less pointed. See also under *A.sararti* sp. nov.

##### Etymology.

The species is named after Herzog Mts. The name is an adjective in the nominative singular.

##### Distribution.

According to the studied material, the species has a disjunct areal: all specimens except one are from Papua New Guinea: Madang, Morobe and East Sepik provinces and only one male (its identification is sure) is known from Yapen Island of Indonesia (Fig. 108). Further sampling activities are necessary to show whether the species is indeed distributed in western part of New Guinea or the Yapen specimen is mislabelled.

##### Habitat.

In the Herzog Mts, the species was collected in shallow and shaded forest pools rich in rotten leaves.

#### ﻿﻿15. Austrelatus inconstans sp. nov.

Taxon classificationAnimaliaColeoptera Dytiscidae

1B84EBBD-8488-5272-BA53-B1262D6D0683

https://zoobank.org/82127944-30E0-4F34-B7E8-5247BA54FCBD

Figs 13–15 , 65 , 107 , 109–110 , 111, 112 , 113

##### Type locality.

Indonesia: Papua Province: Nabire Regency, road Nabire-Enarotali, 54^th^ km, 03°29.51'S, 135°43.91'E, 750–800 m a.s.l.

##### Type material.

***Holotype***: male “West New Guinea/Paniai Prov./IR 19 track Nabire-Ilaga km 54 Basecamp, 750–800m, 16.-27.7.1991 leg: Balke & Hendrich” (ZSM). ***Paratypes*: *IN*: *Papua*: *Nabire Regency***: 52 males, 36 females with the same label as the holotype (MZB, NHMW, ZSM). 9 males, 9 females “IR 19-W, New Guinea, Track Nabire-Ilaga KM 54, basecamp, 750–800m, 16.-27.vii.1991 Balke & Hendrich leg.” (NHMW). 41 males, 12 females “W.-Neuguinea/Paniai Prov. Strasse Nabire-Ilaga km 54 700m, 22.-25.9.1990/IR 11 leg: Balke & Hendrich” (NHMW, ZSM). 6 males, 3 females “West New Guinea/Paniai Prov./IR 20 track Nabire-Ilaga KM 59, ca.750m, 18.7.1991 leg: Balke & Hendrich leg.” (ZSM). 17 males, 3 females “IRIAN JAYA: Paniai Prov. road Nabire – Ilaga, km 65, 29.8.1996, 250m (96 # 6)” (NHMW). 1 male “West New Guinea/Paniai Prov./IR 23 track Nabire-Ilaga km 34 near Topo, 120m, 23.7.1991 leg: Balke & Hendrich” (ZSM). 2 males “IRIAN JAYA: Nabire Prov. rd. Nabire – Ilaga, Km 35 Kali Cemara, 100m, 27.9.1997 (IR97#6)” (NHMW). 1 male, 3 females “IRIAN JAYA: Nabire Prov. rd. Nabire – Ilaga, Km 35 Kali Cemara, 100m, 23.10.1997 (IR97#14)” (NHMW). 1 male “IRIAN JAYA: Paniai Prov. road Nabire – Ilaga, km 38 18.9.1996, 150m (96 # 26)” (NHMW). 2 males “West New Guinea/Paniai Prov./IR 18 River n. Nabire, 2m, 15.7.1991 leg: Balke & Hendrich leg.” (ZSM). 54 males, 36 females “West New Guinea/Paniai Prov./IR 22 track Nabire-Ilaga km 62 250m, 24.7.1991, forest pools leg: Balke & Hendrich” (NHMW, ZSM). 1 female “IRIAN JAYA: Kabup. Nabire rd. Nabire – Ilaga, km 62 200m, IX.1998 leg. Konyorah (62)” (NHMW). 23 males, 2 females “West New Guinea/Paniai Prov./IR 21 track Nabire-Ilaga km 65 Kali Utowa, 250m, 18.&19.7.1991 leg: Balke & Hendrich” (NHMW, ZSM). 9 males, 10 females “IR 23-W. New Guinea, track Nabire-Ilaga KM 62, 250m, 24.vii.1991 Balke & Hendrich leg.” (NHMW). 12 males, 13 females “West New Guinea/Paniai Prov./IR 21 track Nabire-Ilaga km 65 Kali Utowa, 250m, 18.&19.7.1991 leg: Balke & Hendrich” (ZSM). 5 males, 3 females “IR 21-W. New Guinea, track Nabire-Ilaga KM 65, Kali Utowa, 250m, 18.-19.vii.1991 Balke & Hendrich leg.” (ZSM). 1 male, 1 female “Indonesia: Papua, Road Nabire-Enarotali KM 55, 774m, 22.x.2011, 03.29.796S 135.43.885E, UNCEN team (PAP09)”, “5127” [green text] and “5126” [green text], respectively (ZSM). 1 male “5136” [green text], “Indonesia: Papua, Road Nabire-Enarotali KM 62, 340m, 22.x.2011, 03.31.684S 135.42.802E, Uncen (PAP11)” (ZSM). 4 males “W.-Neuguinea /Paniai Prov./ Wanggar- Kali Bumi / IR 14 30.9 & 1.10.90 leg: Balke & Hendrich” (ZSM). 1 male, 1 female “IR 90#14: West New Guinea, Nabire→Wanggar, 100m, 30.ix.1990, Balke” (ZSM). ***Puncak Regency***: 2 males, 1 female “Indonesia: Papua, Rouaffer, Iratoi, hill in forest, 164m, 6.ix.2014, -3,2403 137,332 (Pap028)”, one male and female with additional green text labels “6476” and “6475”, respectively (ZSM). ***West Papua*: *Kaimana Regency***: 1 male “INDONESIA: W-PAPUA vic. Kaimana, road 18 km NE S3°31'11”/E 133°40'15”, 50–80m 21.-25.II.2011 leg. A.Skale (014)” (CAS). ***Teluk Wondama Regency***: 1 male “Indonesia: Irian Jaya, Wandammen, Wasior, DMP, 7.-10.I.2001, Riedel, MB 54”, “54” [green lable] (ZSM). 1 female “Indonesia: West Papua, DMP, Wasior, 7.-10.i.2001, Riedel leg.” (ZSM). 5 males, 2 females “IRIAN JAYA, Wandammen Bay, Wasior, KM 38, Sararti, 100-200 m, 7.-9.I.2001, leg. A. RIEDEL” (SMNS).

##### Description.

***Body size and form***: Beetle large, with oblong-oval habitus (Figs 13–15).

***Measurements***: TL 6.7–8.2 mm, TL-H 6–7.3 mm, MW 3.2–3.9 mm, TL/MW 2.09–2.14; PL 0.95–1.25 mm, PW 2.8–3.5 mm, PL/PW 0.34–0.36; DBE 1.1–1.4 mm, DBE/PW 0.39–0.41. ***Holotype***: TL 7.6 mm, TL-H 6.8 mm, MW 3.55 mm, TL/MW 2.14; PL 1.15 mm, PW 3.2 mm, PL/PW 0.36; DBE 1.3 mm, DBE/PW 0.41.

***Colouration***: With piceous elytra and pronotum and bright yellowish red head, pronotal sides, and basal band on elytron; sometimes yellowish red colouration can be more strongly developed on elytron (Figs 13–15).

Head yellow to yellowish red, narrowly piceous behind eyes. Pronotum dark brown to piceous on disc and gradually paler (to yellow) on sides. Elytron dark brown to piceous, with very distinct yellow to yellowish red basal band, often with yellowish or reddish apex, sometimes also yellow laterally and between striae up to so that whole elytron yellow, with darker disc. Scutellum piceous. Antennae, other head appendages, and pro- and mesolegs yellowish red to yellowish brown, metalegs darker, all legs darker distally. Ventral side reddish brown.

***Surface sculpture***: Dorsal elytral striation very variable, submarginal stria usually absent or present as a short stria or few short strioles apically: (0–10)+(0–1) (Figs 13–15).

Head without strioles, with relatively dense punctation (spaces between punctures 1–3× size of punctures); punctures relatively fine (diameter of punctures smaller than or equal to diameter of microreticulation cells); microreticulation distinct. Pronotum without strioles, with punctation finer than on head and sometimes with longitudinal wrinkles laterally; microreticulation finer than on head. Elytron with striation very variable: from without striae, with three or four puncture lines, which have often some strioles instead of punctures; with up to ten dorsal striae (as in holotype), of which uneven striae usually complete, striae 1 and 2 always reduced basally; submarginal stria usually absent or present as a short stria or few short strioles apically. Elytron with fine punctation and microreticulation. Abdominal ventrite 6 with distinct punctation, sparse medially and forming denser area at each lateral side, with few strioles laterally.

***Structures***: Head relatively broad. Pronotum trapezoid, its lateral margins distinctly convergent anteriorly. Base of prosternum broadly rounded anteriorly, distinctly convex medially; blade of prosternal process broad.

***Male***: Protibia modified: thinner proximally and broader medially and distally due to its curved ventral margin. Proclaws relatively short, subequal in length, anterior claw thicker subapically and more strongly curved downwards than posterior one and due to this with a median incision on its inner margin. Median lobe of aedeagus robust, with two lobes of dorsal sclerite unequal in length and shape, both covered with scale-like structures; left dorsal lobe distinctly broader and longer than right dorsal lobe; left dorsal lobe with a distinct median concavity and its whole apical 1/2 narrowed, its basal part very large, and its apex very slightly curved downwards, with a small crest; right dorsal lobe with apex pointed and membranous area medially; lobes of ventral sclerite sclerotised, pressed together between lobes of dorsal sclerite, but partly visible in left lateral view; left ventral lobe consists of two parts: left sclerotised area (not visible in left lateral view) and less sclerotised right part (visible in left lateral view); sclerotised area with elongate and broad basal 1/2 and long, thread-like apical 1/2, which apex can be visible at median lobe apex; less sclerotised right part long and broad, with its apical part densely covered with long setae; right ventral lobe long and broad. Parameres with dense, long setae occupying slightly more than half of dorsal margin (Fig. 65).

***Female***: Dimorphic: as male, shiny, and matt, with pronotum and elytron very densely covered with fine longitudinal strioles. Matt forms present in almost every population, usually less numerous than shiny ones: e.g., ratio shiny to with strioles is 23:13 in the type locality.

##### Variability.

The species is very variable in the elytral striation and colouration, as described above, within and among populations. The holotype is characteristic: piceous, with yellowish red head, pronotal sides, and basal band on elytron, and with 10+1 elytral striae. All specimens from the type locality (IR19) have such colouration but most of them have some stria reduction up to six dorsal striae. In the neighbour population (locality IR21), almost all specimens have elytra without striae and approximately half of them have yellow elytral colouration strongly developed. From Papua Province (Kaimana, Wandammen, and Iratoi) only striated and dark specimens are recorded.

##### Affinities.

The species belongs to the large species with robust median lobe, covered mainly with scale-like structures. Among them, it is similar to *A.bundunensis* sp. nov. in size, body form, and dorsal colouration, but distinctly differs from it in shape of the median lobe and modified male proclaws.

##### Etymology.

The species name is a Latin adjective meaning inconsistent and indicates very variable elytral striation of the species.

##### Distribution.

Indonesia: West Papua Province and western part of the Papua Province (Fig. 107).

##### Habitat.

Most specimens were collected in shallow (up to 20 cm water depth), shaded or at least partly shaded forest pools and puddles of different size, rich in rotten leaves and twigs, as, for example, a small forest pool at the 62 km of the Nabire-Ilaga track (Figs 109–113). Few specimens were also found in water-filled track hollows on forest tracks.

#### ﻿﻿16. Austrelatus iriatoi sp. nov.

Taxon classificationAnimaliaColeoptera Dytiscidae

33B6DD80-1920-5799-AA0A-BCA7BE6EAE37

https://zoobank.org/50C03E00-528C-4E51-A5AC-630B8FDCC9EF

Figs 25 , 76 , 107

##### Type locality.

Indonesia: Papua Province: Puncak Regency, Iratoi, Rouaffer River, 3°14'25.1"S, 137°19'58.7"E, 164 m a.s.l.

##### Type material.

***Holotype***: male “Indonesia: Papua, Rouaffer, Iratoi, hill in forest, 164m, 6.ix.2014, -3,2403086 137,3329744 (PAP028)” (KSP). ***Paratypes***: 7 males, 9 females with the same label as the holotype, two males with additional labels with green text “6471” and “6472” (MZB, NHMW, ZSM).

##### Description.

***Body size and form***: Beetle small, elongate (Fig. 25).

***Measurements***: TL 4.95–5.55 mm, TL-H 4.5–5.1 mm, MW 2.25–2.5 mm, TL/MW 2.2–2.23; PL 0.75–0.85 mm, PW 2–2.3 mm, PL/PW 0.36–0.38; DBE 0.87–0.95 mm, DBE/PW 0.41–0.44. ***Holotype***: TL 5.45 mm, TL-H 4.8 mm, MW 2.45 mm, TL/MW 2.23; PL 0.8 mm, PW 2.22 mm, PL/PW 0.36; DBE 0.95 mm, DBE/PW 0.43.

***Colouration***: Dorsally piceous, with yellowish red head, pronotal sides and elytral basal band and apex (Fig. 25).

Head yellowish red, narrowly darker behind eyes. Pronotum piceous on disc and gradually paler (to yellowish red) to sides. Elytron piceous, with distinct yellowish red basal band and yellow apically, especially between striae. Scutellum reddish to brown. Antennae, other head appendages, and pro- and mesolegs yellow to yellowish red, metalegs darker, all legs darker distally. Ventral side brown, reddish brown on head, pro- and metaventrite and medially on abdominal ventrites.

***Surface sculpture***: Elytron with 11 complete striae, submarginal stria present: 11+1 (Fig. 25).

Head without strioles, with dense punctation (spaces between punctures 1–3× size of punctures); punctures coarse (diameter of punctures larger than or equal to diameter of microreticulation cells); microreticulation distinct. Pronotum with few strioles at posterior margin to several strioles at anterior and posterior margins, with punctation finer than on head and microreticulation fine. Elytron with 11 complete striae, stria 10 shortly reduced basally; submarginal stria present, long. Elytron with fine, sparse punctation and fine microreticulation.

***Structures***: Head relatively broad. Pronotum trapezoid, its lateral margins convergent anteriorly. Base of prosternum rounded anteriorly, convex medially; blade of prosternal process narrow.

***Male***: Protibia more or less straight, not modified; its ventral margin can be slightly curved proximally. Proclaws very long, straight, subequal in length. Median lobe of aedeagus with two lobes of dorsal sclerite subequal in length, with broadly pointed apexes; left dorsal lobe with lateral margin slightly concave apically and lateral side slightly concave medially, covered laterally with numerous distinct spinula-like structures and dorsally with less numerous scale-like structures, lateral margin without surface structures, smooth; right dorsal lobe covered with scale-like structures; lobes of ventral sclerite partly sclerotised, long, partly visible in left lateral view; left ventral lobe with elongate, narrow sclerotised area, concave apically, with apex pointed and slightly curved; right ventral lobe longer than left lobe, with narrow sclerotised area on right margin, membranous part with long, thin, upwardly curved apex distinctly sticking out in left lateral view. Parameres with dense, long setae occupying distinctly more than half of dorsal margin; on small area proximally shorter and sparser, with several the most proximal setae standing separately (Fig. 76).

***Female***: Only matt forms present.

##### Affinities.

The species is similar to *A.sumokedi* sp. nov. in elongate habitus and dorsal colouration and striation, but differs from it in larger body size, very long, straight male proclaws, and in median lobe structure: slender and more pointed left lobe apex of dorsal sclerite and larger spinula-like structures of left dorsal lobe. In shape and surface structure of the median lobe and long, straight male proclaws, the species is also similar to *A.ohu* sp. nov. but it is larger than the latter, has more elytral striae and slightly differently shaped left dorsal lobe of the median lobe and its spinula-like structures more numerous.

##### Etymology.

The species is named after Iriatoi. The name is a noun in the nominative singular standing in apposition.

##### Distribution.

Indonesia: Papua Province: Puncak Regency. The species is known only from the type locality (Fig. 107).

##### Habitat.

The species was collected in small, shallow and shaded forest pools rich in rotten leaves.

#### ﻿﻿17. Austrelatus kalibumi sp. nov.

Taxon classificationAnimaliaColeoptera Dytiscidae

BEBCB832-3397-55F5-9C19-B2FF040A8F67

https://zoobank.org/36ECD2F5-D604-4866-8FE5-5502AB718BCC

Figs 42 , 91 , 108 , 109 , 115

##### Type locality.

Indonesia: Papua Province: Nabire Regency, Wanggar, Bumi River.

##### Type material.

***Holotype***: male “W.-Neuguinea /Paniai Prov./ Wanggar- Kali Bumi / IR 14 30.9 & 1.10.90 leg: Balke & Hendrich” (ZSM). ***Paratypes***: 63 males, 29 females with the same label as the holotype (NHMW). 16 males, 8 females “West New Guinea/Paniai Prov./IR 23 track Nabire-Ilaga km 34 near Topo, 120m, 23.7.1991 leg: Balke & Hendrich” (MZB, CLH, ZSM). 11 males, 8 females “Irian: Nabire – Ikaga [sic! Ilaga], “KM 35”, Seitenstr. nach K. Cemara, 1991, leg. M. Balke” (ZSM). 1 female “Indonesia: Papua, Road Nabire-Enarotali KM 35, r. Topo, 130m, 22.x.2011, 03 28.727S 135.38.734E, Uncen (PAP09A)” (ZSM). 1 female “Indonesia: Papua, Road Nabire-Enarotali KM 62, 340m, 22.x.2011, 03.31.684S 135.42.802E, Uncen (PAP11)”, “5144” [green text] (ZSM). 6 males, 2 females “IR 90#14: West New Guinea, Nabire→Wanggar, 100m, 30.ix.1990, Balke” (ZSM). 1 male “IRIAN JAYA: Nabire Prov. rd. Nabire – Ilaga, Km 35 Kali Cemara, 100m, 27.9.1997 (IR97#6)” (NHMW). 1 female “IRIAN JAYA: Nabire Prov. rd. Nabire – Ilaga, Km 35 Kali Cemara, 100m, 27.9.1997 (IR97#5)” (NHMW). 1 female “IRIAN JAYA: Nabire Prov. rd. Nabire – Ilaga, Km 35 Kali Cemara, 100m, 23.10.1997 (IR97#14)” (NHMW). 1 male, 2 females “IRIAN JAYA: Paniai Prov. road Nabire – Ilaga, km 38 18.9.1996, 150m (96 # 26)” (NHMW). 1 female “IRIAN JAYA: Paniai Prov. road Nabire – Ilaga, km 90 1.9.1996, 150m (96 # 11)” (NHMW). 4 females “IRIAN JAYA: Kabup, Nabire 30km S Nabire, Kali Cemara, 150m, 15.8.1998 (CE 1)” (NHMW). 2 males, 2 females “IRIAN JAYA: Kabup, Nabire 30km S Nabire, Kali Cemara, 150m, 15.8.1998 (CE 2)” (NHMW).

##### Additional material.

1 female “Indonesia: Papua, Road Nabire-Enarotali KM 62, 340m, 22.x.2011, 03.31.684S 135.42.802E, Uncen (PAP11)” (ZSM).

##### Description.

***Body size and form***: Beetle medium-sized, oblong-oval (Fig. 42).

***Measurements***: TL 6.1–6.7 mm, TL-H 5.5–6.05 mm, MW 2.85–3.25 mm, TL/MW 2.06–2.14; PL 0.85–1 mm, PW 2.4–2.7 mm, PL/PW 0.35–0.37; DBE 0.9–1 mm, DBE/PW 0.37–0.38. ***Holotype***: TL 6.3 mm, TL-H 5.7 mm, MW 3 mm, TL/MW 2.1; PL 0.95 mm, PW 2.6 mm, PL/PW 0.37; DBE 0.95 mm, DBE/PW 0.37.

***Colouration***: Dorsally piceous, with yellowish red pronotal sides, especially anterolaterally, and large yellowish red spot on elytral apex (Fig. 42).

Head piceous, paler (sometimes to yellowish red) posteriorly, between eyes, seldom also anteriorly. Pronotum piceous, with yellowish red sides, often only in anterolateral half. Elytron piceous, with large, yellow spot apically that usually slightly extending laterally. Scutellum piceous. Antennae, other head appendages, and pro- and mesolegs yellowish red, metalegs distinctly darker, especially distally. Ventral side piceous, with slightly paler prosternum.

***Surface sculpture***: Elytron with (10–11)+0 striae; stria 1 usually strongly reduced, stria 2 reduced basally (Fig. 42).

Head without strioles, with rather dense punctation (spaces between punctures 1–3× size of punctures); punctures fine and weakly impressed (diameter of punctures usually smaller than diameter of microreticulation cells); microreticulation distinct. Pronotum without strioles or in females, with numerous strioles laterally, with punctation finer than on head and microreticulation fine. Elytron with 11 dorsal striae, stria 1 often completely absent or present as few tiny strioles in apical 1/2, stria 2 usually absent in basal 1/4 or present as strioles, stria 3 sometimes shortly reduced basally, stria 10 reduced at shoulder or interrupted; submarginal stria absent. Elytron with fine punctation and microreticulation.

***Structures***: Head relatively broad. Pronotum trapezoid, its lateral margins convergent anteriorly. Base of prosternum rounded anteriorly, convex medially; blade of prosternal process relatively narrow.

***Male***: Protibia straight, not modified. Proclaws relatively long, simple, subequal in length. Median lobe of aedeagus with two lobes of dorsal sclerite subequal in length, more or less straight and evenly tapering to broadly pointed apexes; left dorsal lobe with lateral side modified: with distinct, median concavity of triangular shape, covered with numerous, very distinct, strong and long spinula-like structures; lateral margin apically without surface structures, smooth; right dorsal lobe covered with scale-like structures, with median membranous area; lobes of ventral sclerite partly sclerotised, long, partly visible in left lateral view; left ventral lobe with elongate, strong sclerotised area, concave apically, with apex pointed and slightly curved to right and membranous right part with long, thin, upwardly curved apex sometimes sticking out in left lateral view; right ventral lobe longer than left lobe, with narrow sclerotised area on right margin. Parameres with dense, relatively long setae occupying approximately half of dorsal margin (Fig. 91).

***Female***: As males but with stronger elytral punctation and microreticulation and pronotum with strioles, usually numerous, occupying complete sides so that only disc without them.

##### Variability.

There is a variation in dorsal striolation as described above.

##### Affinities.

The species belong to the medium-sized species with median lobe of the aedeagus that has scale- and spinula-like structures on the left dorsal lobe. Based on body size and shape and shape of the median lobe with the characteristic median, triangle concavity of the left dorsal lobe, it is very similar to *A.herzogensis* sp. nov. but differs from it in dark elytron, without yellow basal band and slightly differently shaped median lobe, with its apical part distinctly longer. See also under *A.sararti* sp. nov.

##### Etymology.

The species is named after the Bumi River (= Kali), near Wanggar, Nabire. The name is a noun in the nominative singular standing in apposition.

##### Distribution.

Indonesia: Papua Province: Nabire and Paniai regencies (Fig. 108).

##### Habitat.

Specimens at the type locality were collected in shallow, exposed and flooded meadow and oxbows along Kali (=River) Bumi at Wanggar (Fig. 115). All specimens on the track Nabire to Ilaga at km 34 (Topo), were collected in shallow (up to 20 cm water depth), shaded or at least partly shaded forest pools and road side ditches, rich in rotten leaves and twigs (Fig. 109).

#### ﻿﻿18. Austrelatus kebarensis sp. nov.

Taxon classificationAnimaliaColeoptera Dytiscidae

1C4D515E-5DED-52B9-AAC0-E42FB9210425

https://zoobank.org/B1EAE5A2-5896-4CF5-AA81-6A0A843B6767

Figs 17 , 69 , 107

##### Type locality.

Indonesia: West Papua Province: Manokwari Regency, road from Kebar to Manokwari, 0°48'05.0"S, 133°19'20.6"E, 331 m a.s.l.

##### Type material.

***Holotype***: male “Indonesia: Papua Barat, Kebar to Manokwari, 1h from Kebar, limestone creek and roadside pools,”, “331m, 8.xi.2013, -0.8013 133.3223, Unipa team (BH035)” (KSP). ***Paratypes***: 2 males, 2 females with the same label as the holotype, one male additionally with a green text label “6245” (MZB, NHMW, ZSM). 2 males, 3 females “Indonesia: Papua Barat, Sausapor-Fef, 157m, 30.ix.2014, -0,6975004 132,072253 (BH044)”, one male additionally with a green text label “6485” (ZSM). 1 female “Indonesia: Papua Barat, Kebar Valley, 596m, 6.v.2015, -0,8406 133,2682, Unipa team (BH059)” (ZSM). 2 females “Indonesia: Papua Barat, lowland Manokwari, 66m, 8.v.2015, -0,7433 133,3975, Unipa team (BH065)” (ZSM). 1 male, 1 female “Indonesia: Papua Barat, Manokwari to Kebar, forest stream, 302m, 3.xi.2013, -0.8005 133.3321, Unipa team (BH023)”, with two additional green text labels “6222” and “6221”, respectively (ZSM). 2 males, 1 female “Indonesia: Papua, Manokwari, 140m, 8.ii.2006, 00.55.752S 133.54.448E, Tindige & Balke (BH 09)” (ZSM). 1 male, 3 females “Indonesia. West Papua Manokwari distr., Utai. riv. Arfak Mts., 500m 14.xii.2012 J. Horák leg.” (NMPC).

##### Description.

***Body size and form***: Beetle medium-sized, oblong-oval to elongate (Fig. 17).

***Measurements***: TL 5.1–5.7 mm, TL-H 4.6–5.2 mm, MW 2.4–2.65 mm, TL/MW 2.13–2.15; PL 0.8–0.9 mm, PW 2.1–2.3 mm, PL/PW 0.37–0.39; DBE 0.87–1 mm, DBE/PW 0.41–0.44. ***Holotype***: TL 5.6 mm, TL-H 5.2 mm, MW 2.6 mm, TL/MW 2.15; PL 0.9 mm, PW 2.3 mm, PL/PW 0.39; DBE 1 mm, DBE/PW 0.44.

***Colouration***: Dorsally piceous, with yellowish red head, pronotal sides, basal band and apex of elytron (Fig. 17).

Head yellowish red to red, narrowly piceous behind eyes. Pronotum piceous on disc and gradually paler (to yellowish red) to sides. Elytron piceous, with distinct, broad yellowish red basal band and largely yellow apex, especially between striae. Scutellum reddish brown. Antennae, other head appendages, and pro- and mesolegs yellowish red, metalegs darker, all legs darker distally. Vental side reddish brown.

***Surface sculpture***: Elytron with six dorsal striae and a submarginal stria: 6+1 (Fig. 17).

Head without strioles, with distinct, relatively sparse punctation (spaces between punctures 1–5× size of punctures); punctures relatively fine (diameter of punctures more or less equal to diameter of microreticulation cells); microreticulation distinct. Pronotum without strioles, with punctation finer than on head and microreticulation distinct. Elytron with six dorsal striae; stria 1 can be shortly reduced basally; striae 5 and sometimes 6 shortly reduced basally or present only in apical 1/2; submarginal stria present. Elytron with fine punctation and microreticulation.

***Structures***: Head relatively broad. Pronotum trapezoid, its lateral margins slightly convergent anteriorly. Base of prosternum rounded anteriorly, distinctly convex medially; blade of prosternal process relatively narrow.

***Male***: Protibia straight, not modified. Proclaws relatively long, subequal in length, anterior claw thicker subapically and more strongly curved downwards than posterior one and due to this with a median incision on its inner margin. Median lobe of aedeagus with two lobes of dorsal sclerite subequal in length, covered with large, broad scale-like structures; left dorsal lobe with lateral margin concave apically and with subapical crest, apex broadly pointed and slightly curved upwards; lobes of ventral sclerite partly sclerotised, subequal in length, long, partly visible in left lateral view; left ventral lobe with elongate, narrow sclerotised area, its apex pointed and curved; right ventral lobe more membranous, slightly longer than left lobe, with apex pointed and slightly curved. Parameres with dense, long setae occupying more than half of dorsal margin; on small area proximally shorter and sparser (Fig. 69).

***Female***: As male.

##### Variability.

There is an insignificant variation in the elytral striation and colouration.

##### Affinities.

In body size and shape and dorsal colouration, the species is similar to *A.yamurensis* sp. nov. but distinctly differs from it in the median lobe without submedian knob-like modification of the left dorsal lobe and 6 complete dorsal elytral striae.

##### Etymology.

The species is named after Kebar Village. The name is an adjective in the nominative singular.

##### Distribution.

Indonesia: West Papua Province: Manokwari Regency (Fig. 107).

##### Habitat.

The species was collected in different water bodies: in a limestone creek, roadside pools and a forest stream.

#### ﻿﻿19. Austrelatus kokodensis sp. nov.

Taxon classificationAnimaliaColeoptera Dytiscidae

6D466021-C28D-5424-96EF-7CA69495DFC9

https://zoobank.org/86FB2431-F900-47CC-B647-848205871595

Figs 49 , 97 , 108

##### Type locality.

Papua New Guinea: Central Province, Kokoda, 08°53.481'S, 147°43.648'E, 410 m a.s.l.

##### Type material.

***Holotype***: male “Papua New Guinea: Northern, Kokoda, 410m, i.2008, 53.481S 147.43.648E, Posman, (PNG 174)” (ZSM). ***Paratypes***: 1 male, 2 females with the same label as the holotype (NHMW, ZSM).

##### Description.

***Body size and form***: Beetle medium-sized, rather elongate (Fig. 49).

***Measurements***: TL 5.6–5.65 mm, TL-H 5.1–5.2 mm, MW 2.6–2.65 mm, TL/MW 2.11–2.17; PL 0.8–0.85 mm, PW 2.25–2.3 mm, PL/PW 0.34–0.37; DBE 0.9–0.95 mm, DBE/PW 0.4–0.41. ***Holotype***: TL 5.6 mm, TL-H 5.2 mm, MW 2.65 mm, TL/MW 2.11; PL 0.85 mm, PW 2.3 mm, PL/PW 0.34; DBE 0.95 mm, DBE/PW 0.41.

***Colouration***: With piceous elytra and pronotum and yellowish red head, pronotal sides, and basal band and large apical spot on elytron (Fig. 49).

Head yellowish red, narrowly piceous behind eyes. Pronotum piceous on disc and yellowish red on sides, especially at anterior angles. Elytron piceous, with distinct, rather broad, yellowish red basal band and large, yellow spot on apex often extending between striae and laterally. Scutellum reddish brown to brown. Antennae, other head appendages, and pro- and mesolegs yellowish red, metalegs distinctly darker, especially distally. Ventral side reddish brown to brown, paler on pro- and metaventrite and abdominal ventrites 1–3.

***Surface sculpture***: Elytron with 11+(0–1) striae (Fig. 49).

Head without strioles, with rather dense punctation (spaces between punctures 1–3× size of punctures); punctures rather coarse (diameter of punctures usually equal to and larger than diameter of microreticulation cells); microreticulation distinct. Pronotum with several strioles or more numerous (in females) laterally, with punctation finer than on head and microreticulation fine. Elytron with 11 dorsal striae, striae 1 and 10 usually shortly reduced basally, stria 1 additionally can be interrupted; submarginal stria absent or present as few tiny strioles apically. Elytron with fine punctation and microreticulation.

***Structures***: Head relatively broad. Pronotum trapezoid, its lateral margins convergent anteriorly. Base of prosternum broadly rounded anteriorly, convex medially; blade of prosternal process relatively broad.

***Male***: Protibia straight, not modified. Proclaws relatively long, slightly curved, subequal in length, anterior claw thicker subapically than posterior one and due to this with a shallow median incision on its inner margin. Median lobe of aedeagus with two lobes of dorsal sclerite subequal in length, with broadly pointed, more or less straight apexes; lateral side of left dorsal lobe covered with numerous, small spinula-like structures; lateral margin slightly concave apically, without surface structures, smooth; right dorsal lobe covered with large scale-like structures, with large, distinct median membranous area; lobes of ventral sclerite partly sclerotised, long, subequal in length, partly visible in left lateral view; left ventral lobe with elongate, strong sclerotised area, concave apically, with apex pointed and slightly curved and membranous right part with long, thin, upwardly curved apex sometimes sticking out in left lateral view; right ventral lobe with narrow sclerotised area on right margin. Parameres with relatively dense and long setae occupying approximately half of dorsal margin (Fig. 97).

***Female***: As males but with stronger dorsal punctation and microreticulation and more numerous pronotal strioles.

##### Affinities.

The species belong to the medium-sized species with median lobe of the aedeagus that has scale- and spinula-like structures on the left dorsal lobe and among them, to the species with surface of left dorsal lobe not completely covered by spinula-like structures, upper lateral margin without spinulae, smooth in left lateral and ventral views. Based on body size and dorsal colouration, it is similar to *A.flavocapitatus* sp. nov. but differs from it in more elongate habitus, left dorsal lobe of the median lobe with small spinula-like structures and its apical part slender and more elongate. See also under *A.luteomaculatus* and *A.maindai* sp. nov.

##### Etymology.

The species is named after Kokoda Village. The name is an adjective in the nominative singular.

##### Distribution.

Papua New Guinea: Central Province. The species is known only from the type locality (Fig. 108).

##### Habitat.

Unknown.

#### ﻿﻿20. Austrelatus leptos sp. nov.

Taxon classificationAnimaliaColeoptera Dytiscidae

8A0AB08D-75BF-58FF-BDA9-EFA25008B235

https://zoobank.org/354B356F-655A-4BCF-9183-0B6A08E01F46

Figs 1 , 59 , 107

##### Type locality.

Papua New Guinea: Sandaun, Bewani Stn., ca 03°05.130'S, 141°10.227'E, 300 m a.s.l.

##### Type material.

***Holotype***: male “Papua New Guinea: Sandaun, Bewani Stn., forest puddles @ base of Bewani Mts., 300 m, 12.iv.2006, nr. 03.05.130S 141.10.227E, Balke & Sagata (PNG 38)”, “3210” [green label] (ZSM). ***Paratypes***: 1 female with the same label as the holotype (ZSM).

##### Description.

**Figures 1–4.**
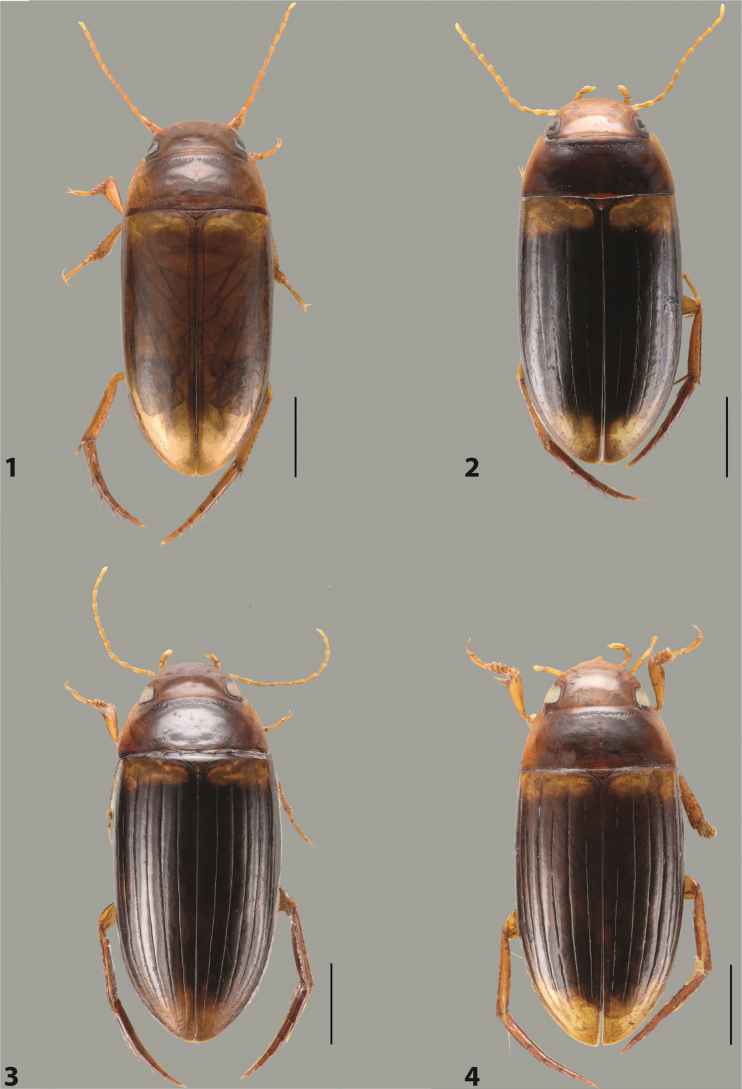
Habitus and colouration of the holotypes of **1***Austrelatusleptos* sp. nov. **2***A.procerus* sp. nov. **3***A.lopintolensis* sp. nov. **4***A.ohu* sp. nov. Scale bars: 1 mm.

***Body size and form***: Beetle small, distinctly elongate, rather slender (Fig. 1).

***Measurements***: TL 4.4–4.5 mm, TL-H 4 mm, MW 1.9 mm, TL/MW 2.32–2.37; PL 0.7–0.75 mm, PW 1.8–1.85 mm, PL/PW 0.38–0.42; DBE 0.9 mm, DBE/PW 0.49–0.5. ***Holotype***: TL 4.5 mm, TL-H 4 mm, MW 1.9 mm, TL/MW 2.37; PL 0.75 mm, PW 1.8 mm, PL/PW 0.42; DBE 0.9 mm, DBE/PW 0.5.

***Colouration***: Dorsally yellowish brown to brown, with distinct yellow basal band on elytron and paler head and pronotal sides; both specimens slightly teneral (Fig. 1).

Head yellowish to yellowish brown, narrowly darker behind eyes. Pronotum brown on disc and paler (yellowish brown to yellow) on sides. Elytron brown, with distinct yellow basal band. Scutellum yellowish to brown. Antennae, other head appendages, and pro- and mesolegs yellowish to yellowish brown, metalegs darker, all legs darker distally. Ventral side yellowish to yellowish brown. Most likely, beetles are darker when more mature.

***Surface sculpture***: Elytron without striae: 0+0, with three or four indistinct puncture lines (Fig. 1).

Head without strioles, with distinct, uneven punctation (spaces between punctures 1–5× size of punctures); punctures fine (diameter of punctures smaller than or equal to diameter of microreticulation cells); microreticulation distinctly impressed. Pronotum with short, weak strioles along posterior margin but mainly at posterior angles, with punctation finer and sparser than on head and microreticulation distinct. Elytron without striae, with three or four indistinct puncture lines (first line near suture sometimes reduced) and separate large punctures between them. Elytron with very fine, sparse punctation and distinct microreticulation.

***Structures***: Head large and broad. Pronotum slightly trapezoid, its lateral margins subparallel. Base of prosternum broadly rounded anteriorly, slightly convex medially; blade of prosternal process broad.

***Male***: Protibia straight, not modified. Proclaws long, slightly curved, subequal in size and form. Median lobe of aedeagus with two lobes of dorsal sclerite subequal in length, covered with scale-like structures; left dorsal lobe with lateral margin slightly concave apically and with some spinula-like structures medially in left lateral view; apex of left dorsal lobe straight, broadly pointed; left lobe of ventral sclerite with elongate, narrow sclerotised area visible in left lateral view, its apex slightly curved upwards; right lobe of ventral sclerite membranous, with long, thin, upwardly curved apex distinctly sticking out in left lateral view. Parameres with relatively dense and long setae occupying more than half of dorsal margin (Fig. 59).

***Female***: As male.

##### Variability.

There is an insignificant variation in the colouration and dorsal punctation described above.

##### Affinities.

The species is very distinct due to its small size, elongate, slender habitus, colouration, elytra without striae, and shape of the median lobe.

##### Etymology.

The species name is a Greek adjective meaning “thin, fine, slender” and indicates small, elongate habitus of the species.

##### Distribution.

Papua New Guinea: Sandaun Province. The species is known only from the type locality (Fig. 107).

##### Habitat.

The species was collected in forest puddles.

#### ﻿﻿21. Austrelatus loloki sp. nov.

Taxon classificationAnimaliaColeoptera Dytiscidae

BA2E5FF2-1B65-5B63-BBEF-26EA39EA7BCD

https://zoobank.org/EA370DAB-AC2D-4EFC-9361-94D07609E02A

Figs 23 , 74 , 107

##### Type locality.

Papua New Guinea: National Capital District: ca. 16 km N from Port Moresby, Loloki River.

##### Type material.

***Holotype***: male “Stn. No. 208”, “Papua: Loloki c. 10 m. N. of Pt. Moresby, 19.III.1965.”, “M.E. Bacchus. B.M. 1965-120” (BMNH).

##### Description.

***Body size and form***: Beetle small, with oblong-oval habitus (Fig. 23).

***Measurements*: *Holotype***: TL 5 mm, TL-H 4.5 mm, MW 2.4 mm, TL/MW 2.08; PL 0.75 mm, PW 2.1 mm, PL/PW 0.36; DBE 0.8 mm, DBE/PW 0.38.

***Colouration***: Dorsally dark brown, elytron with faint yellowish shoulder spot and apicolaterally (Fig. 23).

Head dark brown, narrowly piceous behind eyes and paler on clypeus and posteromedially. Pronotum dark brown on disc and reddish on sides. Elytron dark brown, paler basally, with faint yellowish shoulder spot, yellow at apex and narrowly laterally in more than apical 1/2. Scutellum reddish brown. Antennae, other head appendages, and legs yellowish brown, darker distally. Ventral side brown.

***Surface sculpture***: Elytron with 11 complete, relatively strongly impressed striae, submarginal stria present: 11+1 (Fig. 23).

Head with few strioles between eyes, with distinct, rather dense punctation (spaces between punctures 1–5× size of punctures); punctures relatively coarse (diameter of punctures slightly larger than or equal to diameter of microreticulation cells); microreticulation distinct. Pronotum complete covered with sparse but distinct strioles absent at anterior angles, with punctation finer than on head and microreticulation distinct. Elytron with 11 complete, relatively strongly impressed striae, submarginal stria present, long. Elytron with fine punctation and distinct microreticulation.

***Structures***: Head relatively broad. Pronotum trapezoid, its lateral margins convergent anteriorly. Base of prosternum rounded anteriorly, convex medially; blade of prosternal process relatively broad.

***Male***: Protibia straight, not modified. Proclaws relatively short, slightly curved, subequal in length. Median lobe of aedeagus with two lobes of dorsal sclerite subequal in length, covered with scale-like structures; left dorsal lobe with lateral margin slightly concave medially, with apex truncate and slightly curved downwards; right dorsal lobe narrowed subapically, with broad, rounded apex; lobes of ventral sclerite elongate, mostly sclerotised, almost invisible in lateral view. Parameres with rather short and sparse setae occupying slightly more than half of dorsal margin (Fig. 74).

***Female***: Unknown.

##### Affinities.

The species is well-recognisable by its smaller size, uniform, brown dorsal colouration, strong elytral striation and striolation of head and pronotum, and characteristic shape of the median lobe and paramere setation.

##### Etymology.

The species is named after Loloki River. The name is a noun in the nominative singular standing in apposition.

##### Distribution.

Papua New Guinea: National Capital District. The species is known only from the type locality (Fig. 107).

##### Habitat.

Unknown.

#### ﻿﻿22. Austrelatus lopintolensis sp. nov.

Taxon classificationAnimaliaColeoptera Dytiscidae

F2C1D1DD-C29C-5761-A291-815BB03B7EE9

https://zoobank.org/42A389E2-FBDD-4483-A76F-BD6D141B13FB

Figs 3 , 66 , 107

##### Type locality.

Indonesia: West Papua Province: Raja Ampat Regency, Waigeo Island, Lopintol, Bajon River, 0°07'S, 130°53'E.

##### Type material.

***Holotype***: male “MB759” [green label], “W-PAPUA Raja Ampat Prov. Waigeo Isl., Lopintol, Bajon River, 0°07'S, 130°53'E 11.I.2004 leg. A.,”, “W Papua: Raja Ampat, Waigeo, Lopintol, 11i2004, A. Skale, MB 759” (MZB). ***Paratype***: 1 male “W-PAPUA Raja Ampat Prov. Waigeo Isl., Lopintol 0°07'54"S, 130°53'45"E 11.I.2004 leg. A. Skale UWP” (CAS).

##### Description.

***Body size and form***: Beetle small, elongate (Fig. 3).

***Measurements***: TL 4.7–4.85 mm, TL-H 4.3–4.35 mm, MW 2.1–2.2 mm, TL/MW 2.21–2.24; PL 0.7–0.75 mm, PW 1.9–2 mm, PL/PW 0.37–0.38; DBE 0.9–0.95 mm, DBE/PW 0.47–0.48. ***Holotype***: TL 4.7 mm, TL-H 4.3 mm, MW 2.1 mm, TL/MW 2.24; PL 0.7 mm, PW 1.9 mm, PL/PW 0.37; DBE 0.9 mm, DBE/PW 0.47.

***Colouration***: With dark brown elytra and paler head and pronotal sides; yellow basal band on elytron distinct (Fig. 3).

Head reddish, narrowly darker behind eyes. Pronotum dark brown on disc and gradually paler (to yellowish red) to sides. Elytron dark brown, with distinct yellowish red basal band and pale brown apex. Scutellum brown. Antennae, other head appendages, and pro- and mesolegs yellow, metalegs darker, all legs darker distally. Ventral side yellowish red.

***Surface sculpture***: Elytron with six dorsal striae and a submarginal stria: 6+1 (Fig. 3).

Head without strioles, with distinct, uneven punctation (spaces between punctures 1–5× size of punctures); punctures relatively fine (diameter of punctures more or less equal to diameter of microreticulation cells); microreticulation distinct. Pronotum without strioles, with punctation sparser and microreticulation finer than on head. Elytron with six dorsal striae, striae 1–4 complete, stria 5 reduced basally, stria 6 reduced apically. Elytron with fine punctation and microreticulation.

***Structures***: Head large and broad. Pronotum trapezoid, its lateral margins slightly convergent anteriorly. Base of prosternum rounded anteriorly, convex medially; blade of prosternal process relatively broad.

***Male***: Protibia straight, not modified. Proclaws relatively short, subequal in length, anterior claw thicker subapically and more strongly curved downwards than posterior one and due to this with a median incision on its inner margin. Median lobe of aedeagus with two lobes of dorsal sclerite subequal in length, covered with scale-like structures; left dorsal lobe with lateral margin thickened apically in kind of bead or crest and concave medially, which gives median lobe calla-like shape; this apical crest of left dorsal lobe with small but distinct numerous spinula-like structures; lobes of ventral sclerite partly sclerotised, long, partly visible in left lateral view; left ventral lobe with elongate, narrow sclerotised area, its apex sharply pointed and curved upwards; right ventral lobe more membranous, longer than left lobe, with pointed apex. Parameres with dense, long setae occupying more than half of dorsal margin, proximally shorter and sparser (Fig. 66).

***Female***: Unknown.

##### Affinities.

In body size and shape, elytral striation and dorsal colouration, the species is similar to *A.procerus* sp. nov. and *A.ohu* sp. nov. but distinctly differs from them by its calla-like shape of the median lobe and complete, more strongly impressed elytral striae.

##### Etymology.

The species is named after its type locality, Lopintol. The name is an adjective in the nominative singular.

##### Distribution.

Indonesia: West Papua Province: Raja Ampat Regency, Waigeo Island (Fig. 107).

##### Habitat.

The species was collected in a shaded side pool of a larger river.

#### ﻿﻿23. Austrelatus luteomaculatus

Taxon classificationAnimaliaColeoptera Dytiscidae

(Guignot, 1956)

B0CB90E2-F049-5237-BE42-AD2DB0B7B9F2

Figs 18 , 19 , 70 , 107

Copelatus luteomaculatus Guignot 1956: 53; Guéorguiev 1968: 21; Guéorguiev and Rocchi 1993: 159; Nilsson and Hájek 2023: 50.
Austrelatus luteomaculatus (Guignot, 1956): Shaverdo et al. 2023: 7.

##### Type locality.

Papua New Guinea: Madang Province, Astrolabe Bay, Stephansort, 05°26'38.4"S, 145°44'47.8"E.

##### Type material.

***Holotype***: male “N. Guinea Biró 97.”, “Stephansort Astrolabe B”, “Holotypus 1956 ♂ Copelatusluteomaculatus Guignot” [label with red frame, partly hw by Guignot], “Type” [red label], “Dr F. Guignot det., 1955 Copelatusluteomaculatus sp.n. HoloType ♂” [partly hw by Guignot] (HNHM). ***Paratypes***: 1 female “N. Guinea Biró 97.”, “Stephansort Astrolabe Bai // 18.V. [hw on reverse side]”, “♀”, “Paratype” [red label with black frame] (MNHN); 2 males “N. Guinea Biró 1898”, “Simbang Huon Golf // IX.17. [hw on reverse side]”, “♂”, “Paratype” [red label with black frame] (MNHN); 2 females “N. Guinea Biró 1898”, “Simbang Huon Golf // IX.17. [hw on reverse side]”, “Paratypus 1956 Copelatusluteomaculatus Guignot” [label with red frame, partly hw by Guignot] (HNHM).

##### Additional material.

***PNG*: *Morobe***: 2 males, 1 female “Papua New Guinea: Morobe, Patep, 700m, 20.xi.2006, 06.58.267S 146.37.895E, Balke & Kinibel (PNG 105)” (NHMW, ZSM). 1 female “Papua Neuguinea Morobe Provinz Wau Ecology Institute”, “Wau Station 1217m 15.4.1999 073386S 1467083E leg. M.Schaarschmidt/H.Deumer A.Michalczyk” (ZSM). 2 males, 3 females “Papua New Guinea: Morobe, Herzog Mts., Bundun, 700–800m, 2.iv.2006, 06.51.598S 146.37.07E, Balke & Sagata (PNG 27)” (NHMW, ZSM). 2 males “Papua New Guinea: Morobe, Herzog Mts., backroad to Wagau, 100m, 3.iv.2006, Balke & Sagata (PNG 29)” (ZSM). 1 male “3236” [green label], “Papua New Guinea: Morobe, Huon Pen., rd to Kwapsanek, 250m, 31.iii.2006, 06.30.270S 146.59.581E, Balke & Sagata (PNG 24)” (ZSM). 1 female “Papua New Guinea: Morobe, Sattelberg, Iunjaing, 840m, 16.x.2009, 06.27.239S 147.42.531E, Idaho (09) (PNG209)” (ZSM). 25 males, 16 females “Papua New Guinea: Morobe, Menyamya, coffee garten, 1000m, 14.xi.2006, 07.13.714S 146.01.260E, Balke & Kinibel (PNG 99)”, two males with green labels “3225” and “3226” (NHMW, ZSM). 6 males, 8 females “Stn. No. 114”, “New Guinea: Morobe Distr., Markham R. Valley. Gusap, c.90 m. W. of Lae. 1000ft. 3.xii.1964”, “M.E. Bacchus B.M. 1965-120” (BMNH, NHMW). 3 males, 8 females “Stn. No. 158”, “New Guinea: Morobe Distr., Gusap, Markham Valley. c.90 m. W. of Lae. 1,000 ft. 27-30.i.1965”, “M.E. Bacchus B.M. 1965-120” (BMNH, NHMW). 2 males, 1 female “Stn. No. 159”, “New Guinea: Morobe Distr., Gusap, Markham Valley. c.90 m. W of Lae. 1,000 ft. 27-30.I.1965”, “M.E. Bacchus B.M. 1965-120” (BMNH). 1 male, 1 female “Stn. No. 160”, “New Guinea: Morobe Distr., Gusap, Markham Valley. c.90 m. W. of Lae. 1,000 ft. 27-30.i.1965”, “M.E. Bacchus B.M. 1965-120” (BMNH). 1 male, 4 females “Stn. No. 161”, “New Guinea: Morobe Distr., Gusap, Markham Valley. c.90 m. W. of Lae. 1,000 ft. 27-30.i.1965”, “M.E. Bacchus B.M. 1965-120” (BMNH). 1 female “Stn. No. 131”, “New Guinea: Morobe Distr., Lae-Bulolo Rd., 30.xii.1964.”, “M.E. Bacchus B.M. 1965-120” (BMNH). 8 males, 11 females “Stn. No. 127”, “New Guinea: Morobe Distr., Bulolo, 2,070ft. 28.xii.1964.”, “M.E. Bacchus B.M. 1965-120” (BMNH, NHMW). ***Madand***: 2 males “Papua New Guinea: Madang, Mt Tapo, 180m, ii.2008, 5.24.11.00S 145.36.17.16E, BRC leg. (PNG 178)” (ZSM). 1 male, 2 female “Papua New Guinea: Madang, Brahmin, 150m, 26IX2002, 5 44.953S 145 20.844E Balke & Sagata (PNG 024)” (ZSM). ***Gulf***: 2 males “Papua New Guinea: Gulf, 1500m, 13.xi.2006, 07.11.721S 145.54.746E, Balke & Kinibel (PNG 95)”, one male with a green label “4244” (ZSM).

##### Description.

***Body size and form***: Beetle medium-sized, with oblong-oval habitus (Figs 18, 19).

***Measurements***: TL 5.55–6.6 mm, TL-H 5.1–6 mm, MW 2.7–3.2 mm, TL/MW 2.06–2.07; PL 0.8–0.95 mm, PW 2.3–2.8 mm, PL/PW 0.34–0.35; DBE 0.85–1.01 mm, DBE/PW 0.36–0.39. ***Holotype***: TL 6.2 mm, TL-H 5.6 mm, MW 3 mm, TL/MW 2.07; PL 0.9 mm, PW 2.6 mm, PL/PW 0.35; DBE 1 mm, DBE/PW 0.39.

***Colouration***: Dorsally piceous, with yellowish red head, pronotal sides, basal band and apex of elytron (Figs 18, 19).

Head yellow to red, narrowly piceous behind eyes and darker on clypeus. Pronotum piceous, with yellow to red anterior angles and relatively narrowly on sides, darker to posterolateral angles. Elytron piceous, with bright yellow to red basal band and largely yellow apex, especially between striae. Scutellum reddish to piceous. Antennae, other head appendages, and pro- and mesolegs yellowish red, metalegs darker, all legs darker distally. Ventral side reddish brown, yellowish red on head, pro- and metaventrite. The holotype is a teneral specimen, therefore, distinctly paler.

***Surface sculpture***: Elytron with six dorsal striae and with or without submarginal stria: 6+(0–1) (Figs 18, 19).

Head without strioles, with distinct, relatively sparse punctation (spaces between punctures 2–5× size of punctures); punctures very fine (diameter of punctures smaller than diameter of microreticulation cells); microreticulation distinct. Pronotum with strioles laterally (few at posterolateral angles to rather numerous but sparse on sides), with punctation slightly denser than on head and microreticulation fine. Elytron with six dorsal striae; striae 1, 5, and 6 reduced basally; striae 1 and 5 sometimes more strongly reduced and present only in apical 1/2; sometimes with single strioles between striae; submarginal stria absent or present, sometimes as fine strioles apically. Elytron with fine punctation and microreticulation.

***Structures***: Head relatively broad. Pronotum trapezoid, its lateral margins convergent anteriorly. Base of prosternum narrowly rounded anteriorly, distinctly convex medially; blade of prosternal process relatively narrow.

***Male***: Protibia straight, not modified. Proclaws relatively short, subequal in length, anterior claw thicker subapically and more strongly curved downwards than posterior one and due to this with a median incision on its inner margin. Median lobe of aedeagus with two lobes of dorsal sclerite subequal in length; left dorsal lobe gradually tapering, covered with very dense spinula-like structures except for thickened lateral margin, with apex rounded and more or less straight; right dorsal lobe covered with large scale-like structures, without membranous area medially; lobes of ventral sclerite partly sclerotised, subequal in length, long, partly visible in left lateral view; left ventral lobe with elongate, narrow sclerotised area, well-visible in left lateral view, its apex pointed and curved; right ventral lobe more membranous, slightly longer than left lobe, with apex pointed and slightly curved. Parameres with dense, long setae occupying approximately half of dorsal margin (Fig. 70).

***Female***: As male.

##### Variability.

There is an insignificant variation in the elytral striation and colouration.

##### Affinities.

In colouration and in shape and surfaces structure of the median lobe, the species is similar to *A.flavocapitatus* sp. nov. and *A.kokodensis* sp. nov. but differs from them in larger size, lesser elytral striation and dorsal sclerite shape of the median lobe. *Austrelatusflavocapitatus* sp. nov. has 10 dorsal elytral striae and left lobe of the dorsal sclerite with more concave lateral margin and apex gently curved downwards (with more straight lateral margin and apex gently curved upwards in *A.luteomaculatus*). *Austrelatuskokodensis* sp. nov. has 11 dorsal elytral striae and left dorsal lobe with more concave lateral margin (with more straight lateral margin in *A.luteomaculatus*). Two latter species have right lobe of the dorsal sclerite with membranous area medially, which is lacking in *A.luteomaculatus*.

##### Distribution.

Papua New Guinea: Madang, Morobe, and Gulf provinces (Fig. 107).

##### Habitat.

The species was collected in shallow and shaded forest pools.

#### ﻿﻿24. Austrelatus madangensis sp. nov.

Taxon classificationAnimaliaColeoptera Dytiscidae

557E9222-AABB-5AEA-996F-4AD029A87DD6

https://zoobank.org/BF54D803-7FAF-4A4C-B253-09057B90BF79

Figs 38 , 39 , 88 , 108

##### Type locality.

Papua New Guinea: Madang Province, Wannang, 05°17.235'S, 145°06.160'E, 230 m a.s.l.

##### Type material.

***Holotype***: male “Papua New Guinea: Madang, Wannang, 230m 3.x.2008, 05.17.235S 145.06.160E, Posman (PNG188)” (ZSM). ***Paratypes*: *PNG*: *Madang***: 12 males, 12 females with the same labels as the holotype, one of males additionally with a green label “3765” (NHMW, ZSM). 2 males, 1 female “Papua New Guinea: Madang, Wannang, 270m, 31.x.2008, 05.15.458S 145.02.389E, Posman, (PNG187)”, males with additional green labels “4171” and “3792” (ZSM). 1 male “Papua New Guinea: Wanang I, river, 25.ix.2013, Boukal, 44/2013” (NHMW). 1 female “Ibisca Niugini, PNG 2-4.xii.2012 Wanang -5,227670193 145,0797424”, “FIT-WAN-T-8/8-d16 / Plot 20 / P0707 Vial 22371-CODYTI” (ZSM). 1 male, 1 female “Papua New Guinea: Wanang, Kusa, 24.ix.2013, Boukal, 39/2013” (ZSM). 1 male “Papua New Guinea: Wanang - Kusa, Digitam, 24.ix.2013, Boukal, 40/2013” (ZSM). 4 males, 3 females “PNG Madang Province OHU Village, 14.1.2001 leg.: Lukas Cizek Coll. HENDRICH” (CLH). 1 female “Papua New Guinea: Madang, Madang, Ohu Village, 160m, 30.iv.2006, 05.13.923S 145.40.763E, Balke & Manaono (PNG 49) (ZSM). 1 male, 1 female “Papua New Guinea: Madang, Usino, 260m, 15.iii.2007, 05.31.125S 145.25.316E, Kinibel (PNG 158)” (ZSM). 6 males, 7 females “Papua New Guinea: Madang, Akameku-Brahmin, Bismarck Range, 250–500m, 25.xi.2006, nr 05.47.026S 145.24.131E, Balke & Kinibel (PNG 115)” (NHMW, ZSM). 1 female “Ibisca Niugini, PNG 7–9.xi.2012 Mount Wilhelm 200m -5,739897251 145,3297424 MW0200 / P0866 Vial 07444” (ZSM). 1 male “Ibisca Niugini, PNG 1–3.xi.2012 Mount Wilhelm 200m”, “-5,739897251 145,3297424 FIT-MW200-T-4/8-d08 / P0863 Vial 07369-CODYTI” (ZSM). 1 male “Papua New Guinea Madang Prov. 2002 Hulcr”, “coll. Jiří Hájek National Museum Prague, Czech Republic” (NMPC). 3 males, 3 females “Papua New Guinea: Madang, Adelbert Mts., Keki to Sewan, 650m, 7.v.2006, 04.41.802S 145.25.460E, Balke (PNG 54)”, one male with an additional green label “3202” (NHMW, ZSM). 1 male “Papua New Guinea: Madang, Adalbert [sic!] Mts., Keki, 850m, 4.v.2006, nr 04.42.300S 145.25.089E, Balke & Manaono (PNG 52)”, “3206” [green label] (ZSM). 1 male, 1 female “Papua New Guinea: Madang, Keki – Sewan, Adalbert [sic!] Mts., 700m, 30.xi.2006, nr 04.41.802S 145.25.460E, Binatang Boys (PNG 120)” (NHMW, ZSM). 1 male “N. Guinea Biró 97.”, “Stephansort Astrolabe B.”, “♂”, “papuensis Balf.-Br.” [hw, Guignot], “MUSEUM PARIS 1960 Coll. F.GUIGNOT” [pink label], “MNHN, Paris EC29237 [with QR code]” (MNHN). ***East Sepik***: 2 males “Papua New Guinea: East Sepik, Lembena, 198m, 3.ix.2009, 04.56.974S 143.56.995E, Ibalim & Pius (PNG241)” (ZSM). 1 male, 2 females “Papua New Guinea: East Sepik, Lembena, 198m, 3.ix.2009, 04 46 [!] 974S 143.56.995E, Ibalim & Pius (PNG243)” (ZSM). 1 female “Papua New Guinea: East Sepik, Lembena, 127m, 3.ix.2009, 04 56.953S 143 56.614E, Ibalim & Pius (PNG246)” (ZSM). 9 males, 9 females “Papua New Guinea: East Sepik, Lembena, 117m, 8.ix.2009, 04.57.513S 143 57.296E, Ibalim & Pius (PNG248)”, four males and one female with additional green text labels “5989”, “5991”, “5992”, “5993”, and “5990”, respectively (ZSM). 6 males, 2 females “Papua New Guinea: East Sepik, Lembena, 110m, 10.ix.2009, 04.57.512S 143.57.366E, Ibalim & Pius (PNG249)” (ZSM). 2 males, 1 female “Papua New Guinea: East Sepik, Lembena, 335m, 10.ix.2009, 04.56.859S 143.59.375E, Ibalim & Pius (PNG250)” (ZSM). 2 females “Papua New Guinea: East Sepik, Lembena, 335m, 10.ix.2009, 04 56.859S 143 57.379E, Ibalim & Pius (PNG251)” (ZSM). 1 male “Papua New Guinea: East Sepik, Lembena, 335m, 10.ix.2009, 04 56.921S 143 57.478E, Ibalim & Pius (PNG252)” (ZSM). 2 males, 1 female “Papua New Guinea: East Sepik, Prince Alexander Mts., Wewak-Angoram, 300m, 21.iv.2006, 03.42.129S 143., Balke (PNG 46)”, female with a green label “3222” (ZSM). ***EHL***: 1 male “6438”, “Papua New Guinea: Eastern Highlands, Bena – pass to Goroka valley, 1550m, 5.iv.2006, 06.14.567S 145.29.634E, Balke & Sagata (PNG33)” (ZSM).

##### Description.

***Body size and form***: Beetle large, oblong-oval (Figs 38, 39).

***Measurements***: TL 6.8–8.15 mm, TL-H 6.2–7.35 mm, MW 3.25–4 mm, TL/MW 2.04–2.09; PL 1–1.2 mm, PW 2.9–3.55 mm, PL/PW 0.34–0.35; DBE 1.15–1.35 mm, DBE/PW 0.37–0.4. ***Holotype***: TL 7.6 mm, TL-H 6.8 mm, MW 3.7 mm, TL/MW 2.08; PL 1.15 mm, PW 3.25 mm, PL/PW 0.35; DBE 1.2 mm, DBE/PW 0.37.

***Colouration***: Elytron yellowish red to reddish brown between striae, piceous on pronotal disc, with yellowish red head and pronotal sides (Figs 38, 39).

Head yellowish red, narrowly darker behind eyes. Pronotum brown to piceous on disc and gradually paler (to yellowish red) to sides. Elytral colouration varies: usually yellowish red to reddish brown between striae and piceous on striae, sometimes narrowly brown to piceous on disc, seldom elytron brown to piceous, with yellowish red basal band and yellow apex or apical 1/2. Scutellum yellowish red to brown. Antennae, other head appendages, and pro- and mesolegs yellowish red to reddish, metalegs darker, all legs darker distally. Ventral side brown, paler on prosternum and posteriorly and laterally on metacoxal plates and abdominal ventrites.

***Surface sculpture***: Elytral striation varies: usually elytron with 11 dorsal striae, stria 1 or striae 1–3 reduced in basal 1/2, sometimes stria 1 absent and other reduced or interrupted in basal 1/2, submarginal stria present: (10–11)+1 (Figs 38, 39).

Head without strioles, with rather sparse punctation (spaces between punctures 1–5× size of punctures); punctures rather fine (usually diameter of punctures smaller than or equal to diameter of microreticulation cells); microreticulation distinct. Pronotum without or with strioles posterolaterally, more numerous in females, with punctation finer than on head and microreticulation fine. Elytral striation varies: usually elytron with 11 dorsal striae, stria 1 or striae 1–3 absent in basal 1/2, sometimes more striae (e.g., striae 5–7, 9) reduced basally or differently interrupted; maximal reduction with stria 1 absent and almost all other striae absent or interrupted in basal 1/2; submarginal stria present, sometimes as apical strioles. Elytron with fine, sparse punctation and fine microreticulation.

***Structures***: Head relatively broad. Pronotum trapezoid, its lateral margins convergent anteriorly. Base of prosternum rounded anteriorly, convex medially; blade of prosternal process relatively broad.

***Male***: Protibia more or less straight: its ventral margin slightly curved proximally. Proclaws long, subequal in length, anterior claw thicker apically and more strongly curved downwards than posterior one and due to this with a subapical incision on its inner margin. Median lobe of aedeagus robust, with two lobes of dorsal sclerite covered with scale-like structures; left dorsal lobe slightly concave medially, its whole apical 1/2 broad, more or less evenly tapering to rather large, curved downwards, truncate apex bearing broad crest; right dorsal lobe shorter than left one, broad, with pointed apex and membranous area medially; lobes of ventral sclerite partly sclerotised, pressed together between lobes of dorsal sclerite, slightly visible in left lateral view; left ventral lobe consists of two parts: left sclerotised area and less sclerotised right part; sclerotised area with elongate and broad basal 1/2 and long, thread-like apical 1/2; less sclerotised right part long and broad, with its apical part densely covered with long setae (sometimes visible at apex); right ventral lobe partly sclerotised, long and broad. Parameres with dense, long setae occupying approximately half of dorsal margin (Fig. 88).

***Female***: Dimorphic: as male, but with much more numerous pronotal strioles and distinctly denser punctation and stronger microreticulation on pronotum and elytron, and matt, with pronotum and elytron very densely covered with fine longitudinal strioles. Interestingly, the matt forms are characteristic only for the populations with male specimens having reduced elytral striae, e.g., ones of Adelbert and Alexander Mts or Ohu Village. In latter population, the normal form is also present but less numerous: ratio shiny to with strioles is 1:3.

##### Variability.

There is a strong variation in dorsal colouration and striolation as described above. As mentioned above, specimens with reduced elytral striae are observed in three regions: Ohu Village and, especially Adelbert and Alexander Mts. Beetles from the two latter regions also have larger, broader, and more robust habitus and sturdier median lobe of aedeagus.

##### Affinities.

The species belongs to the large species with robust median lobe, covered mainly with scale-like structures. Among them, in shape of the median lobe, it is similar to *A.mamberamo* sp. nov. and differs from it in more pale elytral colouration and apex of left dorsal lobe of the median lobe shorter, with more vertical, broader crest. See also under *A.mamberamo* sp. nov.

##### Etymology.

The species is named after Madang Province where it is largely distributed. The name is an adjective in the nominative singular.

##### Distribution.

Papua New Guinea: Madang, Eastern Highlands and East Sepik provinces (Fig. 108).

##### Habitat.

Unknown.

#### ﻿﻿25. Austrelatus maindai sp. nov.

Taxon classificationAnimaliaColeoptera Dytiscidae

F02519B0-51B3-5E7E-BD8B-B66F89EBF785

https://zoobank.org/E25F8356-6F8B-454C-B882-48957BC20320

Figs 47 , 96 , 108 , 114

##### Type locality.

Indonesia: Papua Province: Sarmi Regency, Waskey, 01°58'17.0"S, 138°50'56.9"E, 70 m a.s.l.

##### Type material.

***Holotype***: male “Indonesia: Papua, Sarmi area, 70m, 25.ix.2014,-1.9713908 138.8491402, Tim UNCEN: Balke & Menufandu (Pap032)” (KSP). ***Paratypes***: 2 males, 1 female with the same label as the holotype, one male with an additional green text label “6469” (MZB, ZSM). 1 male “Indonesia: Papua, Sarmi, Waaf, N Foja Mts, waterfall in forest, 120m, 23.ix.2014, -2.3317793, 138.7500472, Tim UNCEN: Balke & Menufandu (Pap031)”, “6467” [green text] (ZSM). 1 male “Indonesia: Papua, Foja Mountaints N foot, N Waaf vill., pondok, 150m, 4.-7.vi.2016,”, “-2.06142 138.743949, Sumoked (Pap061)” (NHMW). 1 male, 1 female “Indonesia: West Papua, Sarmi Dest, Tor riv. Togonfo vill. 2°15'07.03"S 138°51'25.89"E, 80 m, hollowed log with water, 25.v.2019, leg. Bretschneider Exp.” (ZSM).

##### Description.

***Body size and form***: Beetle medium-sized, oblong-oval (Fig. 47).

***Measurements***: TL 5.4–5.7 mm, TL-H 4.95–5.25 mm, MW 2.6–2.7 mm, TL/MW 2.04–2.11; PL 0.8–0.85 mm, PW 2.3–2.4 mm, PL/PW 0.35; DBE 0.9–0.95 mm, DBE/PW 0.38–0.4. ***Holotype***: TL 5.5 mm, TL-H 5.1 mm, MW 2.7 mm, TL/MW 2.04; PL 0.85 mm, PW 2.4 mm, PL/PW 0.35; DBE 0.9 mm, DBE/PW 0.38.

***Colouration***: With piceous elytra and pronotum and yellowish red head, pronotal sides, and basal band and large apical spot on elytron (Fig. 47).

Head yellowish red, narrowly piceous behind eyes. Pronotum piceous on disc and yellowish red on sides, especially at anterior angles. Elytron piceous, with distinct, rather broad, yellowish red basal band and large, often extending between striae and laterally, yellow spot on apex. Scutellum reddish brown to piceous. Antennae, other head appendages, and pro- and mesolegs yellowish red, metalegs distinctly darker, especially distally. Ventral side reddish brown to brown, paler on pro- and metaventrite and abdominal ventrites 1–3.

***Surface sculpture***: Elytron with 11+1 striae (Fig. 47).

Head without strioles, with rather dense punctation (spaces between punctures 1–3× size of punctures); punctures rather coarse (diameter of punctures usually equal to and larger than diameter of microreticulation cells); microreticulation distinct. Pronotum with several strioles posterolaterally or in females, more numerous laterally, with punctation finer than on head and microreticulation fine. Elytron with 11 complete dorsal striae, striae 1 and 10 usually shortly reduced basally; submarginal stria present. Elytron with fine punctation and microreticulation.

***Structures***: Head relatively broad. Pronotum trapezoid, its lateral margins convergent anteriorly. Base of prosternum broadly rounded anteriorly, convex medially; blade of prosternal process relatively broad.

***Male***: Protibia straight, not modified. Proclaws relatively long, slightly curved, subequal in length, anterior claw thicker subapically than posterior one and due to this with a median incision on its inner margin. Median lobe of aedeagus with two lobes of dorsal sclerite subequal in length, with broadly pointed, more or less straight apexes; lateral side of left dorsal lobe with large but shallow, subapical concavity, covered with numerous, small spinula-like structures; lateral margin slightly concave apically, without surface structures, smooth; right dorsal lobe covered with large scale-like structures, with very small, indistinct median membranous area; lobes of ventral sclerite partly sclerotised, long, subequal in length, partly visible in left lateral view; left ventral lobe with elongate, strong sclerotised area, concave subapically, with apex pointed and slightly curved to right and membranous right part with long, thin, upwardly curved apex distinctly projecting in left lateral view; right ventral lobe with narrow sclerotised area on right margin. Parameres with dense, long setae occupying distinctly more than half of dorsal margin; setae on small area proximally shorter and sparser, several most proximal setae standing separately (Fig. 96).

***Female***: As males but with stronger dorsal punctation and microreticulation and more numerous pronotal strioles.

##### Affinities.

The species belong to the medium-sized species with median lobe of the aedeagus that has scale- and spinula-like structures on the left dorsal lobe and among them, to the species with surface of left dorsal lobe not completely covered by spinula-like structures, upper lateral margin without spinulae, smooth in left lateral and ventral views. Based on body size and shape and dorsal colouration, it is similar to *A.flavocapitatus* sp. nov. and *A.kokodensis* sp. nov. but differs from them in left dorsal lobe of the median lobe with large lateral concavity.

##### Etymology.

The species is named after our colleague Tobias Mainda, a great naturalist and next-generation rove beetle expert. The specific epithet is a substantive in the genitive case.

##### Distribution.

Indonesia: Papua Province: Sarmi Regency (Fig. 108).

##### Habitat.

In Foja Mts, the species was collected from small puddles near a waterfall in forest. The specimens from Togonfo were sampled from a water-filled hollow in a tree trunk (Fig. 114).

#### ﻿﻿26. Austrelatus mamberamo sp. nov.

Taxon classificationAnimaliaColeoptera Dytiscidae

DE44B26F-8FE9-55F6-8BF8-69BEE66C2165

https://zoobank.org/0A2FE69A-DF08-46F5-BCC6-4F8BC9CEC018

Figs 40 , 89 , 108

##### Type locality.

Indonesia: Papua Province: Mamberamo Raya Regency, Rouffaer Mts, Noiadi, ca. 02°46'S, 137°46'E, 150–200 m a.s.l.

##### Type material.

***Holotype***: male “IRAN JAYA: Jayapura Prov. Mamberamo, Rouffaer Mts., Noiadi, 150–200m 17.3.1999, leg. Riedel” (NHMW). ***Paratypes***: 1 male with the same label as the holotype (NHMW).

##### Description.

***Body size and form***: Beetle large, oblong-oval (Fig. 40).

***Measurements***: TL 7.5–7.9 mm, TL-H 6.8–7.3 mm, MW 3.6–3.9 mm, TL/MW 2.03–2.08; PL 1.1–1.15 mm, PW 3.3–3.45 mm, PL/PW 0.33; DBE 1.3–1.35 mm, DBE/PW 0.39. ***Holotype***: TL 7.5 mm, TL-H 6.8 mm, MW 3.6 mm, TL/MW 2.08; PL 1.1 mm, PW 3.3 mm, PL/PW 0.33; DBE 1.3 mm, DBE/PW 0.39.

***Colouration***: Dorsally piceous, with reddish brown head and reddish pronotal sides, elytral basal band and apex (Fig. 40).

Head reddish brown, narrowly darker behind eyes. Pronotum piceous on disc and gradually paler (to reddish) to sides. Elytron piceous, with narrow yellowish red to reddish basal band and yellowish red spot on apex. Scutellum reddish brown to piceous. Antennae, other head appendages, and pro- and mesolegs reddish, metalegs darker, all legs darker distally. Ventral side piceous, paler on prosternum and posteriorly on metacoxal plates and abdominal ventrites.

***Surface sculpture***: Elytron with 11 dorsal striae, stria 1 reduced to strioles in apical 1/2, some other striae reduced or interrupted basally, submarginal stria present: 11+1 (Fig. 40).

Head without strioles, with dense punctation (spaces between punctures 1–3× size of punctures); punctures relatively fine (usually diameter of punctures equal to or smaller than diameter of microreticulation cells); microreticulation distinct. Pronotum without or with few strioles posterolaterally, with punctation finer than on head and microreticulation fine. Elytron with 11 dorsal striae: stria 1 absent on basal 1/2 and reduced to strioles in apical 1/2, striae 2 and 3 strongly reduced basally, sometimes striae 5–7 shortly reduced basally; submarginal stria present, long. Elytron with fine, sparse punctation and fine microreticulation.

***Structures***: Head relatively broad. Pronotum trapezoid, its lateral margins convergent anteriorly. Base of prosternum rounded anteriorly, convex medially; blade of prosternal process relatively broad.

***Male***: Protibia modified: thinner proximally and broader medially and distally due to its curved ventral margin. Proclaws long, subequal in length, anterior claw thicker apically and more strongly curved downwards than posterior one and due to this with a subapical incision on its inner margin. Median lobe of aedeagus robust, with two lobes of dorsal sclerite covered with scale-like structures; left dorsal lobe slightly concave medially, its whole apical 1/2 broad, more or less evenly tapering to rather large, elongate, curved downwards, truncate apex bearing broad crest; right dorsal lobe shorter than left one, broad, with pointed apex and membranous area medially; lobes of ventral sclerite partly sclerotised, pressed together between lobes of dorsal sclerite, slightly visible in left lateral view; left ventral lobe consists of two parts: left sclerotised area and less sclerotised right part; sclerotised area with elongate and broad basal 1/2 and long, thread-like apical 1/2; less sclerotised right part long and broad, with its apical part densely covered with long setae (sometimes visible at apex); right ventral lobe partly sclerotised, long and broad. Parameres with dense, long setae occupying approximately half of dorsal margin (Fig. 89).

***Female***: Unknown.

##### Affinities.

The species belongs to the large species with robust median lobe, covered mainly with scale-like structures. Among them, in shape of the median lobe, it is similar to *A.madangensis* sp. nov. and differs from it in elytron darker, with narrow, yellowish red basal band and apex of left dorsal lobe of the median lobe more elongate, with more transverse, narrower crest. See also under *A.madangensis* sp. nov. and *A.noiadi* sp. nov.

##### Etymology.

The species is named after the Mamberamo Raya Regency. The name is a noun in the nominative singular standing in apposition.

##### Distribution.

Indonesia: Papua Province: Mamberamo Raya Regency. The species is known only from the type locality (Fig. 108).

##### Habitat.

Unknown.

#### ﻿﻿27. Austrelatus mianminensis sp. nov.

Taxon classificationAnimaliaColeoptera Dytiscidae

F5905D1D-C997-56F2-A9E7-B232ADAC2620

https://zoobank.org/0C7A4E92-B360-4057-B2F4-64E348F98101

Figs 44 , 93 , 108

##### Type locality.

Papua New Guinea: Sandaun Province, Mianmin, 04°53'17.5"S, 141°34'07.1"E, 670 m a.s.l.

##### Type material.

***Holotype***: male “Papua New Guinea: Sandaun, Mianmin, 670m 20.x.2008, 4.53.292S 141.34.118E, Ibalim (PNG 191)” (ZSM). ***Paratypes*: *PNG*: *Sandaun***: 22 male, 36 female with the same label as the holotype (NHMW, ZSM). 1 male, 1 female “Papua New Guinea: Sandaun, Mianmin (pool), 990m, 23.x.2008, 4.54.570S 141.35.490E, Ibalim (PNG 193)” (ZSM). 1 male, 1 female “Papua New Guinea: Sandaun, Mianmin (pool), 1080m, 24.x.2008, 04.55.780S 141.38.185E, Ibalim (PNG 196)”, male with an additional green label “3771” (ZSM). 2 males, 2 females “Papua New Guinea: Sandaun, Mianmin (river) 700m, 21.x.2008, 04.52.858S 141.31.706E Ibalim (PNG 197)”, one male with an additional green label “3786” (ZSM). 8 males, 14 females “Papua New Guinea: Sandaun, Mianmin (pool), 700m, 21.x.2008, 04.52.858S 141.31.706E, Ibalim (PNG 198)” (NHMW, ZSM). 7 males, 7 females “Papua New Guinea: Sandaun, Mianmin, 670m, 22.x.2008, 04.53.329S 141.35.263E S. Ibalim PNG189” (NHMW, ZSM). 5 males, 1 female “Papua New Guinea: Sandaun, Mianmin area, >700m, 7.i.2010, Ibalim & Pius (PNG231)”, two males with additional green text labels “6017” and “6018” (ZSM). 1 male “Papua New Guinea: Sandaun, Mianmin, Fak River, 775m, 13.xi.2003, 4.53.53.00S 141.36.39.40E, K. Sagata (WB31)”, “4197” [green label] (ZSM).

##### Additional material.

***PNG*: *Madang***: 1 male, 4 females “Papua New Guinea: Madang, Trans Gogol, 30 m, ii.2008, 5 18.0915S 145 36.4532E, BRC leg. (PNG 179)” [teneral specimens] (ZSM).

##### Description.

***Body size and form***: Beetle medium-sized, oblong-oval (Fig. 44).

***Measurements***: TL 5.6–6.4 mm, TL-H 5.2–5.8 mm, MW 2.8–3.1 mm, TL/MW 2–2.07; PL 0.8–0.9 mm, PW 2.3–2.6 mm, PL/PW 0.34–0.35; DBE 0.9–0.95 mm, DBE/PW 0.36–0.39. ***Holotype***: TL 6 mm, TL-H 5.5 mm, MW 2.9 mm, TL/MW 2.07; PL 0.85 mm, PW 2.5 mm, PL/PW 0.34; DBE 0.9 mm, DBE/PW 0.36.

***Colouration***: Dorsally piceous, with yellowish red to reddish brown head, anterior angles of pronotum, and yellow to yellowish red spot on elytral apex (Fig. 44).

Head yellowish red to reddish brown, narrowly piceous behind eyes. Pronotum piceous, yellowish red to reddish brown in anterior of sides, sometime more, sometimes less extending. Elytron piceous, with yellow to yellowish red apical spot, sometimes rather small and rounded, sometimes larger or even slightly extending laterally. Scutellum piceous. Antennae, other head appendages, and pro- and mesolegs yellowish red to reddish brown, metalegs darker, especially distally. Ventral side piceous, with brown prosternum.

***Surface sculpture***: Elytron with 11+1 striae; stria 1 often differently reduced (Fig. 44).

Head without strioles, with rather dense punctation (spaces between punctures 1–3× size of punctures); punctures rather coarse (diameter of punctures usually equal to or larger than diameter of microreticulation cells); microreticulation distinct. Pronotum with distinct strioles laterally, with punctation finer than on head and microreticulation fine. Elytron with 11 dorsal striae, stria 1 usually weakly impressed, often absent in basal 1/2 or present as strioles, maximal reduction to few strioles in apical 1/2, striae 2 and 10 can be shortly reduced basally; submarginal stria present. Elytron with fine but distinct punctation and fine microreticulation.

***Structures***: Head relatively broad. Pronotum trapezoid, its lateral margins convergent anteriorly. Base of prosternum rounded anteriorly, convex medially; blade of prosternal process relatively narrow.

***Male***: Protibia straight, not modified. Proclaws relatively long, slightly curved, subequal in length, anterior claw thicker at its apex than posterior one and due to this with a subapical incision on its inner margin. Median lobe of aedeagus with two lobes of dorsal sclerite subequal in length, more or less straight, with very broadly pointed apexes, apex of left dorsal lobe slightly curved downwards; left dorsal lobe with lateral side covered with numerous, distinct, strong spinula-like structures; lateral margin apically without surface structures, smooth; right dorsal lobe covered with scale-like structures, with distinct median membranous area; lobes of ventral sclerite partly sclerotised, long, subequal in length, partly visible in left lateral view; left ventral lobe with elongate, strong sclerotised area, concave subapically, with apex pointed and slightly curved to right and membranous right part long, thin, apically covered with setae sometimes visible in left lateral view; right ventral lobe with narrow sclerotised area on right margin. Parameres with dense, long setae occupying slightly more than 1/2 of dorsal margin (Fig. 93).

***Female***: As males but with stronger dorsal punctation and microreticulation and pronotum with strioles, usually numerous, occupying sides completely so that only disc without strioles.

##### Variability.

There is a variation in dorsal colouration and striolation as described above.

##### Affinities.

The species belong to the medium-sized species with median lobe of the aedeagus that has scale- and spinula-like structures on the left dorsal lobe and among them, to the species with surface of left dorsal lobe not completely covered by spinula-like structures, upper lateral margin without spinulae, smooth in left lateral and ventral views. Based on body size and shape and dorsal colouration and striolation, it is similar to *A.kalibumi* sp. nov. but differs from it in paler head colour and median lobe without concavity of its left dorsal lobe. See also under *A.pseudomianminensis* sp. nov., *A.flavocapitatus* sp. nov. and *A.sararti* sp. nov.

##### Etymology.

The species is named after Mianmin area. The name is an adjective in the nominative singular.

##### Distribution.

Papua New Guinea. The species is mainly known from Mianmin area of the Sandaun Province; five teneral specimens, most likely belonged to the species, are recorded from Madang Province (Fig. 108).

##### Habitat.

In the Mianmin area, the species was collected from a forest pool and a river side pool.

#### ﻿﻿28. Austrelatus miltokarenos sp. nov.

Taxon classificationAnimaliaColeoptera Dytiscidae

633BEE9A-7C5E-5AFC-B8BD-A5E266563136

https://zoobank.org/7AD55950-D944-45DC-B20D-AA5DD62DEC53

Figs 22 , 73 , 107

##### Type locality.

Papua New Guinea: Morobe Province, Garaina, 07°52.516'S, 147°10.427'E, 770 m a.s.l.

##### Type material.

***Holotype***: male “Papua New Guinea: Garaina, 770m, vi.2008 07.52.516S 147.10.427E Ibalim & Sosanika (PNG219)” (ZSM). ***Paratypes***: 2 males, 4 females with the same label as the holotype (NHMW, ZSM). 1 female “Papua New Guinea: Garaina, 770m, 25.vi.2008, 07.50.859S 147.08.614E, Ibalim & Sosanika, (PNG222)” (ZSM)

##### Description.

***Body size and form***: Beetle large, elongate (Fig. 22).

***Measurements***: TL 7.3–8.2 mm, TL-H 6.5–7.4 mm, MW 3.3–3.8 mm, TL/MW 2.16–2.21; PL 1.1–1.25 mm, PW 2.95–3.45 mm, PL/PW 0.36–0.37; DBE 1.25–1.4 mm, DBE/PW 0.41–0.42. ***Holotype***: TL 8.2 mm, TL-H 7.4 mm, MW 3.8 mm, TL/MW 2.16; PL 1.25 mm, PW 3.45 mm, PL/PW 0.36; DBE 1.4 mm, DBE/PW 0.41.

***Colouration***: Dorsally piceous, with yellowish red head and pronotal sides (Fig. 22).

Head yellowish red, narrowly piceous behind eyes. Pronotum reddish brown to piceous on disc and gradually paler on sides, to yellowish red at anterior angles. Elytron piceous, seldom with faint, small reddish spot(s) at shoulder and/or reddish along suture. Scutellum reddish to piceous. Antennae, other head appendages, and legs yellowish red to red, darker distally. Ventral side reddish brown to brown.

***Surface sculpture***: Dorsal elytral striation variable, usually with ten complete and partly reduced striae, submarginal stria present: (6–11)+1 (Fig. 22).

Head without strioles, with relatively sparse punctation (spaces between punctures 2–5× size of punctures); punctures relatively fine (diameter of punctures smaller than or equal to diameter of microreticulation cells); microreticulation distinct. Pronotum with strioles laterally, with punctation finer than on head and microreticulation fine. Elytron with striation variable, usually with ten dorsal, complete or partly reduced, striae, sometimes with six striae with some strioles between them and stria 1 reduced basally (as in holotype), seldom with 11 stria, stria 1 short, present only medially; submarginal stria long, distinct. Elytron with very fine, sparse punctation and fine microreticulation.

***Structures***: Head relatively broad. Pronotum trapezoid, its lateral margins distinctly convergent anteriorly. Base of prosternum rounded anteriorly, distinctly convex medially; blade of prosternal process relatively broad.

***Male***: Protibia straight, not modified. Proclaws very long, slightly curved, subequal in size, anterior claw with slight median incision of its inner margin subapically, thinner apically. Median lobe of aedeagus robust, with two lobes of dorsal sclerite subequal in length and shape, both covered with scale-like structures; left dorsal lobe slightly shorter and apically broader than right dorsal lobe, with a distinct median concavity and its whole apical 1/2 narrowed, its proximal part very large, and its apex slightly curved downwards, with a distinct crest; right dorsal lobe with apex pointed and membranous area medially; lobes of ventral sclerite sclerotised, pressed together between lobes of dorsal sclerite, but partly visible in left lateral view; left ventral lobe consists of two parts: left sclerotised area (visible as a triangular in left lateral view) and less sclerotised right part (visible in left lateral view); sclerotised area with elongate and broad basal 1/2 and long, thread-like apical 1/2; less sclerotised right part long and broad, with its apical part densely covered with long setae; right ventral lobe long and broad. Parameres with dense, long setae occupying approximately half of dorsal margin (Fig. 73).

***Female***: As male, except for more numerous pronotal strioles, sometimes absent only on disc.

##### Variability.

There is a variation in the elytral striation and colouration described above.

##### Affinities.

The species belongs to the large species with robust median lobe, covered mainly with scale-like structures. Among them, it is similar to *A.bundunensis* sp. nov. in simple male proclaws and shape of the median lobe but differs from it in larger and elongate body, absence of basal elytral band and slightly different structure of median lobe sclerites: see under *A.bundunensis* sp. nov. Also see under *A.xanthocephalus*.

##### Etymology.

The species name is a combination of the Greek words *miltos* (red lead, red) and *karenon* (head), meaning red-headed and refers to the distinct yellowish red head colouration of the species. The species name is an adjective in the nominative singular.

##### Distribution.

Papua New Guinea: Morobe Province (Fig. 107).

##### Habitat.

Unknown.

#### ﻿﻿29. Austrelatus noiadi sp. nov.

Taxon classificationAnimaliaColeoptera Dytiscidae

34B0D786-A126-5B49-8CF5-609B4B83C377

https://zoobank.org/7D938245-BF30-4A60-A053-823D76F0CFDB

Figs 37 , 87 , 108

##### Type locality.

Indonesia: Papua Province: Mamberamo Raya Regency, Rouffaer Mts, Noiadi, ca. 02°46'S, 137°46'E, 150–200 m a.s.l.

##### Type material.

***Holotype***: male “IRAN JAYA: Jayapura Prov. Mamberamo, Rouffaer Mts., Noiadi, 150–200m 17.3.1999, leg. Riedel” (NHMW).

##### Description.

***Body size and form***: Beetle large, oblong-oval (Fig. 37).

***Measurements*: *Holotype***: TL 7.9 mm, TL-H 7.1 mm, MW 3.8 mm, TL/MW 2.08; PL 1.1 mm, PW 3.3 mm, PL/PW 0.33; DBE 1.3 mm, DBE/PW 0.39.

***Colouration***: Elytron piceous in basal 1/2 and yellowish red between striae in apical 1/2 and laterally, with narrow, faint, reddish basal band; head reddish brown, pronotal sides reddish (Fig. 37).

Head reddish brown, narrowly darker behind eyes. Pronotum piceous on disc and gradually paler (to reddish) to sides. Elytron piceous basally on disc and yellowish red between striae in apical 1/2 and laterally, with narrow, faint, reddish basal band, partly conjoined with yellowish red lateral area. Scutellum reddish. Antennae, other head appendages, and pro- and mesolegs reddish, metalegs darker, all legs darker distally. Ventral side piceous, paler on prosternum and posteriorly on abdominal ventrites.

***Surface sculpture***: Elytron with 11 dorsal striae, stria 1 reduced basally, submarginal stria present: 11+1 (Fig. 37).

Head without strioles, with dense punctation (spaces between punctures 1–3× size of punctures); punctures relatively fine (usually diameter of punctures equal to or smaller than diameter of microreticulation cells); microreticulation distinct. Pronotum with strioles posterolaterally, with punctation finer than on head and microreticulation fine. Elytron with 11 dorsal striae: stria 1 reduced basally, striae 2 and 3 interrupted basally; submarginal stria present. Elytron with fine, sparse punctation and fine microreticulation.

***Structures***: Head relatively broad. Pronotum trapezoid, its lateral margins convergent anteriorly. Base of prosternum rounded anteriorly, convex medially; blade of prosternal process relatively broad.

***Male***: Protibia straight, not modified. Proclaws relatively long, subequal in length, anterior claw thicker subapically and slightly more strongly curved downwards than posterior one and due to this with a median incision on its inner margin. Median lobe of aedeagus robust, with two lobes of dorsal sclerite covered with scale-like structures; left dorsal lobe without concavity, its whole apical 1/2 more or less evenly tapering to small, slightly curved downward and truncate apex bearing small crest; right dorsal lobe shorter than left one, broad, with pointed apex and membranous area medially; lobes of ventral sclerite partly sclerotised, pressed together between lobes of dorsal sclerite, slightly visible in left lateral view; left ventral lobe consists of two parts: left sclerotised area and less sclerotised right part; sclerotised area with elongate and broad basal 1/2 and long, thread-like apical 1/2; less sclerotised right part long and broad, with its apical part densely covered with long setae (sometimes visible at apex); right ventral lobe partly sclerotised, long and broad. Parameres with dense, long setae occupying approximately half of dorsal margin (Fig. 87).

***Female***: Unknown.

##### Affinities.

The species belongs to the large species with robust median lobe, covered mainly with scale-like structures. Among them, in shape of the median lobe, it is similar to *A.mamberamo* sp. nov. and differs from it in elytron paler apically, male anterior proclaw thicker subapically, with median incision of its inner margin, and apex of left dorsal lobe of the median lobe distinctly shorter, with smaller crest.

##### Etymology.

The species is named after Noiadi Village. The name is a noun in the nominative singular standing in apposition.

##### Distribution.

Indonesia: Papua Province: Mamberamo Raya Regency. The species is known only from the type locality (Fig. 108).

##### Habitat.

Unknown.

#### ﻿﻿30. Austrelatus normanbyensis sp. nov.

Taxon classificationAnimaliaColeoptera Dytiscidae

06694269-22D7-5A50-BCC0-C5882F8F3A0B

https://zoobank.org/5925A873-F633-4CCE-9E68-8D7F46852F2C

Figs 21 , 72 , 107

##### Type locality.

Papua New Guinea: Milne Bay Province, Normanby Island, Sewa Bay, Sibonai, 10°02.418'S, 150°58.461'E, 35 m a.s.l.

##### Type material.

***Holotype***: male “Papua New Guinea: Milne Bay, Normanby Isl., Sewa Bay, Sibonai, 35 m”, “30.vi.2017, 10°02.418’S 150°58.461’E, Riedel”, “8722” [green text] (MZB). ***Paratypes***: 3 males with the same lable as holotype and additional labels with green text “8723”, “8724” and “8727” (NHMW, SMNK, ZSM). 6 males, 4 females “Papua New Guinea: Milne Bay Prov., Normanby Isl., Sewa Bay, Sibonai,”, “10°02.418’S 150°58.461’E, 35 m, beaten & hand-collected,”, “30-VI-2017 – position 1, A. Riedel.” (NHMW, SMNK, ZSM). 3 males, 4 females “Papua New Guinea: Milne Bay Prov., Fergusson Isl., Salamo area, Dei Dei,”, “09°39.386’S 150°52.460’E to 09°39.230’S 150°52.586’E, 110 m,”, “beaten & hand-collected, 28-II-2017 – position 1 to 2, A. Riedel.” (NHMW, SMNK, ZSM). 1 male “Papua New Guinea: Milne Bay, Fergusson Isl., Salamo area, Dei Dei,”, “110 m, 28.iii.2017, 09°39.230’S 150°52.586’E, Riedel”, “8729” [green text] (SMNK).

##### Description.

***Body size and form***: Beetle large, with oblong-oval habitus (Fig. 21).

***Measurements***: TL 6.5–7 mm, TL-H 5.8–6.3 mm, MW 3.1–3.4 mm, TL/MW 2.06–2.12; PL 1–1.1 mm, PW 2.7–3 mm, PL/PW 0.36–0.37; DBE 1.1–1.2 mm, DBE/PW 0.4–0.41. ***Holotype***: TL 6.9 mm, TL-H 6.1 mm, MW 3.25 mm, TL/MW 2.12; PL 1 mm, PW 2.8 mm, PL/PW 0.36; DBE 1.15 mm, DBE/PW 0.41.

***Colouration***: With piceous elytra and pronotum and yellowish red head, pronotal sides, and broad basal band on elytron (Fig. 21).

Head yellowish red, narrowly piceous behind eyes. Pronotum piceous, yellowish red on sides, broader at anterior angles. Elytron piceous, with yellowish red broad basal band, often with yellowish on apex, sometimes expanded between striae laterally. Scutellum piceous. Antennae, other head appendages, and legs yellowish red, legs darker distally. Ventral side reddish brown, with paler prosternum.

***Surface sculpture***: Elytron with six dorsal striae, stria 1 reduced in basal 1/2, submarginal stria present: 6+1 (Fig. 21).

Head without strioles, with relatively dense punctation (spaces between punctures 1–3× size of punctures); punctures relatively coarse (diameter of punctures larger than or equal to diameter of microreticulation cells); microreticulation distinct. Pronotum with several strioles at posterolateral angles, with punctation finer than on head and microreticulation fine. Elytron with 6 dorsal striae, stria 1 absent in basal 1/2, submarginal stria present, sometimes only apically. Elytron with very fine, sparse punctation and fine microreticulation.

***Structures***: Head relatively broad. Pronotum trapezoid, its lateral margins distinctly convergent anteriorly. Base of prosternum broadly rounded anteriorly, distinctly convex medially; blade of prosternal process narrow.

***Male***: Protibia straight, not modified. Proclaws relatively short, subequal in length, anterior claw slightly thicker subapically and more strongly curved downwards than posterior one and due to this with a median incision on its inner margin. Median lobe of aedeagus robust, with two lobes of dorsal sclerite subequal in length, with pointed apexes and covered with scale-like structures; left dorsal lobe concave medially, with apex curved downwards, with small apical crest; right dorsal lobe with membranous area medially; lobes of ventral sclerite sclerotised, pressed together between lobes of dorsal sclerite, but partly visible in left lateral view; left ventral lobe consists of two parts: left sclerotised area (not visible in left lateral view) and less sclerotised right part (visible in left lateral view); sclerotised area with elongate and broad basal 1/2 and long, hair-like apical 1/2; less sclerotised right part long and broad, with a tuft of long setae; right ventral lobe long and broad. Parameres with dense, long setae occupying approximately half of dorsal margin; more distally situated setae longer than more proximal ones (Fig. 72).

***Female***: As male.

##### Variability.

There is a variation in the elytral striation and colouration described above.

##### Affinities.

The species belongs to the large species with robust median lobe, covered mainly with scale-like structures. Among them, it is similar to *A.inconstans* sp. nov., *A.bundunensis* sp. nov. and *A.centralensis* sp. nov. in body form and dorsal colouration, but differs from them in smaller size and median lobe with narrow, distinctly bent downwards apex of left dorsal lobe.

In addition to *A.normanbyensis* sp. nov., one more *Austrelatus* species, *A.garainensis*Shaverdo et al., 2023, is known from Normanby Island (see below). The species belongs to the *A.neoguineensis* species group, representatives of which have a differently shaped male median lobe without surface sculptures (Shaverdo et al. 2023). The species is distinctly smaller in size than *A.normanbyensis* sp. nov. and has 11 complete dorsal striae on the elytron.

##### Etymology.

The species is named after Normanby Island. The name is an adjective in the nominative singular.

##### Distribution.

Papua New Guinea: Milne Bay Province, Normanby and Fergusson islands (Fig. 107).

##### Habitat.

Unknown.

#### ﻿﻿31. Austrelatus ohu sp. nov.

Taxon classificationAnimaliaColeoptera Dytiscidae

E1ADA580-1468-5387-8F54-950D5DC418C2

https://zoobank.org/8DC9BEF8-E230-4BA7-B355-EF946F14FEA1

Figs 4 , 67 , 107

##### Type locality.

Papua New Guinea: Madang Province, 15 km southwest of Madang, Ohu Village.

##### Type material.

***Holotype***: male “PNG Madang Province OHU Village, 14.1.2001 leg.: Lukas Cizek Coll. HENDRICH” (NHMW). ***Paratypes***: 11 males, 21 females with the same label as the holotype (CLH, NHMW, ZSM). 1 male “NEW GUINEA 25 km. N Madang 3 km. W Sempi 27 I 1989 leg. M.&R. Hołyński” (ZSM).

##### Description.

***Body size and form***: Beetle small, elongate (Fig. 4).

***Measurements***: TL 4.35–5 mm, TL-H 3.9–4.25 mm, MW 1.9–2.23 mm, TL/MW 2.24–2.29; PL 0.7–0.8 mm, PW 1.73–1.95 mm, PL/PW 0.39–0.42; DBE 0.8–0.95 mm, DBE/PW 0.46–0.5. ***Holotype***: TL 4.8 mm, TL-H 4.25 mm, MW 2.1 mm, TL/MW 2.29; PL 0.75 mm, PW 1.95 mm, PL/PW 0.39; DBE 0.9 mm, DBE/PW 0.46.

***Colouration***: Dorsally dark brown, with yellowish red head, pronotal sides and basal band on elytron (Fig. 4).

Head yellowish red, narrowly darker behind eyes. Pronotum brown to dark brown on disc and gradually paler (to yellowish red) to sides. Elytron brown to dark brown, with distinct yellowish red basal band and often yellow apex. Scutellum yellowish red to brown. Antennae, other head appendages, and pro- and mesolegs yellowish to yellowish red, metalegs darker, all legs darker distally. Ventral brown. Specimens slightly to strongly teneral.

***Surface sculpture***: Elytron with six dorsal striae and usually with submarginal stria: 6+(0–1) (Fig. 4).

Head without strioles, with distinct, sparse punctation (spaces between punctures 2–5× size of punctures); punctures relatively fine (diameter of punctures more or less equal to diameter of microreticulation cells); microreticulation distinct. Pronotum without strioles, with punctation as on head and microreticulation distinct. Elytron with six dorsal striae, sometimes striae 1, 3, 5, and 6 shortly reduced basally and/or interrupted, stria 1 can be more strongly reduced; submarginal stria present, seldom absent or represented by tiny strioles. Elytron with fine punctation and microreticulation.

***Structures***: Head large and broad. Pronotum slightly trapezoid, its lateral margins subparallel. Base of prosternum broadly rounded anteriorly, convex medially; blade of prosternal process relatively broad.

***Male***: Protibia straight, not modified. Proclaws very long, straight, subequal in length. Median lobe of aedeagus with two lobes of dorsal sclerite subequal in length, with broadly pointed apexes; left dorsal lobe with lateral margin slightly concave apically and lateral side slightly concave medially, covered laterally with numerous distinct spinula-like structures and dorsally with less numerous scale-like structures, lateral margin without surface structures, smooth; right dorsal lobe covered with scale-like structures; lobes of ventral sclerite partly sclerotised, long, partly visible in left lateral view; left ventral lobe with elongate, narrow sclerotised area, concave apically, with apex pointed and slightly curved; right ventral lobe longer than left lobe, with narrow sclerotised area on right margin, membranous part with long, thin, upwardly curved apex distinctly sticking out in left lateral view. Parameres with relatively dense and long setae occupying distinctly more than half of dorsal margin; setae on small area proximally shorter and sparser, with several most proximal setae standing separately (Fig. 67).

***Female***: Only matt, strongly striolated form known.

##### Variability.

There is an insignificant variation in the elytral striation.

##### Affinities.

In body size and shape, elytral striation and dorsal colouration, the species is similar to *A.procerus* sp. nov. and *A.lopintolensis* sp. nov. but distinctly differs from them by its long, straight male proclaws and shape of the median lobe. See also under *A.iriatoi* sp. nov.

##### Etymology.

The species is named after its type locality, Ohu Village. The name is a noun in the nominative singular standing in apposition.

##### Distribution.

Papua New Guinea: Madang Province (Fig. 107).

##### Habitat.

Unknown.

#### ﻿﻿32. Austrelatus papuensis

Taxon classificationAnimaliaColeoptera Dytiscidae

(Balfour-Browne, 1939)

8511C43F-9EE0-5B68-8069-1B6A8B6DC280

Figs 34 , 35 , 85 , 108

Copelatus papuensis Balfour-Browne 1939: 86; Guignot (1956: 52, 55); Guéorguiev (1968: 10); Guéorguiev and Rocchi (1993: 159); Nilsson and Hájek (2023: 65).
Austrelatus papuensis (Balfour-Browne, 1939): Shaverdo et al. (2023: 7).

##### Type locality.

Papua New Guinea: Central Province, Kokoda, 08°52'28.4"S, 147°44'16.1"E, ca. 365 m a.s.l. Note: In Balfour-Browne (1939: 87), it is given as “♂ holotype, ♀ allotype, 14 ♂♂ and 10 ♀♀ paratypes, Papua: Kokoda, 1200ft., v.1933 (*Miss Chesman*) (British Museum, 1933-577).”

##### Type material.

***Holotype***: male “♂” [hw, next to beetle], “Type” [round label with red frame], “PAPUA: Kokoda. 1,200ft. v.1933. L.E.Cheesman. B.M.1933-577.”, “57” [hw on reverse side], “Copelatuspapuensis, B-B. ♂ TYPE” [hw by J. Balfour-Browne], “Manuscript name” [printed in red], “Holotype” [red label] (BMNH). ***Paratypes***: 1 female “♀” [hw, next to beetle], “Type” [round label with red frame], “PAPUA: Kokoda. 1,200ft. v.1933. L.E.Cheesman. B.M.1933-577.”, “57” [hw on reverse side], “Copelatuspapuensis, B-B. ♀ TYPE” [hw by J. Balfour-Browne] (BMNH). 1 male “♂” [hw, next to genitalia], “Type” [round label with yellow frame], “PAPUA: Kokoda. 1,200ft. v.1933. L.E.Cheesman. B.M.1933-577.”, “57” [hw on reverse side], “Copelatuspapuensis, B-B. ♂” [hw by J. Balfour-Browne] (BMNH). 8 males, 7 females “PAPUA: Kokoda. 1,200ft. v.1933. L.E.Cheesman. B.M.1933-577.”, “57” [hw on reverse side], “Type” [round label with yellow frame], “Copelatuspapuensis, B-B. ♂ [or ♀]” [hw by J. Balfour-Browne] (BMNH). 1 male “Under stones: river side.”, “PAPUA: Kokoda. 1,200ft. v.1933. L.E.Cheesman. B.M.1933-577.”, “57” [hw on reverse side], “Type” [round label with yellow frame], “Copelatuspapuensis, B-B. ♂” [hw by J. Balfour-Browne] (BMNH). 1 female “♀” [hw, next to beetle], “Type” [round label with yellow frame], “PAPUA: Kokoda. 1,200ft. v.1933. L.E.Cheesman. B.M.1933-577.”, “57” [hw on reverse side], “Copelatuspapuensis, B-B. ♀” [hw by J. Balfour-Browne] (BMNH). 1 female “♀” [hw, next to beetle], “Type” [round label with yellow frame], “PAPUA: Kokoda. 1,200ft. v.1933. L.E.Cheesman. B.M.1933-577.”, “57” [hw on reverse side], “Copelatuspapuensis, B-B. ♀” [hw by J. Balfour-Browne], “measured J. Parkin 73” (BMNH). 1 male “♂” [hw, next to beetle], “Type” [round label with yellow frame], “PAPUA: Kokoda. 1,200ft. v.1933. L.E.Cheesman. B.M.1933-577.”, “57” [hw on reverse side], “Copelatuspapuensis, B-B. ♂” [hw by J. Balfour-Browne] (BMNH). 1 male “PAPUA: Kokoda. 1,200ft. v.1933. L.E.Cheesman. B.M.1933-577.”, “57” [hw on reverse side], “Type” [round label with yellow frame], “Copelatuspapuensis, B-B. ♂” [hw by J. Balfour-Browne], “measured J. Parkin 72” (BMNH).

##### Additional material.

***PNG*: *Morobe***: 188 males, 98 females “Papua New Guinea: Garaina, 720m, vi.2008 07 51.032S 147 07.007E Ibalim & Sosanika (PNG216)” (NHMW, ZSM). 32 males, 33 females “Papua New Guinea: Garaina 800m, vi.2008 07.53.091S 147.07.915E Ibalim & Sosanika, PNG217” (NHMW, ZSM). 16 males, 9 females “Papua New Guinea: Garaina, 770m, vi.2008 07 52.516S 147.10.427E Ibalim & Sosanika (PNG219)”, one of males additionally with a green label “4074” (NHMW, ZSM). 4 males, 1 female “Papua New Guinea: Garaina, 800m, 27.vi.2009 (PNG220) 7.52.669S 147.07.196E Ibalim & Sosanika”, one of males additionally with a green label “3845” (ZSM). 19 males, 11 females “Papua New Guinea: Garaina, 770m, 25.vi.2008, 07.50.859S 147.08.614E, Ibalim & Sosanika, (PNG222)” (NHMW, ZSM). 1 female “Papua New Guinea: Garaina, 820m, 24.vi.2008 07.52.287S 147.06.297E, Ibalim & Sosanika, (PNG224)” (ZSM). ***EHL***: 1 male, 1 female “Papua New Guinea: Eastern Highlands, Bena Brigde, Unggai Bena, 1393m, 27.viii.2005, K.Sagata, (WB136)” (ZSM).

##### Description.

***Body size and form***: Beetle large, oblong-oval or elongate (Figs 34, 35).

***Measurements***: TL 6.9–7.8 mm, TL-H 6.35–7.1 mm, MW 3.25–3.6 mm, TL/MW 2.12–2.23; PL 0.95–1.15 mm, PW 2.85–3.2 mm, PL/PW 0.33–0.37; DBE 1.2–1.3 mm, DBE/PW 0.4–0.42. ***Holotype***: TL 7.5 mm, TL-H 6.8 mm, MW 3.4 mm, TL/MW 2.21; PL 1.15 mm, PW 3.1 mm, PL/PW 0.37; DBE 1.3 mm, DBE/PW 0.42.

***Colouration***: Elytron yellowish red to reddish brown between striae, narrowly piceous on pronotal and elytral discs, with yellowish red head and pronotal sides (Figs 34, 35).

Head yellowish red, narrowly darker behind eyes. Pronotum piceous on disc and gradually paler (to yellowish red) to sides. Elytron narrowly brown to piceous on disc, with yellowish red basal band broadened at shoulder area, yellow apex, and usually intensive yellowish red colouration between striae, striae brown to piceous. Scutellum yellowish red to brown. Antennae, other head appendages, and pro- and mesolegs yellowish red to reddish, metalegs darker, all legs darker distally. Ventral side reddish brown, paler on prosternum and abdominal ventrites.

***Surface sculpture***: Elytron with 10–11 dorsal striae, striae 1–3 reduced in basal 1/2, submarginal stria present: (10–11)+1 (Figs 34, 35).

Head without strioles, with dense punctation (spaces between punctures 1–3× size of punctures); punctures relatively fine (usually diameter of punctures equal to or smaller than diameter of microreticulation cells); microreticulation distinct. Pronotum with few to several strioles posterolaterally, more numerous in females, with punctation finer than on head and microreticulation fine. Elytron with 10 or 11 dorsal striae: striae 1–3 reduced in basal 1/2, stria 1 strongly to completely reduced, striae 5 and 9 can be interrupted; submarginal stria present, often as apical strioles. Elytron with fine, sparse punctation and fine microreticulation.

***Structures***: Head relatively broad. Pronotum trapezoid, its lateral margins convergent anteriorly, seldom rounded so that pronotal base slightly broader than that of elytra. Base of prosternum broadly rounded anteriorly, convex medially; blade of prosternal process relatively broad.

***Male***: Protibia straight, not modified. Proclaws long, subequal in length, anterior claw thicker subapically and often more strongly curved downwards than posterior one and due to this with a median incision on its inner margin. Median lobe of aedeagus robust, with two lobes of dorsal sclerite covered with scale-like structures; left dorsal lobe without distinct median concavity and its whole apical 1/2 more or less evenly tapering to elongate, slightly curved, evenly or unevenly narrowly rounded apex bearing small crest; right dorsal lobe shorter, with membranous area medially; lobes of ventral sclerite partly sclerotised, pressed together between lobes of dorsal sclerite, slightly visible in left lateral view; left ventral lobe consists of two parts: left sclerotised area and less sclerotised right part; sclerotised area with elongate and broad basal 1/2 and long, thread-like apical 1/2; less sclerotised right part long and broad, with its apical part densely covered with long setae; right ventral lobe partly sclerotised, long and broad. Parameres with dense, long setae occupying approximately half of dorsal margin (Fig. 85).

***Female***: Dimorphic: as male but with more numerous pronotal strioles and matt, with pronotum and elytron very densely covered with fine longitudinal strioles. Matt forms seldom; in the studied material, known only from the PNG219 population, with ratio shiny to with strioles is 7:3.

##### Variability.

There is a variation in dorsal colouration and striolation described above as well as in shape of left dorsal lobe apex of the median lobe: more or less rounded and curved even within one population. Less rounded, curved apex is characteristic of the holotype.

##### Affinities.

The species belongs to the large species with robust median lobe, covered mainly with scale-like structures. Among them, it is similar to *A.posmani* in shape of the median lobe and differs from it in weaker elytral striation, with striae 1–3 reduced in basal 1/2 and stria 1 sometimes absent, male anterior proclaw with median incision of its inner margin, and apex of left dorsal lobe of the median lobe broader, with more distinct crest. See also under *A.posmani* sp. nov.

##### Distribution.

Papua New Guinea: Central, Morobe, and Eastern Highlands provinces (Fig. 108). According to Guignot (1956: 52), the species occurs also in Madang Province, Stephansort, Astrolabe Bay; however, this record has not been confirmed. During our study of Guignot’s collection in MNHN, the specimen, on which the records were based, has been found and indentified as *A.madangensis* sp. nov. (see above).

##### Habitat.

Unknown.

#### ﻿﻿33. Austrelatus posmani sp. nov.

Taxon classificationAnimaliaColeoptera Dytiscidae

B469F43A-FF56-5C66-8D4E-6ED358C07361

https://zoobank.org/098A9F81-BDCA-49E6-BAFF-B64AA2DE553D

Figs 33 , 84 , 108

##### Type locality.

Papua New Guinea: Central Province, Moreguina, 10°00'57"S, 148°28'27"E.

##### Type material.

***Holotype***: male “Papua New Guinea: Central, Moreguina 16.viii.2008 Posman (PNG183)” (ZSM). ***Paratypes***: 1 male with the same label as the holotype (ZSM).

##### Description.

***Body size and form***: Beetle large, oblong-oval (Fig. 33).

***Measurements***: TL 7–7.7 mm, TL-H 6.3–6.9 mm, MW 3.4–3.6 mm, TL/MW 2.06–2.14; PL 1–1.2 mm, PW 2.9–3.15 mm, PL/PW 0.35–0.38; DBE 1.15–1.2 mm, DBE/PW 0.38–0.4. ***Holotype***: TL 7 mm, TL-H 6.3 mm, MW 3.6 mm, TL/MW 2.06; PL 1 mm, PW 2.9 mm, PL/PW 0.35; DBE 1.15 mm, DBE/PW 0.4.

***Colouration***: With almost completely yellowish red elytron, yellowish red head and pronotal sides (Fig. 33).

Head yellowish red, narrowly darker behind eyes. Pronotum piceous on disc and broadly yellowish red on sides. Elytron yellowish red due to strongly developed basal band and colouration between striae, piceous on small part of disc, on striae and very narrowly on sides. Scutellum yellowish red. Antennae, other head appendages, and pro- and mesolegs yellowish red, metalegs darker, all legs darker distally. Ventral side reddish brown, paler on prosternum and abdominal ventrites.

***Surface sculpture***: Elytron with 11 complete, strongly impressed dorsal striae, submarginal stria present: 11+1 (Fig. 33).

Head without strioles, with dense punctation (spaces between punctures 1–3× size of punctures); punctures relatively fine (usually diameter of punctures equal to or smaller than diameter of microreticulation cells); microreticulation distinct. Pronotum with strioles laterally, mainly at posterior margin, with punctation slightly finer than on head and microreticulation fine. Elytron with 11 complete, strongly impressed dorsal striae: stria 1 shortly reduced basally; submarginal stria present, long. Elytron with fine, sparse punctation and fine microreticulation.

***Structures***: Head relatively broad. Pronotum trapezoid, its lateral margins convergent anteriorly, distinctly rounded so that pronotal base slightly broader than that of elytra. Base of prosternum broadly rounded anteriorly, convex medially; blade of prosternal process broad.

***Male***: Protibia more or less straight: its ventral margin very slightly curved proximally. Proclaws long, slightly curved, subequal in length, anterior claw thicker at its apex than posterior one and due to this with a subapical incision on its inner margin. Median lobe of aedeagus robust, with two lobes of dorsal sclerite covered with scale-like structures, with their apexes broadly pointed; left dorsal lobe without distinct median concavity and its whole apical 1/2 relatively narrow, more or less evenly tapering to elongate, straight apex bearing small crest; right dorsal lobe shorter, with small membranous area medially; lobes of ventral sclerite partly sclerotised, pressed together between lobes of dorsal sclerite, slightly visible in left lateral view; left ventral lobe consists of two parts: left sclerotised area and less sclerotised right part; sclerotised area with elongate and broad basal 1/2 and long, thread-like apical 1/2; less sclerotised right part long and broad, with its apical part densely covered with long setae; right ventral lobe partly sclerotised, long and broad. Parameres with dense, long setae occupying approximately half of dorsal margin (Fig. 84).

***Female***: Unknown.

##### Affinities.

The species belongs to the large species with robust median lobe, covered mainly with scale-like structures. Among them, it is distinguished by yellow colouration and strong striation of its elytra. It is similar to *A.papuensis* in shape of the median lobe and differs from it in stronger elytral striation, male anterior proclaw thicker at its apex than posterior one and with subapical incision of its inner margin, and apex of left dorsal lobe of the median lobe narrower, with crest weaker. See also under *A.papuensis* sp. nov.

##### Etymology.

The species is named after its collector A. Posman. The name is a noun in the genitive case.

##### Distribution.

Papua New Guinea: Central Province. The species is known only from the type locality (Fig. 108).

##### Habitat.

Unknown.

#### ﻿﻿34. Austrelatus procerus sp. nov.

Taxon classificationAnimaliaColeoptera Dytiscidae

D10CB8BF-F431-53CD-BCD9-9447AA3442DC

https://zoobank.org/C6D50444-65C7-49B4-B2F5-0AF9A04CA0B5

Figs 2 , 60 , 107

##### Type locality.

Indonesia: West Papua Province: Sorong Regency, road Sorong-Teminabuan, 01°08'08.6"S, 131°54'00.1"E, 130 m a.s.l.

##### Type material.

***Holotype***: male “Indonesia: Papua Barat, Sorong-Teminabuan, 130 m, 2.x.2014, -1,1357267 131,9000149, UNIPA team (BH047)” (KSP). ***Paratypes***: 2 females “Indonesia: Papua Barat, Sorong-Teminabuan, 50 m, 2.x.2014, -1,1092904 131,6125645, B. Sumoked (BH046)”, with two green text labels “6453” and “6454”, respectively (MZB, ZSM).

##### Description.

***Body size and form***: Beetle small, distinctly elongate (Fig. 2).

***Measurements***: TL 4.5–4.55 mm, TL-H 4.1 mm, MW 2–2.05 mm, TL/MW 2.22–2.25; PL 0.7–0.75 mm, PW 1.83–1.9 mm, PL/PW 0.38; DBE 0.85–0.9 mm, DBE/PW 0.47. ***Holotype***: TL 4.5 mm, TL-H 4.1 mm, MW 2.05 mm, TL/MW 2.22; PL 0.75 mm, PW 1.9 mm, PL/PW 0.38; DBE 0.9 mm, DBE/PW 0.47.

***Colouration***: Elytra piceous, head and pronotum paler; yellow basal band on elytron distinct and broad (Fig. 2).

Head yellowish red, narrowly darker behind eyes. Pronotum dark brown on disc and gradually paler (to yellowish red) to sides. Elytron piceous, with broad yellow basal band and yellow apex. Scutellum yellowish red. Antennae, other head appendages, and pro- and mesolegs yellowish to yellowish red, metalegs darker, all leg darker distally. Ventral side yellowish to yellowish red.

***Surface sculpture***: Elytron without striae or with 6 weakly impressed striae, submarginal stria absent: (0–6)+0 (Fig. 2).

Head without strioles, with distinct, uneven punctation (spaces between punctures 1–5× size of punctures); punctures fine (diameter of punctures smaller than or equal to diameter of microreticulation cells); microreticulation distinct. Pronotum without or with short, weak strioles on sides, with punctation sparser than on head and microreticulation distinct. Elytron without striae, with four puncture lines, or as in holotype, with six weakly impressed striae: striae 2 and 4 complete, other striae strongly reduced, especially basally. Elytron with fine, sparse punctation and distinct microreticulation. Abdominal ventrite 6 with distinct punctation sparse medially and forming denser area at each lateral side, with few strioles laterally.

***Structures***: Head broad. Pronotum trapezoid, its lateral margins convergent anteriorly. Base of prosternum rounded anteriorly, convex medially; blade of prosternal process short and broad.

***Male***: Protibia straight, not modified. Proclaws long, subequal in length, anterior claw thicker subapically and more strongly curved downwards than posterior one and due to this with a median incision on its inner margin. Median lobe of aedeagus with two lobes of dorsal sclerite subequal in length, covered with scale-like structures; left dorsal lobe with lateral margin concave apically, with its side concave subapically and medially, and with some spinula-like structures medially in left lateral view; apex of left dorsal lobe straight, with thickened tip; left lobe of ventral sclerite with elongate, narrow sclerotised area, with its apex visible in left lateral view; right lobe of ventral sclerite membranous. Parameres with relatively long and dense setae occupying distinctly more than half of dorsal margin; more distally situated setae denser and longer than more proximal ones (Fig. 60).

***Female***: As male, but elytron without striae and pronotum with numerous, weak strioles on its sides.

##### Variability.

There is a variation in the elytral striation described above: the holotype has elytron with 6 dorsal striae and both females are without elytral striae.

##### Affinities.

The species is similar to *A.leptos* sp. nov. in small size, elongate habitus, and dorsal colouration, but differs from it in sturdier habitus, convergent anteriorly pronotal lateral margins, elytron with striae and broader yellow basal band, and different shape of the median lobe and male proclaws.

##### Etymology.

The species name is a Latin adjective meaning slender, and indicates the elongate habitus of the species.

##### Distribution.

Indonesia: West Papua Province: Sorong Regency. The species is known only from the type locality (Fig. 107).

##### Habitat.

The species was collected in small forest pools rich in rotten leaves.

#### ﻿﻿35. Austrelatus pseudogestroi sp. nov.

Taxon classificationAnimaliaColeoptera Dytiscidae

75C28A58-D825-5C6D-98A0-587CF0403E91

https://zoobank.org/B222C677-92BA-47E9-AB57-E3135B282E2E

Figs 53 , 102 , 108

##### Type locality.

Papua New Guinea: National Capital District Province, Port Moresby, Brown River Road situated nearby to the suburbs Morata and Waigani.

##### Type material.

***Holotype***: male “Stn. No. 195”, “Papua: Pt. Moresby Brown R. Rd., 15.III.1965”, “M.E.Bacchus, B.M. 1965-120” (BMNH).

##### Description.

***Body size and form***: Beetle medium-sized, oblong-oval (Fig. 53).

***Measurements*: *Holotype***: TL 6.9 mm, TL-H 6.2 mm, MW 3.3 mm, TL/MW 2.09; PL 0.95 mm, PW 2.7 mm, PL/PW 0.35; DBE 1 mm, DBE/PW 0.37.

***Colouration***: With piceous elytra and pronotum and yellowish red head, pronotal sides, and elytral basal band and apex (Fig. 53).

Head almost completely yellowish red, narrowly piceous behind eyes and with V-shaped, brown spot medially. Pronotum piceous on disc and broadly yellowish red on sides, especially at anterior angles. Elytron piceous, with rather broad yellowish red basal band; elytron broadly yellow apically, usually extending between striae, especially laterally (up to elytral half). Scutellum piceous. Antennae, other head appendages, and pro- and mesolegs yellowish red, metalegs darker, especially distally. Ventral side piceous, with paler prosternum.

***Surface sculpture***: Elytron with 11+1 striae, striae 1–3 reduced basally (Fig. 53).

Head without strioles, with relatively dense punctation (spaces between punctures 1–3× size of punctures); punctures relatively fine (diameter of punctures usually equal to and smaller than diameter of microreticulation cells); microreticulation distinct. Pronotum without strioles, with punctation finer than on head and microreticulation fine. Elytron with 11 dorsal striae, stria 1 absent in basal 1/2 and interrupted in apical 1/2; striae 2 and 3 absent in basal ¼ and additionally interrupted basally; submarginal stria present, well-developed. Elytron with fine punctation and microreticulation.

***Structures***: Head relatively broad. Pronotum trapezoid, its lateral margins convergent anteriorly. Base of prosternum rounded anteriorly, convex medially; blade of prosternal process relatively narrow.

***Male***: Protibia straight, not modified. Proclaws relatively long; anterior claw shorter, thicker and more curved than posterior one and due to this with a median incision on its inner margin. Median lobe of aedeagus relatively broad, with two lobes of dorsal sclerite with narrowly rounded apexes, left straight, right slightly curved to left; left dorsal lobe slightly longer than right dorsal lobe, distinctly narrowed to apex in its apical 1/3; lateral side of left dorsal lobe covered with large scale-like structures towards dorsal side and smaller scale-like structures towards apex, with numerous small spinula-like structures on slightly beaded lateral margin and numerous large, short spinula-like structures medially; right dorsal lobe covered with large scale-like structures, with small median membranous area; lobes of ventral sclerite sclerotised, long, broad, partly visible in left lateral view; left ventral lobe with elongate, strong sclerotised area, with curved apex and broad slightly sclerotised part rounded apically; right ventral lobe distinctly longer than left one, slightly sclerotised, with long curved apex visible in left lateral view. Parameres with dense, long setae occupying distinctly more than half of dorsal margin; on small area proximally very distinctly shorter and sparser (Fig. 102).

***Female***: Unknown.

##### Affinities.

The species belong to the medium-sized species with median lobe of the aedeagus that has scale- and spinula-like structures on the left dorsal lobe and among them, to the species with surface of left dorsal lobe completely covered by scale- and spinula-like structures in left lateral and ventral views. Based on dorsal colouration and general shape of the median lobe, it is similar to *A.asteios* sp. nov., *A.epicharis* sp. nov., *A.wasurensis* sp. nov., and *A.yeretuar* sp. nov. but differs from them in larger body size and left dorsal lobe of the median lobe with narrow, shallow, concavity under the lateral margin and different organisation of the surface structures. In body size, shape and colouration, it is very similar to *A.gestroi* sp. nov. but differs from it in the elytron with a broader yellowish red basal band and different shape of the median lobe: the left dorsal lobe is broader and its apical part is distinctly shorter.

##### Etymology.

The species is named after its similarity to *A.gestroi*. The name is a noun in the nominative singular standing in apposition.

##### Distribution.

Papua New Guinea: National Capital District. The species is known only from the type locality (Fig. 108).

##### Habitat.

Unknown.

#### ﻿﻿36. Austrelatus pseudomianminensis sp. nov.

Taxon classificationAnimaliaColeoptera Dytiscidae

2E6B4695-A960-5A9C-B938-19E739B1B3A4

https://zoobank.org/352382ED-8FBA-4E4C-940C-D57E39B82C64

Figs 45 , 94 , 108

##### Type locality.

Indonesia: Papua Province: Puncak Regency, Iratoi, Rouaffer River, 3°14'25.1"S, 137°19'58.7"E, 164 m a.s.l.

##### Type material.

***Holotype***: male “Indonesia: Papua, Rouaffer, Iratoi, hill in forest, 164m, 6.ix.2014, -3,2403086 137,3329744, UNCEN team (PAP028)” (KSP). ***Paratypes***: 2 females with the same label as the holotype (MZB, ZSM).

##### Description.

***Body size and form***: Beetle medium-sized, oblong-oval (Fig. 45).

***Measurements***: TL 5.3–5.5 mm, TL-H 4.95–5.1 mm, MW 2.6–2.7 mm, TL/MW 2.04–2.06; PL 0.75–0.8 mm, PW 2.3 mm, PL/PW 0.33–0.35; DBE 0.85 mm, DBE/PW 0.37. ***Holotype***: TL 5.5 mm, TL-H 5.05 mm, MW 2.7 mm, TL/MW 2.04; PL 0.8 mm, PW 2.3 mm, PL/PW 0.35; DBE 0.85 mm, DBE/PW 0.37.

***Colouration***: Dorsally piceous, with yellowish red to reddish brown head, anterior angles of pronotum, and yellow spot on elytral apex (Fig. 45).

Head yellowish red to reddish, narrowly piceous behind eyes. Pronotum piceous, yellowish red to reddish on anterior half of sides. Elytron piceous, with large yellow apical spot, slightly extending laterally. Scutellum reddish to piceous. Antennae, other head appendages, and pro- and mesolegs yellowish red, metalegs distinctly darker, especially distally. Ventral side piceous, with brown prosternum.

***Surface sculpture***: Elytron with 11+1 complete, rather strongly impressed striae (Fig. 45).

Head without strioles, with rather dense punctation (spaces between punctures 1–3× size of punctures); punctures rather coarse (diameter of punctures usually equal to diameter of microreticulation cells); microreticulation distinct. Pronotum with distinct strioles broadly laterally, only disc without them, with punctation finer than on head and microreticulation fine. Elytron with 11 complete, rather strongly impressed dorsal striae, stria 10 shortly reduced basally; submarginal stria present. Elytron with fine and microreticulation.

***Structures***: Head relatively broad. Pronotum trapezoid, its lateral margins convergent anteriorly. Base of prosternum rounded anteriorly, convex medially; blade of prosternal process relatively narrow.

***Male***: Protibia straight, not modified. Proclaws relatively long, slightly curved, subequal in length, anterior claw thicker at its apex than posterior one and due to this with a subapical incision on its inner margin. Median lobe of aedeagus with two lobes of dorsal sclerite subequal in length, more or less straight, with very broadly pointed apexes, apex of left dorsal lobe slightly curved downwards; left dorsal lobe with lateral side covered with numerous, distinct, strong spinula-like structures; lateral margin apically without surface structures, smooth; right dorsal lobe covered with scale-like structures, with distinct median membranous area; lobes of ventral sclerite partly sclerotised, long, subequal in length, partly visible in left lateral view; left ventral lobe with elongate, strong sclerotised area, concave subapically, with apex pointed and slightly curved to right and membranous right part long, thin, apically covered with setae sometimes visible in left lateral view; right ventral lobe with narrow sclerotised area on right margin. Parameres with dense, long setae occupying approximately half of dorsal margin; more distally situated setae longer than more proximal ones (Fig. 94).

***Female***: As males but with stronger dorsal punctation.

##### Affinities.

The species belong to the medium-sized species with median lobe of the aedeagus that has scale- and spinula-like structures on the left dorsal lobe and among them, to the species with surface of left dorsal lobe not completely covered by spinula-like structures, upper lateral margin without spinulae, smooth in left lateral and ventral views. Based on shape of the median lobe, it is very similar to *A.mianminensis* sp. nov. but differs from it in smaller body size, elytral striae complete and rather strongly impressed, more numerous pronotal strioles, and smaller median lobe, with the lateral margin of left dorsal lobe with edge, below which spinula-like structures situated, more sharp, prominent. See also under *A.flavocapitatus* sp. nov. and *A.sararti* sp. nov.

##### Etymology.

At the beginning, the species was considered to belong to *A.mianminensis* sp. nov. due to their similarity. The name is a noun in the nominative singular standing in apposition.

##### Distribution.

Indonesia: Papua Province: Puncak Regency. The species is known only from the type locality (Fig. 108).

##### Habitat.

The species was collected in small and shaded forest pools rich in rotten leaves.

#### ﻿﻿37. Austrelatus robustus sp. nov.

Taxon classificationAnimaliaColeoptera Dytiscidae

C0B3781B-DFC1-5F8D-B237-62EB5076DC5A

https://zoobank.org/0747FD25-78D8-4E39-8E01-6BD1DC547A62

Figs 30 , 81 , 107

##### Type locality.

Papua New Guinea: Madang Province, Highway near Madang, 05°24.405'S, 145°38.213'E, 80 m a.s.l.

##### Type material.

***Holotype***: male “Papua New Guinea: Madang, Highway nr. Madang, ford, 80m, 26.xi./2.-3.xii.1994, 05.24.405S 145.38.213E, Binatang Boys, (PNG 117)” (ZSM). ***Paratypes*: *PNG*: *Madang***: 1 male with the same label as the holotype (NHMW). ***Central***: 1 male “Papua New Guinea: Central, Myola, 1110m, i.2008, [09]12.630S 147 31.880E, Posman (PNG 177)”, “4119” [green label] (ZSM).

##### Description.

***Body size and form***: Beetle large, oblong-oval (Fig. 30).

***Measurements***: TL 7.45–7.9 mm, TL-H 6.8–7.1 mm, MW 3.75–3.9 mm, TL/MW 1.99–2.03; PL 1.1–1.2 mm, PW 3.35–3.6 mm, PL/PW 0.33–0.33; DBE 1.3–1.45 mm, DBE/PW 0.39–0.4. ***Holotype***: TL 7.45 mm, TL-H 6.8 mm, MW 3.75 mm, TL/MW 1.99; PL 1.1 mm, PW 3.35 mm, PL/PW 0.33; DBE 1.3 mm, DBE/PW 0.39.

***Colouration***: Dorsally piceous, with reddish head, pronotal sides and elytral base and apex (Fig. 30).

Head red, narrowly darker behind eyes. Pronotum brown on disc and gradually paler (to dark yellowish red) to sides, especially at anterior angles. Elytron dark brown to piceous, with short, faint, dark yellowish red to reddish basal band present on shoulder area and with yellowish red apex, sometimes more developed laterally. Scutellum dark brown. Antennae, other head appendages, and pro- and mesolegs reddish, metalegs darker, all legs darker distally. Ventral side dark brown, sometimes paler medially and on abdominal ventrites.

***Surface sculpture***: Elytron with 11 complete striae, submarginal stria present: 11+1 (Fig. 30).

Head without strioles, with dense punctation (spaces between punctures 1–3× size of punctures); punctures coarse (usually diameter of punctures larger than diameter of microreticulation cells); microreticulation distinct. Pronotum with few strioles on sides, with punctation finer than on head and microreticulation fine. Elytron with 11 complete striae, striae 1 and 10 usually shortly reduced basally; submarginal stria present. Elytron with fine, sparse punctation and fine microreticulation.

***Structures***: Head relatively broad. Pronotum trapezoid, its lateral margins convergent anteriorly. Base of prosternum rounded anteriorly, convex medially; blade of prosternal process relatively broad.

***Male***: Protibia modified: thinner proximally and broader medially and distally due to its curved ventral margin. Proclaws relatively long, subequal in length, anterior claw thicker subapically and more strongly curved downwards than posterior one and due to this with a distinct median incision on its inner margin. Median lobe of aedeagus very robust, with two lobes of dorsal sclerite covered with scale-like structures; left dorsal lobe very strongly concave medially, with apical 1/2 very broad and apex slightly rounded and bearing small but rather distinct crest; right dorsal lobe shorter, narrower, more or less evenly tapering to pointed apex, with small membranous area medially; lobes of ventral sclerite mostly visible in left lateral view; left ventral lobe consists of two parts: left sclerotised area and less sclerotised right part; sclerotised area with elongate and broad basal 1/2 (visible in left lateral view) and long, thread-like apical 1/2 (not visible); less sclerotised right part long and broad, with its apical part densely covered with long setae (partly visible in left lateral view); right ventral lobe mostly sclerotised, long and broad (partly visible in left lateral view). Parameres with dense, long setae occupying slightly more than half of dorsal margin, with proximal setae shorter (Fig. 81).

***Female***: Unknown.

##### Variability.

There is an insignificant variation in dorsal colouration described above.

##### Affinities.

The species belongs to the large species with robust median lobe, covered mainly with scale-like structures. Among them, it is very similar to *A.bosaviensis* sp. nov. and differs from it in broader male anterior claw with median inner margin incision and narrower apical 1/2 of the left dorsal lobe of the median lobe. See also under *A.bosaviensis* sp. nov.

##### Etymology.

The species name is a Latin adjective meaning “hard and strong like oak” and indicates the large, broad, robust habitus and median lobe of the species.

##### Distribution.

Papua New Guinea: Madang and Central provinces (Fig. 107).

##### Habitat.

In Madang, the species was collected at a stream ford.

#### ﻿﻿38. Austrelatus sararti sp. nov.

Taxon classificationAnimaliaColeoptera Dytiscidae

001C7378-7762-5B19-8FE4-82F87494A13F

https://zoobank.org/E8A96AB9-9877-4834-B567-53D5AA379FEF

Figs 48 , 98 , 108

##### Type locality.

Indonesia: West Papua: Teluk Wondama Regency, Wasior, Sararti 100–200 m a.s.l.

##### Type material.

***Holotype***: male “Irian Jaya: Wandammen Bay, Wasior, Sararti 100–200 m, 7.-9.I.2001, leg A. Riedel” (SMNS).

##### Description.

***Body size and form***: Beetle medium-sized, oblong-oval (Fig. 48).

***Measurements*: *Holotype***: TL 5.7 mm, TL-H 5.1 mm, MW 2.75 mm, TL/MW 2.07; PL 0.85 mm, PW 2.35 mm, PL/PW 0.36; DBE 0.95 mm, DBE/PW 0.4.

***Colouration***: Dorsally almost uniformly piceous (Fig. 48).

Head dark brown, slightly paler posteriorly, narrowly piceous behind eyes. Pronotum piceous, dark brown on sides, brown at anterior angles. Elytron piceous, slightly paler apically. Scutellum piceous. Antennae, other head appendages, and pro- and mesolegs reddish brown, metalegs darker, especially distally. Ventral side dark brown.

***Surface sculpture***: Elytron with 11+1 striae (Fig. 48).

Head without strioles, with rather dense punctation (spaces between punctures 1–3× size of punctures); punctures rather coarse (diameter of punctures usually equal to diameter of microreticulation cells); microreticulation distinct. Pronotum with several strioles laterally, with punctation finer than on head and microreticulation fine. Elytron with 11 dorsal striae, striae 1 and 10 usually shortly reduced basally, striae 1 additionally interrupted; submarginal stria present, long. Elytron with fine punctation and microreticulation.

***Structures***: Head relatively broad. Pronotum trapezoid, its lateral margins convergent anteriorly. Base of prosternum rounded anteriorly, convex medially; blade of prosternal process relatively narrow.

***Male***: Protibia straight, not modified. Proclaws long, rather straight, subequal in length, anterior claw thicker subapically than posterior one and due to this with a median incision on its inner margin. Median lobe of aedeagus with two lobes of dorsal sclerite relative narrow, subequal in length; apex of left dorsal lobe curved upwards, rounded; left dorsal lobe with lateral margin distinctly concave apically and with lateral side covered with large scale-like structures and at lateral margin medially additionally with small spinula-like structures; right dorsal lobe with broadly pointed apex, covered with large scale-like structures, with distinct median membranous area; lobes of ventral sclerite partly sclerotised, long, subequal in length, partly visible in left lateral view; left ventral lobe with elongate, strong sclerotised area, concave subapically, with apex pointed and slightly curved and membranous right part long, thin, apically covered with setae slightly visible in left lateral view; right ventral lobe with narrow sclerotised area on right margin. Parameres with dense, long setae occupying slightly more than half of dorsal margin, with proximal setae shorter (Fig. 98).

***Female***: Unknown.

##### Affinities.

The species belong to the medium-sized species with median lobe of the aedeagus that has scale- and spinula-like structures on the left dorsal lobe and among them, to the species with surface of left dorsal lobe completely covered by scale- and spinula-like structures in left lateral and ventral views. Based on body size and dark dorsal colouration, it is similar to *A.kalibumi* sp. nov., *A.herzogensis* sp. nov., *A.mianminensis* sp. nov., and *A.pseudomianminensis* sp. nov. but differs from them in almost uniformly piceous elytron colouration and median lobe of different shape and surface of left dorsal lobe completely covered by surface structures. See also under *A.fuscus* sp. nov. and *A.wasiorensis* sp. nov.

##### Etymology.

The species is named after Sararti Village. The name is a noun in the nominative singular standing in apposition.

##### Distribution.

Indonesia: West Papua Province: Teluk Wondama Regency. The species is known only from the type locality (Fig. 108).

##### Habitat.

Unknown.

#### ﻿﻿39. Austrelatus sumokedi sp. nov.

Taxon classificationAnimaliaColeoptera Dytiscidae

ED2FF693-FF55-5FC1-A48A-28423CF0F146

https://zoobank.org/3E4E5A70-038C-4919-8FCE-64E15538F846

Figs 24 , 75 , 107

##### Type locality.

Indonesia: Papua Province: Mimika Regency,04°30.330'S, 136°46.53'E, 24 m a.s.l.

##### Type material.

***Holotype***: male “Indonesia: Papua, Kabupaten Mimika, 24m, 25–30.v.2017, S 04°30.330’”, “E 136°46.53’, B. Sumoked (Pap69-Bob07)” (KSP).

##### Description.

***Body size and form***: Beetle small, elongate (Fig. 24).

***Measurements*: *Holotype***: TL 4.85 mm, TL-H 4.4 mm, MW 2.2 mm, TL/MW 2.21; PL 0.7 mm, PW 1.9 mm, PL/PW 0.37; DBE 0.81 mm, DBE/PW 0.43.

***Colouration***: With yellowish red head, brownish pronotum with yellowish red sides and dark brown elytra, with broad yellowish red basal band and yellowish apically and laterally (Fig. 24).

Head yellowish red, narrowly darker behind eyes. Pronotum brown on disc, darker on anterior and posterior margins and gradually paler (to yellowish red) to sides. Elytron dark brown, with broad yellowish red basal band and broadly yellow apically and laterally in apical 1/2. Scutellum reddish. Antennae, other head appendages, and legs yellowish red, legs darker distally. Ventral side reddish brown, darker on metacoxal plates.

***Surface sculpture***: Elytron with 11 complete striae, submarginal stria present: 11+1 (Fig. 24).

Head without strioles, with distinct, dense punctation (spaces between punctures 1–3× size of punctures); punctures coarse (diameter of punctures larger than or equal to diameter of microreticulation cells); microreticulation distinct. Pronotum without strioles, with punctation slightly sparser and finer than on head and microreticulation fine. Elytron with 11 complete striae; submarginal stria present, long. Elytron with very fine, sparse punctation and fine microreticulation.

***Structures***: Head broad. Pronotum trapezoid, its lateral margins convergent anteriorly. Base of prosternum rounded anteriorly, convex medially; blade of prosternal process relatively broad.

***Male***: Protibia straight, not modified. Proclaws relatively short, slightly curved, subequal in length. Median lobe of aedeagus with two lobes of dorsal sclerite subequal in length, covered with scale-like structures; left dorsal lobe with lateral margin concave apically, with straight, thick, and rounded apex, and scale-like structures with tiny spinulae covering whole lateral surface; right dorsal lobe broad, tapering to broadly pointed apex; lobes of ventral sclerite subequal in length, visible apically in left lateral view; left ventral lobe with elongate, narrow sclerotised area, with its apex slightly curved; right ventral lobe mostly membranous. Parameres with rather spase and short setae especially proximally; setae occupying almost whole dorsal margin (Fig. 75).

***Female***: Unknown.

##### Affinities.

The species is similar to *A.iriatoi* sp. nov. in elongate habitus and dorsal colouration, but differs from it in smaller body size, simple, slightly curved male proclaws (straight in *A.iriatoi* sp. nov.), and in median lobe structure: thicker and more rounded left lobe apex of dorsal sclerite and smaller and differently organised spinula-like structures of left dorsal lobe.

##### Etymology.

The species is dedicated to the collector and our friend Bob Sumoked (Tomohom, Sulawesi). The species name is a noun in the genitive case.

##### Distribution.

Indonesia: Papua Province: Mimika Regency. The species is known only from the type locality (Fig. 107).

##### Habitat.

Unknown.

#### ﻿﻿40. Austrelatus wanangensis sp. nov.

Taxon classificationAnimaliaColeoptera Dytiscidae

5712D169-0155-5810-B0B5-2E8C1E6D945E

https://zoobank.org/4B2DE3F6-CF50-4BEA-B0AF-16CACE3B645B

Figs 26 , 77 , 107

##### Type locality.

Papua New Guinea: Madang Province, Wanang Village, Wanang River.

##### Type material.

***Holotype***: male “PNG: Madang, Wanang Vill., Wanang Riv., 25.IX.13, #44 leg., D. Boukal” [hw, H. Shaverdo], “6442” [green text label] (ZSM). ***Paratypes*: *PNG*: *Sandaun***: 2 females “Papua New Guinea: Sandaun, Toricelli Mts., 2h walk fr Sibilanga Stn, 350m, 19.iv.2006, 03.39.121S 142.29.991E, Balke (PNG 44), one with an additional green label “3234” (ZSM).

##### Description.

***Body size and form***: Beetle small, elongate (Fig. 26).

***Measurements*: *Holotype***: TL 5.3 mm, TL-H 4.9 mm, MW 2.35 mm, TL/MW 2.25; PL 0.85 mm, PW 2.15 mm, PL/PW 0.4; DBE 0.9 mm, DBE/PW 0.42.

***Colouration***: Dorsally brown, with yellowish red head, pronotal sides and broadly on elytral base, sides and apex (Fig. 26).

Head yellowish red, narrowly darker behind eyes. Pronotum brown on disc and gradually paler (to yellowish red) to sides. Elytron brown, with broad yellowish red basal band, conjoined with yellowish red lateral side and apex, so that only elytral disc brown. Scutellum yellowish red. Antennae, other head appendages, and pro- and mesolegs yellowish red, metalegs darker, all legs darker distally. Ventral side brown.

***Surface sculpture***: Elytron with 11 complete striae, submarginal stria present: 11+1 (Fig. 26).

Head without strioles, with dense punctation (spaces between punctures 1–3× size of punctures); punctures coarse (diameter of punctures larger than or equal to diameter of microreticulation cells); microreticulation distinct. Pronotum with few strioles at middle of lateral sides, with punctation finer than on head and microreticulation fine. Elytron with 11 complete striae, stria 10 shortly reduced basally; a submarginal stria present, long. Elytron with fine, relatively dense punctation and fine microreticulation.

***Structures***: Head relatively broad. Pronotum trapezoid, relatively long, its lateral margins convergent anteriorly. Base of prosternum rounded anteriorly, convex medially; blade of prosternal process relatively narrow.

***Male***: Protibia straight, not modified. Proclaws relatively long, slightly curved, subequal in length, anterior claw with very slight median incision of its inner margin. Median lobe of aedeagus with two lobes of dorsal sclerite subequal in length, with broadly pointed apexes; left dorsal lobe with lateral margin slightly concave apically and lateral side slightly concave medially, covered laterally with numerous distinct spinula-like structures and dorsally with less numerous scale-like structures, lateral margin without surface structures, smooth; right dorsal lobe covered with scale-like structures; lobes of ventral sclerite partly sclerotised, long, partly visible in left lateral view; left ventral lobe with elongate, narrow sclerotised area, concave apically, with apex pointed and slightly curved; right ventral lobe longer than left lobe, with narrow sclerotised area on right margin, membranous part with long, thin, upwardly curved apex distinctly sticking out in left lateral view. Parameres with relatively dense and long setae occupying more than half of dorsal margin; proximally shorter and sparser, with several the most proximal setae standing separately (Fig. 77).

***Female***: As male but with more numerous pronotal strioles on sides and less developed yellow colouration of elytron, absent laterally.

##### Affinities.

The species is very similar to *A.iriatoi* sp. nov. in elongate habitus, dorsal colouration and striation and shape and surface structure of the median lobe, but differs from it in slightly curved male proclaws (straight in *A.iriatoi* sp. nov.) and left dorsal lobe of the median lobe with shorter and less concave apex and its surface structures slightly differently situated.

##### Etymology.

The species is named after Wanang Village. The name is an adjective in the nominative singular.

##### Distribution.

Papua New Guinea: Sandaun and Madang provinces (Fig. 107).

##### Habitat.

At the Sibilnaga Station, the species was collected in small and shallow forest pools rich in rotten leaves.

#### ﻿﻿41. Austrelatus wasiorensis sp. nov.

Taxon classificationAnimaliaColeoptera Dytiscidae

88A047A6-AAC0-5C7E-B34F-2F60F755C191

https://zoobank.org/D16D6B61-C218-4429-9C68-71E916EA1FD1

Figs 51 , 100 , 108

##### Type locality.

Indonesia: West Papua: Teluk Wondama Regency, Wasior, Sararti 100–200 m a.s.l.

##### Type material.

***Holotype***: male “Irian Jaya: Wandammen Bay, Wasior, Sararti 100–200 m, 7.-9.I.2001, leg A. Riedel” (SMNS). ***Paratypes***: 2 males, 1 female “Irian Jaya, Wandammen Bay, Wasior, KM 38, Sararti, 100–200 m, 7.-9.I.2001, leg A. Riedel” (NHMW, SMNS). 1 female “Irian Jaya, Wandammen Bay, Wasior, blok, 200 m, 8.I.2001, leg A. Riedel” (SMNS). 1 female “53” [green label], “Indonesia: West Papua, DMP, Wasior, 7.-10.i.2001, Riedel leg.” (ZSM).

##### Description.

***Body size and form***: Beetle medium-sized, oblong-oval (Fig. 51).

***Measurements***: TL 5.95–6.4 mm, TL-H 5.4–5.9 mm, MW 2.9–3.2 mm, TL/MW 1.99–2.05; PL 0.75–1 mm, PW 2.4–2.7 mm, PL/PW 0.31–0.37; DBE 0.95–1.05 mm, DBE/PW 0.39–0.41. ***Holotype***: TL 6.15 mm, TL-H 5.55 mm, MW 3.1 mm, TL/MW 1.99; PL 0.9 mm, PW 2.55 mm, PL/PW 0.35; DBE 1.05 mm, DBE/PW 0.41.

***Colouration***: Dorsally piceous, with reddish head, pronotal sides and elytral apex (Fig. 51).

Head reddish, slightly darker narrowly behind eyes. Pronotum piceous, with reddish sides. Elytron piceous, reddish apically, sometimes with small, yellow spot at apex, seldom with faint reddish basal spots medially. Scutellum brown to piceous. Antennae, other head appendages, and pro- and mesolegs reddish, metalegs slightly darker, especially distally. Ventral side brown, darker on posterior margins of abdominal ventrites 3–5 and on abdominal ventrite 6.

***Surface sculpture***: Elytron with 11+1 striae (Fig. 51).

Head without strioles, with rather dense punctation (spaces between punctures 1–3× size of punctures); punctures rather coarse (diameter of punctures usually equal to diameter of microreticulation cells); microreticulation distinct. Pronotum with numerous strioles mainly laterally, with few very tiny strioles on disc, with punctation finer than on head and microreticulation fine. Elytron with 11 dorsal striae, striae 10 and 11 shortly reduced basally; submarginal stria present, sometimes as apical strioles. Elytron with fine punctation and relatively distinct microreticulation.

***Structures***: Head relatively broad. Pronotum trapezoid, its lateral margins convergent anteriorly. Base of prosternum broadly rounded anteriorly, convex medially; blade of prosternal process relatively narrow.

***Male***: Protibia straight, not modified. Proclaws relatively long, simple, curved, anterior claw slightly longer than posterior. Median lobe of aedeagus with two lobes of dorsal sclerite with broadly pointed apexes; left dorsal lobe slightly longer than right dorsal lobe; lateral side of left dorsal lobe subapically with narrow, shallow concavity, covered with numerous large, short spinula-like structures and towards apex and dorsal side with scale-like structures; lateral margin slightly concave at apical tip; right dorsal lobe covered with large scale-like structures, with distinct median membranous area; lobes of ventral sclerite partly sclerotised, long, subequal in length, partly visible in left lateral view; left ventral lobe with elongate, strong sclerotised area, with apex elongate, rather straight and pointed and broad membranous part apically covered with setae not visible in left lateral view; right ventral lobe with relatively broad sclerotised area on right margin. Parameres with rather short and sparse setae occupying approximately half of dorsal margin (Fig. 100).

***Female***: As males but with stronger dorsal punctation and microreticulation and more numerous pronotal strioles.

##### Affinities.

The species belong to the medium-sized species with median lobe of the aedeagus that has scale- and spinula-like structures on the left dorsal lobe and among them, to the species with surface of left dorsal lobe completely covered by scale- and spinula-like structures in left lateral and ventral views. Based on body size and dark dorsal colouration, it is similar to *A.kalibumi* sp. nov., *A.herzogensis* sp. nov., *A.mianminensis* sp. nov., and *A.pseudomianminensis* sp. nov. but differs from them in left dorsal lobe of the median lobe with narrow, shallow, subapical concavity and surface of left dorsal lobe completely covered by surface structures. It is also similar to *A.fuscus* sp. nov. and *A.sararti* sp. nov. but differs from them in anterior proclaw simple, without median incision and left dorsal lobe of the median lobe with subapical concavity and large, but short spinula-like structures.

##### Etymology.

The species is named after Wasior District. The name is an adjective in the nominative singular.

##### Distribution.

Indonesia: West Papua Province: Teluk Wondama Regency (Fig. 108).

##### Habitat.

Unknown.

#### ﻿﻿42. Austrelatus wasurensis sp. nov.

Taxon classificationAnimaliaColeoptera Dytiscidae

140CB0AF-9B3C-5AB5-9FD8-292B64DFBABA

https://zoobank.org/84581F99-E0A5-4280-9212-62CD5A7153DC

Figs 54 , 103 , 108 , 116

##### Type locality.

Indonesia: Papua Province: Merauke Regency, Wasur, -07.6756°S, 140.4526°E, 20 m a.s.l.

##### Type material.

***Holotype***: male “Indonesia: Papua, Merauke, Wasur, pools, 20m, 15–16.x.2011, -7.6756° 140.4526°, UNCEN team (PAP02)” (KSP). ***Paratypes***: 17 males, 12 females with the same label as the holotype, one male and one female with additional labels with green text “5012” and “5013”, respectively (MZB, NHMW, ZSM).

##### Additional material.

1 female “7887” [green text], “Indonesia: Papua, Kabupaten Mimika, Timika, 149m, 25–30.v.2017,”, “-4.252020° 136.643384°, B.Sumoked (Pap68-Bob06)” (ZSM).

##### Description.

***Body size and form***: Beetle medium-sized, oblong-oval (Fig. 54).

***Measurements***: TL 5.4–6.3 mm, TL-H 4.9–5.8 mm, MW 2.6–3 mm, TL/MW 2.08–2.14; PL 0.8–0.9 mm, PW 2.2–2.6 mm, PL/PW 0.35–0.36; DBE 0.85–1 mm, DBE/PW 0.39–0.4. ***Holotype***: TL 6.2 mm, TL-H 5.5 mm, MW 2.9 mm, TL/MW 2.14; PL 0.9 mm, PW 2.5 mm, PL/PW 0.36; DBE 1 mm, DBE/PW 0.4.

***Colouration***: Piceous, with reddish head, pronotal anterior angles, and elytral basal band (Fig. 54).

Head almost completely reddish, narrowly piceous behind eyes and sometimes with obscure, V-shape, brownish spot medially. Pronotum piceous, reddish at anterior angles and sometimes on sides. Elytron piceous, with reddish basal band, sometimes rather distinct, sometimes obscure; elytral apex shortly yellow. Scutellum piceous. Antennae, other head appendages, and pro- and mesolegs yellowish brown, metalegs darker, especially distally. Ventral side piceous, with paler prosternum.

***Surface sculpture***: Elytron with 11 complete dorsal striae, submarginal stria present, long: 11+1 (Fig. 54).

Head without strioles, with relatively dense punctation (spaces between punctures 1–3× size of punctures); punctures relatively fine (diameter of punctures usually equal to or smaller than diameter of microreticulation cells); microreticulation distinct. Pronotum with several strioles at lateral margins, more numerous in females, with punctation finer than on head and microreticulation fine. Elytron with 11 complete, relatively strongly impressed dorsal striae; submarginal stria present, long, reaching more than half of elytral length. Elytron with fine punctation and microreticulation.

***Structures***: Head relatively broad. Pronotum trapezoid, its lateral margins convergent anteriorly. Base of prosternum narrowly rounded anteriorly, strongly convex medially; blade of prosternal process relatively narrow.

***Male***: Protibia straight, not modified. Proclaws relatively long; anterior claw shorter, thicker and more curved than posterior one and due to this with a median incision on its inner margin. Median lobe of aedeagus with two lobes of dorsal sclerite with broadly pointed apexes, more or less straight; left dorsal lobe slightly longer than right dorsal lobe, with not beaded, slightly concave at its apical 1/3 lateral margin; lateral side of left dorsal lobe completely covered with numerous large, single spinula-like structures; right dorsal lobe covered with large scale-like structures, with small, indistinct median membranous area; lobes of ventral sclerite sclerotised, long, broad, slightly visible in left lateral view; left ventral lobe with elongate, strong sclerotised area, with curved apex and broad slightly sclerotised part rounded apically; right ventral lobe distinctly longer than left one, slightly sclerotised, with long, thin apex. Parameres with dense, relatively long setae occupying distinctly more than half of dorsal margin; proximally shorter and sparser, several most proximal setae standing separately (Fig. 103).

***Female***: As males but with stronger dorsal punctation and microreticulation and more numerous pronotal strioles, in some specimens covering whole pronotum.

##### Affinities.

The species belong to the medium-sized species with median lobe of the aedeagus that has scale- and spinula-like structures on the left dorsal lobe and among them, to the species with surface of left dorsal lobe completely covered by scale- and spinula-like structures in left lateral and ventral views. Based on body shape and size and general shape of the median lobe, it is similar to *A.asteios* sp. nov., *A.epicharis* sp. nov., and *A.yeretuar* sp. nov. but differs from them in darker dorsal colouration, with usually indistinct, reddish basal band of the elytron and in median lobe with large, single spinulae on surface of its left dorsal lobe.

##### Etymology.

The species is named after Wasur. The name is an adjective in the nominative singular.

##### Distribution.

Indonesia: Papua Province: Merauke Regency (Fig. 108) but according to the additional material, it might have broader distribution in Papua.

##### Habitat.

At the type locality, the species was collected in shallow pools rich in rotten leaves and surrounded by wet savannah forest (Fig. 116). The species co-occurs with *Austrelatusclarkii* (Sharp, 1882) and numerous Bidessini species.

#### ﻿﻿43. Austrelatus weigeli sp. nov.

Taxon classificationAnimaliaColeoptera Dytiscidae

6B89F490-E320-518A-B768-6E4CE9DB3009

https://zoobank.org/CC3AC79B-9373-4739-AF02-851A0CE9F193

Figs 41 , 90 , 108

##### Type locality.

Papua New Guinea: East New Britain Province, 30 km SW Kokopo, Arabam, 04°35.75'S, 152°06.84'E, 200 m a.s.l.

##### Type material.

***Holotype***: male “PNG: E New Britain Prov. 30km SW Kokopo, Arabam, 200m 04°35'75"S, 152°06'84"E 21.II-04.III.2000 leg. A. Weigel KL” (ZSM). ***Paratypes***: 1 male, 1 female with the same label as the holotype (NHMW, ZSM).

##### Description.

***Body size and form***: Beetle medium-sized, oblong-oval to elongate (Fig. 41).

***Measurements***: TL 6.4–6.6 mm, TL-H 5.7–5.95 mm, MW 2.99–3.1 mm, TL/MW 2.08–2.2; PL 0.95–1 mm, PW 2.5–2.6 mm, PL/PW 0.37–0.39; DBE 1.1–1.2 mm, DBE/PW 0.44–0.46. ***Holotype***: TL 6.45 mm, TL-H 5.8 mm, MW 3.1 mm, TL/MW 2.08; PL 0.95 mm, PW 2.6 mm, PL/PW 0.37; DBE 1.15 mm, DBE/PW 0.44.

***Colouration***: With almost completely yellowish red elytron, yellowish red head and pronotal sides (Fig. 41).

Head yellowish red, narrowly darker behind eyes. Pronotum piceous on disc and broadly yellowish red on sides. Elytron yellowish red due to strongly developed basal band and colouration between striae; seldom piceous, with broad yellowish red basal band and yellowish red colouration between striae apically. Scutellum brownish. Antennae, other head appendages, and pro- and mesolegs yellowish red, metalegs brown, all legs darker distally. Ventral side brown, paler on prosternum and metaventrite and darker on abdominal ventrites.

***Surface sculpture***: Elytron with 11 complete, strongly impressed dorsal striae, submarginal stria present: 11+1 (Fig. 41).

Head with few strioles between eyes, with relatively sparse punctation (spaces between punctures 1–5× size of punctures); punctures fine (diameter of punctures smaller than or equal to diameter of microreticulation cells); microreticulation distinct. Pronotum with numerous but sparse strioles absent in middle and anterolaterally, with punctation sparser than on head and microreticulation fine. Elytron with 11 complete strongly impressed dorsal striae, stria 10 shortly reduced basally, sometimes with few small strioles between striae; submarginal stria present. Elytron with fine, sparse punctation and fine microreticulation.

***Structures***: Head relatively large and broad. Pronotum trapezoid, its lateral margins convergent anteriorly. Base of prosternum narrowly rounded anteriorly, convex medially; blade of prosternal process broad.

***Male***: Protibia straight, not modified. Proclaws relatively long, subequal in length, anterior claw thicker apically and slightly more strongly curved downwards than posterior one and due to this with a subapical incision on its inner margin. Median lobe of aedeagus with two lobes of dorsal sclerite subequal in length; left dorsal lobe slightly longer than right one, with broadly pointed and curved upwards apex; its lateral side with strong, elongate concavity subapically under upper lateral margin and covered with numerous, small but distinct scale-like structures bearing small spinulae; lateral margin and apex covered with small scale-like structures without spinula, lateral concavity covered with rather large scale-like structures; right dorsal lobe with broad, straight, pointed apex and covered with rather large scale-like structures, without membranous area on lateral side; lobes of ventral sclerite partly sclerotised, long, subequal in length, partly visible in left lateral view; left ventral lobe with elongate, narrow sclerotised area, with apex pointed and slightly curved to right; right ventral lobe with narrow, rather weak sclerotised area on right margin. Parameres with dense, long setae occupying distinctly more than half of dorsal margin; on small area proximally shorter and sparser, several most proximal setae standing separately (Fig. 90).

***Female***: As male but darker: elytron piceous, with broad yellowish red basal band and yellowish red colouration between striae apically.

##### Affinities.

The species belong to the medium-sized species with median lobe of the aedeagus that has scale- and spinula-like structures on the left dorsal lobe. However, it can be distinguished from them by yellowish red colouration and 11+1 complete, strongly impressed striae of its elytron and elongate, subapical concavity on the lateral side of the left dorsal lobe.

##### Etymology.

The species is named after our colleague Andreas Weigel, a specialist for xylophagous beetles, who collected the type series. The specific epithet is a substantive in the genitive case.

##### Distribution.

Papua New Guinea: East New Britain Province. The species is known only from the type locality (Fig. 108).

##### Habitat.

The species was collected in small forest pools.

#### ﻿﻿44. Austrelatus yamurensis sp. nov.

Taxon classificationAnimaliaColeoptera Dytiscidae

01AAB9B3-360D-52EF-8239-6E6BCCF06215

https://zoobank.org/76F8B7F4-72DF-49E3-A656-53B732667A97

Figs 16 , 68 , 107

##### Type locality.

Indonesia: West Papua Province: Kaimana Regency, Fakfak District, Lake Yamur area, 50–100 m a.s.l.

##### Type material.

***Holotype***: male “IRIAN JAYA: Fak Fak dist. Lake Yamur area, IV.1998 ca. 50 – 100m, Waldtümpel” (NHMW). ***Paratypes***: 3 males with the same label as the holotype (MZB, NHMW, ZSM).

##### Description.

***Body size and form***: Beetle medium-sized, elongate (Fig. 16).

***Measurements***: TL 5.6–5.8 mm, TL-H 5–5.1 mm, MW 2.45–2.6 mm, TL/MW 2.23–2.29; PL 0.9 mm, PW 2.3 mm, PL/PW 0.39; DBE 0.95 mm, DBE/PW 0.41. ***Holotype***: TL 5.8 mm, TL-H 5.1 mm, MW 2.6 mm, TL/MW 2.23; PL 0.9 mm, PW 2.3 mm, PL/PW 0.39; DBE 0.95 mm, DBE/PW 0.41.

***Colouration***: Dorsally piceous, with yellowish red head and anterolateral pronotal angles, broad yellow basal band and large yellow apical spot on elytron (Fig. 16).

Head yellowish red, narrowly darker behind eyes. Pronotum piceous, with yellowish red anterior angles and narrowly on sides. Elytron piceous, with distinct, broad yellow basal band and large yellow apical spot. Scutellum yellowish red to piceous. Antennae, other head appendages, and pro- and mesolegs yellowish red, metalegs darker, all legs darker distally. Ventral side reddish brown. All specimens except for the holotype are teneral.

***Surface sculpture***: Elytron with six dorsal striae but looks like with 5 dorsal striae due to stria 6 strongly reduced and weakly impressed; submarginal stria absent: 6+0 (Fig. 16).

Head without strioles, with distinct, relatively sparse punctation (spaces between punctures 1–5× size of punctures); punctures relatively fine (diameter of punctures more or less equal to diameter of microreticulation cells); microreticulation distinct. Pronotum without strioles, with punctation finer than on head and microreticulation distinct. Elytron with six dorsal striae, but stria 6 strongly reduced and weakly impressed, present as short stria or separate strioles only in apical 1/2 near stria 5; dorsal striae weakly impressed; striae 1 and 5 absent in basal 1/2; submarginal stria absent. Elytron with fine punctation and microreticulation.

***Structures***: Head relatively broad. Pronotum trapezoid, its lateral margins slightly convergent anteriorly. Base of prosternum narrowly rounded anteriorly, distinctly convex medially; blade of prosternal process relatively narrow.

***Male***: Protibia straight, not modified. Proclaws relatively long, slightly curved, subequal in length; anterior claw slightly thicker subapically than posterior one, with weak median incision of its inner margin. Median lobe of aedeagus with two lobes of dorsal sclerite covered with large, thick scale-like structures; left dorsal lobe slightly longer than right one, with apex broadly pointed and curved upwards, strong subapical crest, lateral margin concave apically, lateral groove, and with characteristic submedian knob-like modification; lobes of ventral sclerite partly sclerotised, subequal in length, long, partly visible in left lateral view; left ventral lobe with elongate, narrow sclerotised area, its apex pointed and curved; right ventral lobe more membranous, with apex pointed and slightly curved. Parameres with dense, relatively long setae occupying more than half of dorsal margin (Fig. 68).

***Female***: Unknown.

##### Variability.

There is an insignificant variation in the elytral striation.

##### Affinities.

In body size and shape and dorsal colouration, the species is similar to *A.kebarensis* sp. nov. but distinctly differs from it in its characteristic shape of the median lobe, with submedian knob-like modification of the left dorsal lobe, and only five complete dorsal elytral striae.

##### Etymology.

The species is named after its type locality, Lake Yamur area. The name is an adjective in the nominative singular.

##### Distribution.

Indonesia: West Papua Province: Kaimana Regency, Fakfak District. The species is known only from the type locality (Fig. 107).

##### Habitat.

The species was collected in a forest pool.

#### ﻿﻿45. Austrelatus yeretuar sp. nov.

Taxon classificationAnimaliaColeoptera Dytiscidae

32A66E2F-5AAD-5F20-A341-AA49720F5E81

https://zoobank.org/3C7AF79D-E9AB-48DC-A146-3097A3509C83

Figs 55 , 104 , 108

##### Type locality.

Indonesia: Papua Province: Nabire Regency, Yeretuar, 10 m a.s.l.

##### Type material.

***Holotype***: male “IRIAN JAYA: Kabup. Nabire Wandammen penins., Yeretua 10m, 17.-20.8.1998 (WA 18) (KSP). ***Paratypes***: 8 males, 8 females with the same label as the holotype (MZB, NHMW, ZSM).

##### Description.

***Body size and form***: Beetle small, elongate or oblong-oval (Fig. 55).

***Measurements***: TL 4.8–5.55 mm, TL-H 4.3–5 mm, MW 2.25–2.65 mm, TL/MW 2.09–2.21; PL 0.7–0.8 mm, PW 2–2.35 mm, PL/PW 0.34–0.38; DBE 0.85–0.95 mm, DBE/PW 0.4–0.44. ***Holotype***: TL 5.2 mm, TL-H 4.6 mm, MW 2.35 mm, TL/MW 2.21; PL 0.8 mm, PW 2.1 mm, PL/PW 0.38; DBE 0.9 mm, DBE/PW 0.43.

***Colouration***: With piceous elytra and pronotum and yellowish red head, pronotal sides, and elytral basal band (Fig. 55).

Head yellowish red medially, narrowly piceous behind eyes and brownish anteriorly. Pronotum piceous on disc and broadly yellowish red on sides, especially at anterior angles. Elytron piceous, with rather narrow yellowish red basal band, usually not reaching shoulders and suture; elytron sometimes with narrow yellow spot apically. Scutellum brownish to piceous. Antennae, other head appendages, and pro- and mesolegs yellowish red, metalegs slightly darker, especially distally. Ventral side brownish, with paler prosternum and darker posterior margins of ventrites.

***Surface sculpture***: Elytron with 11 complete dorsal striae, submarginal stria present, long: 11+1 (Fig. 55).

Head without strioles, with relatively dense punctation (spaces between punctures 1–3× size of punctures); punctures relatively fine (diameter of punctures usually equal to or smaller than diameter of microreticulation cells); microreticulation distinct. Pronotum with sparse strioles on lateral parts, only disc without strioles, with punctation finer than on head and microreticulation fine. Elytron with 11 complete dorsal striae, striae 1–3 less strongly impressed, especially anteriorly; submarginal stria present, long, often reaching more than ½ elytral length. Elytron with fine punctation and microreticulation.

***Structures***: Head relatively broad. Pronotum trapezoid, its lateral margins convergent anteriorly. Base of prosternum rounded anteriorly, strongly convex medially; blade of prosternal process narrow.

***Male***: Protibia straight, not modified. Proclaws relatively long, simple, subequal in length. Median lobe of aedeagus slender, apically thin, with two lobes of dorsal sclerite with broadly pointed apexes, straight, subequal in length; left dorsal lobe with lateral margin not beaded, slightly concave at its apical 1/2; lateral side of left dorsal lobe completely covered with tiny spinulae situated in groups on scale-like structures; right dorsal lobe covered with large scale-like structures, with distinct median membranous area; lobes of ventral sclerite partly sclerotised, long, slightly visible in left lateral view; left ventral lobe with elongate, strong sclerotised area, with curved apex and slightly sclerotised part distinctly shorter, with dense setae apically; right ventral lobe longer than left one, partly sclerotised, with apex curved left and covered with setae usually distinctly sticking out in left lateral view. Parameres with long and dense setae occupying approximately half of dorsal margin; more distally situated setae longer and denser than more proximal ones, with single most proximal setae standing separately (Fig. 104).

***Female***: As males.

##### Affinities.

Although the species is relatively small, it belongs to the medium-sized species with median lobe of the aedeagus that has scale- and spinula-like structures on the left dorsal lobe and among them, to the species with surface of left dorsal lobe completely covered by scale- and spinula-like structures in left lateral and ventral views. Based on body shape, dorsal colouration and general shape of the median lobe, it is similar and probably closely related to *A.asteios* sp. nov. and *A.epicharis* sp. nov. but differs from them in smaller body size and median lobe median lobe slender and its apex distinctly narrower in left lateral view. After first examination of the specimens of these three species, we thought to consider them as one species with two subspecies in Manokwary Regency (with sturdier median lobe) and Wandammen Peninsula (with slenderer median lobe). However, after further study, it was decided to treat them as separate species since there are other morphological differences. Material from the gap areas needs to confirm this decision.

##### Distribution.

Indonesia: Papua Province: Nabire Regency. The species is known only from the type locality (Fig. 108).

##### Habitat.

The species was collected in different forest pools and puddles, rich in rotten leaves.

#### ﻿﻿46. Austrelatus xanthocephalus

Taxon classificationAnimaliaColeoptera Dytiscidae

(Régimbart, 1899)

0AB9D462-14B4-505E-81F3-957B897107A9

**Figures 5–8.**
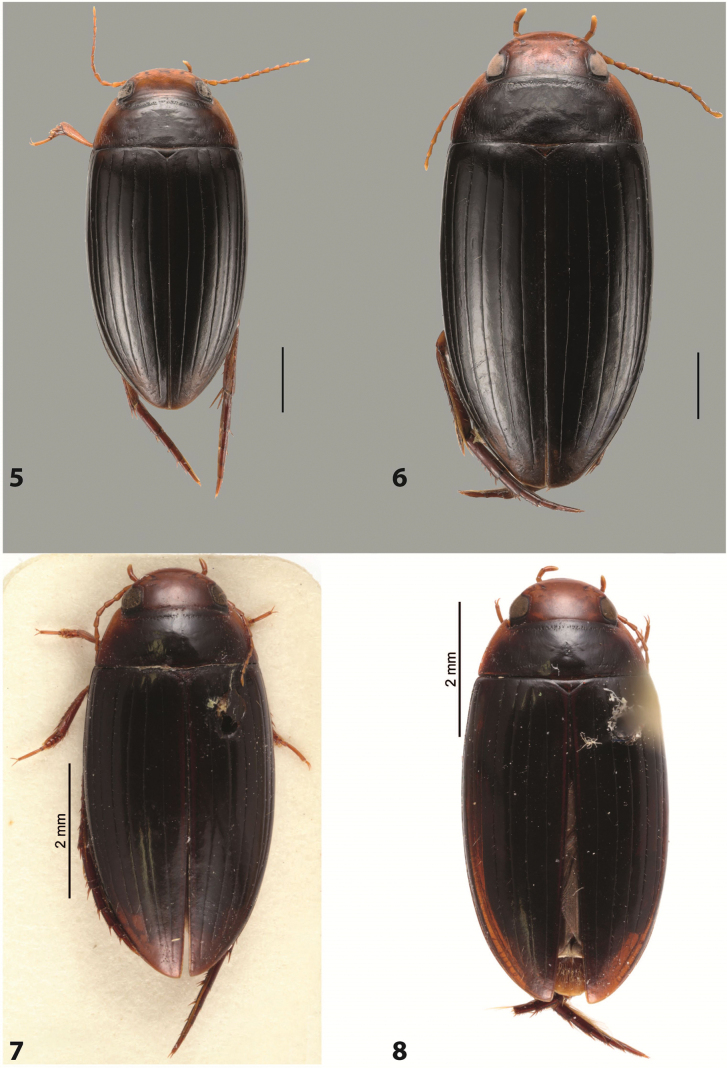
Habitus and colouration of **5***Austrelatusxanthocephalus* (Régimbart, 1899), Ransiki - Anggi (BH03) **6***A.xanthocephalus*, Testega (BH054) **7***A.xanthocephalus*, lectotype **8***A.xanthocephalus*, paralectotype. **7** and **8** photographs by Christophe Rivier (MNHN). Scale bars: 1 mm (**5, 6**).

Figs 5–8 , 61 , 62 , 107

Copelatus xanthocephalus Régimbart 1899: 293; Zimmermann (1919: 200, 1920: 144); Guignot (1956: 55); Guéorguiev (1968: 25); Guéorguiev and Rocchi (1993: 160); Nilsson and Hájek (2023: 50).
Austrelatus xanthocephalus (Régimbart, 1899): Shaverdo et al. (2023: 7).

##### Type locality.

According to Régimbart (1899: 293), “Nouvelle-Guinée (A. Raffray, coll. du Muséum de Paris)”. The types labels say “Nouvelle-Guinée (Amberbaki) A. Raffray 1878”. “Amberbaki” is most likely Amberbaken District (Indonesia: West Papua Province: Tambrauw Regency).

##### Type material.

***Lectotype***: male [small, red, square label], “2795 78” [round label, hw], “Nolle [illegible, probably short form from Nouvelle] Guinée” [hw], “MUSEUM PARIS COLL. MAURICE REGIMBART 1908”, “Muséum Paris Nouvelle-Guinée (Amberbaki) A. Raffray 1878”, “SYNTYPE” [red label], “SYNTYPE Copelatusxanthocephalus Régimbart, 1899”, “MNHN, Paris EC14220 [barcode]”, “Lectotype *Copelatusxanthocephalus* Régimbart, 1899 des. H. Shaverdo 2023” [red label] (MNHN) (Fig. 7). ***Paralectotype***: 1 female “2795 78” [round label, hw], “Nolle [illegible, probably short form from Nouvelle] Guinée Raffray” [hw], “MUSEUM PARIS COLL. MAURICE REGIMBART 1908”, “xanthocephalus Rég” [hw], “Muséum Paris Nouvelle-Guinée (Amberbaki) A. Raffray 1878”, “SYNTYPE” [red label], “SYNTYPE Copelatusxanthocephalus Régimbart, 1899”, “MNHN, Paris EC14219 [barcode]”, “Paralectotype *Copelatusxanthocephalus* Régimbart, 1899 des. H. Shaverdo 2023” [red label] (MNHN) (Fig. 8).

##### Additional material.

***IN*: *West Papua*: *Raja Ampat Regency*: *Waigeo***: 2 males “Indonesia: Papua, Waigeo, Waifoi, Mt.Nok, 500m, 11.ii.2006, 00.05.076S 130.44.586E, Tindige & Balke (BH 11)” (ZSM). 1 male, 1 female “Indonesia: Papua, Waigeo, Waifoi, < 50m, 10.ii.2006, 00 06.088S 130 42.855E, Tindige & Balke (BH 10)” (ZSM). 1 male “N.Dutch New Guinea Waigeu, Camp Nok. 2,500ft. vi.1938. L.E.Cheesman. B.M. 1938-593.” (BMNH). 9 male, 9 females “Irian Jaya: Sorong Prov. Waigeo Isl., Kabui Bay Wawiay, 14.-15.11.1996 0–250m, leg. A.Riedel” (NHMW, ZSM). 1 male, 1 female “Indonesia: Papua, Waigeo, Waifoi, Mt.Nok, 500m, 11.ii.2006,”, “00.05.076S 130.44.586E, Tindige & Balke (BH 11)”, “7594” and “7595” respectively [labels with green text] (ZSM). 6 males, 1 female “N.Dutch New Guinea Waigeu, Mt.Nok. Camp 2. (Buffelhorn.) vi.1938. L.E.Cheesman. B.M. 1938-593.” (BMNH, NHMW). ***Batanta***: 26 males, 22 females “Indonesia: Papua, Batanta Selatan, Wailebet, 100m, 17.ii.2006, inland 00.53.957S 130.39.951E, Tindige & Prativi (BH 15)”, one male and one female with additional green labels “4256” and “4254”, respectively (NHMW, ZSM). 5 males, 4 females “W-PAPUA Raja Ampat Pr. Yensawai Batanta, 9 km W Ross-River 0°49'23"S 130°35'52"E 17.I.2004 leg. A. Skale (CAS). 10 males, 3 females “Indonesia: Papua, Batanta Utara, 20m, 14.ii.2006, 00.50.125S 130.42.856E, Tindige & Balke, (BH 12)” (MZB, NHMW, ZSM). 5 male, 3 females “Indonesia: Papua, Batanta Selatan, Wailebet, 280m, 17.ii.2006, inland 00.53.957S 130.39.951E, Tindige & Prativi (BH 16)” (NHMW, ZSM). ***Salawati***: 6 male, 2 females “Indonesia: Papua, Salawatti Utara, 100–250m, 18.ii.2006, 00.57.954S 130.40.531E, Tindige & Balke (BH 18)” (NHMW, ZSM). ***Sorong Regency***: 1 female “Indonesia: Papua Barat, Sorong-Sausapor, 300m, 29.ix.2014, -0,7629653 131,6177023, B. Sumoked (BH041)” (ZSM). 4 males, 4 females “Indonesia: Papua Barat, Sausapor-Fef, 157m, 30.ix.2014, -0,6975004 132,072253, B. Sumoked (BH044)”, one male and two females additionally with green text labels “6484”, “6486”, and “6487”, respectively (ZSM). 6 males, 5 females “Indonesia: Papua Barat, Sorong-Teminabuan, 50m, 2.x.2014, -1,1092 131,6125, B. Sumoked (BH046)”, two males additionally with labels with green text “6448” and “6449” (ZSM). 3 males “Indonesia: West Papua, Malawor 50m 28.i.2001 Riedel leg.” (ZSM). 1 male “58” [green label], “Indonesia: Irian Jaya, Malawor, 28.i.2001, Riedel, MB 58” (ZSM). ***South Sorong Regency***: 3 males, 1 female “Indonesia: Papua Barat, Sorong-Teminabuan, 130m, 2.x.2014, -1,1357267 131,9000149, B.Sumoked (BH047)”, one male additionally with label with green text “6488” (ZSM). ***Manokwari Regency***: 2 males, 2 females “Indonesia: Papua Barat, Kebar to Manokwari, 1h from Kebar, limestone creek and roadside pools,”, “331m, 8.xi.2013, -0.80138488 133.32238254, UNIPA team (BH035)”, one male with an additional label with green text “6246” (ZSM). 1 male, 3 females “Indonesia: Papua Barat, Kebar Valley, 596m, 6.v.2015, -0,840623751282691 133,268257565796, UNIPA team (BH059)” (ZSM). 2 males “Indonesia: Papua Barat, Tamrau Mts N of Kebar, forest stream, 750m, 7.xi.2013, -0.7831 133.0721, UNIPA team (BH033)” (ZSM). 6 males, 10 females “Indonesia: Papua Barat, Kebar to Aibogar, forest stream, 644m, 4.xi.2013, -0,8533 132,8713 UNIPA Team (BH024)” (ZSM). 35 males, 23 females “Indonesia: Papua Barat, Kebar to Aibogar, slow forest stream, 503m, -0,8624 132,8299 UNIPA team (BH025)”, two males additionally with labels with green text “6219” and “6220” (NHMW, ZSM). 2 males, 3 females “Indonesia: Papua Barat, Fumato, forest stream, 820m, -0.90427148 132.71981431 UNIPA team (BH027)” (ZSM). 10 males, 2 females “IN: WP: Manokwari Reg., nr Testega Vill., ca. 1200 m, forest puddles, 03.V.2015, 01°22'11"S, 133°35'34"E UNIPA team (2015-WP25)” (NHMW). 1 male “IN: WP: Manokwari Reg., Testega, ca. 1000 m, forest streams, 1.V.2015, 01°22'54"S, 133°35'44"E, leg. Shaverdo (2015-WP18)” (NHMW). 1 female “IN: WP: Manokwari Reg., nr Testega Vill., ca. 1200 m, forest puddles, 03.V.2015, 01°22'15"S, 133°35'37"E UNIPA team (2015-WP23)” (NHMW). 1 female “IN: WP: Manokwari Reg., nr Testega Vill., ca. 1200 m, puddles in streambed, 03.V.2015, 01°22'11"S, 133°35'34"E UNIPA team (2015-WP24)” (NHMW). 22 males, 10 females “Indonesia: Papua Barat, Testega, 1212m, 3.v.2015, -1,36869 133,5908, UNIPA team (BH054)”, two males with additional labels with green text “7229” and “7228” (ZSM). 2 males, 1 female “IRIAN JAYA: Manokwari Iba 1300m 7.-8.4.1993 leg. A. Riedel” (NHMW). 3 females “Indonesia: Papua Barat, lowland Manokwari, 66m, 8.v.2015, -0,7433 133,3975, UNIPA team (BH065)” (ZSM). ***South Manokwari Regency***: 1 male, 3 females “Indonesia: Papua, Ransiki-Anggi, 1160m, 30.i.2006, 01.25.536S 134.02.456E, Tindige & Balke (BH 03)” (MZB, ZSM). ***Fakfak Regency***: 2 males, 2 females “West New Guinea / Fak-Fak / IR 27 Kali Mati, 4km N of Fak-Fak 260 m, 8. & 9.8.1991 leg. Balke & Hendrich” (CLH, ZSM). 1 male “Indonesia: Fak Fak, 800m, 23.ii.2008, Tindige, (FakFak)” (ZSM). 1 male “1323” [green label], “Indonesia: Irian Jaya Barat, Fak Fak, 310m, 23.ii.2006, Tindige” (ZSM). 1 male “Indonesia: Irian Jaya Barat, Fak Fak, 310m, 23.ii.2006, 2.53.756S 132.18.074E, Tindige, (FakFak)” (ZSM).

##### Description.

***Body size and form***: Beetle small to large, with habitus oblong-oval to more or less elongate (Figs 5–8).

***Measurements***: TL 4.7–7.1 mm, TL-H 4.3–6.5 mm, MW 2.3–3.3 mm, TL/MW 2.04–2.15; PL 0.78–1.1 mm, PW 2.05–3 mm, PL/PW 0.37–0.38; DBE 0.9–1.3 mm, DBE/PW 0.43–0.45. ***Lectotype*** (measured from photo): TL 6.11 mm, TL-H 5.5 mm, MW 2.84 mm, TL/MW 2.15; PL 0.9 mm, PW 2.42 mm, PL/PW 0.37; DBE 1.08 mm, DBE/PW 0.45.

***Colouration***: Dorsally piceous, with reddish head and pronotal sides (Figs 5–8).

Head yellowish red to dark red, narrowly piceous behind eyes. Pronotum dark brown to piceous on disc and gradually paler on sides, to yellowish red at anterior angles. Elytron almost always piceous, seldom with faint, small reddish spot(s) at shoulder and/or reddish along suture. Scutellum reddish to piceous. Antennae, other head appendages, and legs yellowish red to red, darker distally. Ventral side reddish brown to brown.

***Surface sculpture***: Elytron with 6+(0–1) striae, submarginal stria usually absent (Figs 5–8).

Head without strioles, with relatively dense punctation (spaces between punctures 1–3× size of punctures); punctures relatively coarse (diameter of punctures larger than or equal to diameter of microreticulation cells); microreticulation distinct. Pronotum with strioles (sometimes numerous) mainly at posterolateral angles, sometimes at posterior margin except for its middle, with punctation and microreticulation finer than on head. Elytron with a stable striation: six complete dorsal striae; submarginal stria usually absent, seldom present as a short stria or few short strioles apically. Elytron with punctation finer than on pronotum and fine microreticulation.

***Structures***: Head relatively broad. Pronotum trapezoid, its lateral margins distinctly convergent anteriorly. Base of prosternum rounded anteriorly, convex medially; blade of prosternal process relatively narrow.

***Male***: Protibia more or less straight, not modified; its ventral margin can be slightly curved proximally in larger specimens. Proclaws short, slightly curved, subequal in size and form. Median lobe of aedeagus with two lobes of dorsal sclerite subequal in length and shape, thin in apical 1/2, with slightly thickened, pointed apexes in lateral view, both densely covered with striola-like structures; usually left dorsal lobe slightly longer than right lobe, sometimes they of equal length, seldom left dorsal lobe slightly shorter than right lobe; apex of left dorsal lobe with hook-like tip, which placed into spoon-like tip of right lobe apex; lobes of ventral sclerite well-developed, mostly membranous, both with elongate sclerotised areas basally, distinctly shorter than dorsal lobes, broad, placed freely and well-visible ventrally, slightly visible laterally. Parameres with dense, long setae occupying distinctly more than half of dorsal margin; on small area distally longer and denser, proximal setae distinctly shorter and sparser (Figs 61, 62).

***Female***: As male, but with more numerous pronotal strioles.

##### Variability.

The species is very variable in body size. The smallest specimens are those from the islands (TL 4.7–5.5 mm). The medium-sized beetles are from Sorong (TL 5.1–5.8 mm), Kebar (TL 5.6–6.9 mm), Fumato (TL 5.8–6.1 mm), Manokwari (TL 5.9–6.2 mm), Anggi (TL 5.3–5.4 mm), and Fakfak (TL 5.8–6.5 mm). The largest specimens are from Testega (TL 6.6–7.1 mm). The variability in a relative length of lobes of dorsal sclerite of the median lobe is observed: most specimens have left dorsal lobe slightly longer than right lobe or equal to it (Figs 61C, 62E), only few specimens from Waigeo and Kedar have left dorsal lobe slightly shorter than right lobe, which is also characteristic for the lectotype (Fig. 62B, D).

##### Affinities.

The nominative subspecies differs from *A.xanthocephalusnabirensis* ssp. nov. the by its striated elytra, simple male protibia and usually smaller size. The species is similar to *A.miltokarenos* sp. nov. in body shape and colouration but differs from it in its smaller size, less striated elytra and completely different shape of the median lobe.

##### Distribution.

Indonesia: West Papua Province (Fig. 107).

##### Habitat.

The species was collected in many different water bodies: forest puddles and streams, a limestone creek and different roadside pools.

#### ﻿﻿46a. Austrelatus xanthocephalus nabirensis ssp. nov.

Taxon classificationAnimaliaColeoptera Dytiscidae

6E1D89D1-87A7-510D-9902-9BFB2DDE1705

https://zoobank.org/6E6BF65B-8692-427B-8067-63D2CE80F66F

**Figures 9–12.**
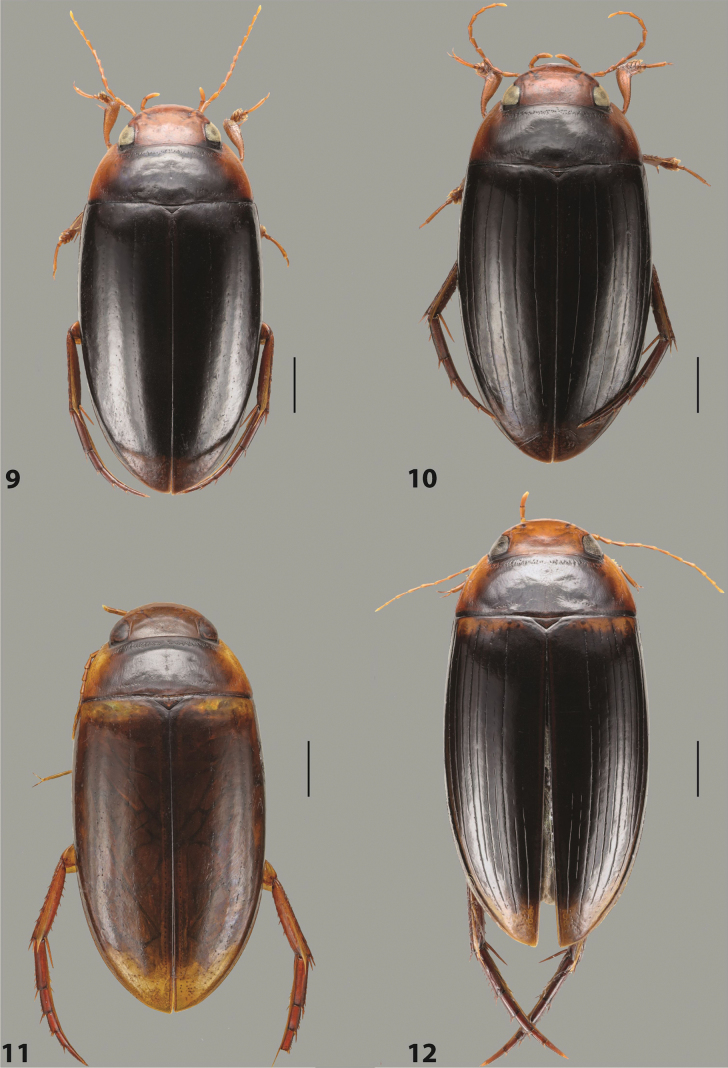
Habitus and colouration of **9***Austrelatusxanthocephalusnabirensis* ssp. nov., holotype, without elytral striae **10***A.x.nabirensis* ssp. nov., with elytral striae **11***A.bundunensis* sp. nov., without elytral striae **12***A.bundunensis* sp. nov., holotype, with elytral striae. Scale bars: 1 mm.

Figs 9 , 10 , 63 , 107 , 111 , 112 , 113

##### Type locality.

Indonesia: Papua Province: Nabire Regency, road Nabire-Enarotali, 54^th^ km, 03°29.51'S, 135°43.91'E, 750–800 m a.s.l.

##### Type material.

***Holotype***: male “West New Guinea/Paniai Prov./IR 19 track Nabire-Ilaga km 54 Basecamp, 750–800m, 16.-27.7.1991 leg: Balke & Hendrich” (ZSM). ***Paratypes***: 14 males, 12 females with the same label as the holotype (MZB, NHMW, ZSM). 11 males, 10 females “IRIAN JAYA: Paniai Prov. road Nabire – Ilaga, km 65, 29.8.1996, 250m (96 # 6)” (NHMW). 1 female “IRIAN JAYA: Nabire Prov., road Nabire – Ilaga, km 54, 26./27.8.1996, 750–800m (96 # 2)” (NHMW). 3 males “IRIAN JAYA: Nabire Prov., Nabire – Ilaga, km 54, 26.9.1997, 750m (# 4)” (NHMW). 1 male, 1 female “IRIAN JAYA: Nabire Prov., rd. Nabire - Ilaga, km 54, 03°29'51"S 135°43'91"E 750m, IV.1998”, “Restpfütze eines Baches” (NHMW). 4 males “Irian Jaya: Nabire distr., road Nabire-Ilaga, km 54, 03.29'517"S 135.43'913"E, 750m, iv.1998,” (ZSM). 1 female “IRIAN JAYA: Nabire Prov. rd. Nabire – Ilaga, Km 35 Kali Cemara, 100m, 27.9.1997 (IR97#6)” (NHMW). 1 male “IRIAN JAYA: Paniai Prov. road Nabire – Ilaga, Km 54, 30.8.1996, 750m (96 # 9)” (NHMW). 1 female “IRIAN JAYA: Paniai Prov. road Nabire – Ilaga, Km 54, 10.9.1996, 800m (96 # 20)” (NHMW). 5 males, 7 females “IRIAN JAYA: Paniai Prov. road Nabire – Ilaga, km 54, 10.9.1996, 900m (96 # 19)” (NHMW). 1 female “IRIAN JAYA: Paniai Prov. road Nabire – Ilaga, km 80, 1.9.1996, 200m (96 # 10)” (NHMW). 6 males “W.-Neuguinea/Paniai Prov. Straße Nabire-Ilaga km 54 700m, 22.-25.9.1990/IR 11 leg: Balke & Hendrich” (ZSM). 2 males, 2 females “IR90-11: W. New Guinea, Trek Nabire-Ilaga, km55, 19–25.ix.1990, Balke” (ZSM). 1 male “IR 19-W, New Guinea, Track Nabire-Ilaga KM 54, basecamp, 750–800m, 16.-27.vii.1991 Balke & Hendrich leg.” (ZSM). 4 males, 3 females “West New Guinea/Paniai Prov./IR 20 track Nabire-Ilaga KM 59, ca.750m, 18.7.1991 leg: Balke & Hendrich” (CLH, ZSM). 3 males, 1 female “West New Guinea/Paniai Prov./IR 24 track Nabire-Ilaga km 54 Basecamp, 750m, 25.7.1991 leg: Balke & Hendrich” (ZSM). 2 male, 2 females “IR #91-7 (IR 24). West New Guinea, Nabire-Ilaga km 54, basecamp 750m, 25. & 27.vii.1991, Balke” (ZSM). 1 male, 1 female “IR 23-W. New Guinea, track Nabire-Ilaga KM 62, 250m, 24.vii.1991 Balke & Hendrich leg.” (ZSM). 15 males, 5 females “West New Guinea/Paniai Prov./IR 22 track Nabire-Ilaga km 62 250m, 24.7.1991, forest pools leg: Balke & Hendrich” (CLH, ZSM). 6 males, 5 females “Indonesia: Papua, Road Nabire-Enarotali KM 55, 774m, 22.x.2011, 03.29.796S 135.43.885E, UNCEN team (PAP09)”, one male with an additional label with green text “5128” (ZSM). 4 males, 2 females “Indonesia: Papua, Road Nabire-Enarotali KM 52, 555m, 23.x.2011, 03.30.107S 135.42.971E, UNCEN team (PAP17)” (MZB, KSP, ZSM). 1 male “Indonesia: Papua, Road Nabire-Enarotali KM 60, 640m, 22.x.2011, 03.30.474S 135.42.611E, UNCEN team (PAP10)” (ZSM). 1 male “Indonesia: Papua, Road Nabire-Enarotali KM 62, 340m, 22.x.2011, 03.31.684S 135.42.802E, UNCEN team (PAP11)”, “5143” (ZSM).

##### Additional material.

1 male “IRIAN JAYA: Paniai Prov. road Nabire – Ilaga, km 54, 10.9.1996, 900m (96 # 19)” (NHMW).

##### Description.

***Body size and form***: Beetle medium-sized to large, with oblong-oval habitus (Figs 9, 10).

***Measurements***: TL 6.1–7.1 mm, TL-H 5.6–6.6 mm, MW 2.9–3.5 mm, TL/MW 2.03–2.1; PL 0.95–1.15 mm, PW 2.6–3.05 mm, PL/PW 0.36–0.38; DBE 1.1–1.3 mm, DBE/PW 0.41–0.43. ***Holotype***: TL 6.9 mm, TL-H 6.2 mm, MW 3.3 mm, TL/MW 2.09; PL 1.1 mm, PW 2.9 mm, PL/PW 0.38; DBE 1.2 mm, DBE/PW 0.41.

***Colouration***: As in nominative subspecies: dorsally piceous, with reddish head and pronotal sides (Figs 9, 10).

***Surface sculpture***: As in the nominative subspecies except for elytron usually without striae, with 3 rows of punctures or strioles, seldom with six dorsal striae; submarginal stria absent: (0–6)+0 (Figs 9, 10).

***Structures***: As in the nominative subspecies.

***Male***: Protibia modified: thinner proximally and broader medially and distally due to its curved ventral margin. Proclaws short, slightly curved, subequal in size and form. Median lobe of aedeagus and parameres as in the nominative subspecies except for left dorsal lobe never shorter than right lobe; it may be slightly longer than right lobe or of equal length (Fig. 63).

***Female***: Dimorphic: as male, but with more numerous pronotal strioles and sometimes elytral strioles between puncture lines or striae, and matt forms.

##### Variability.

There is an insignificant variation in the elytral striation: from all listed specimens, only seven have striated elytra. It is interesting that one male from the locality 96#19 (see “Additional material”) has shape of the median lobe different from those of the other males of the same locality: it is broader in ventral view and has very thin, forceps-like apexes of the dorsal sclerite lobes. The specimen also has striated elytra. It is most likely that this median lobe shape is a type of teratology or variation rather than this specimen being a new distinct species. However, more material from the area is necessary to clarify the matter.

##### Affinities.

The subspecies differs from the nominative subspecies by its elytra without striae, modified male protibia, and larger size.

##### Etymology.

The subspecies is named after Nabire Regency. The name is an adjective in the nominative singular.

##### Distribution.

Indonesia: Papua Province. The subspecies is known only from the area of the Nabire-Enarotali road (Fig. 107).

##### Habitat.

Most specimens around Nabire at km 54–62 of the Nabire-Ilaga track were collected in shallow (up to 20 cm water depth), shaded or at least partly shaded forest pools and puddles of different size, rich in rotten leaves and twigs (Figs 111–113). Few specimens were also found in water-filled track hollows on forest tracks and puddles remained in dried up streambeds.

### ﻿﻿Key to species of *Austrelatuspapuensis* group of New Guinea

The key is based mostly on male characters, since many species are rather similar in external morphology (for females, in internal morphology as well) and in most cases, the male genitalia (especially median lobe) need to be studied for reliable species identification. However, female identification is possible and for that, males and females from the same locality should not be separated for identification. If co-occurring species are not numerous (2–4 species), successful identifications of the females in association with the males of the same locality is highly possible.

A strong variability in the elytral striae is characteristic for the representatives of this group. Therefore, some species are placed twice or more in the key and their species dichotomies are composed so that they present all possible striae numbers. It is advisable also to use, if possible, several specimens of the same population for identification.

Numbers in square brackets placed before the species names refer to the species number above in the systematic account.

**Table:** 

1	Without elytral striae, with 2–4 rows of punctures or strioles	**2**
–	With elytral striae	**6**
2	Beetle small, TL 4.4–4.55 mm, elongate, with subparallel elytral sides (Figs 1, 2)	**3**
–	Beetle larger, TL > 6.5 mm, elongate-oval (Figs 5–15)	**4**
3	Beetle slender, with pronotal sides subparallel. Elytron without striae, with a narrower yellow basal band (Fig. 1). Male proclaws subequal in size and form. Male genitalia as in Fig. 59	[20] ***A.leptos* sp. nov.**
–	Beetle sturdier, with pronotal sides convergent anteriorly. Elytron with (0–6)+0 striae, with a broader yellow basal band (Fig. 2). Male anterior proclaw thicker subapically and more strongly curved downwards than posterior one and due to this with a median incision on its inner margin. Male genitalia as in Fig. 60	[34] ***A.procerus* sp. nov.**
4	Elytron uniformly dark brown to piceous, rarely with indistinct reddish brown basal spots. Elytron with (0–6)+0 striae: usually without striae, with 3 rows of punctures or strioles and seldom with 6 dorsal striae; submarginal stria absent. TL 6.1–7.1 mm (Figs 9, 10). Median lobe more slender, especially apically (Fig. 63)	[46a] ***A.xanthocephalusnabirensis* ssp. nov.**
–	Elytron with a distinct reddish yellow basal band. Elytral striation very variable: (0–10)+(0–1) striae. TL 6.7–8.2 mm. Median lobe robuster	**5**
5	Median lobe as in Fig. 64: left lobe of dorsal sclerite with a stronger median concavity, its apical 1/2 broader, apical crest larger. Male proclaws subequal in size and form. Elytron with narrower yellowish red basal band and yellowish red apex. TL 7.1–7.9 mm (Figs 11, 12)	[5] ***A.bundunensis* sp. nov.**
–	Median lobe as in Fig. 65: left lobe of dorsal sclerite with a weaker median concavity, its apical 1/2 narrower, apical crest smaller. Male anterior proclaw thicker subapically and more strongly curved downwards than posterior one and due to this with a median incision on its inner margin. Elytron with broader yellowish red basal band and sometimes additionally yellowish red laterally, apically and between striae. TL 6.7–8.2 mm (Figs 13–15)	[15] ***A.inconstans* sp. nov.**
6	Elytron with 6+(0–1) striae, sometimes with small strioles or reduced striae in-between	**7**
–	Elytron with (10–11)+(0–1) striae	**19**
7	Beetle smaller, TL ≤ 6 mm, elongate, almost parallel-sided. Elytron with a yellowish red basal band	**8**
–	Beetle larger, usually TL > 6 mm, elongate-oval. Elytron with or without a yellowish red basal band	**12**
8	Beetle smaller, TL ≤ 5 mm	**9**
–	Beetle larger, TL 5.1–5.8 mm	**11**
9	Elytron with (0–6)+0 striae; if present, striae weakly impressed, partly reduced. TL 4.5–4.55 mm (Fig. 2). Male genitalia as in Fig. 60	[34] ***A.procerus* sp. nov.**
–	Elytron with 6+(0–1) striae; striae more strongly impressed, complete or some of them shortly reduced basally	**10**
10	Elytron with 6+1 striae; stria 5 shortly reduced basally. TL 4.7 mm (Fig. 3). Male anterior proclaw thicker subapically and more strongly curved downwards than posterior one and due to this with a median incision on its inner margin. Median lobe as in Fig. 66: left lobe of dorsal sclerite broader, more strongly developed, with a subapical crest, which gives median lobe calla-like shape	[22] ***A.lopintolensis* sp. nov.**
–	Elytron with 6+(0–1) striae; sometimes striae 1, 3, 5 and 6 shortly reduced basally, stria 1 can be more strongly reduced. TL 4.35–5 mm (Fig. 4). Male proclaws very long, straight. Median lobe as in Fig. 67: left lobe of dorsal sclerite narrower, especially apically, without crest	[31] ***A.ohu* sp. nov.**
11	Elytron with 6+0 striae but looks like with 5 dorsal striae due to stria 6 strongly reduced, weakly impressed and slightly visible only apically; striae 1 and 5 reduced in basal 1/2; submarginal stria absent (Fig. 16). Median lobe as in Fig. 68: left lobe of dorsal sclerite with a distinct submedian knob-like modification	[44] ***A.yamurensis* sp. nov.**
–	Elytron with 6+1 striae: striae more strongly impressed, striae 1, 5, and 6 sometimes reduced basally, submarginal stria present (Fig. 17). Median lobe as in Fig. 69: left lobe of dorsal sclerite without such modification	[18] ***A.kebarensis* sp. nov.**
12	Elytron with a yellowish red basal band	**13**
–	Elytron uniformly piceous, without a distinct yellowish red basal band, seldom with reddish basal spots or reddish basally	**17**
13	Beetle smaller, TL 5.55–6.6 mm. Elytron with 6+(0–1) striae: striae more weakly impressed, striae 1, 5 and 6 reduced basally (Figs 18, 19). Median lobe as in Fig. 70: left lobe of dorsal sclerite not concave medially	[23] ***A.luteomaculatus***
–	Beetle usually larger, TL ≥ 6.3 mm. Elytron with 6+(0–1) striae: striae more strongly impressed, stria 1 reduced in basal 1/2, sometimes with additional strioles or striae in-between. Median lobe with left lobe of dorsal sclerite concave medially	**14**
14	Median lobe as in Fig. 71: apex of left dorsal lobe narrow, pointed, without crest. Elytron with (6–9)+1 striae: submarginal stria sometimes present as traces. TL 7–8 mm (Fig. 20)	[6] ***A.centralensis* sp. nov.**
–	Median lobe: apex of left dorsal lobe broader, with crest	**15**
15	Median lobe as in Fig. 65: left lobe of dorsal sclerite with a weak median concavity and very small apical crest. Elytral striation very variable: (0–10)+(0–1) striae. Elytron sometimes yellowish red laterally, apically and between striae additionally to yellowish red basal band. TL 6.7–8.2 mm (Figs 13–15)	[15] ***A.inconstans* sp. nov.**
–	Median lobe: left lobe of dorsal sclerite with a strong median concavity and larger apical crest. Elytron with yellowish red basal band and yellowish apically	**16**
16	Beetle larger, TL 7.1–7.9 mm. Elytron with (0–10)+(0–1) striae and narrower yellowish red basal band (Figs 11, 12). Median lobe as in Fig. 64: apex of left dorsal lobe broad, rather straight	[5] ***A.bundunensis* sp. nov.**
–	Beetle smaller, TL 6.5–7 mm. Elytron with 6+1 striae and broad yellowish red basal band (Fig. 21). Median lobe as in Fig. 72: apex of left dorsal lobe narrower, distinctly bent downwards	[30] ***A.normanbyensis* sp. nov.**
17	Beetle larger, TL 7.3–8.2 mm. Elytron with (6–11)+1 striae (Fig. 22). Median lobe as in Fig. 73: left lobe of dorsal sclerite concave medially	[28] ***A.miltokarenos* sp. nov.**
–	Beetle smaller, TL 4.7–7.1 mm. Elytron with (0–6)+(0–1) striae. Median lobe as in Figs 61–63: left lobe of dorsal sclerite not concave medially	**18**
18	Elytron with 6+(0–1) striae. Beetle usually smaller, TL 4.7–7.1 mm (Figs 5–8). Protibia simple or seldom slightly modified. Median lobe as in Figs 61, 62: usually left dorsal lobe longer than right one, seldom other way around	[46] ***A.xanthocephalus***
–	Elytron almost always without striae, very seldom with 6+0 striae. Beetle usually larger, TL 6.1–7.1 mm (Figs 9, 10). Protibia modified: thinner proximally and broader medially and distally due to its curved ventral margin. Median lobe as in Fig. 63: dorsal lobes equal or left lobe slightly longer than right one	[46a] ***A.xanthocephalusnabirensis* ssp. nov.**
19	Beetle smaller, TL < 5.5 mm, elongate	**20**
–	Beetle larger, TL > 5.5 mm, mostly elongate-oval	**23**
20	Elytron dark brown, paler basally and apically, with faint yellowish red shoulder spot. Elytron with 11+1 striae. TL 5 mm (Fig. 23). Median lobe as in Fig. 74: left lobe of dorsal sclerite apically thick, ventral sclerite not visible in left lateral view	[21] ***A.loloki* sp. nov.**
–	Elytron with a distinct yellowish red basal band. Median lobe: left lobe of dorsal sclerite apically thin, ventral sclerite visible in left lateral view	**21**
21	Beetle smaller, TL 4.9 mm (Fig. 24). Male genitalia as in Fig. 75: median lobe narrower in ventral view, its left dorsal lobe with thicker and more rounded apex and surface completely covered with scale-like structures with tiny spinulae in left lateral view	[39] ***A.sumokedi* sp. nov.**
–	Beetle larger, TL 5–5.5 mm (Figs 25, 26). Male genitalia: median lobe broader in ventral view, its left dorsal lobe with narrower apex and surface covered with larger spinula-like structures, absent on smooth upper lateral margin in left lateral view	**22**
22	Male proclaws very long, straight. Median lobe as in Fig. 76: apex of left dorsal lobe longer and more concave in left lateral view	[16] ***A.iriatoi* sp. nov.**
–	Male proclaws distinctly shorter, slender, slightly curved. Median lobe as in Fig. 77: apex of left dorsal lobe shorter and less concave in left lateral view	[40] ***A.wanangensis* sp. nov.**
23	Beetle with habitus elongate, slightly discontinuous between pronotum and elytra; pronotum large and long, with slightly rounded sides; TL 5.9 mm (Fig. 27). Male genitalia as in Fig. 78: median lobe robust, its left dorsal lobe thick, with a curved downwards apex and distinct apical crest	[8] ***A.decoris* sp. nov.**
–	Beetle elongate-oval; pronotum usually shorter, distinctly continuous with elytra. If median lobe similar, then beetle larger, TL > 6.3 mm; or, if beetle smaller, median lobe more slender, its left dorsal lobe thin, with evenly concave lateral margin, apex pointed or slightly rounded	**24**
24	Beetle larger, TL > 6.5 mm, except for *A.craterensis* (TL 6.4 mm). Median lobe sturdier, its left dorsal lobe thick, often with a curved downwards apex and small apical crest, laterally evenly tapering or differently concave; surface of left dorsal lobe often smoother due to scale-like structures	**25**
–	Beetle smaller, TL < 6.5 mm, seldom TL up to 6.7 mm. Median lobe more slender, its left dorsal lobe thin, with evenly concave lateral margin, with apex pointed or slightly rounded; surface of left dorsal lobe often more rough due to distinct predominant spinula-like structures	**38**
25	Beetle smaller, TL 6.4 mm (Fig. 28). Median lobe as in Fig. 79: left lobe of dorsal sclerite evenly tapering, its apex straight, with small but distinct incision and crest	[7] ***A.craterensis* sp. nov.**
–	Beetle larger, TL > 6.5 mm. Median lobe: left lobe of dorsal sclerite often with a curved downwards apex and small apical crest but without incision, laterally evenly tapering or differently concave	**26**
26	Median lobe as in Fig. 80: left lobe of dorsal sclerite distinctly concave apically; its apex narrow, with a long crest; ventral sclerite visible only in small apical part in left lateral view. TL 6.5–7.6 mm. Elytron with 11+0 striae: dorsal striae complete, always evidently 11. Elytron piceous, without a yellowish red basal band, yellowish apically (Fig. 29)	[1] ***A.aiyurensis* sp. nov.**
–	Median lobe: left lobe of dorsal sclerite with a distinct median concavity and its whole apical 1/2 narrowed or broadened, or without distinct median concavity and its whole apical 1/2 evenly tapering; apex of left dorsal lobe broad, with a small crest or without it; ventral sclerite not visible or visible in whole apical 1/2 in left lateral view	**27**
27	Median lobe: left lobe of dorsal sclerite with very strong median concavity, its apical 1/2 very broad. Elytron with 11+1 striae: stria 1 reduced basally, submarginal stria present. Elytron piceous, with narrow, sometimes short and faint yellowish red basal band	**28**
–	Median lobe: left lobe of dorsal sclerite with a distinct median concavity and its whole apical 1/2 narrowed or without distinct median concavity and its whole apical 1/2 evenly tapering. Elytron with (0–10)+(0–1) striae; with or without a yellowish red basal band	**29**
28	Male anterior proclaw thicker and more strongly curved downwards. Male protibia distinctly modified: thinner proximally and broader medially and distally due to its curved ventral margin. Median lobe as in Fig. 81: left lobe of dorsal sclerite with apical 1/2 narrower. TL 7.45–7.9 mm (Fig. 30)	[37] ***A.robustus* sp. nov.**
–	Male anterior proclaw narrower, straight. Male protibia slightly modified. Median lobe as in Fig. 82: left lobe of dorsal sclerite with apical 1/2 broader. TL 7.25–8.3 mm (Fig. 31)	[4] ***A.bosaviensis* sp. nov.**
29	Median lobe: left lobe of dorsal sclerite with a distinct median concavity and its whole apical 1/2 narrowed	**30**
–	Median lobe: left lobe of dorsal sclerite without distinct median concavity and its whole apical 1/2 more or less evenly tapering	**33**
30	Median lobe as in Fig. 83: left lobe of dorsal sclerite with apical 1/2 narrower and apex straight, without distinct crest, sometimes with a weak crest due to small, very shallow tip concavity, more or less evenly rounded. Elytron with (10–11)+(0–1) striae; with or without yellowish red basal band. TL 6.45–7.7 mm (Fig. 32)	[9] ***A.dekai* sp. nov.**
–	Median lobe: left lobe of dorsal sclerite with apical 1/2 broader and apex slightly curved downwards, with a small but distinct crest, slightly truncate or broadly pointed	**31**
31	Median lobe as in Fig. 65: left lobe of dorsal sclerite with a weaker median concavity, its apical 1/2 narrower, apical crest smaller. Elytral striation very variable: (0–10)+(0–1) striae. Elytron often yellowish red laterally, apically, and between striae additionally to yellowish red basal band. TL 6.7–8.2 mm (Figs 13–15)	[15] ***A.inconstans* sp. nov.**
–	Median lobe: left lobe of dorsal sclerite with a stronger median concavity, its apical 1/2 broader, apical crest larger	**32**
32	Elytron with (6–11)+1 striae, without yellowish red basal band. TL 7.3–8.2 mm (Fig. 22). Median lobe as in Fig. 73: left lobe of dorsal sclerite with apical 1/2 narrower and right dorsal lobe shorter	[28] ***A.miltokarenos* sp. nov.**
–	Elytron with (0–10)+(0–1) striae, with yellowish red basal band. TL 7.1–7.9 mm (Figs 11, 12). Median lobe as in Fig. 64: left lobe of dorsal sclerite with apical 1/2 broader and right dorsal lobe longer	[5] ***A.bundunensis* sp. nov.**
33	Median lobe: apex of left dorsal lobe elongate, almost straight, evenly or unevenly rounded or broadly pointed, with a small crest	**34**
–	Median lobe: apex of left dorsal lobe broader, slightly truncate and curved downwards, with more distinct crest	**35**
34	Elytron with 11+1 striae: dorsal striae complete, only stria 1 shortly reduced basally. TL 7–7.7 mm (Fig. 33). Male anterior proclaw thicker at its apex than posterior one and with slight subapical incision of its inner margin. Median lobe as in Fig. 84: apex of left dorsal lobe narrower, with weaker crest	[33] ***A.posmani* sp. nov.**
–	Elytron with (10–11)+1 striae: striae 1–3 reduced in basal 1/2, stria 1 strongly to completely reduced. TL 6.9–7.8 mm (Figs 34, 35). Male anterior proclaw thicker subapically and more strongly curved downwards than posterior one and due to this with a median incision on its inner margin. Median lobe as in Fig. 85: apex of left dorsal lobe broader, with more distinct crest	[32] ***A.papuensis***
35	Median lobe as in Fig. 86: left lobe of dorsal sclerite broad and flat, with broader and more rounded scale-like structures and small, short apex. TL 6.9–7.8 mm. Elytron with 11+1 striae: stria 1 shortly reduced basally (Fig. 36)	[3] ***A.bewaniensis* sp. nov.**
–	Median lobe: left lobe of dorsal sclerite distinctly narrower, with narrower scale-like structures and larger, more elongate apex	**36**
36	Male anterior proclaw thicker subapically and slightly more strongly curved downwards than posterior one and due to this with a median incision on its inner margin. Median lobe as in Fig. 87: left lobe of dorsal sclerite with shorter, more rounded apex. TL 7.9 mm (Fig. 37)	[29] ***A.noiadi* sp. nov.**
–	Male anterior proclaw thicker apically and more strongly curved downwards than posterior one, with subapical incision on its inner margin. Median lobe: left lobe of dorsal sclerite with longer apex	**37**
37	Median lobe as in Fig. 88: apex of left dorsal lobe shorter, with more vertical, broader crest. Elytron often yellowish red due to strongly developed, broad basal band and lateral and apical yellowish colouration; usually with 11+1 striae, seldom with a strong stria reduction: dorsal striae present only in apical part and some of them as strioles, stria 1 can be absent. TL 6.8–8.15 mm (Figs 38, 39)	[24] ***A.madangensis* sp. nov.**
–	Median lobe as in Fig. 89: apex of left dorsal lobe more elongate, with more transverse, narrower crest. Elytron darker, with narrow yellowish red basal band; with 11+1 striae: striae 1–3 reduced in basal 1/2, stria 1 can be reduced to apical strioles. TL 7.5–7.9 mm (Fig. 40)	[26] ***A.mamberamo* sp. nov.**
38	Median lobe: left lobe of dorsal sclerite with a distinct, deep surface concavity under upper lateral margin in left lateral view	**39**
–	Median lobe: left lobe of dorsal sclerite without such lateral concavity or with shallow one	**41**
39	Median lobe as in Fig. 90: left lobe of dorsal sclerite with subapical, elongate concavity. Elytron yellowish or with yellow basal band, with 11+1 complete, strongly impressed striae. TL 6.4–6.6 mm (Fig. 41)	[43] ***A.weigeli* sp. nov.**
–	Median lobe: left lobe of dorsal sclerite with concavity situated more medially, of triangular shape. Elytron with (10–11)+0 striae: stria 1 strongly reduced basally (visible in traces in apical 1/2), sometimes completely reduced, stria 2 or striae 2 and 3 usually reduced basally	**40**
40	Elytron without yellowish red basal band, piceous, yellowish red apically. TL 6.1–6.7 mm (Fig. 42). Median lobe as in Fig. 91, with its apical part distinctly longer	[17] ***A.kalibumi* sp. nov.**
–	Elytron with a broad yellowish red basal band, piceous, yellowish red apically. TL 5.65–6.7 mm (Fig. 43). Median lobe as in Fig. 92, with its apical part distinctly shorter	[14] ***A.herzogensis* sp. nov.**
41	Median lobe: surface of left dorsal lobe not completely covered by spinula-like structures in apical 1/2, upper lateral margin without spinulae, smooth, especially medially, in left lateral and ventral views	**42**
–	Median lobe: surface of left dorsal lobe completely covered by spinula-like structures in apical 1/2 including median part in left lateral and ventral views	**46**
42	Elytron without yellowish red basal band, with a yellow apical spot	**43**
–	Elytron with a yellowish red basal band	**44**
43	Beetle larger, TL 5.6–6.4 mm. Elytron with 11+1 striae but stria 1 usually weakly impressed or differently reduced (Fig. 44). Median lobe as in Fig. 93: lateral margin of left dorsal lobe with weak edge, below which spinula-like structures are situated	[27] ***A.mianminensis* sp. nov.**
–	Beetle smaller, TL 5.3–5.5 mm. Elytron with 11+1 complete, strongly impressed striae (Fig. 45). Median lobe as in Fig. 94: lateral margin of left dorsal lobe with more sharp and prominent edge, below which spinula-like structures are situated	[36] ***A.pseudomianminensis* sp. nov.**
44	Median lobe as in Fig. 95: left dorsal lobe covered with larger spinula-like structures, its apex slightly curved downwards. TL 5.4–6.1 mm, TL/MW 2.04–2.1 (Fig. 46)	[11] ***A.flavocapitatus* sp. nov.**
–	Median lobe: left dorsal lobe covered with smaller spinula-like structures, its apex more or less straight	**45**
45	Median lobe as in Fig. 96: lobes of dorsal sclerite broader, left dorsal lobe with a shallow surface concavity in left lateral view. Beetle more oval: TL 5.4–5.7 mm, TL/MW 2.04–2.11 (Fig. 47)	[25] ***A.maindai* sp. nov.**
–	Median lobe as in Fig. 97: lobes of dorsal sclerite narrower, left dorsal lobe without concavity in left lateral view. Beetle more elongate: TL 5.6–5.65 mm, TL/MW 2.11–2.17 (Fig. 49)	[19] ***A.kokodensis* sp. nov.**
46	Elytron without yellowish red basal band, piceous, sometimes yellowish red apically	**47**
–	Elytron with a yellowish red basal band	**49**
47	Beetle smaller, TL 5.7 mm. Elytron with 11+1 striae (Fig. 48). Median lobe as in Fig. 98: left lobe of dorsal sclerite without concavity, covered with small spinula-like structures	[38] ***A.sararti* sp. nov.**
–	Beetle larger, TL 5.95–6.4 mm. Median lobe: left lobe of dorsal sclerite with shallow concavity, covered with large spinula-like structures	**48**
48	Elytron with 10+1 striae: submarginal stria present as weak apical strioles (Fig. 50). Median lobe as in Fig. 99: left lobe of dorsal sclerite with a shallow, large, median concavity, covered with large, long spinula-like structures	[12] ***A.fuscus* sp. nov.**
–	Elytron with 11+1 striae (Fig. 51). Median lobe as in Fig. 100: left lobe of dorsal sclerite with a shallow, narrow, subapical concavity, covered with large, short spinula-like structures	[41] ***A.wasiorensis* sp. nov.**
49	Elytron with (10–11)+1 striae, striae 1–3 reduced basally, often stria 1 present only apically as strioles, sometimes completely reduced. Median lobe: lateral margin of left dorsal lobe beaded. Beetle usually larger, TL 5.8–6.9 mm	**50**
–	Elytron with 11+1 complete striae, seldom stria 1 reduced basally. Median lobe: lateral margin of left dorsal lobe simple, not beaded. Beetle usually smaller, TL 4.8–6.6 mm	**51**
50	Elytron with narrower yellowish red basal band. TL 5.8–6.8 mm (Fig. 52). Median lobe as in Fig. 101: left lobe of dorsal sclerite narrower and narrowed to apex in its apical 1/2	[13] ***A.gestroi***
–	Elytron broader with yellowish red basal band. TL 6.9 mm (Fig. 53). Median lobe as in Fig. 102: left lobe of dorsal sclerite broader and narrowed to apex in its apical 1/3	[35] ***A.pseudogestroi* sp. nov.**
51	Elytron piceous, usually with a short, median reddish basal band. TL 5.4–6.3 mm (Fig. 54). Median lobe as in Fig. 103: surface of left dorsal lobe with large, single spinulae	[42] ***A.wasurensis* sp. nov.**
–	Elytron usually with a distinct yellowish red basal band. TL 5.7–6.6 mm. Median lobe: surface of left dorsal lobe with tiny spinulae situated in groups on scale-like structures	**52**
52	Beetle smaller, TL 4.8–5.5 mm (Fig. 55). Male genitalia as in Fig. 104: median lobe slender, its apex distinctly narrower in left lateral view	[45] ***A.yeretuar* sp. nov.**
–	Beetle larger, usually TL > 5.5 mm. Male genitalia: median lobe sturdier, its apex broader in left lateral view	**53**
53	Beetle paler: elytron piceous, with a distinct, broad yellowish red basal band and yellowish between striae laterally, sometimes additionally apically, seldom whole elytron slightly yellowish. TL 5.7–6.6 mm (Fig. 57). Male genitalia as in Fig. 105: median lobe sturdier, left dorsal lobe with apex thicker and lateral margin more strongly concave apically and therefore ventral lobes more visible in left lateral view	[2] ***A.asteios* sp. nov.**
–	Beetle darker: elytron piceous, with a distinct yellowish red basal band and apical spot. TL 5.1–6.35 mm (Fig. 56). Male genitalia as in Fig. 106: median lobe slender, left dorsal lobe with apex thinner and lateral margin more straight apically and therefore ventral lobes less visible in left lateral view	[10] ***A.epicharis* sp. nov.**

**Figures 13–15.**
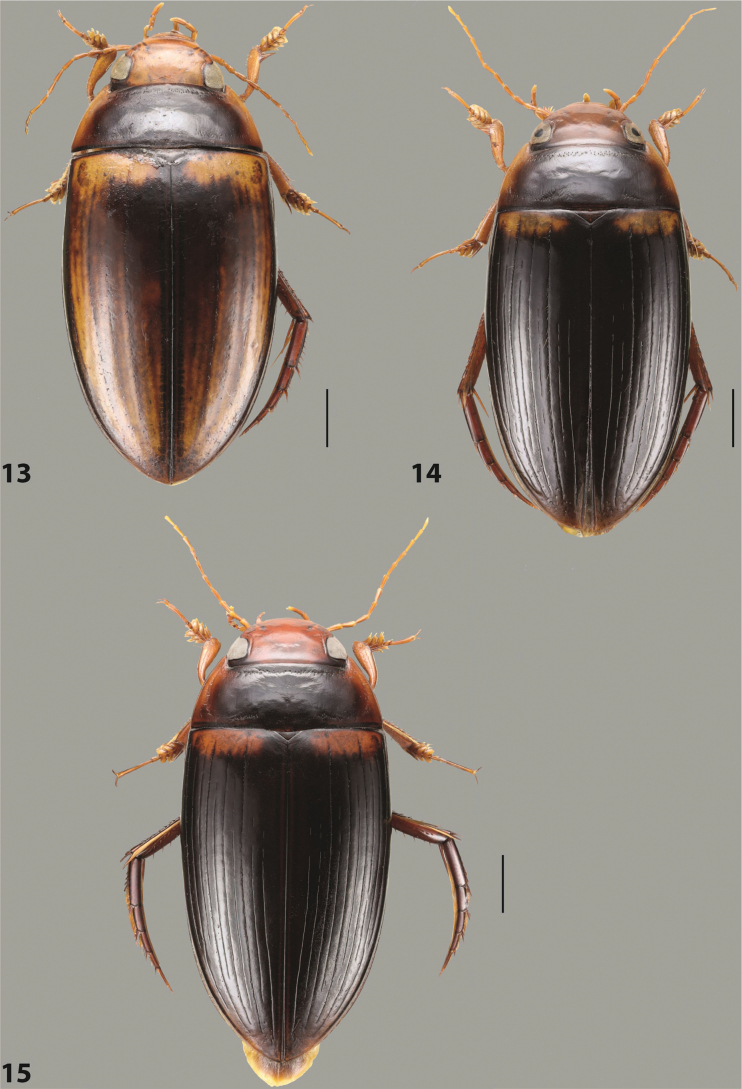
Habitus and colouration of **13***Austrelatusinconstans* sp. nov., without elytral striae **14** and **15***A.inconstans* sp. nov., holotype, with elytral striae. Scale bar: 1 mm.

**Figures 16–19.**
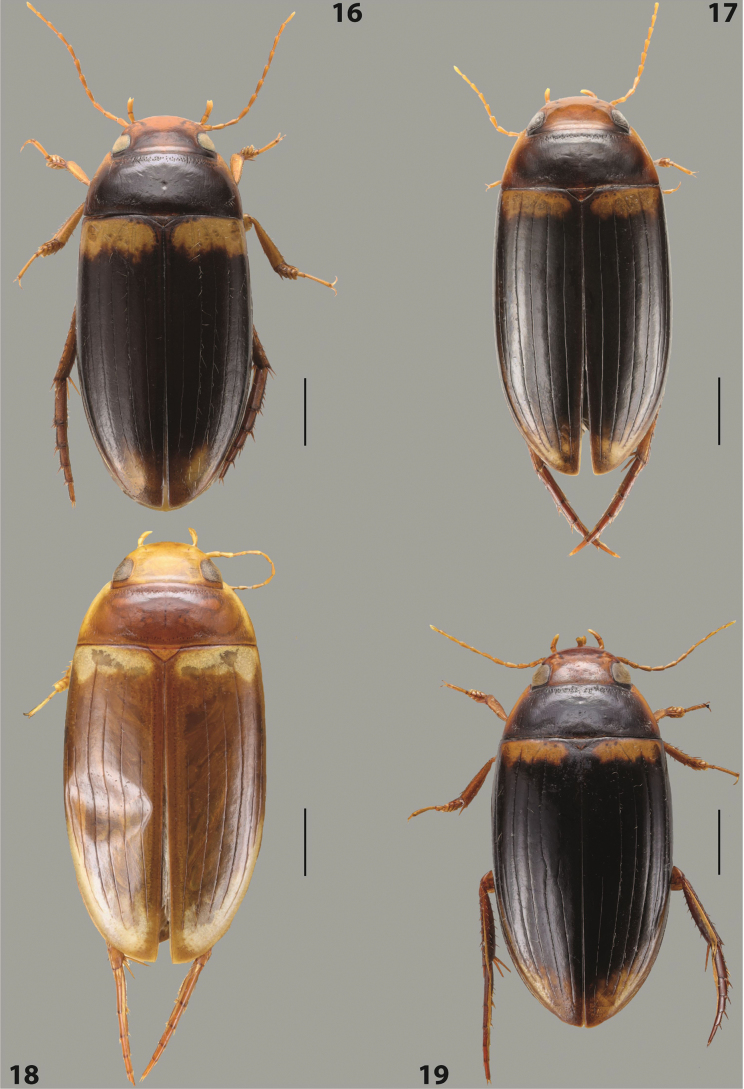
Habitus and colouration of **16***Austrelatusyamurensis* sp. nov., holotype **17***A.kebarensis* sp. nov., holotype **18***A.luteomaculatus* (Guignot, 1956), lectotype **19***A.luteomaculatus*. Scale bar: 1 mm.

**Figures 20–22.**
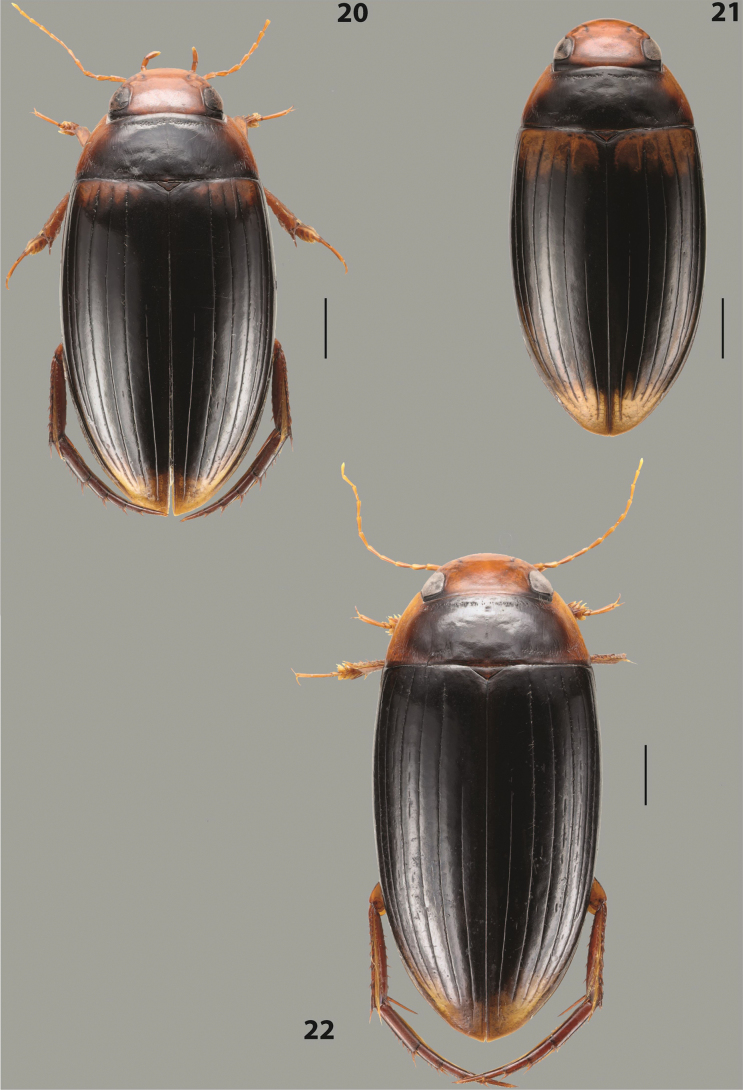
Habitus and colouration of **20***Austrelatuscentralensis* sp. nov., paratype **21***A.normanbyensis* sp. nov., holotype **22***A.miltokarenos* sp. nov., holotype. Scale bar: 1 mm.

**Figures 23–26.**
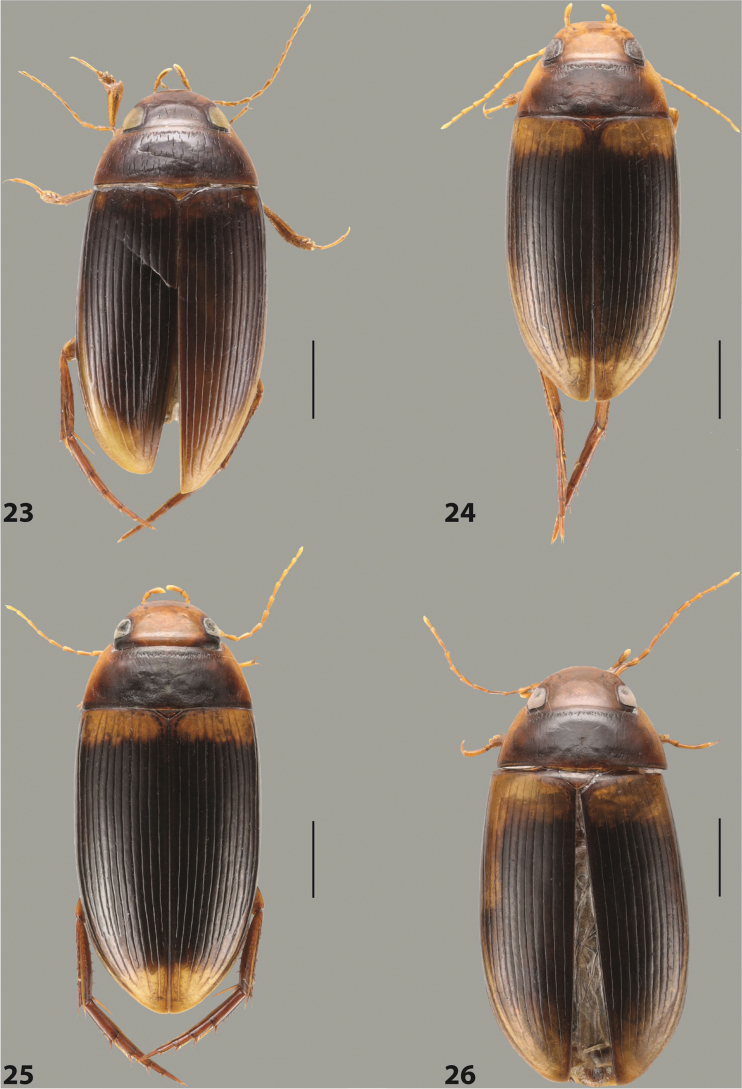
Habitus and colouration of the holotypes of **23***Austrelatusloloki* sp. nov. **24***A.sumokedi* sp. nov. **25***A.iriatoi* sp. nov. **26***A.wanangensis* sp. nov. Scale bar: 1 mm.

**Figures 27–30.**
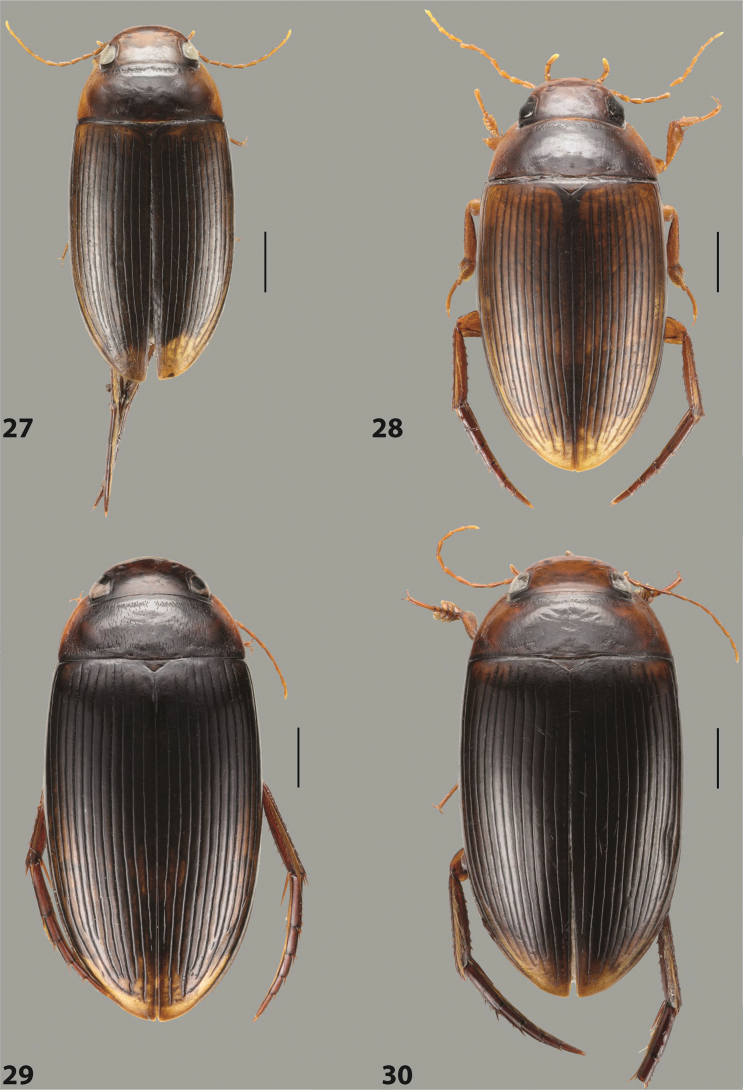
Habitus and colouration of the holotypes of **27***Austrelatusdecoris* sp. nov. **28***A.craterensis* sp. nov. **29***A.aiyurensis* sp. nov. **30***A.robustus* sp. nov. Scale bars: 1 mm.

**Figures 31–33.**
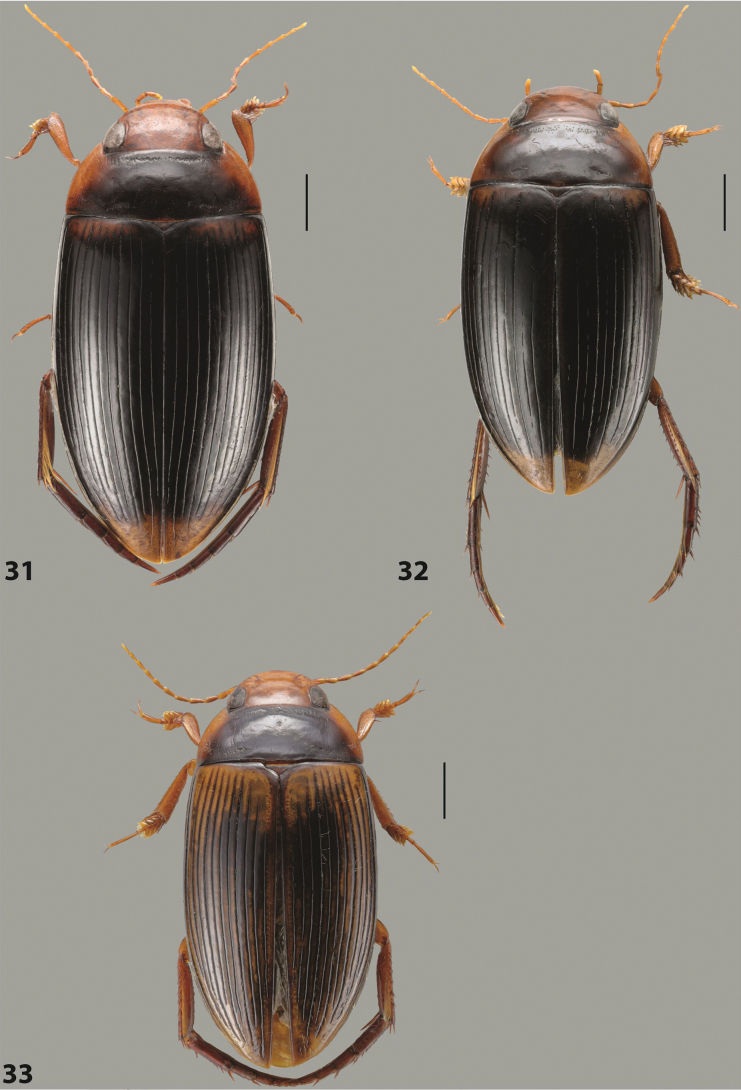
Habitus and colouration of the holotypes of **31***Austrelatusbosaviensis* sp. nov. **32***A.dekai* sp. nov. **33***A.posmani* sp. nov. Scale bars: 1 mm.

**Figures 34–37.**
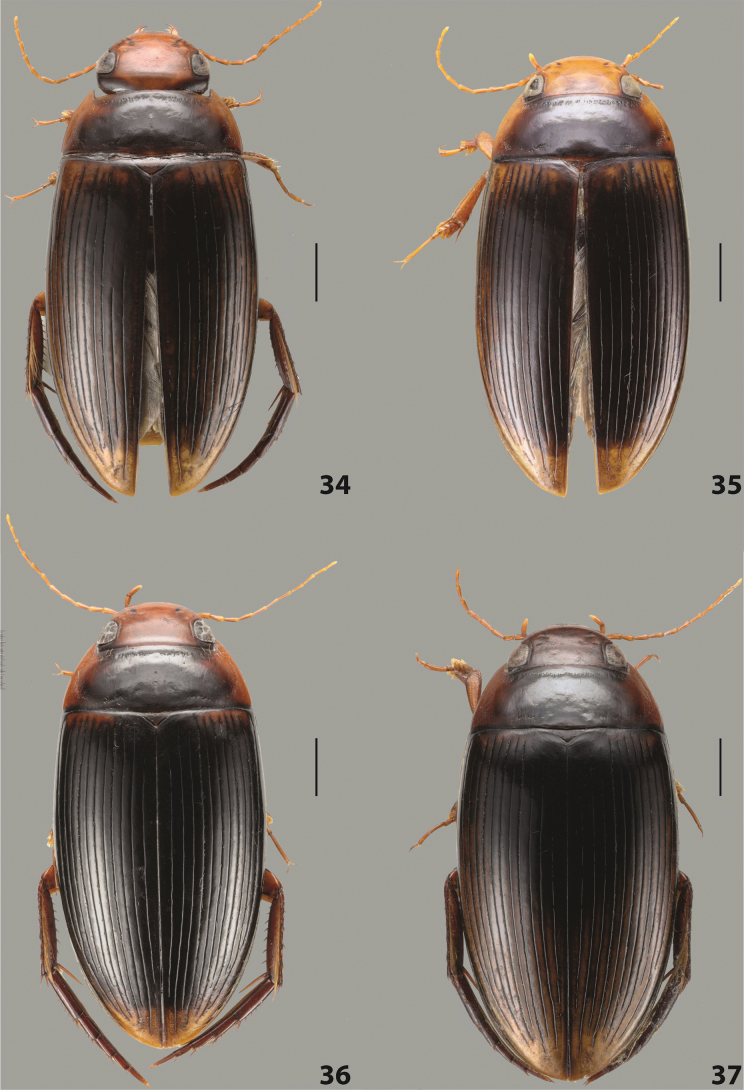
Habitus and colouration of **34***Austrelatuspapuensis* (J. Balfour-Browne, 1939) **35***A.papuensis*, holotype **36***A.bewaniensis* sp. nov., holotype **37***A.noiadi* sp. nov., holotype. Scale bars: 1 mm.

**Figures 38–41.**
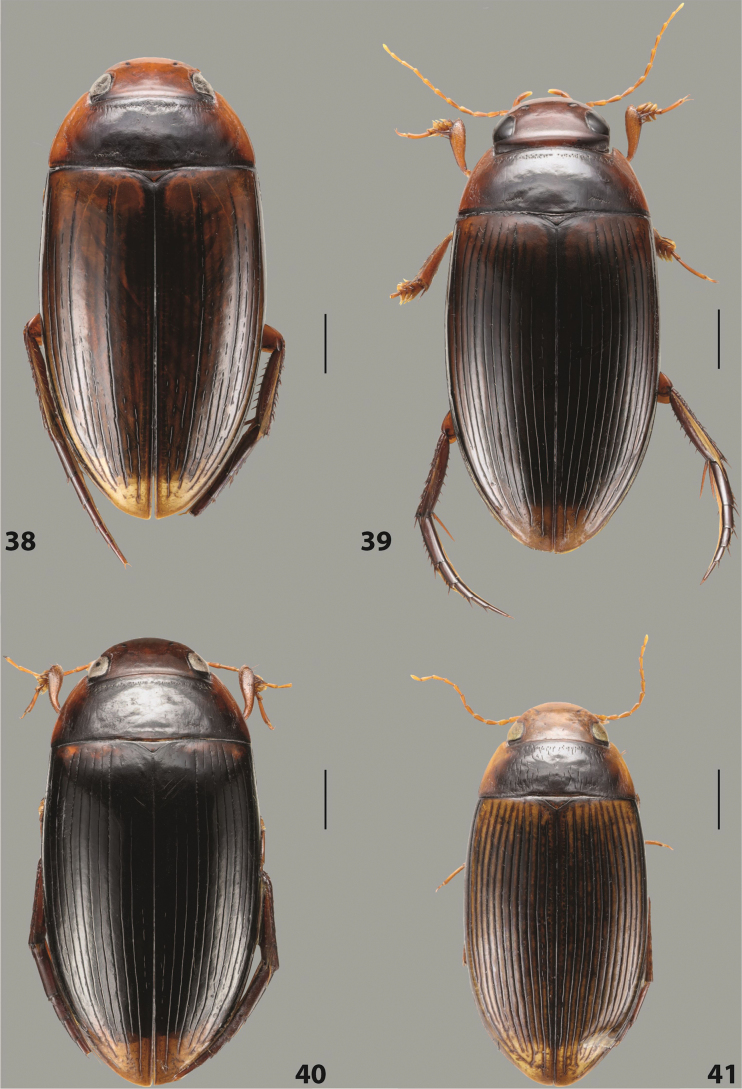
Habitus and colouration of **38***Austrelatusmadangensis* sp. nov. **39***A.madangensis* sp. nov., holotype **40***A.mamberamo* sp. nov., holotype **41***A.weigeli* sp. nov., holotype. Scale bars: 1 mm.

**Figures 42–45.**
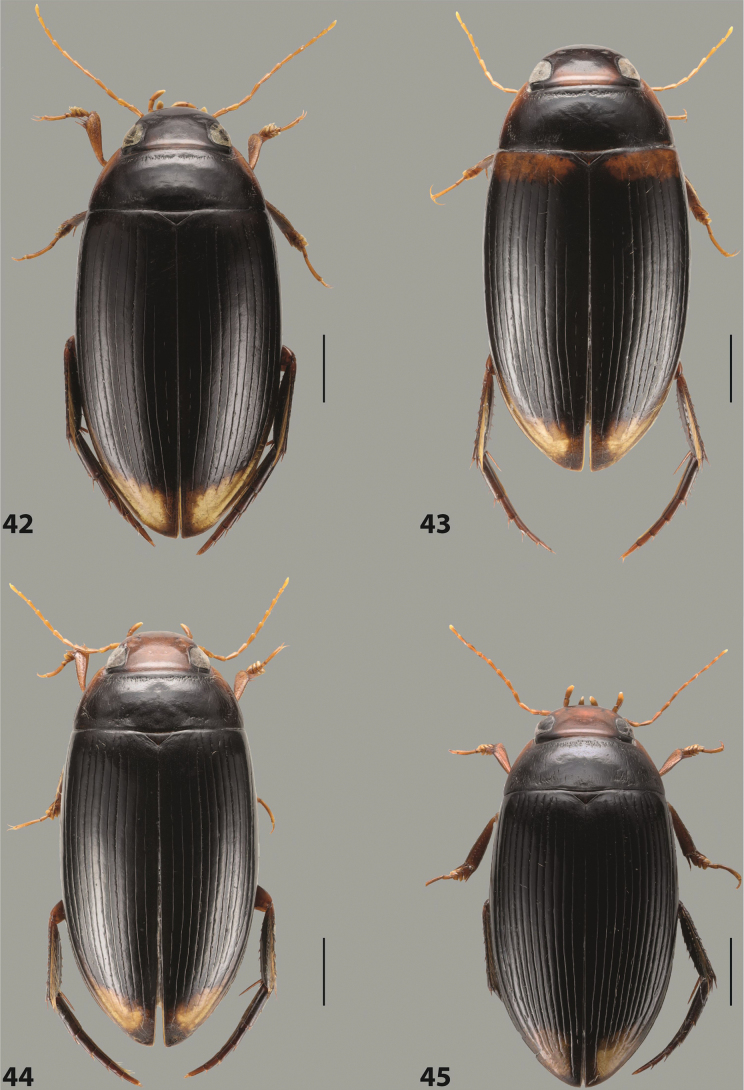
Habitus and colouration of the holotypes of **42***Austrelatuskalibumi* sp. nov. **43***A.herzogensis* sp. nov. **44***A.mianminensis* sp. nov. **45***A.pseudomianminensis* sp. nov. Scale bars: 1 mm.

**Figures 46–49.**
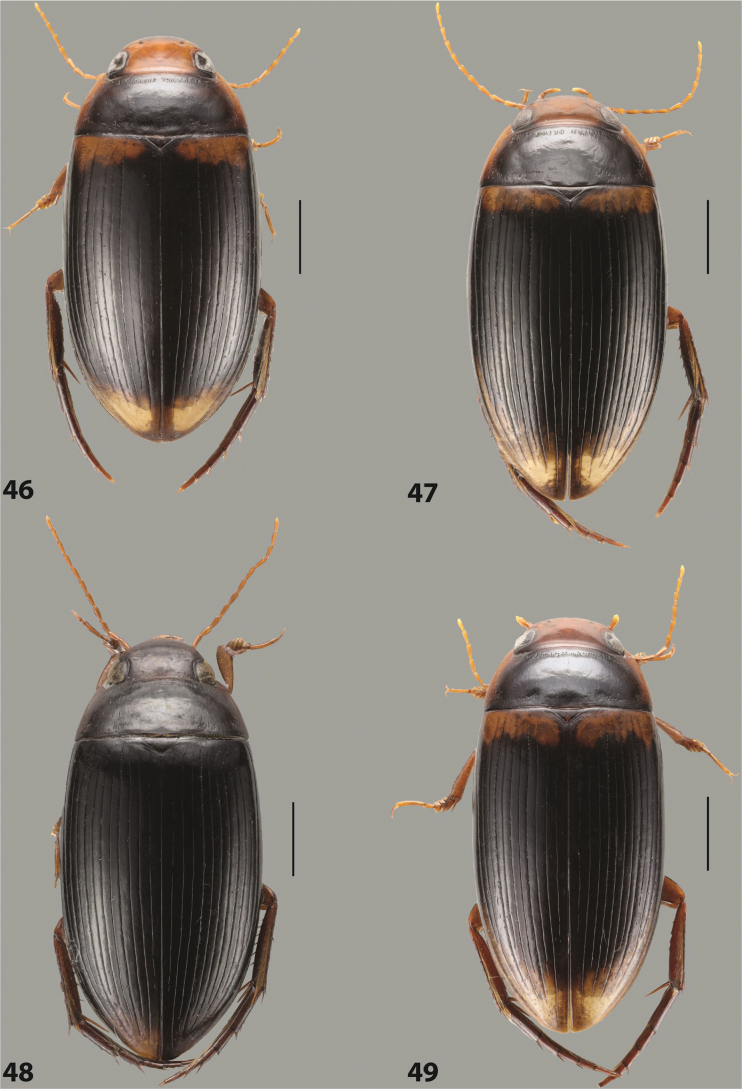
Habitus and colouration of the holotypes of **46***Austrelatusflavocapitatus* sp. nov. **47***A.maindai* sp. nov. **48***A.sararti* sp. nov. **49***A.kokodensis* sp. nov. Scale bars: 1 mm.

**Figures 50–53.**
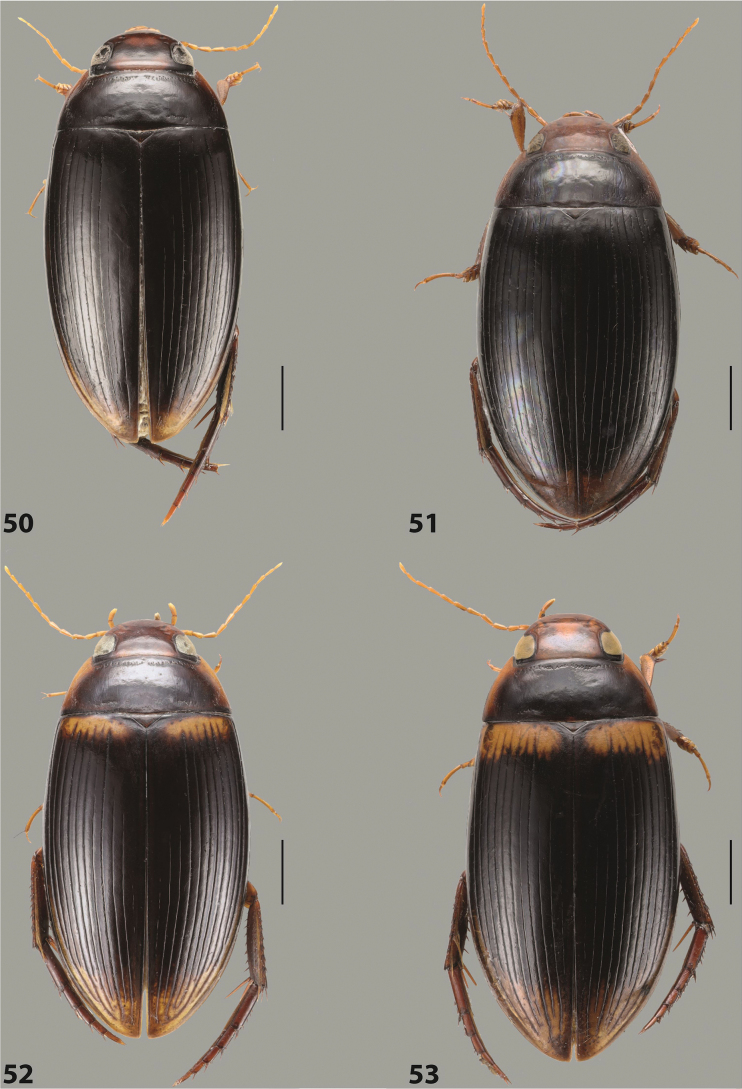
Habitus and colouration of **50***Austrelatusfuscus* sp. nov., holotype **51***A.wasiorensis* sp. nov., holotype **52***A.gestroi* (Régimbart, 1892) **53***A.pseudogestroi* sp. nov., holotype. Scale bars: 1 mm.

**Figures 54–57.**
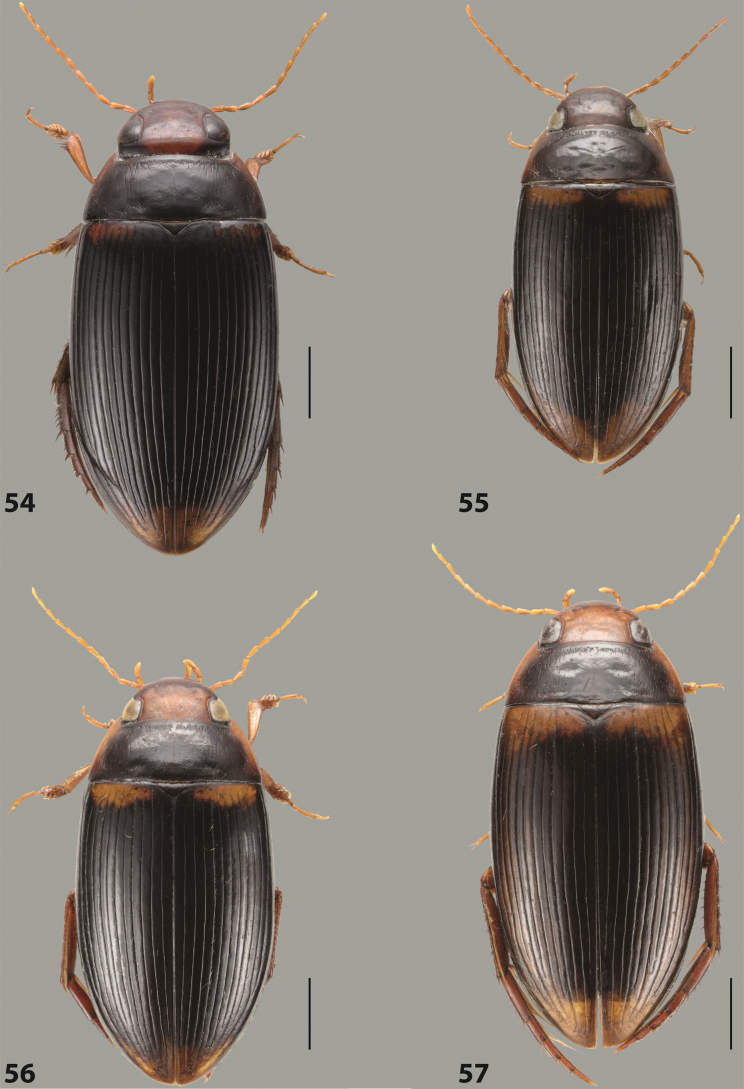
Habitus and colouration of the holotypes of **54***Austrelatuswasurensis* sp. nov. **55***A.yeretuar* sp. nov. **56***A.epicharis* sp. nov. **57***A.asteios* sp. nov. Scale bars: 1 mm.

**Figure 58.**
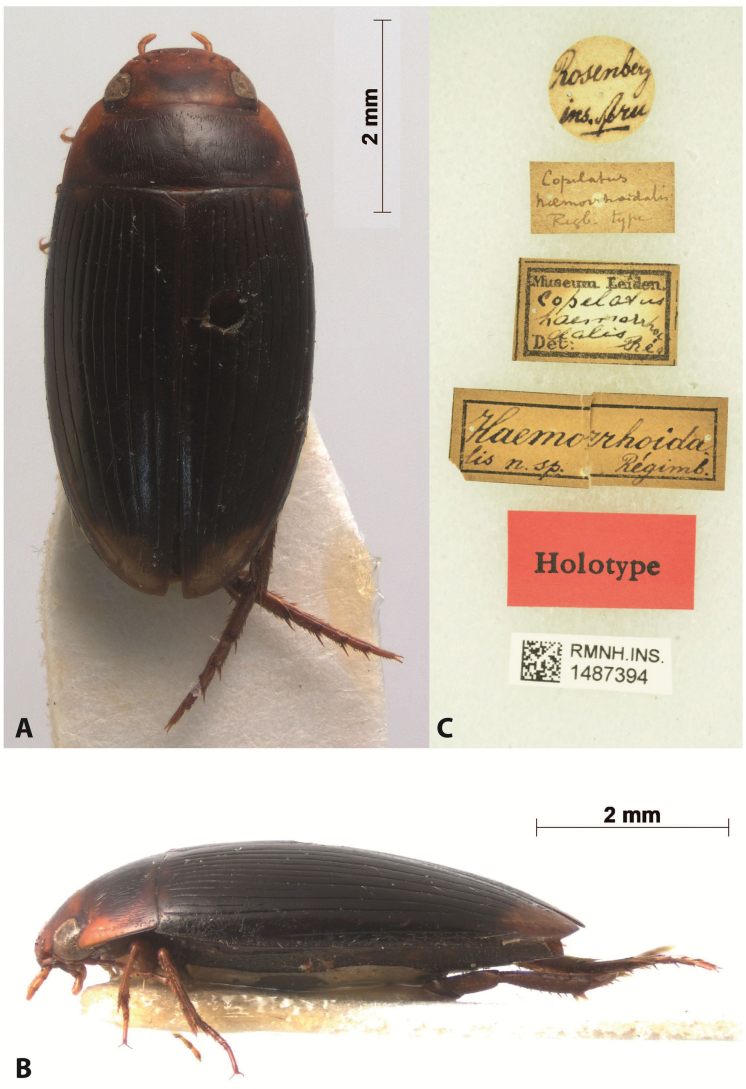
*Copelatushaemorrhoidalis* Régimbart, 1883, holotype (RMNH.INS.1487394), female **A** dorsal view **B** lateral view **C** labels. Photographs by Yvonne van Dam (RMNH).

**Figure 59.**
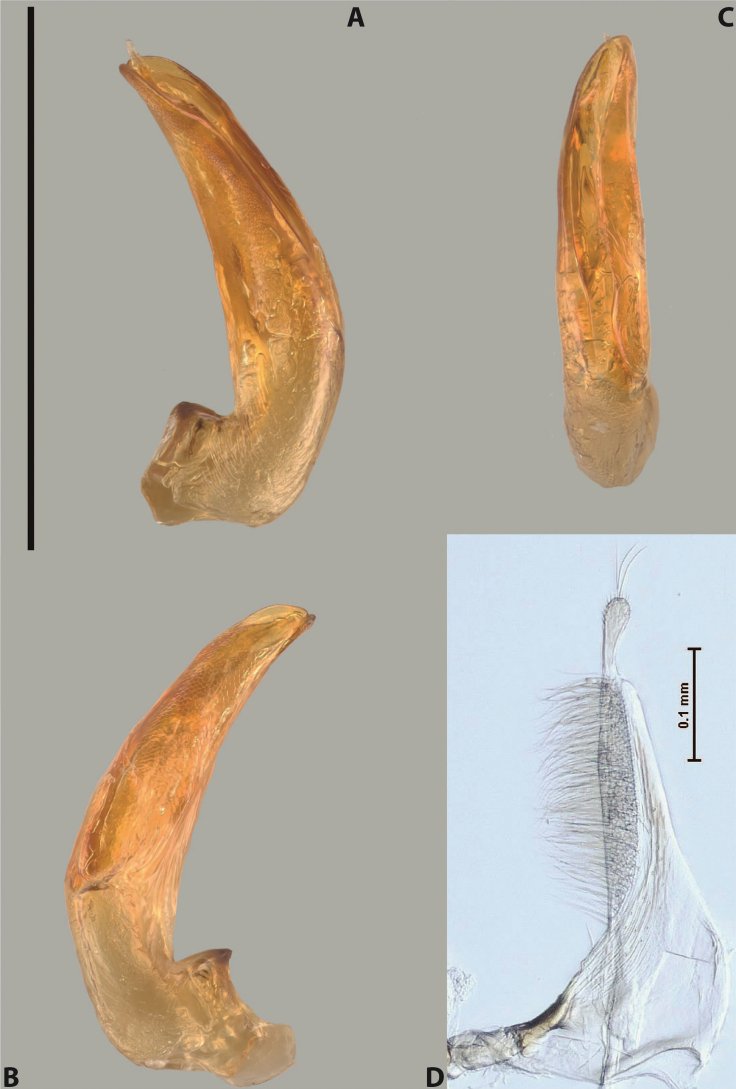
*Austrelatusleptos* sp. nov., holotype, median lobe **A** left lateral view **B** right lateral view **C** ventral view **D** left paramere in external view. Scale bar: 1 mm (**A–C**).

**Figure 60.**
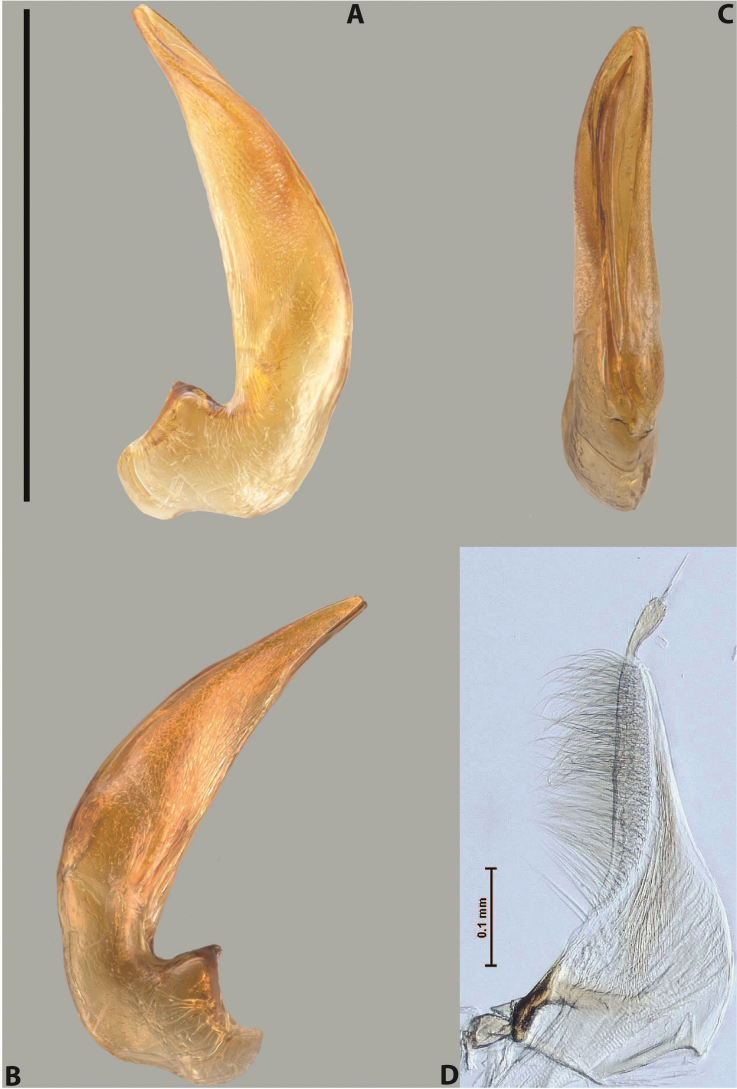
*Austrelatusprocerus* sp. nov., holotype, median lobe **A** left lateral view **B** right lateral view **C** ventral view **D** left paramere in external view. Scale bar: 1 mm (**A–C**).

**Figure 61.**
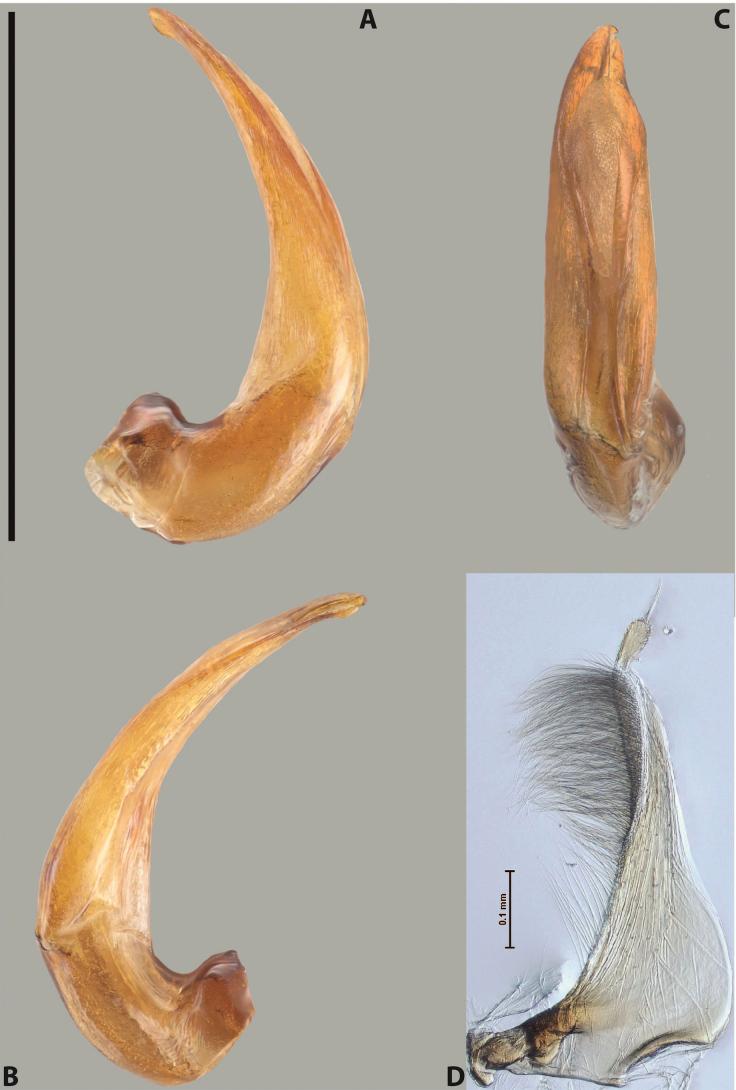
*Austrelatusxanthocephalus* (Régimbart, 1899), Ransiki - Anggi (BH03), median lobe **A** left lateral view **B** right lateral view **C** ventral view **D** left paramere in external view. Scale bar: 1 mm (**A–C**).

**Figure 62.**
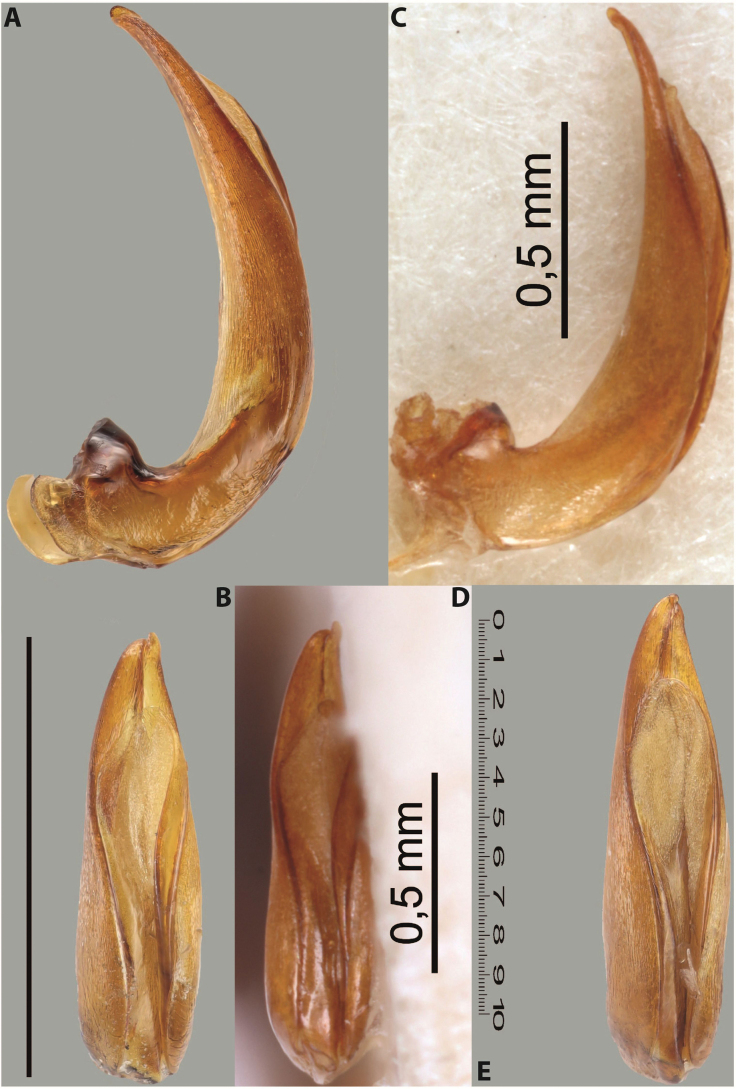
*Austrelatusxanthocephalus* (Régimbart, 1899), median lobe **A, C** left lateral view **B, D, E** ventral view **A, B** Kebar (BH035) **C, D** lectotype, photographs by Ch. Rivier (MNHN) **E** Testega (BH054). Scale bar: 1 mm (**A, B, E**).

**Figure 63.**
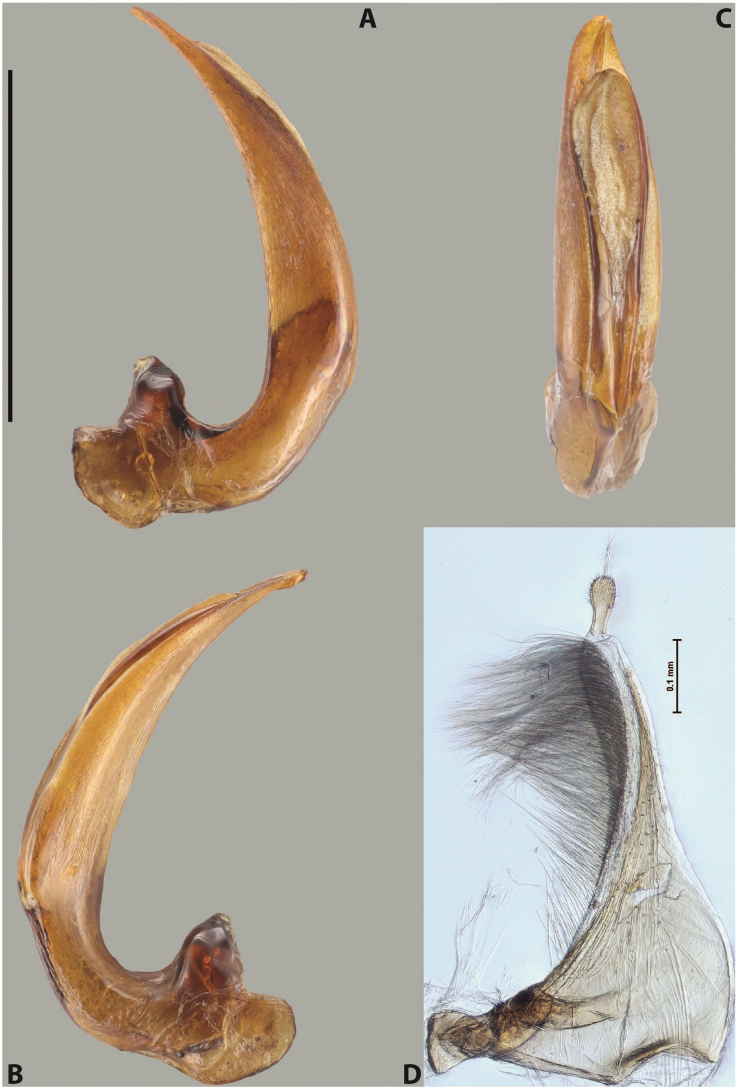
*Austrelatusxanthocephalusnabirensis* ssp. nov., holotype, median lobe **A** left lateral view **B** right lateral view **C** ventral view **D** left paramere in external view. Scale bar: 1 mm (**A–C**).

**Figure 64.**
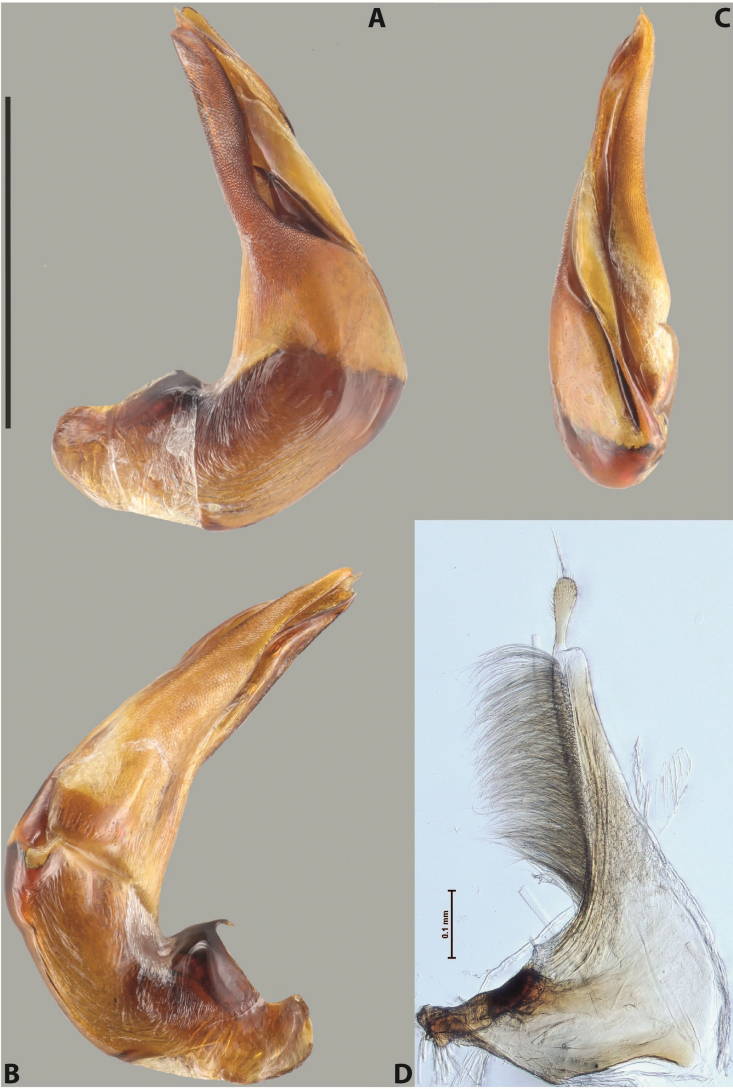
*Austrelatusbundunensis* sp. nov., holotype, median lobe **A** left lateral view **B** right lateral view **C** ventral view **D** left paramere in external view. Scale bar: 1 mm (**A–C**).

**Figure 65.**
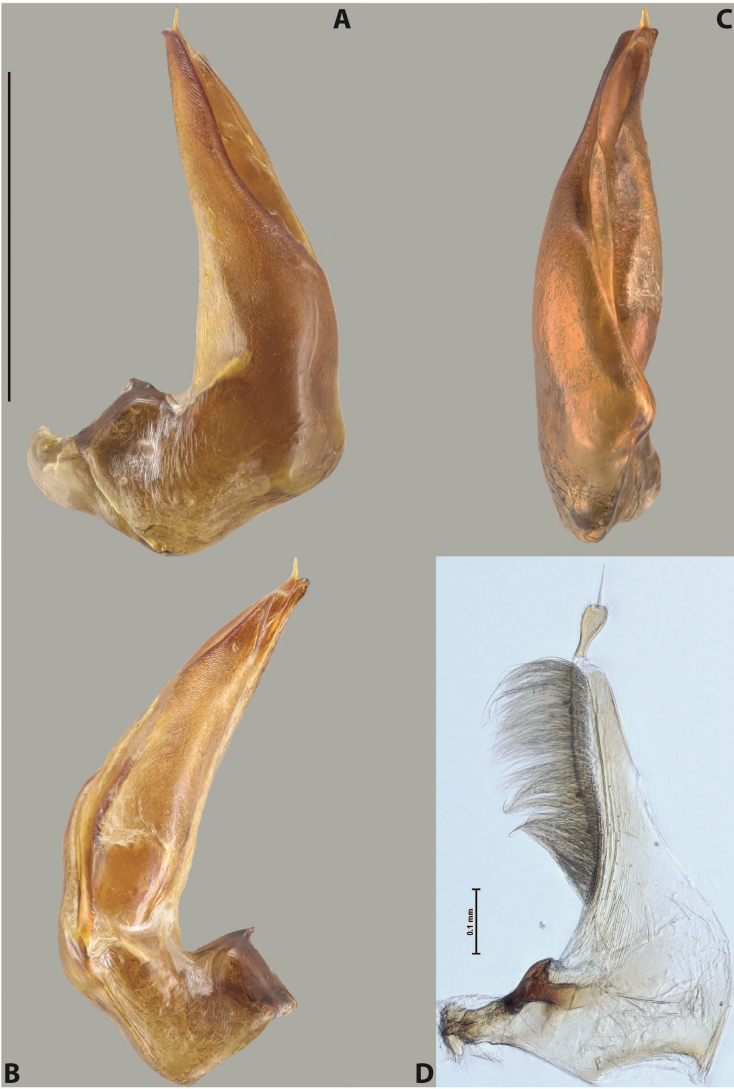
*Austrelatusinconstans* sp. nov., paratype, median lobe **A** left lateral view **B** right lateral view **C** ventral view **D** left paramere in external view. Scale bar: 1 mm (**A–C**).

**Figure 66.**
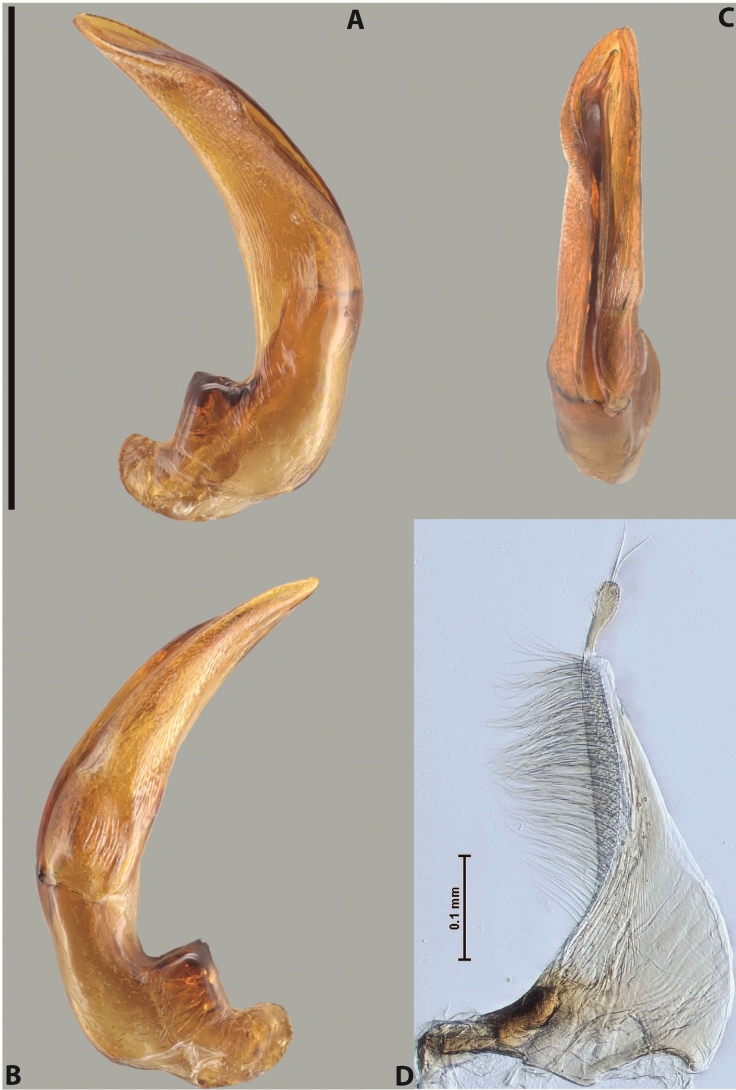
*Austrelatuslopintolensis* sp. nov., holotype, median lobe **A** left lateral view **B** right lateral view **C** ventral view **D** left paramere in external view. Scale bar: 1 mm (**A–C**).

**Figure 67.**
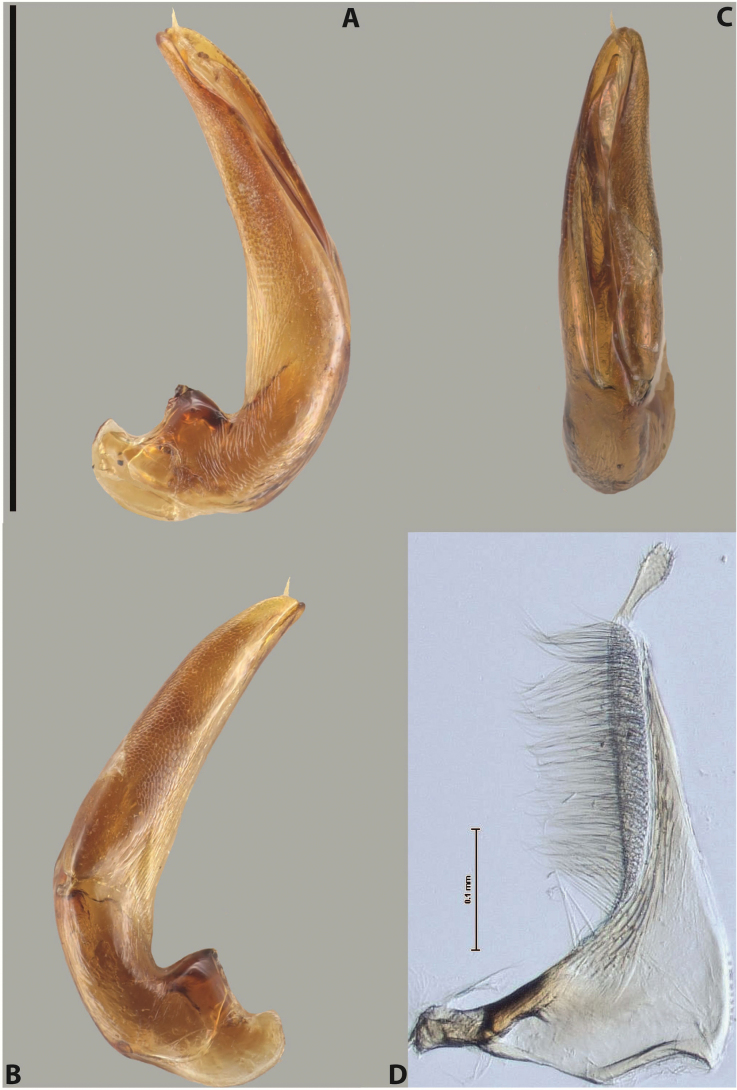
*Austrelatusohu* sp. nov., holotype, median lobe **A** left lateral view **B** right lateral view **C** ventral view **D** left paramere in external view. Scale bar: 1 mm (**A–C**).

**Figure 68.**
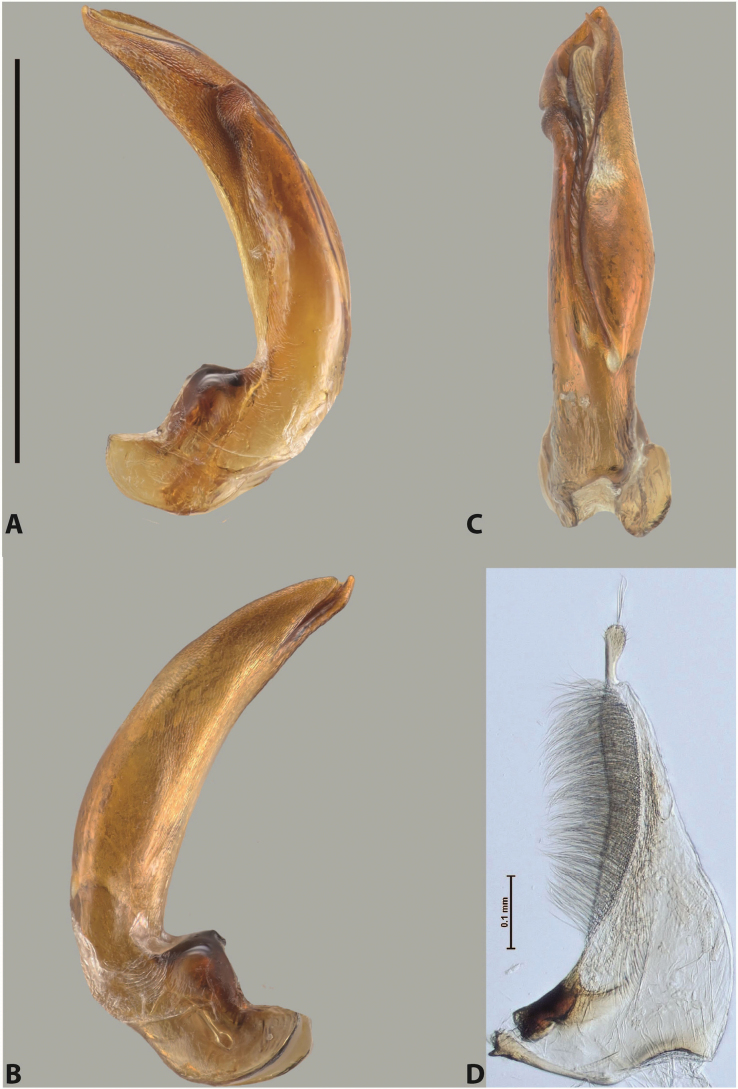
*Austrelatusyamurensis* sp. nov., holotype, median lobe **A** left lateral view **B** right lateral view **C** ventral view **D** left paramere in external view. Scale bar: 1 mm (**A–C**).

**Figure 69.**
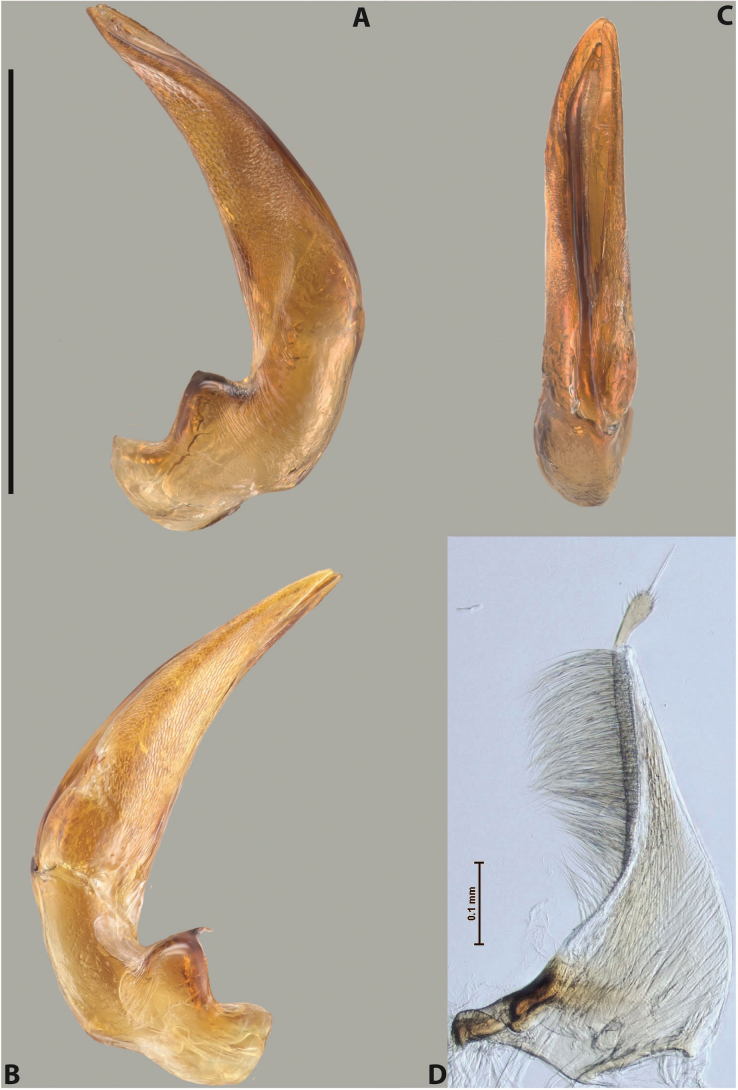
*Austrelatuskebarensis* sp. nov., holotype, median lobe **A** left lateral view **B** right lateral view **C** ventral view **D** left paramere in external view. Scale bar: 1 mm (**A–C**).

**Figure 70.**
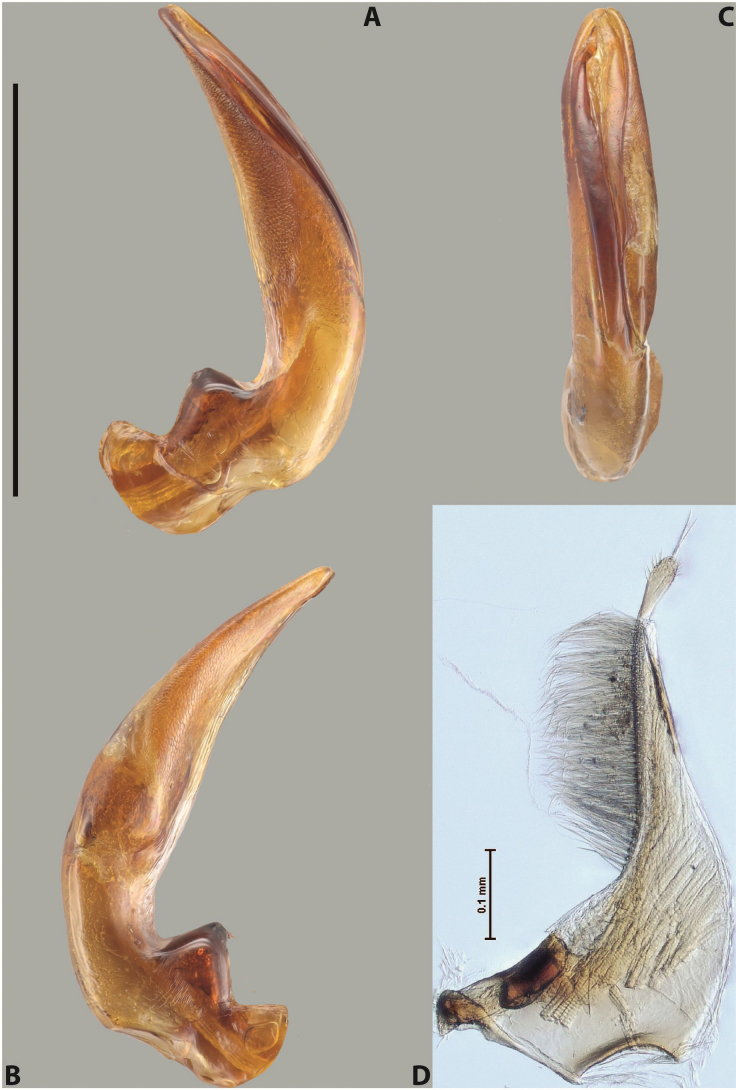
*Austrelatusluteomaculatus* (Guignot, 1956), median lobe **A** left lateral view **B** right lateral view **C** ventral view **D** left paramere in external view. Scale bar: 1 mm (**A–C**).

**Figure 71.**
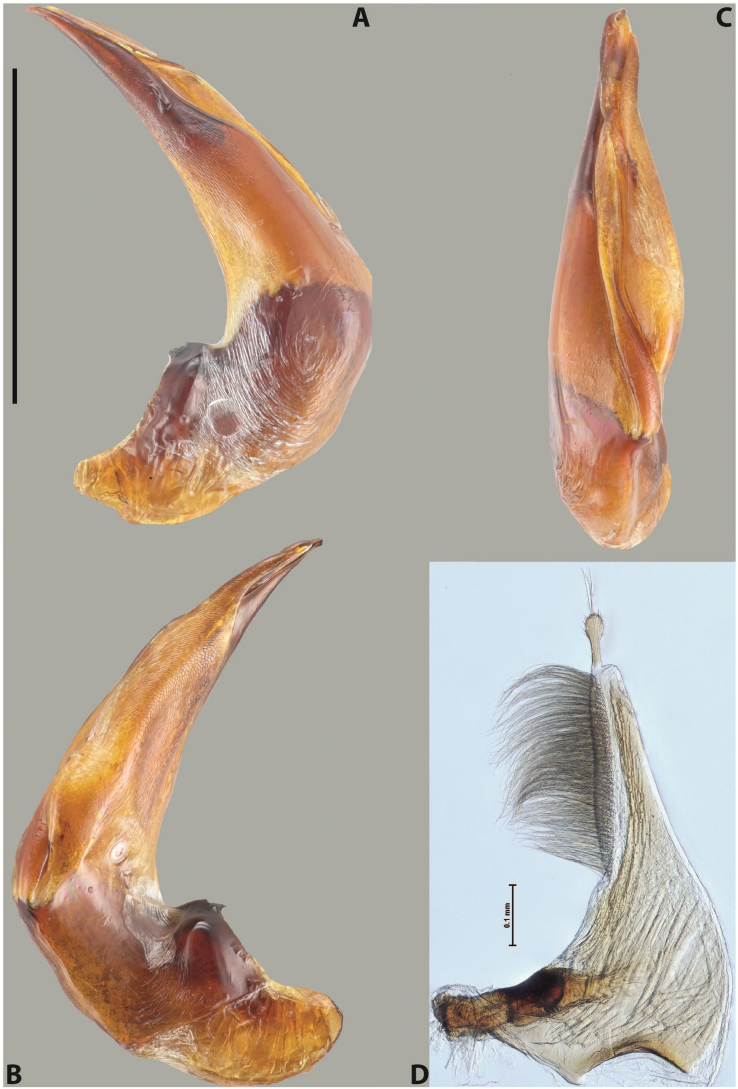
*Austrelatuscentralensis* sp. nov., paratype, median lobe **A** left lateral view **B** right lateral view **C** ventral view **D** left paramere in external view. Scale bar: 1 mm (**A–C**).

**Figure 72.**
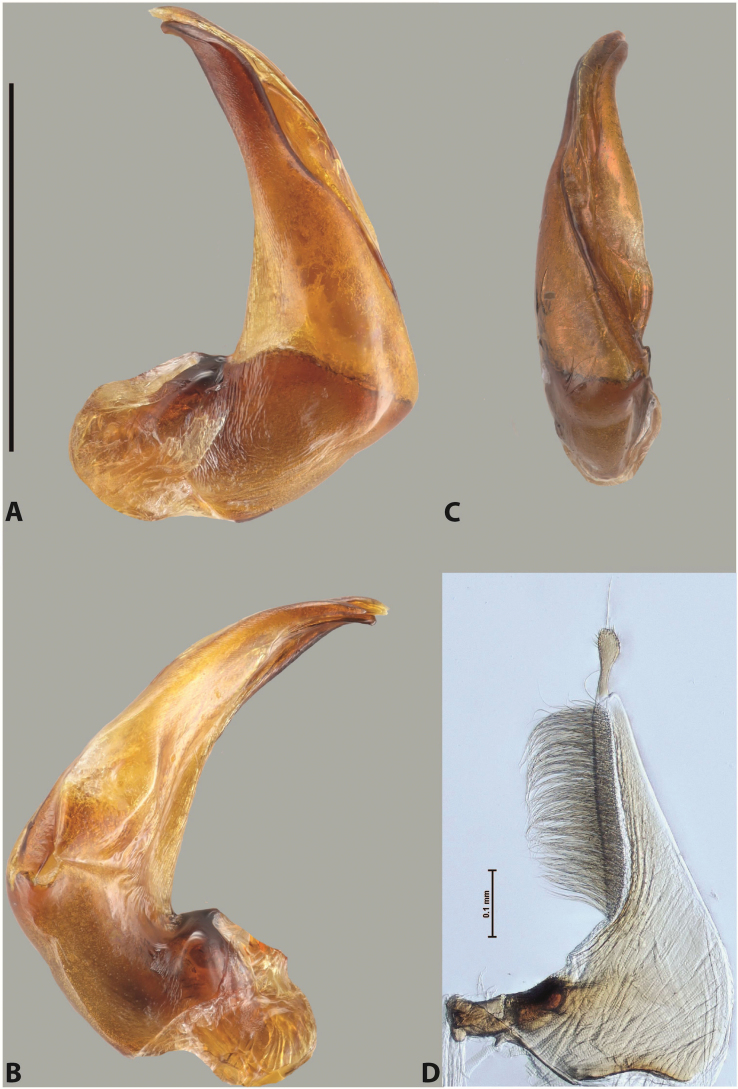
*Austrelatusnormanbyensis* sp. nov., holotype, median lobe **A** left lateral view **B** right lateral view **C** ventral view **D** left paramere in external view. Scale bar: 1 mm (**A–C**).

**Figure 73.**
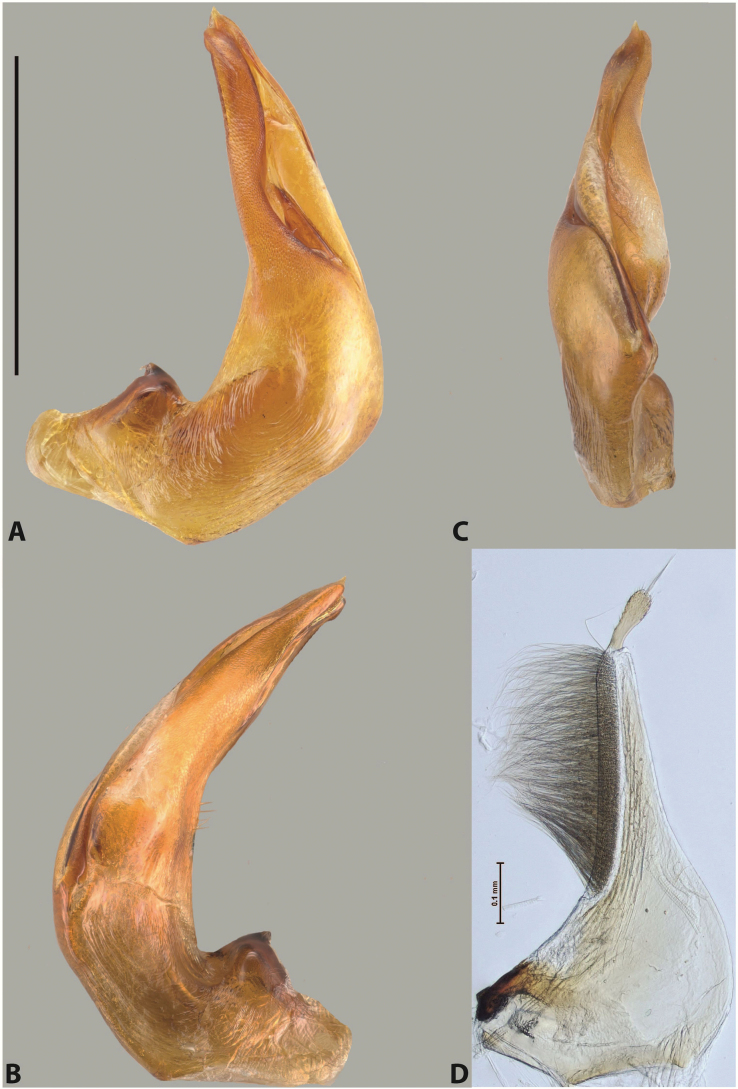
*Austrelatusmiltokarenos* sp. nov., holotype, median lobe **A** left lateral view **B** right lateral view **C** ventral view **D** left paramere in external view. Scale bar: 1 mm (**A–C**).

**Figure 74.**
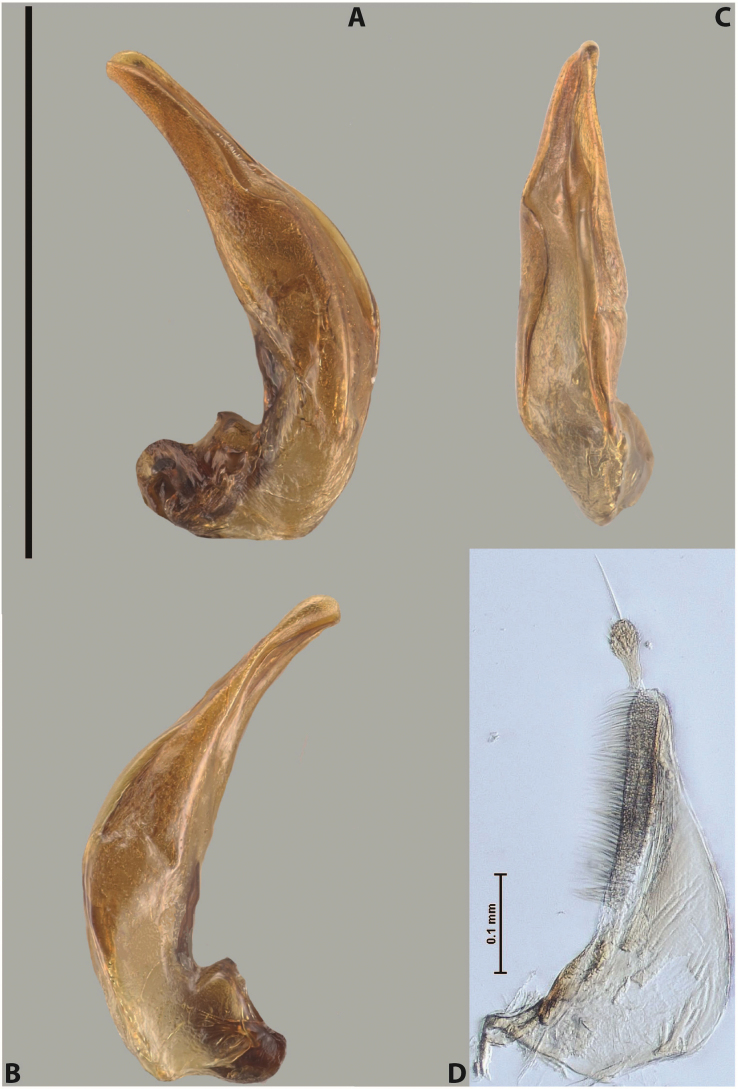
*Austrelatusloloki* sp. nov., holotype, median lobe **A** left lateral view **B** right lateral view **C** ventral view **D** left paramere in external view. Scale bar: 1 mm (**A–C**).

**Figure 75.**
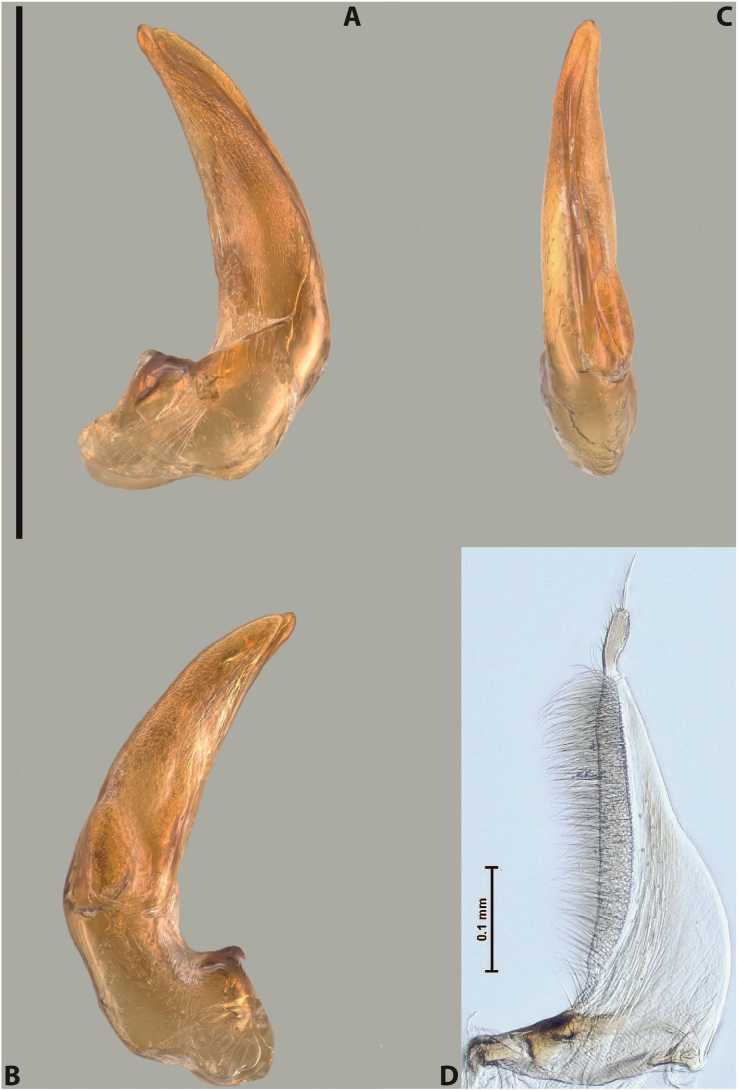
*Austrelatussumokedi* sp. nov., holotype, median lobe **A** left lateral view **B** right lateral view **C** ventral view **D** left paramere in external view. Scale bar: 1 mm (**A–C**).

**Figure 76.**
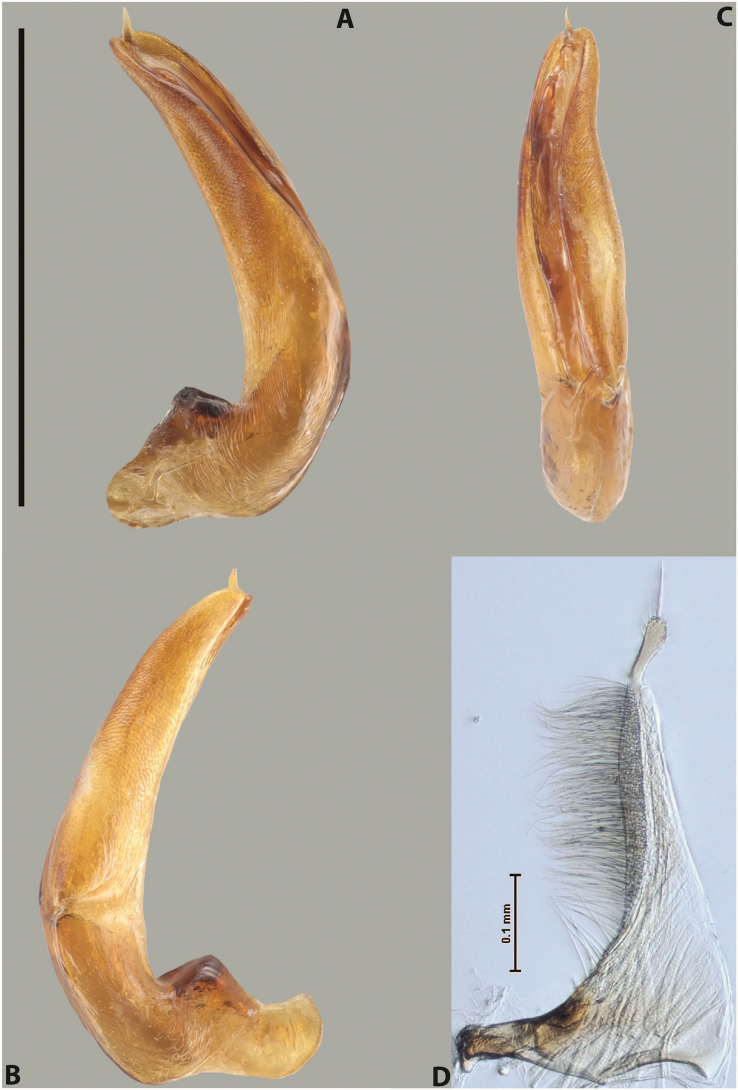
*Austrelatusiriatoi* sp. nov., holotype, median lobe **A** left lateral view **B** right lateral view **C** ventral view **D** left paramere in external view. Scale bar: 1 mm (**A–C**).

**Figure 77.**
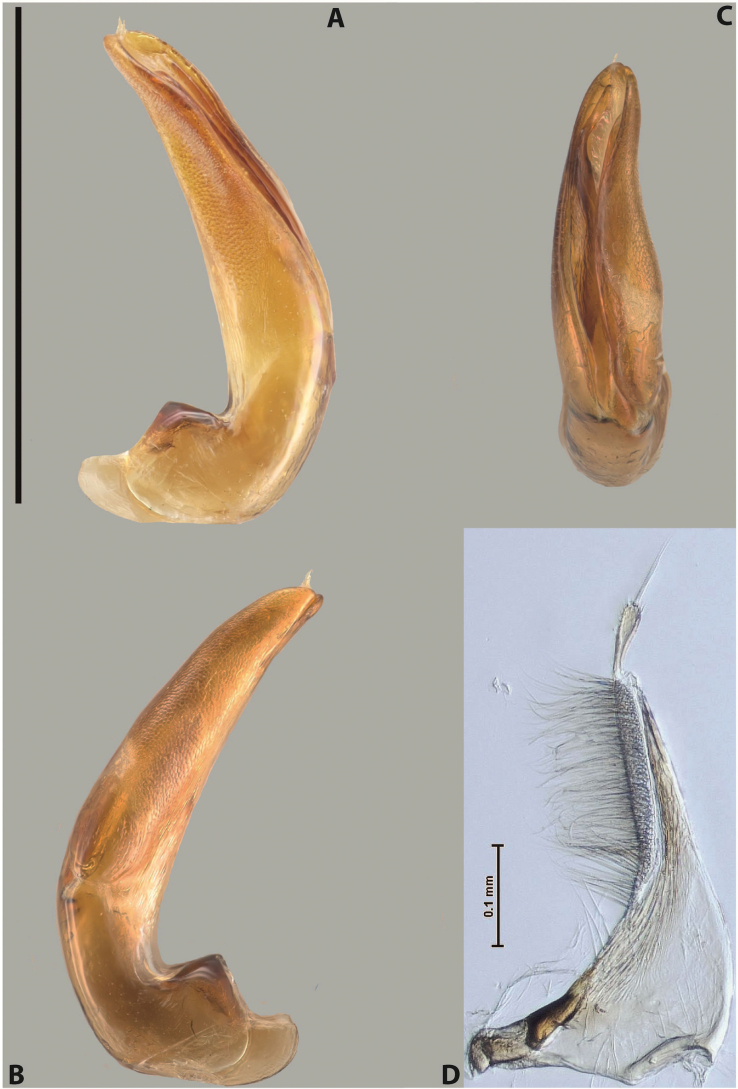
*Austrelatuswanangensis* sp. nov., holotype, median lobe **A** left lateral view **B** right lateral view **C** ventral view **D** left paramere in external view. Scale bar: 1 mm (**A–C**).

**Figure 78.**
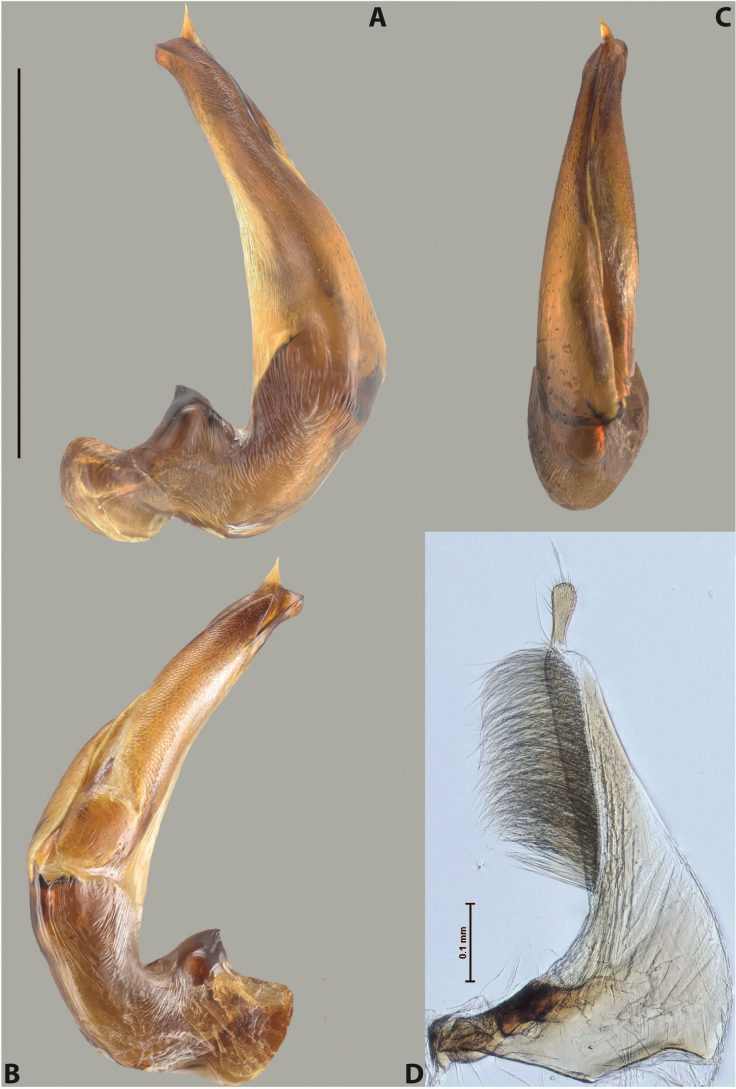
*Austrelatusdecoris* sp. nov., holotype, median lobe **A** left lateral view **B** right lateral view **C** ventral view **D** left paramere in external view. Scale bar: 1 mm (**A–C**).

**Figure 79.**
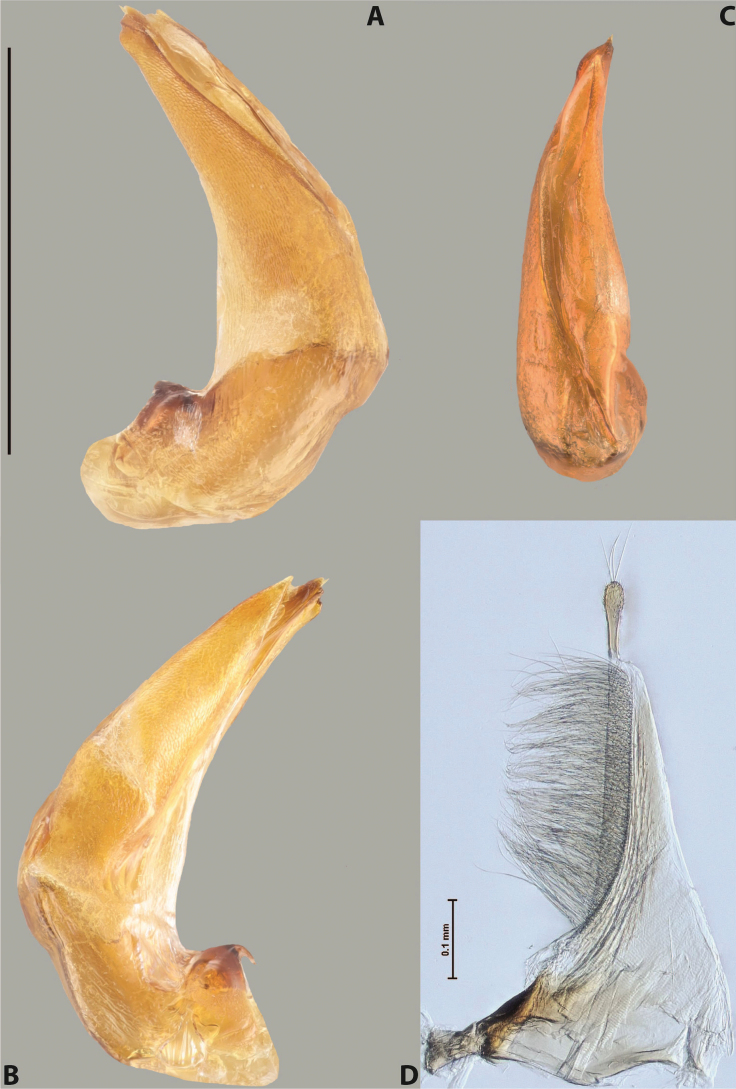
*Austrelatuscraterensis* sp. nov., holotype, median lobe **A** left lateral view **B** right lateral view **C** ventral view **D** left paramere in external view. Scale bar: 1 mm (**A–C**).

**Figure 80.**
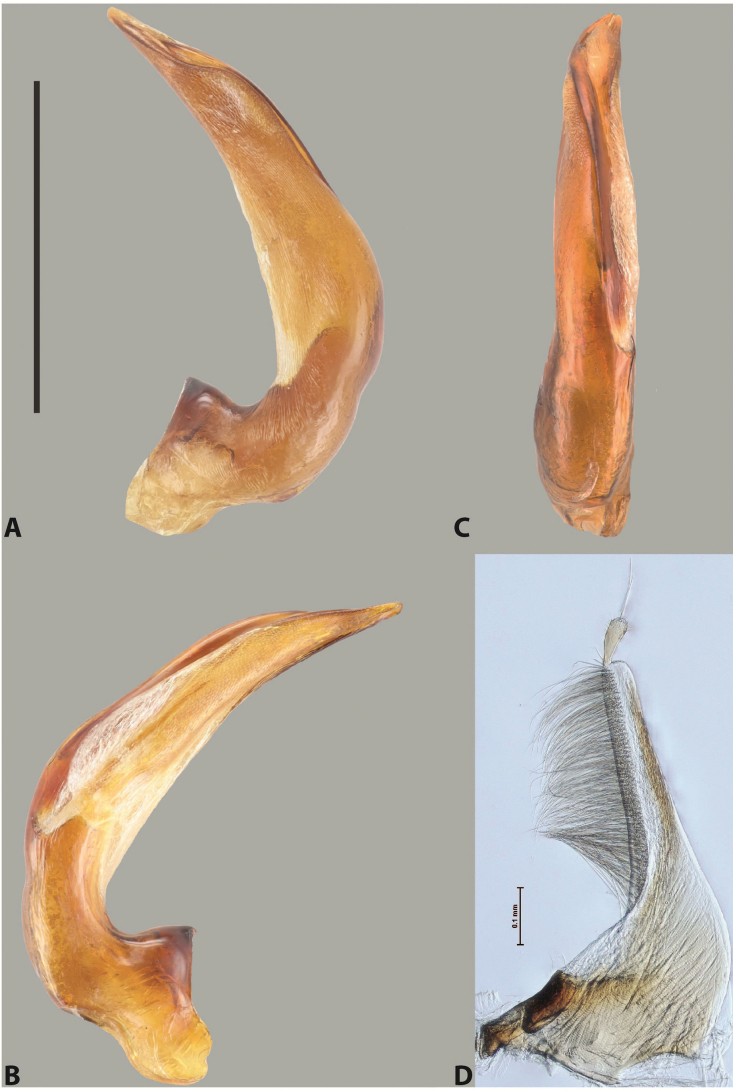
*Austrelatusaiyurensis* sp. nov., holotype, median lobe **A** left lateral view **B** right lateral view **C** ventral view **D** left paramere in external view. Scale bar: 1 mm (**A–C**).

**Figure 81.**
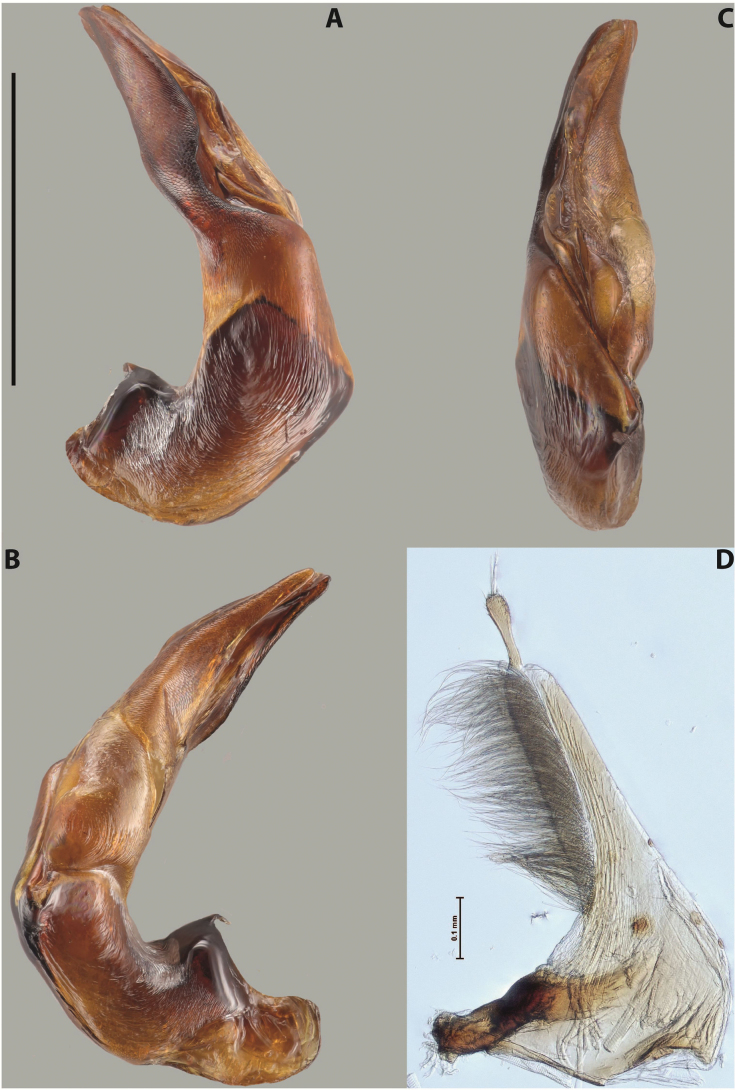
*Austrelatusrobustus* sp. nov., holotype, median lobe **A** left lateral view **B** right lateral view **C** ventral view **D** left paramere in external view. Scale bar: 1 mm (**A–C**).

**Figure 82.**
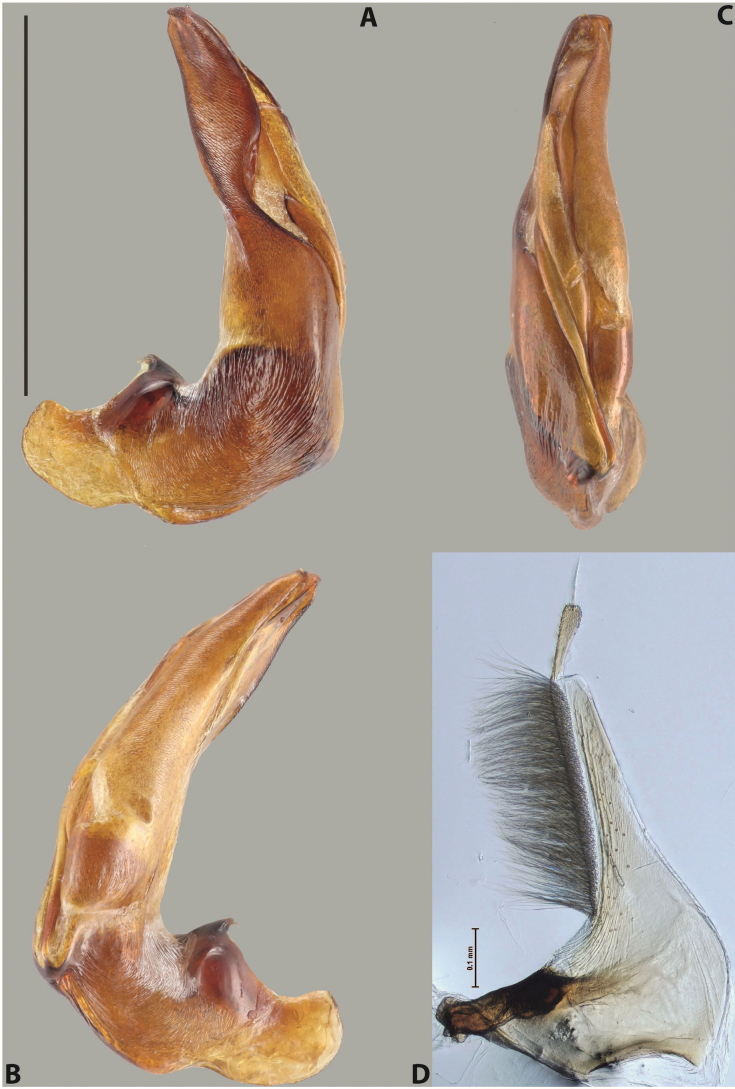
*Austrelatusbosaviensis* sp. nov., holotype, median lobe **A** left lateral view **B** right lateral view **C** ventral view **D** left paramere in external view. Scale bar: 1 mm (**A–C**).

**Figure 83.**
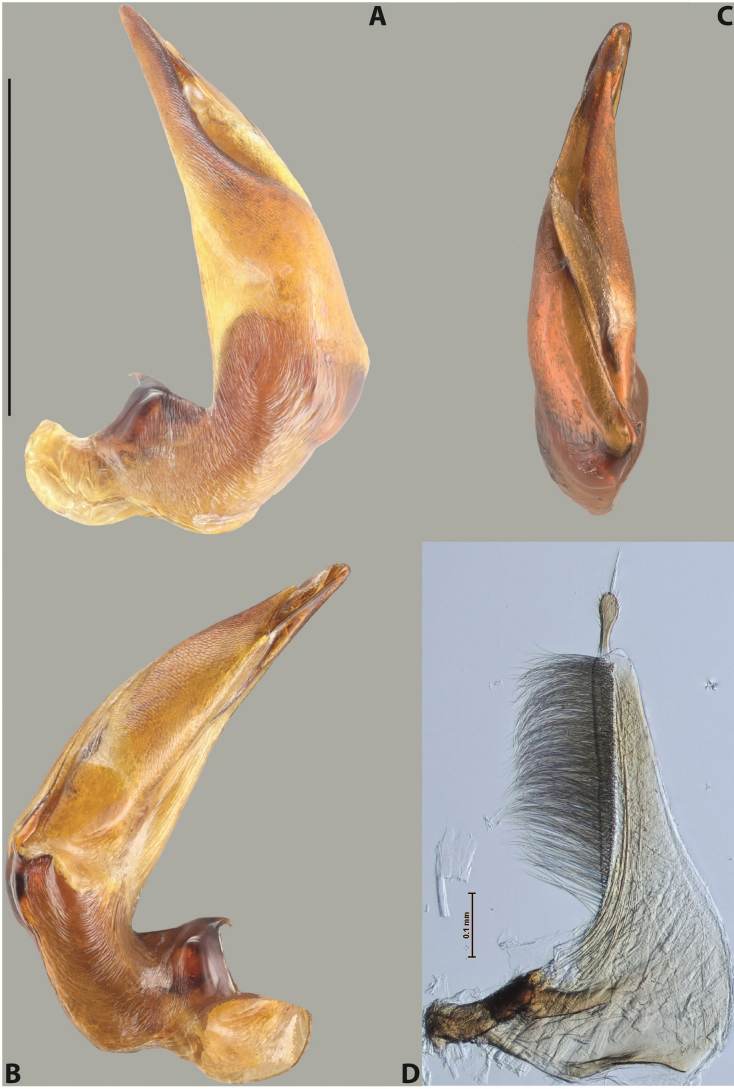
*Austrelatusdekai* sp. nov., holotype, median lobe **A** left lateral view **B** right lateral view **C** ventral view **D** left paramere in external view. Scale bar: 1 mm (**A–C**).

**Figure 84.**
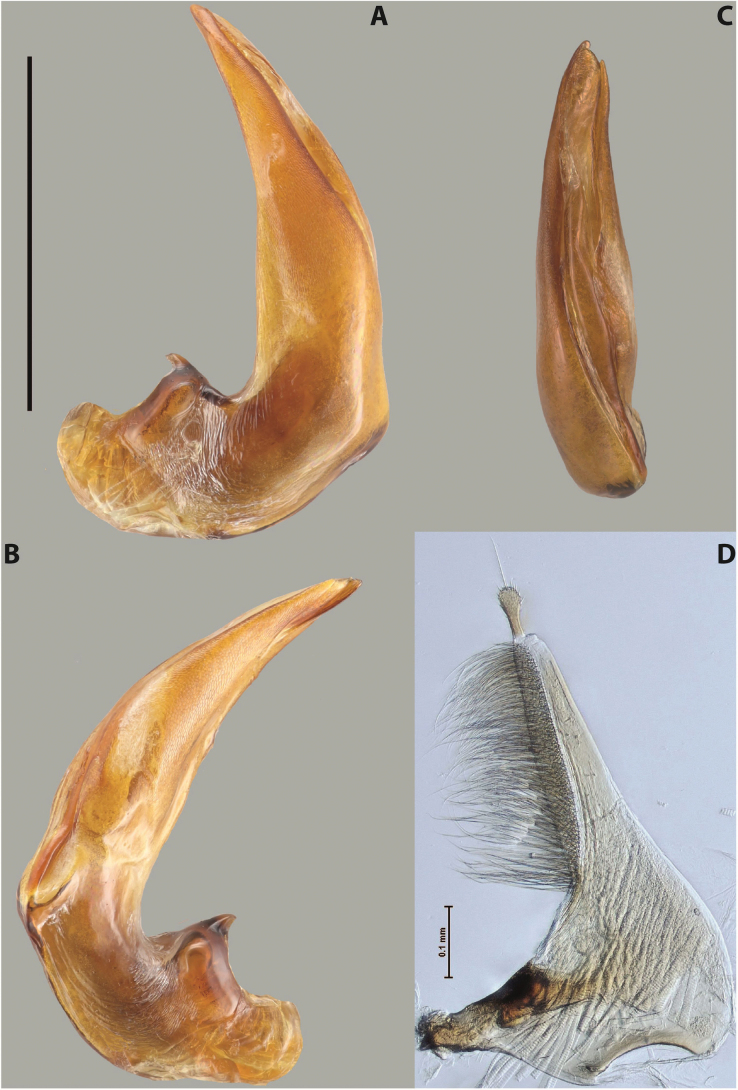
*Austrelatusposmani* sp. nov., holotype, median lobe **A** left lateral view **B** right lateral view **C** ventral view **D** left paramere in external view. Scale bar: 1 mm (**A–C**).

**Figure 85.**
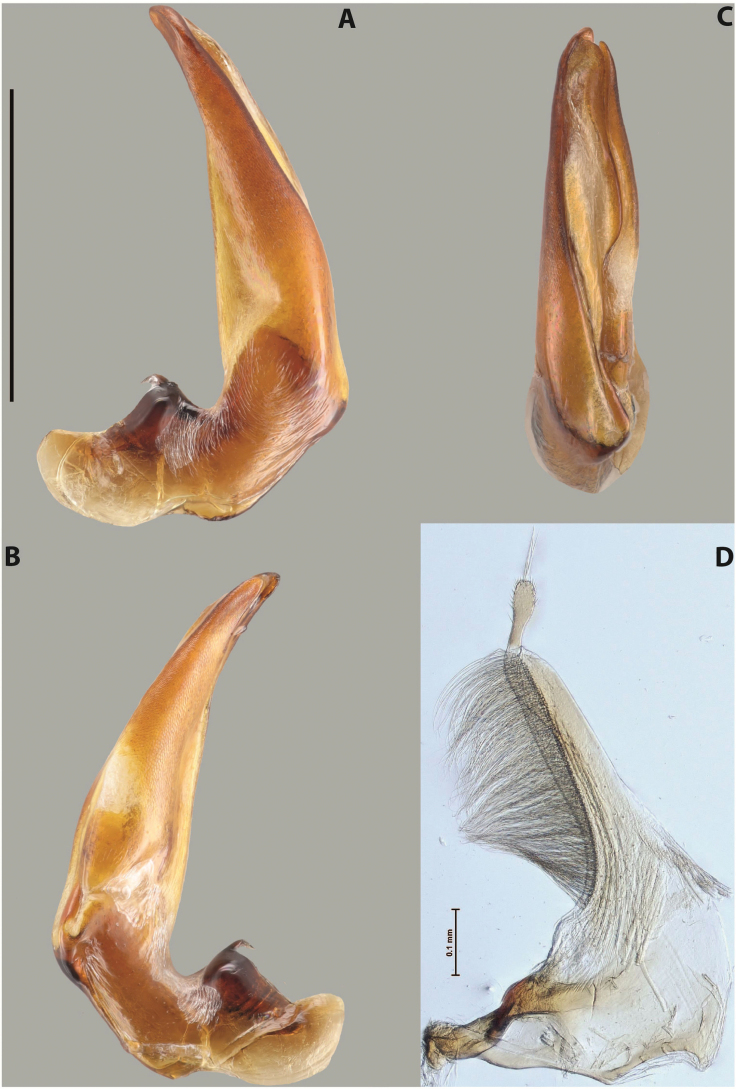
*Austrelatuspapuensis* (J. Balfour-Browne, 1939), median lobe **A** left lateral view **B** right lateral view **C** ventral view **D** left paramere in external view. Scale bar: 1 mm (**A–C**).

**Figure 86.**
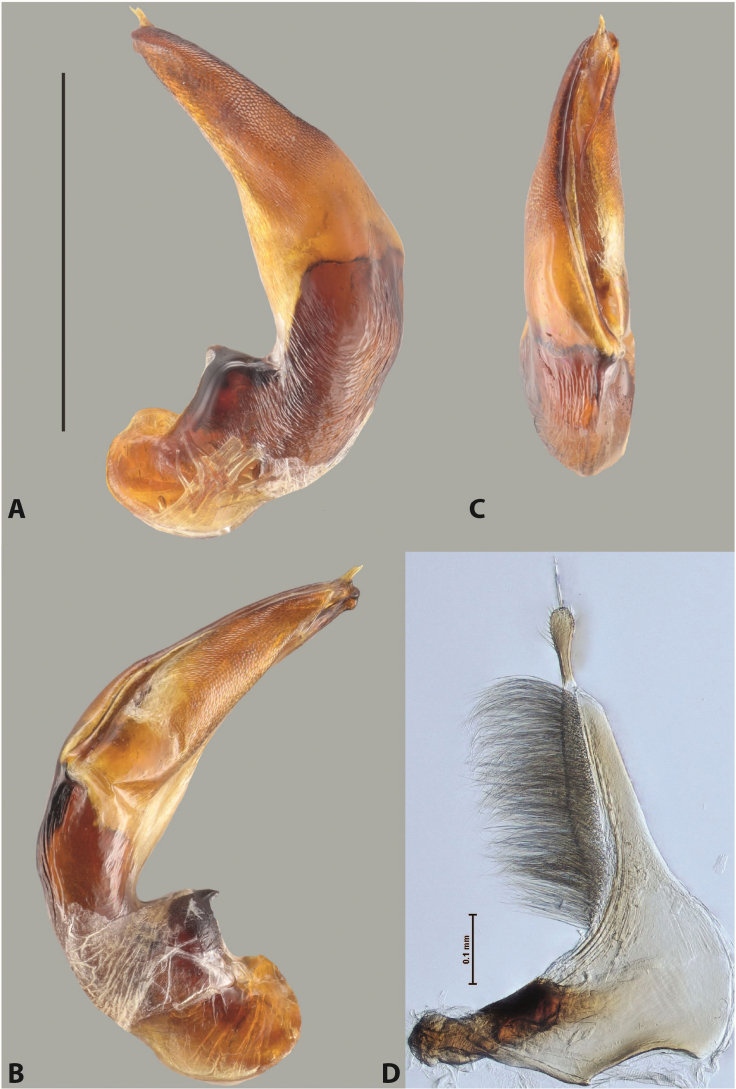
*Austrelatusbewaniensis* sp. nov., holotype, median lobe **A** left lateral view **B** right lateral view **C** ventral view **D** left paramere in external view. Scale bar: 1 mm (**A–C**).

**Figure 87.**
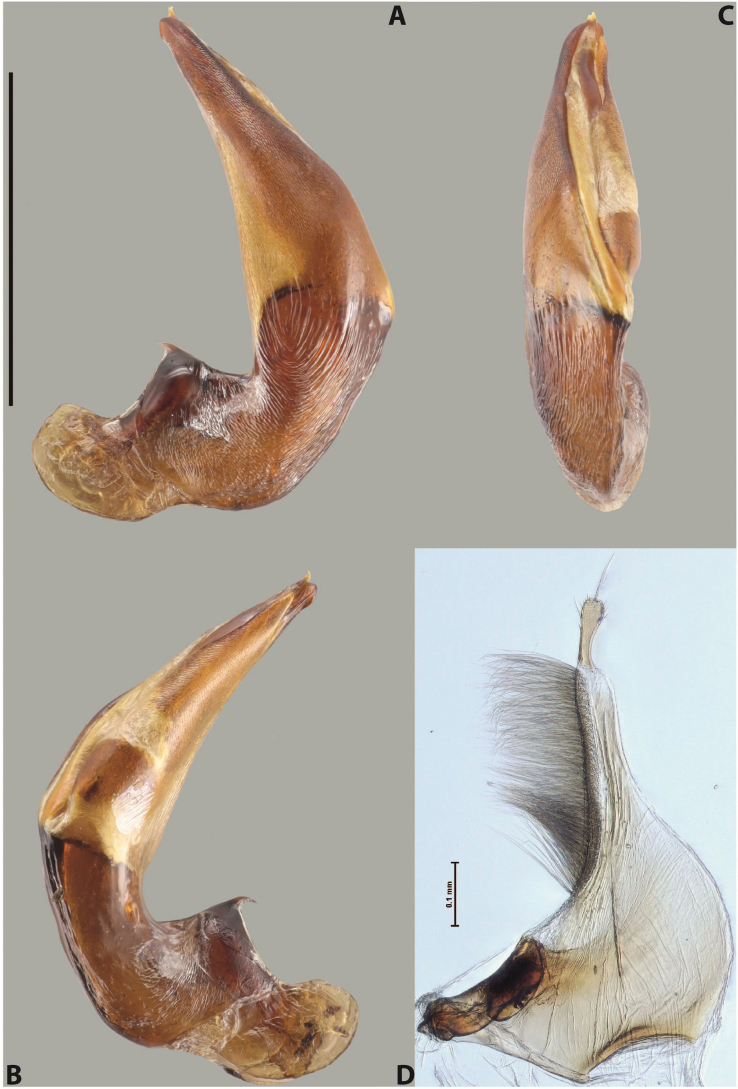
*Austrelatusnoiadi* sp. nov., holotype, median lobe **A** left lateral view **B** right lateral view **C** ventral view **D** left paramere in external view. Scale bar: 1 mm (**A–C**).

**Figure 88.**
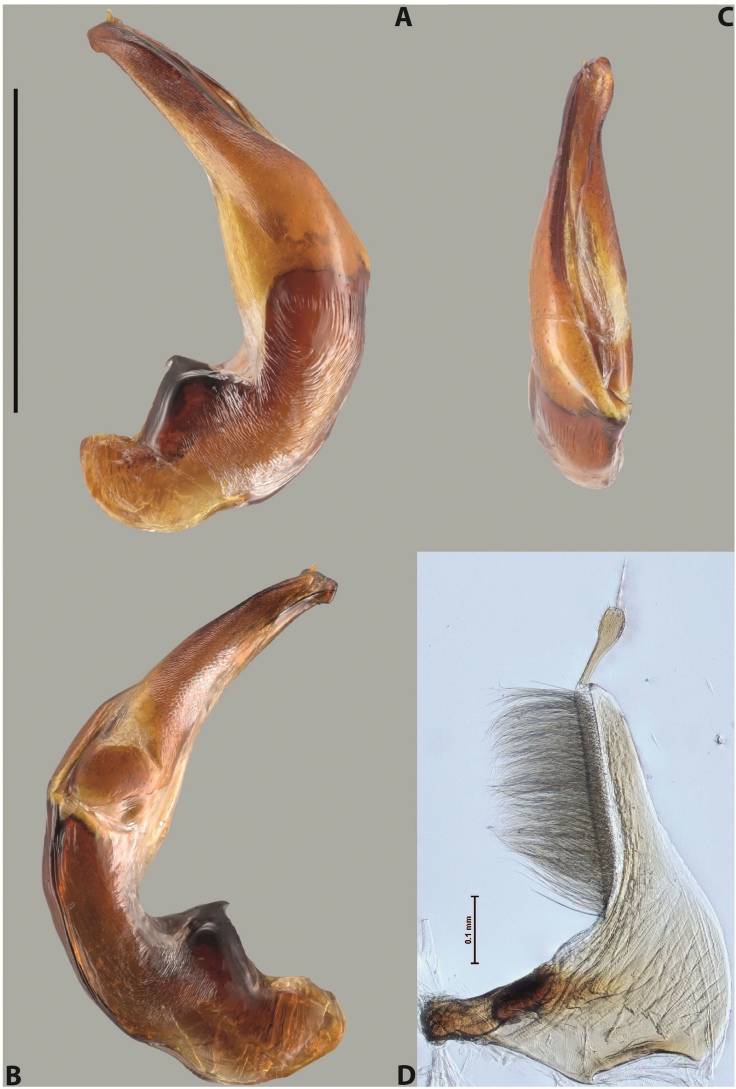
*Austrelatusmadangensis* sp. nov., holotype, median lobe **A** left lateral view **B** right lateral view **C** ventral view **D** left paramere in external view. Scale bar: 1 mm (**A–C**).

**Figure 89.**
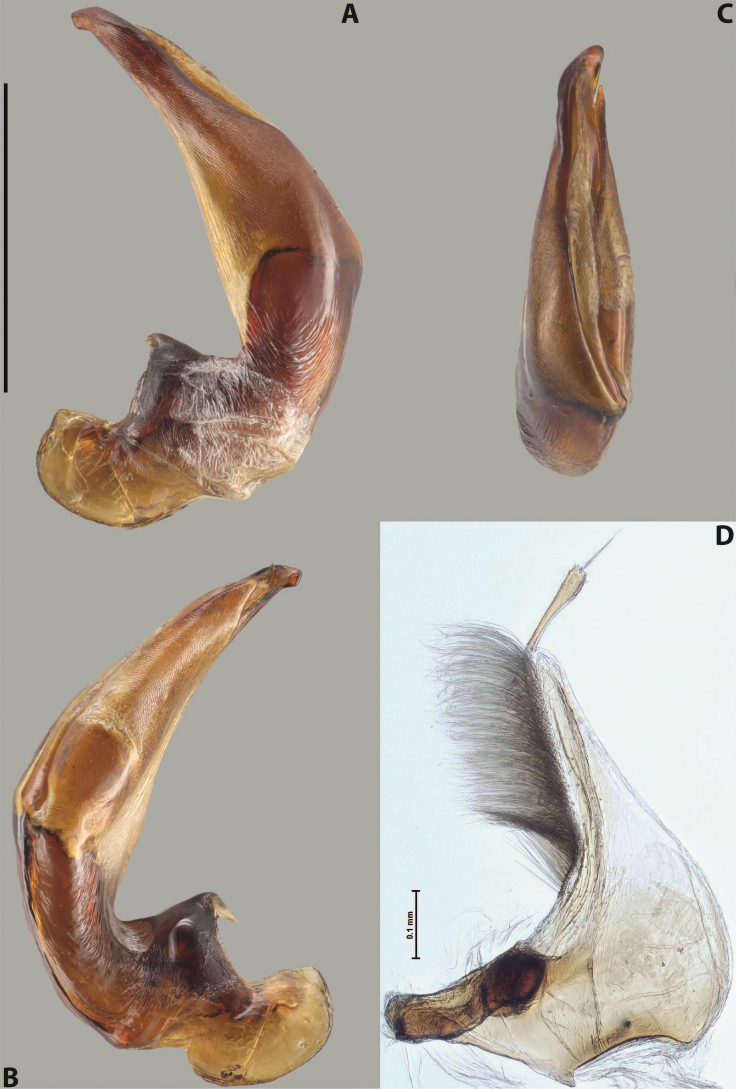
*Austrelatusmamberamo* sp. nov., holotype, median lobe **A** left lateral view **B** right lateral view **C** ventral view **D** left paramere in external view. Scale bar: 1 mm (**A–C**).

**Figure 90.**
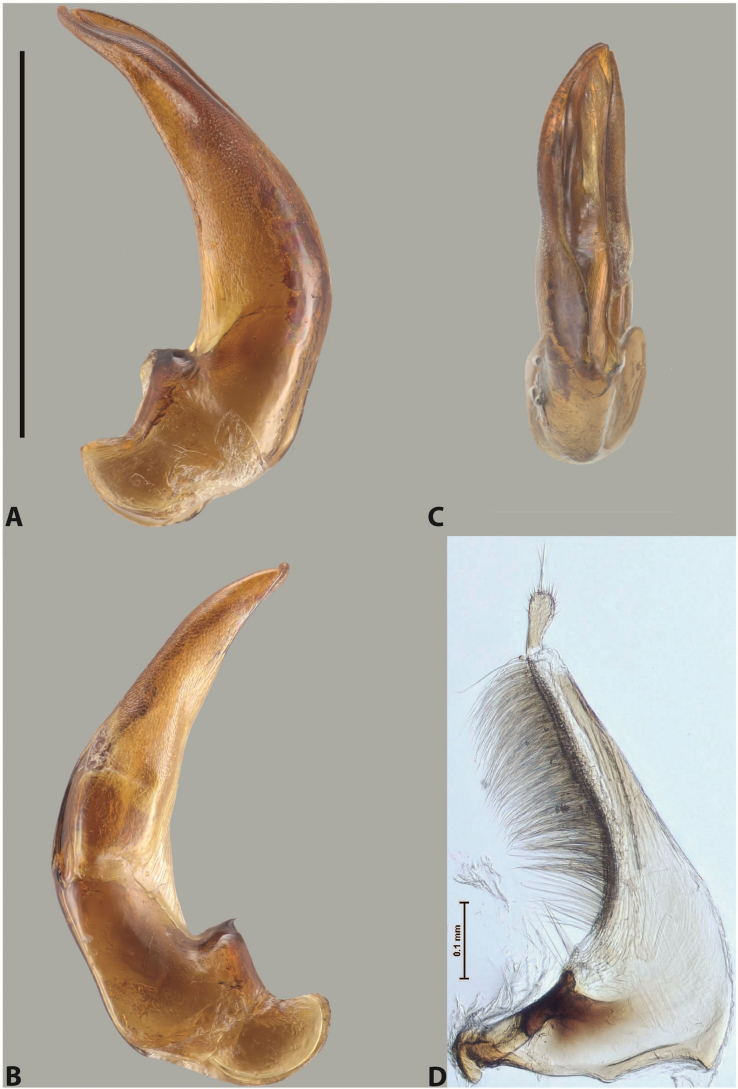
*Austrelatusweigeli* sp. nov., holotype, median lobe **A** left lateral view **B** right lateral view **C** ventral view **D** left paramere in external view. Scale bar: 1 mm (**A–C**).

**Figure 91.**
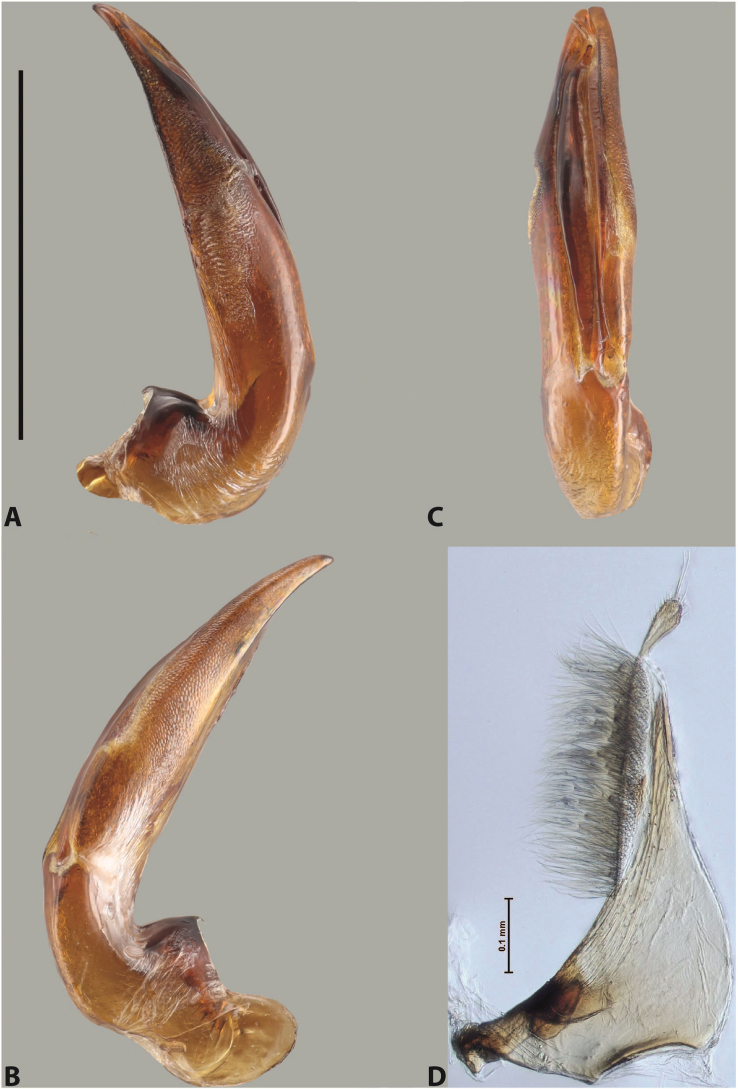
*Austrelatuskalibumi* sp. nov., holotype, median lobe **A** left lateral view **B** right lateral view **C** ventral view **D** left paramere in external view. Scale bar: 1 mm (**A–C**).

**Figure 92.**
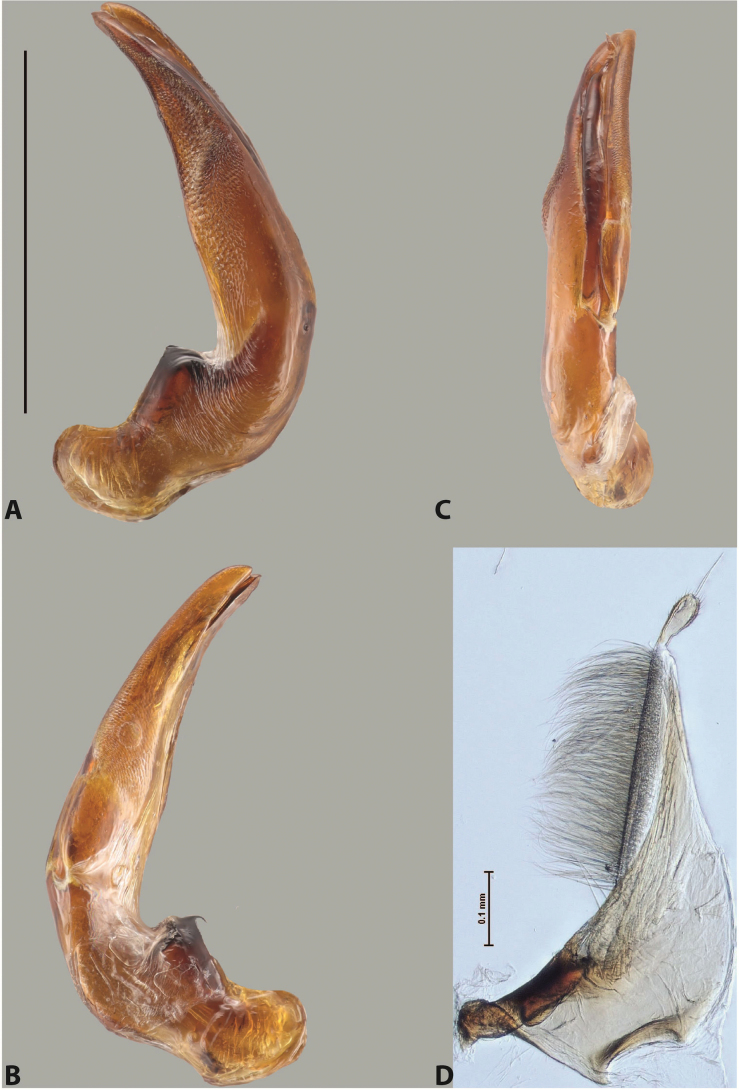
*Austrelatusherzogensis* sp. nov., holotype, median lobe **A** left lateral view **B** right lateral view **C** ventral view **D** left paramere in external view. Scale bar: 1 mm (**A–C**).

**Figure 93.**
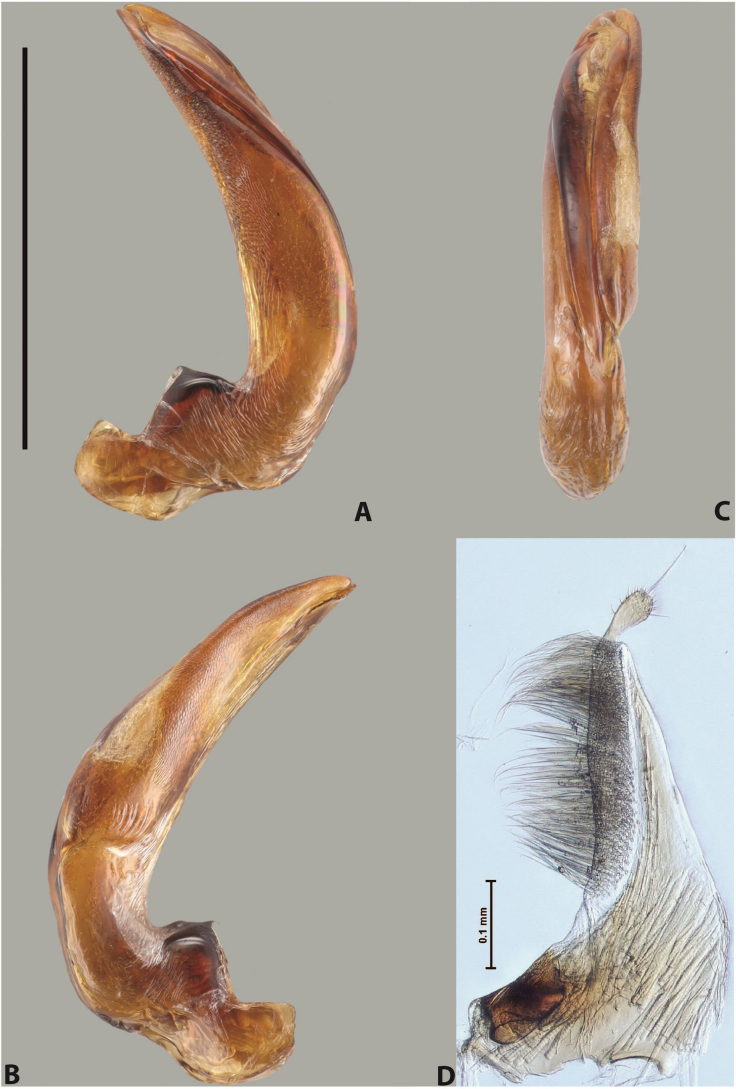
*Austrelatusmianminensis* sp. nov., holotype, median lobe **A** left lateral view **B** right lateral view **C** ventral view **D** left paramere in external view. Scale bar: 1 mm (**A–C**).

**Figure 94.**
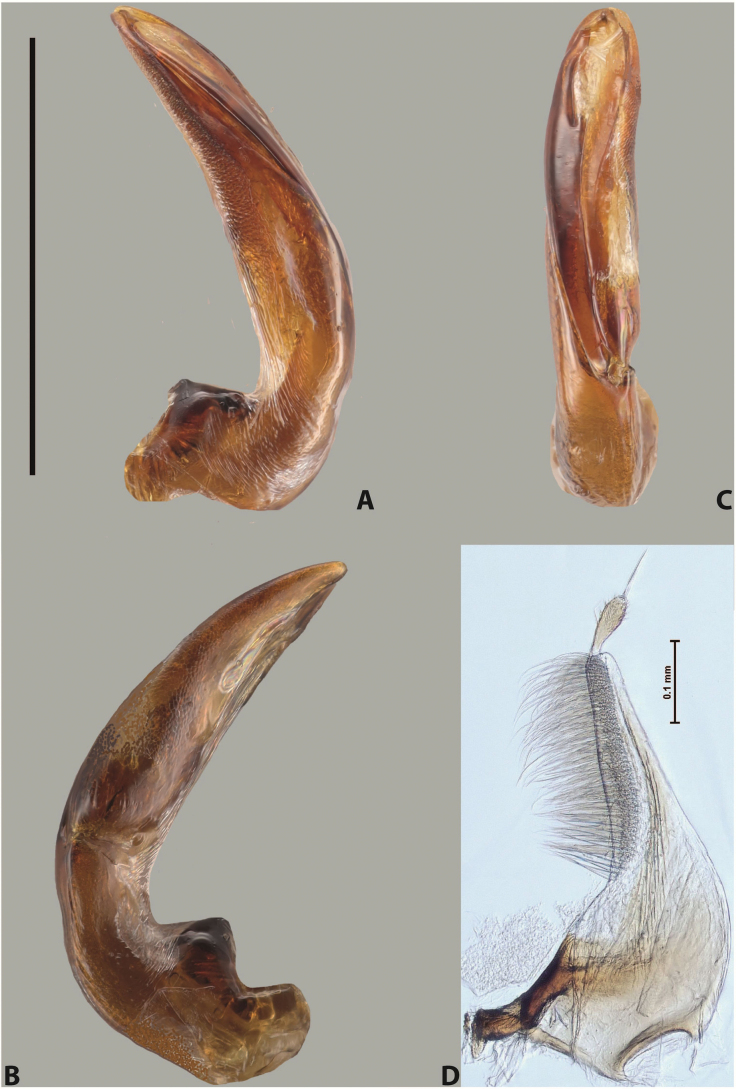
*Austrelatuspseudomianminensis* sp. nov., holotype, median lobe **A** left lateral view **B** right lateral view **C** ventral view **D** left paramere in external view. Scale bar: 1 mm (**A–C**).

**Figure 95.**
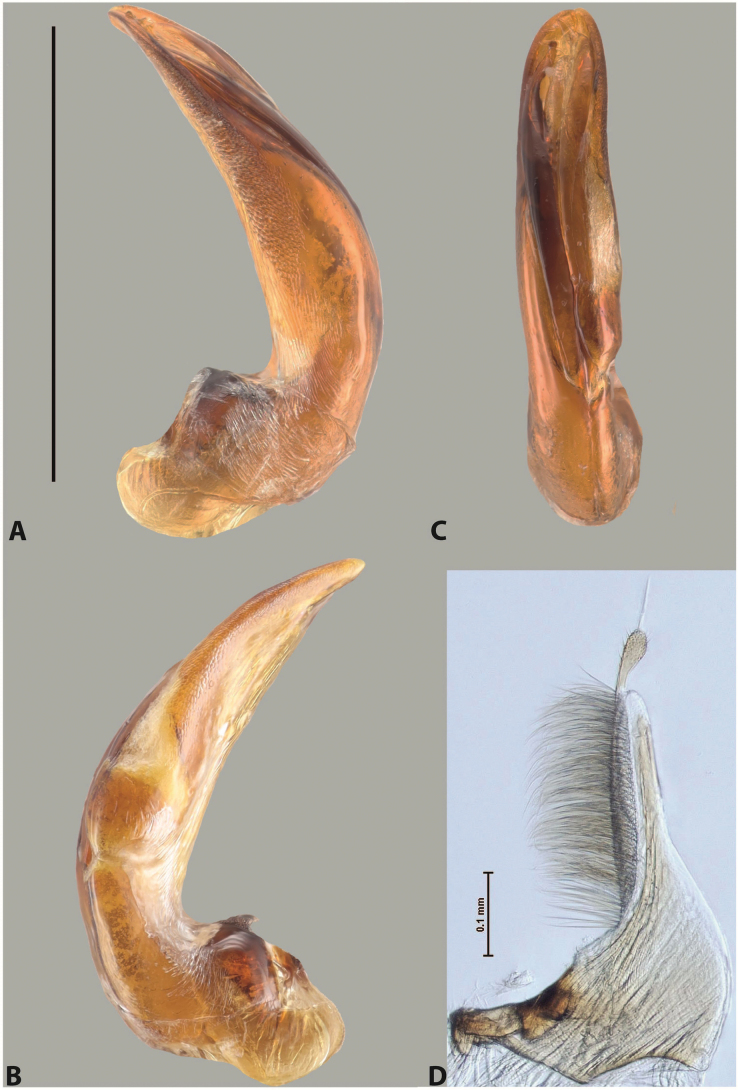
*Austrelatusflavocapitatus* sp. nov., holotype, median lobe **A** left lateral view **B** right lateral view **C** ventral view **D** left paramere in external view. Scale bar: 1 mm (**A–C**).

**Figure 96.**
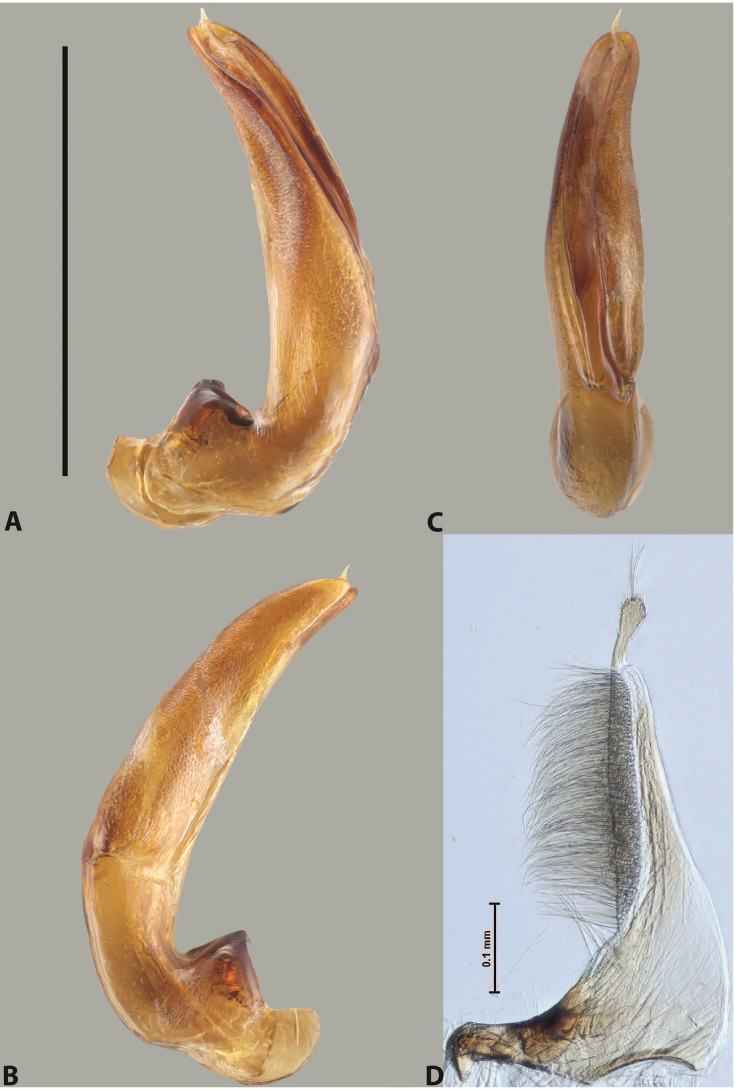
*Austrelatusmaindai* sp. nov., holotype, median lobe **A** left lateral view **B** right lateral view **C** ventral view **D** left paramere in external view. Scale bar: 1 mm (**A–C**).

**Figure 97.**
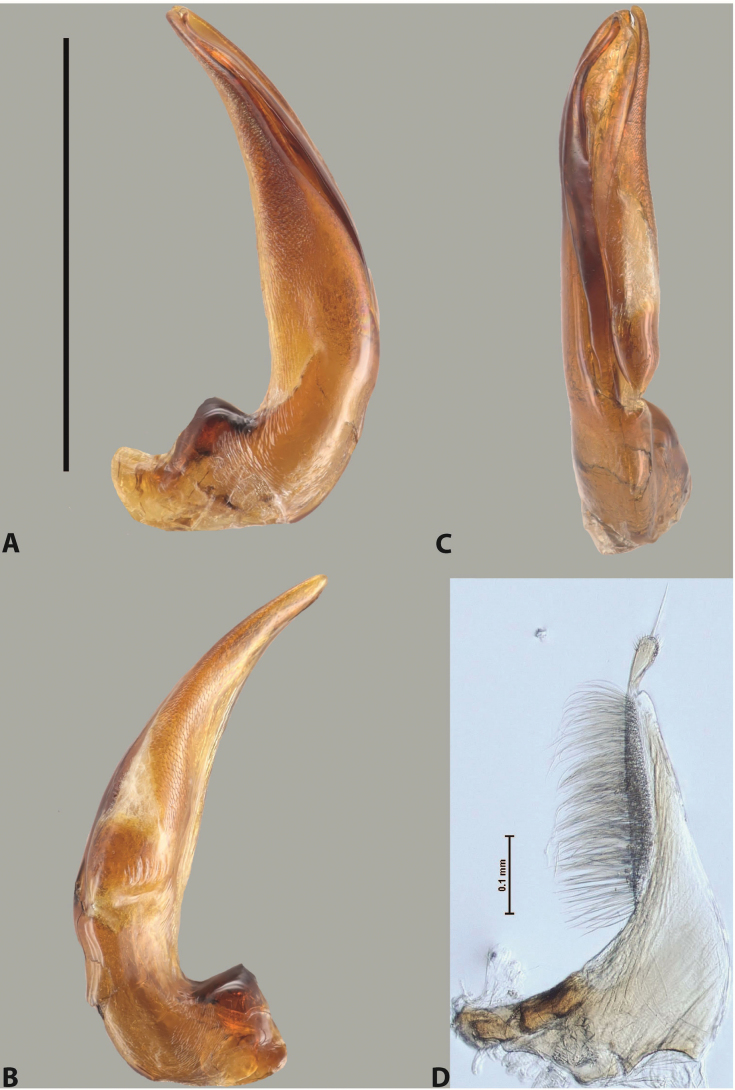
*Austrelatuskokodensis* sp. nov., holotype, median lobe **A** left lateral view **B** right lateral view **C** ventral view **D** left paramere in external view. Scale bar: 1 mm (**A–C**).

**Figure 98.**
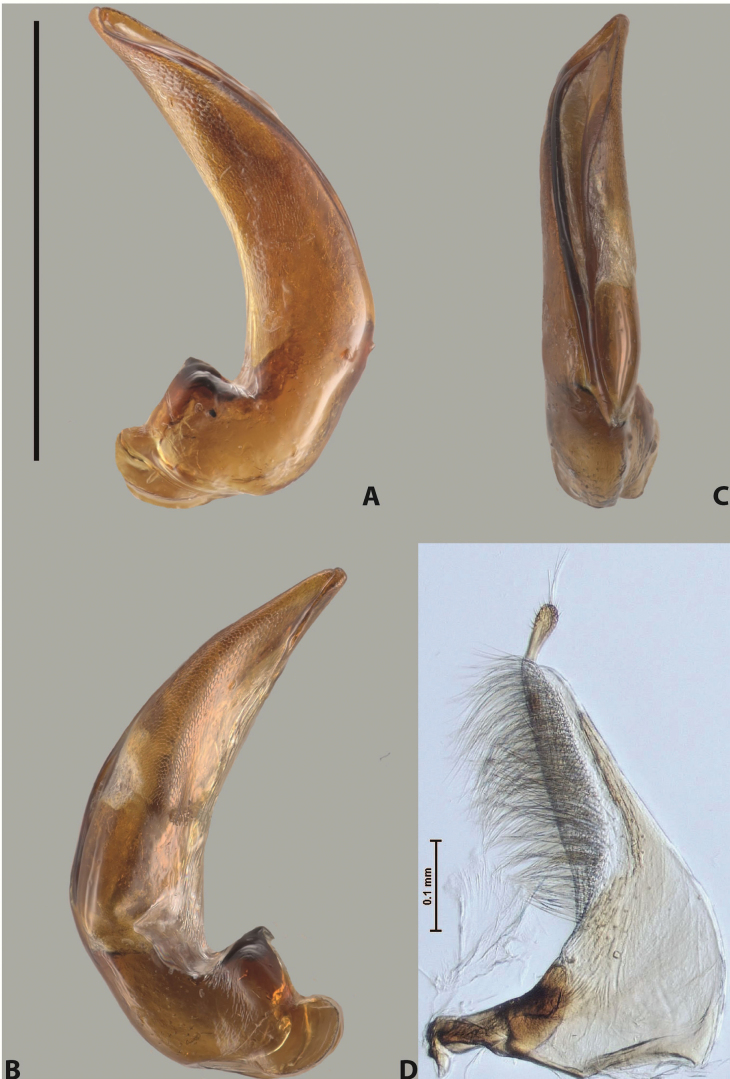
*Austrelatussararti* sp. nov., holotype, median lobe **A** left lateral view **B** right lateral view **C** ventral view **D** left paramere in external view. Scale bar: 1 mm (**A–C**).

**Figure 99.**
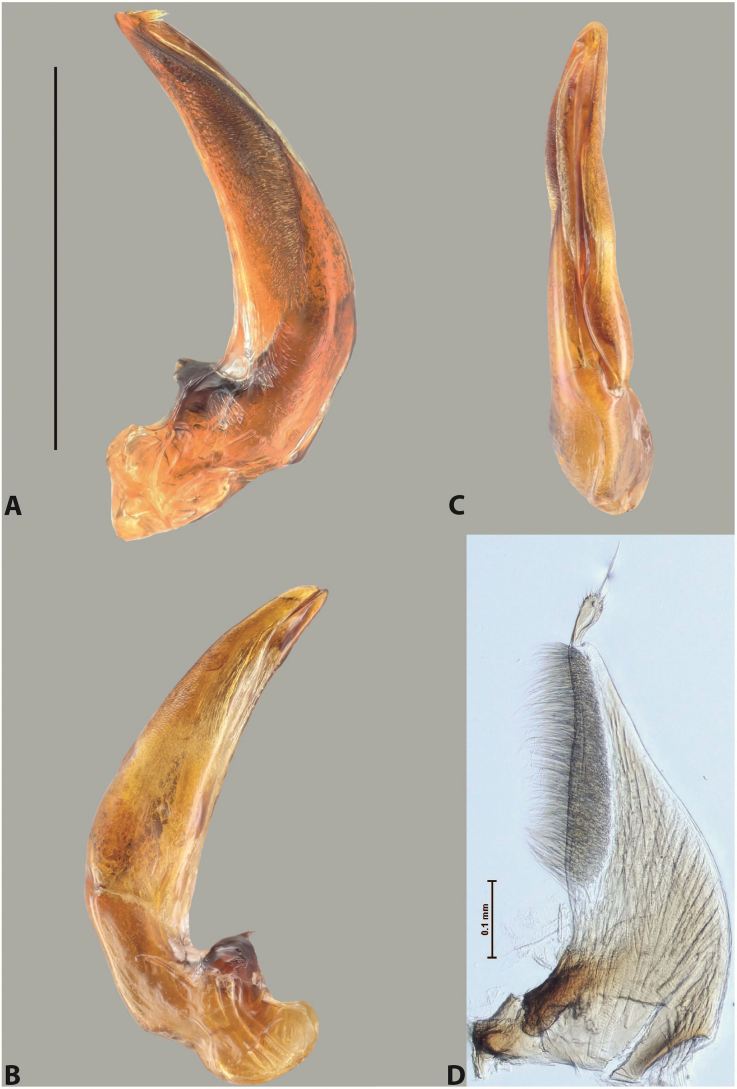
*Austrelatusfuscus* sp. nov., holotype, median lobe **A** left lateral view **B** right lateral view **C** ventral view **D** left paramere in external view. Scale bar: 1 mm (**A–C**).

**Figure 100.**
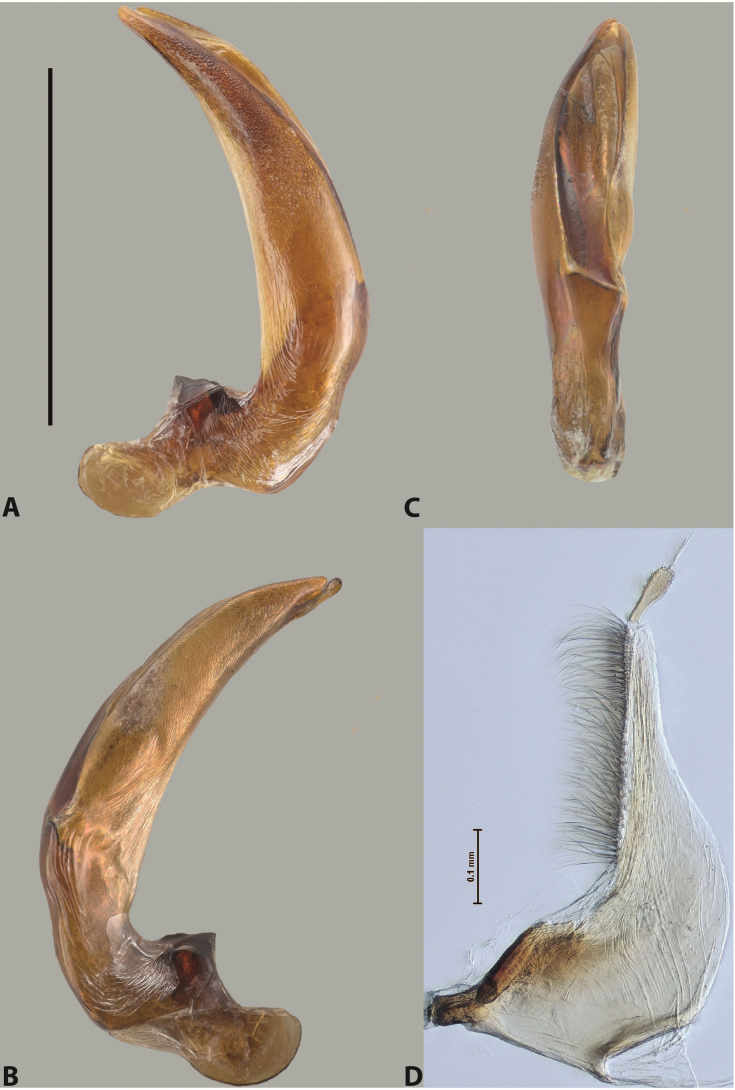
*Austrelatuswasiorensis* sp. nov., holotype, median lobe **A** left lateral view **B** right lateral view **C** ventral view **D** left paramere in external view. Scale bar: 1 mm (**A–C**).

**Figure 101.**
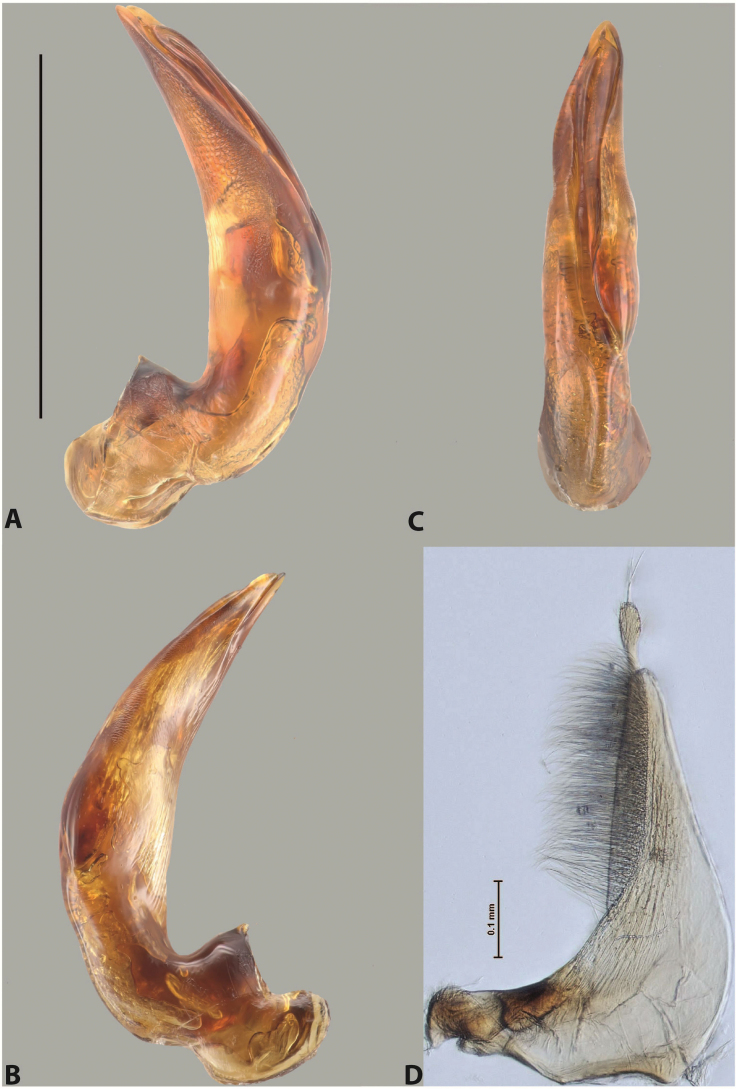
*Austrelatusgestroi* (Régimbart, 1892), lectotype, median lobe **A** left lateral view **B** right lateral view **C** ventral view **D** left paramere in external view. Scale bar: 1 mm (**A–C**).

**Figure 102.**
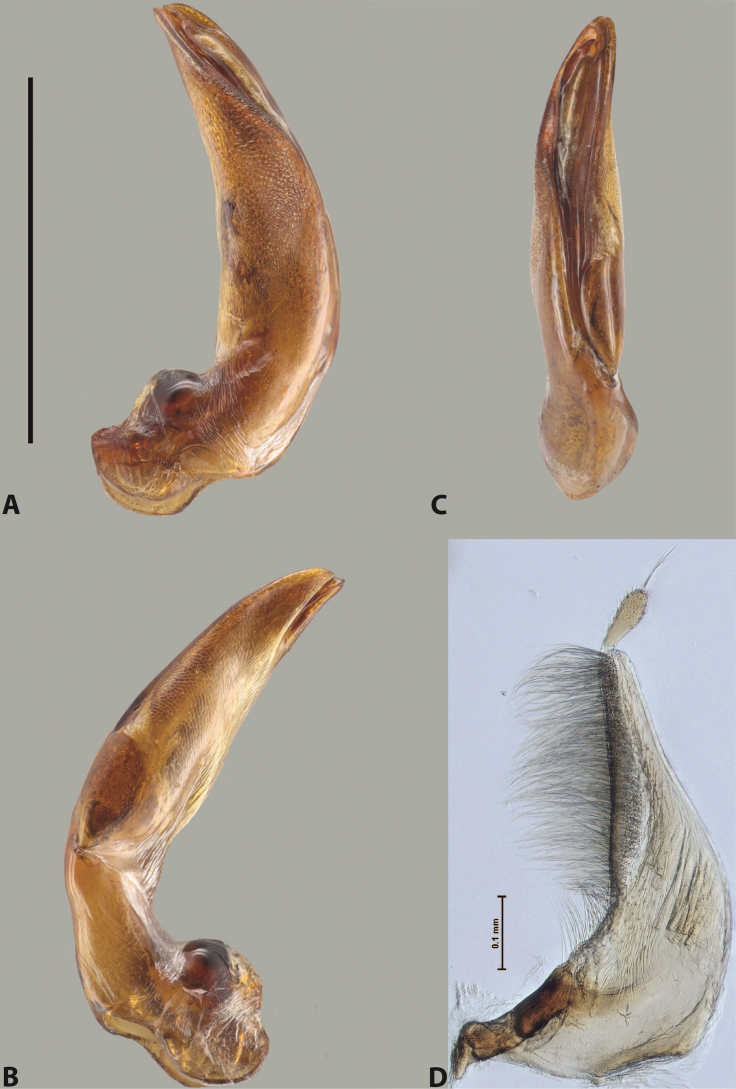
*Austrelatuspseudogestroi* sp. nov., holotype, median lobe **A** left lateral view **B** right lateral view **C** ventral view **D** left paramere in external view. Scale bar: 1 mm (**A–C**).

**Figure 103.**
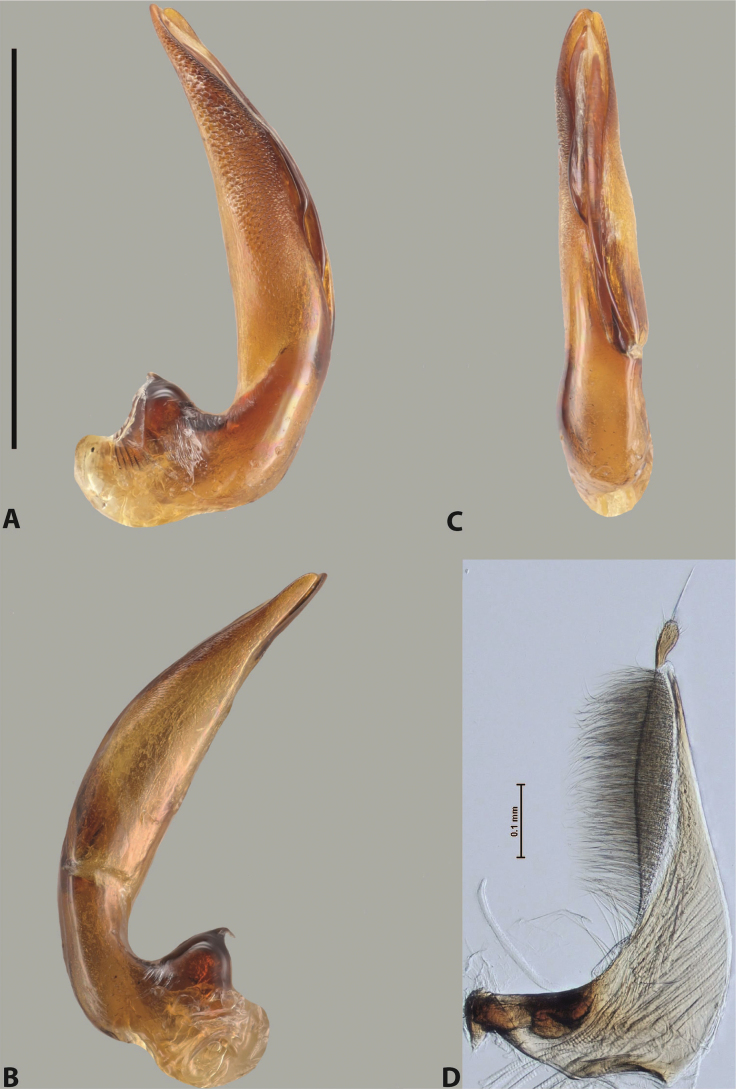
*Austrelatuswasurensis* sp. nov., holotype, median lobe **A** left lateral view **B** right lateral view **C** ventral view **D** left paramere in external view. Scale bar: 1 mm (**A–C**).

**Figure 104.**
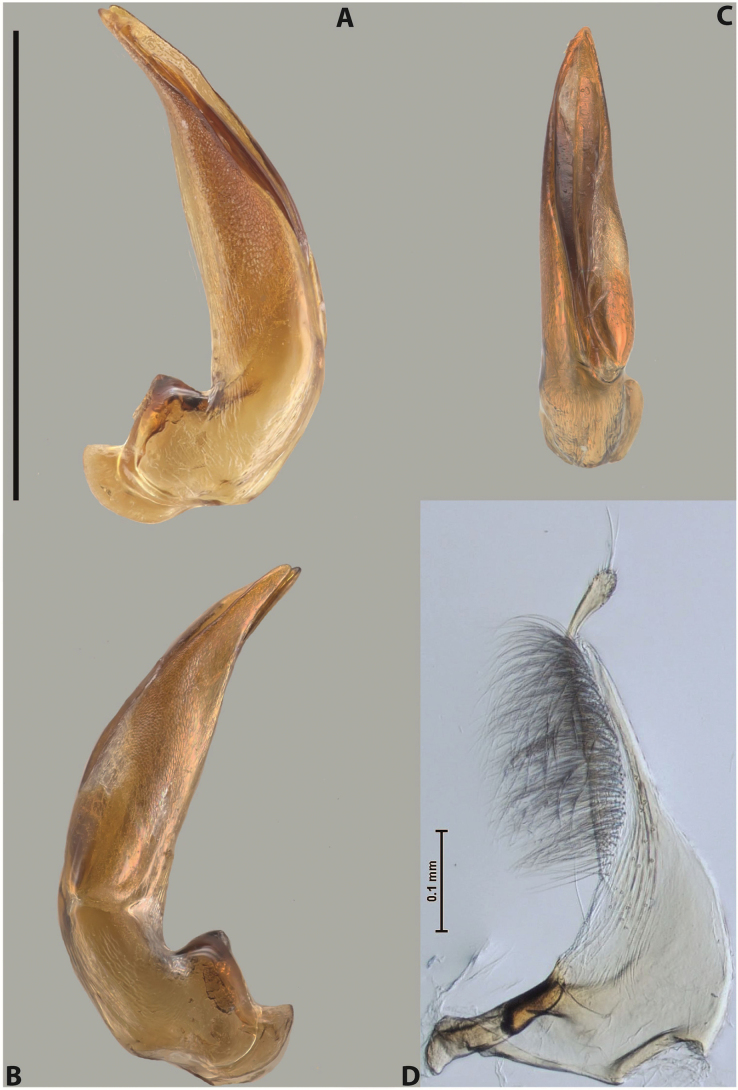
*Austrelatusyeretuar* sp. nov., holotype, median lobe **A** left lateral view **B** right lateral view **C** ventral view **D** left paramere in external view. Scale bar: 1 mm (**A–C**).

**Figure 105.**
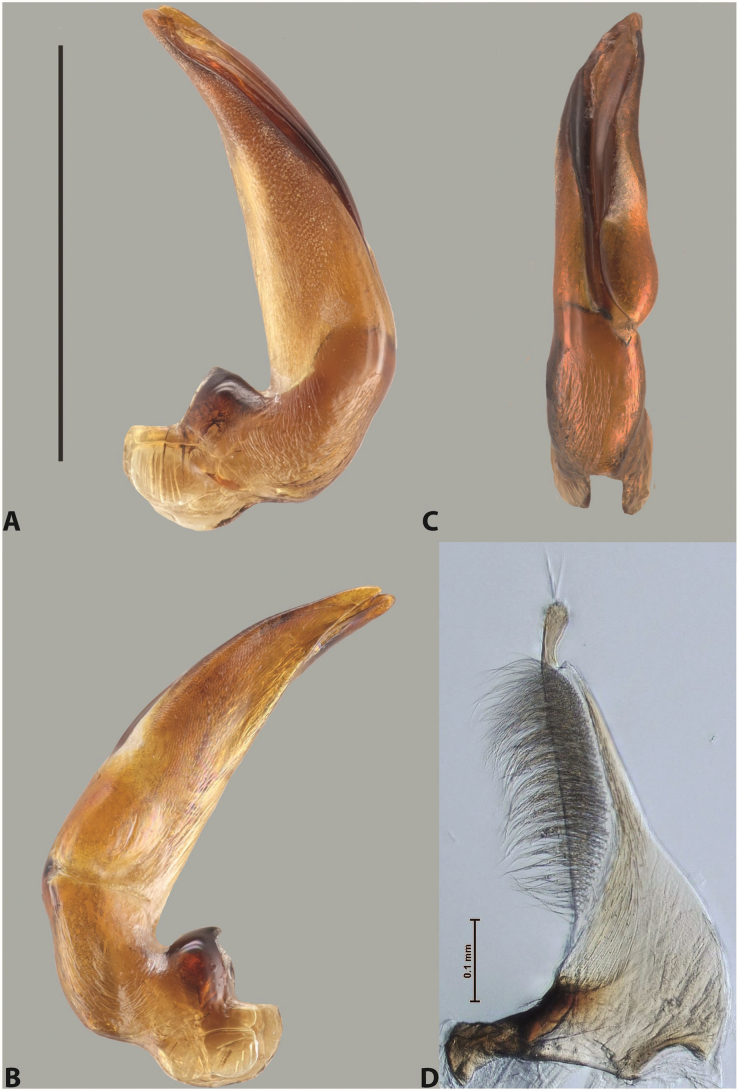
*Austrelatusasteios* sp. nov., holotype, median lobe **A** left lateral view **B** right lateral view **C** ventral view **D** left paramere in external view. Scale bar: 1 mm (**A–C**).

**Figure 106.**
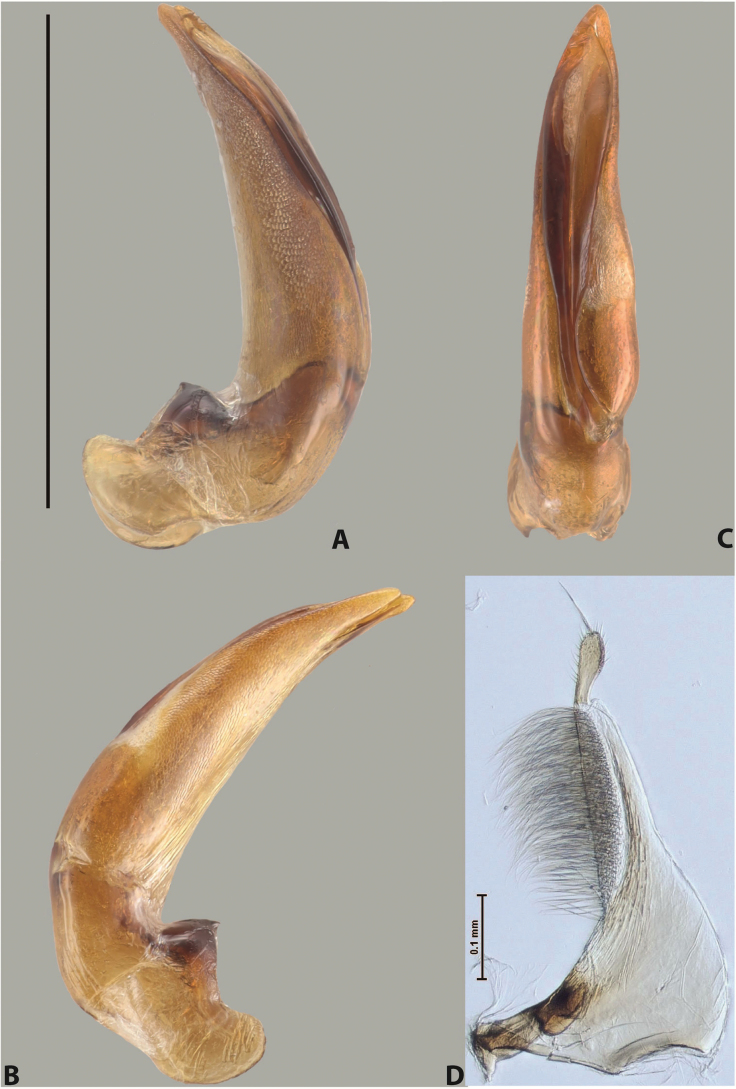
*Austrelatusepicharis* sp. nov., holotype, median lobe **A** left lateral view **B** right lateral view **C** ventral view **D** left paramere in external view. Scale bar: 1 mm (**A–C**).

**Figure 107.**
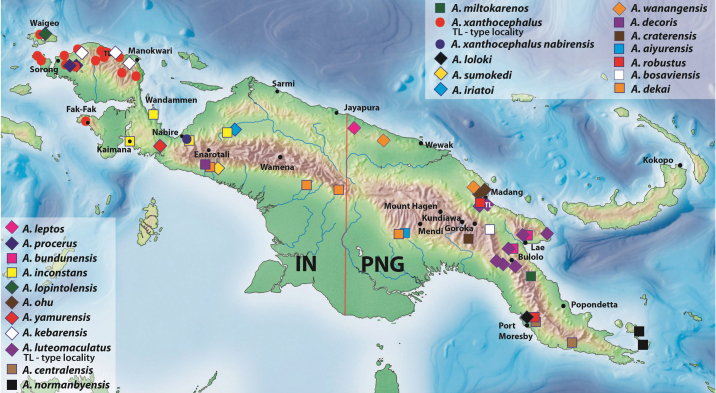
Map of New Guinea showing distribution of 23 *Austrelatus* species and one subspecies.

**Figure 108.**
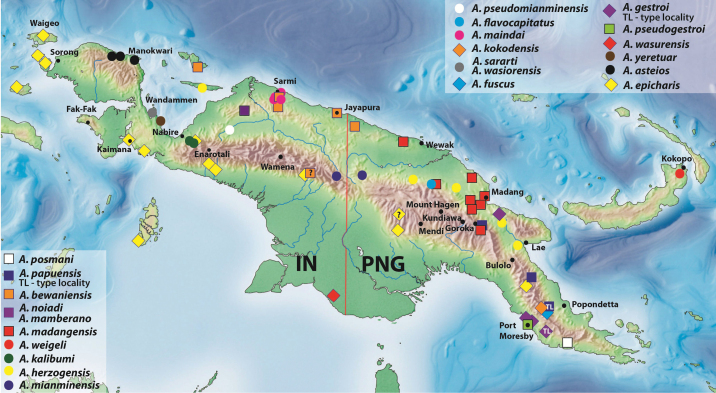
Map of New Guinea showing distribution of 23 *Austrelatus* species.

**Figures 109, 110.**
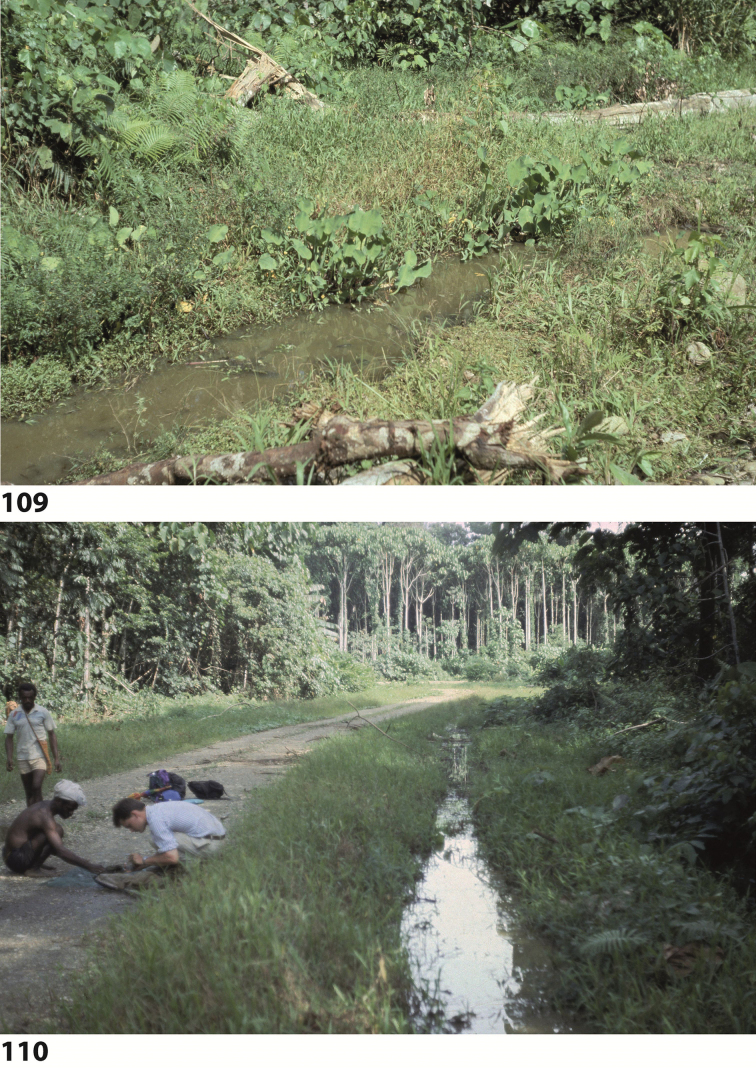
Habitats of *Austrelatus* species **109***A.epicharis* sp. nov., *A.kalibumi* sp. nov., and *A.inconstans* sp. nov.: Papua Province, Nabire Regency, track Nabire – Ilaga, km 34 (Topo), roadside ditch rich in rotten leaves **110***A.epicharis* sp. nov. and *A.inconstans* sp. nov.: track Nabire – Ilaga, km 35, partly shaded roadside ditch. Photographs by MB.

**Figures 111, 112.**
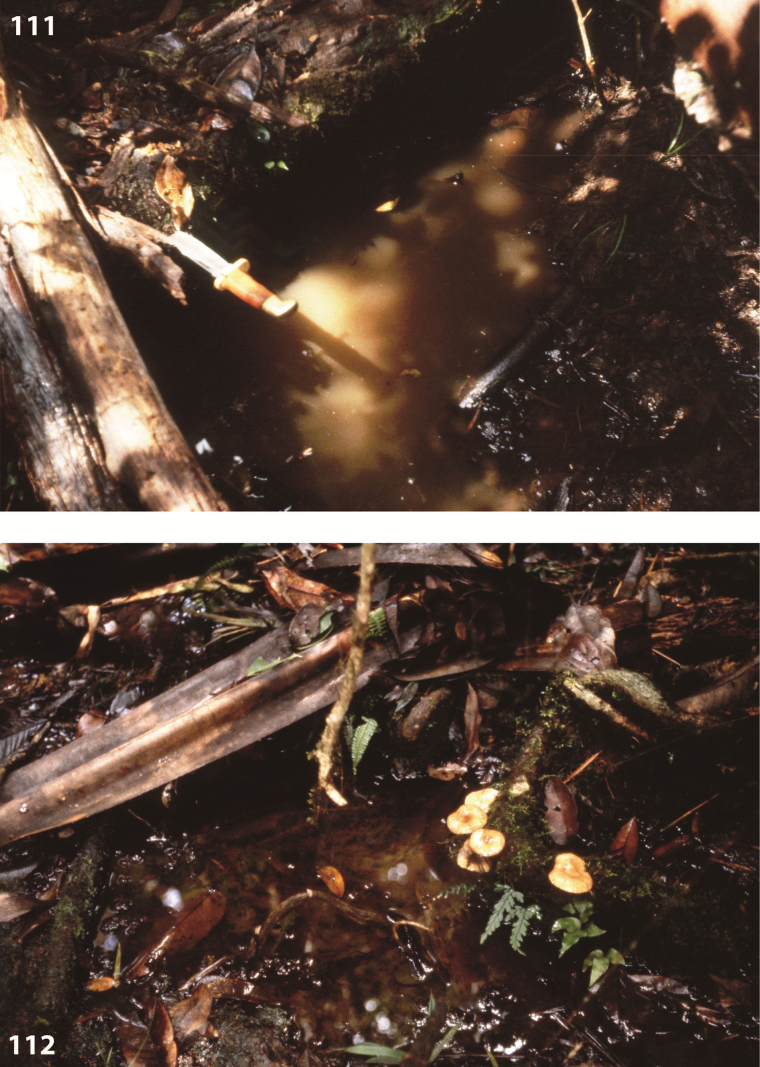
Habitats of *Austrelatusxanthocephalusnabirensis* ssp. nov. and *A.inconstans* sp. nov. Papua Province, Nabire Regency, track Nabire – Ilaga, km 54, shaded, temporary and small pools in primary rainforest, rich in rotten debris. Photographs by LH.

**Figures 113, 114.**
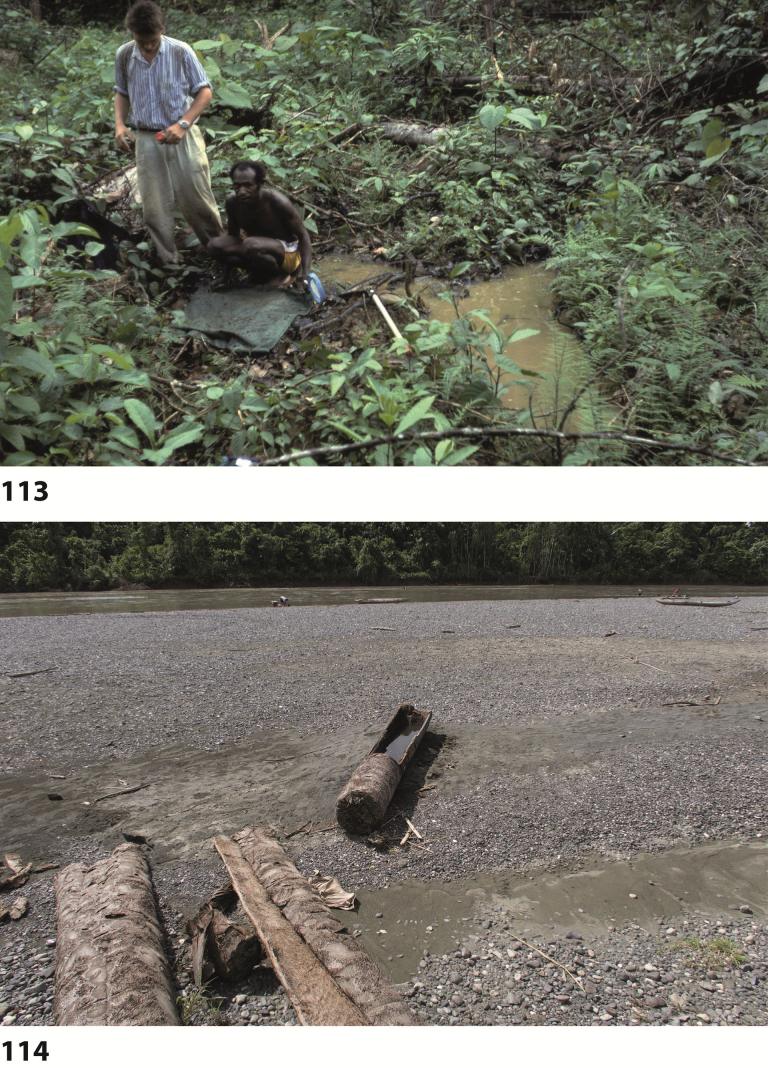
Habitats of *Austrelatus* species **113***A.xanthocephalusnabirensis* ssp. nov. and *A.inconstans* sp. nov.: Papua Province, Nabire Regency, track Nabire – Ilaga, km 54–62, exposed to sun, temporary puddle in rainforest clearing **114***A.maindai* sp. nov.: Sarmi Regency, Tor River, Togonfo, water filled tree trunk. Photographs by MB.

**Figures 115, 116.**
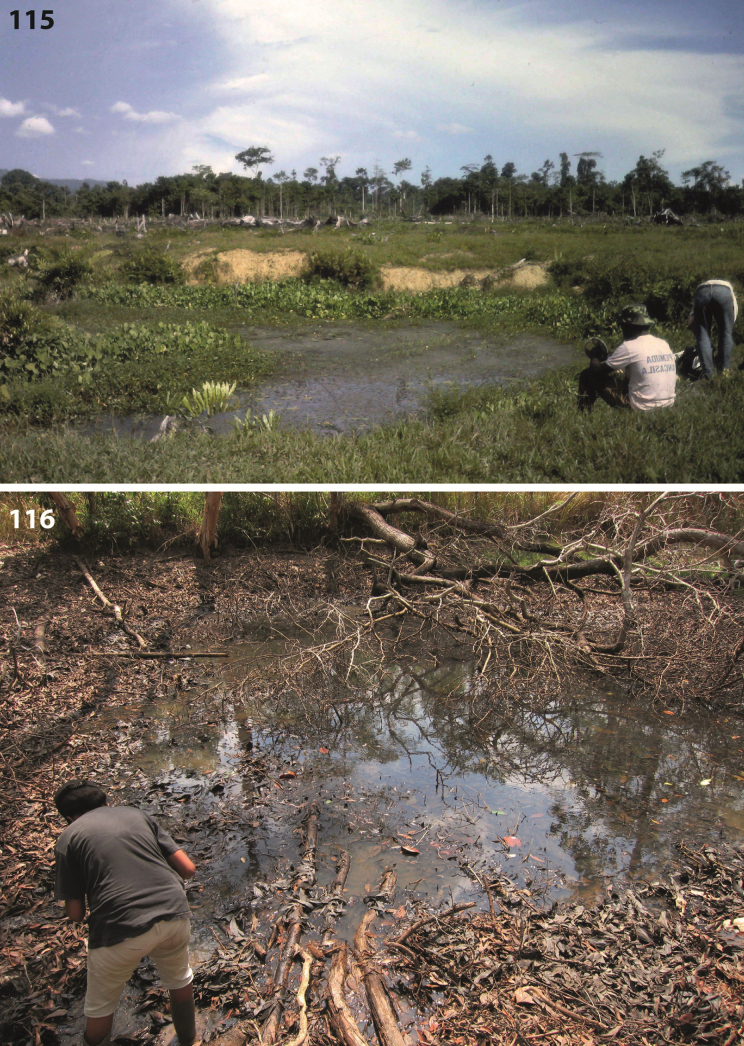
Habitats of *Austrelatus* species **115** type locality of *A.epicharis* sp. nov. and *A.kalibumi* sp. nov.: Papua Province, Nabire Regency, Wanggar, flooded meadows and shallow oxbows near Bumi River **116***A.wasurensis* sp. nov. and *A.clarkii* (Sharp, 1882): Papua Province, Merauke Regency, Wasur, shallow, almost dry pools in wet savannah forest. Beetles hiding deep in mud and rotten leaves. Photographs by MB.

### ﻿﻿Notes on other *Austrelatus* species from New Giunea

#### ﻿*Austrelatusclarki* (Sharp, 1882)

During the study of Régimbart’s collection in MNHN, a female specimen indicated as “*Copelatusvagestriatus* Rég.” was found: 1 female “NeuGuinea”, “vagestriatus Rég. n. sp. typ.” [hw, Régimbart], “MUSEUM PARIS COLL MAURICE REGIMBART 1908”, “MNHN, Paris EC29238 [with QR code]” (MNHN).

Zimmermann (1919: 199) described *Copelatusvagestriatus* with a note “(Rég. in litt.)” and clearly wrote that he based the description on the only female from the Berlin-Dahlem collection. With the discovery of the additional specimen with the similar labels in the Paris collection, it is obvious that Régimbart used at least these two specimens to indicate his unpublished species. Similar to the holotype, the newly found female also belongs to *A.clarki* but, having seven dorsal and one submarginal striae on the elytron, is more striated than the holotype.

#### ﻿*Austrelatusgarainensis*Shaverdo et al., 2023

First record from Normanby Island: 1 male, 2 females “Papua New Guinea: Milne Bay Prov., Normanby Isl., Sewa Bay, Sibonai,”, “10°02.418’S 150°58.461’E, 35 m, beaten & hand-collected,”, “30-VI-2017 – position 1, A. Riedel.” (SMNK, ZSM).

#### ﻿*Austrelatuskaszabi* (Guignot, 1956)

During the study of Guignot’s collection in MNHN, nine additional paratypes of *Copelatuskaszabi* Guignot, 1956 were found: 1 male “Stephansort Astrolabe B”, “N. Guinea Biró 97.”, “♂”, “Paratype” [red label with black frame]; 1 male, 7 females “N. Guinea Biró 1898”, “Simbang Huon Golf // IX.17. [hw on reverse side]”, “Paratype” [red label with black frame]. As well as one paratype in IRSNB: 1 male “N. Guinea Biro 1898”, “Simbang Huon Golf”, “♂”, “Paratype” [red label with black frame].

## Supplementary Material

XML Treatment for Austrelatus aiyurensis

XML Treatment for Austrelatus asteios

XML Treatment for Austrelatus bewaniensis

XML Treatment for Austrelatus bosaviensis

XML Treatment for Austrelatus bundunensis

XML Treatment for Austrelatus centralensis

XML Treatment for Austrelatus craterensis

XML Treatment for Austrelatus decoris

XML Treatment for Austrelatus dekai

XML Treatment for Austrelatus epicharis

XML Treatment for Austrelatus flavocapitatus

XML Treatment for Austrelatus fuscus

XML Treatment for Austrelatus gestroi

XML Treatment for Austrelatus herzogensis

XML Treatment for Austrelatus inconstans

XML Treatment for Austrelatus iriatoi

XML Treatment for Austrelatus kalibumi

XML Treatment for Austrelatus kebarensis

XML Treatment for Austrelatus kokodensis

XML Treatment for Austrelatus leptos

XML Treatment for Austrelatus loloki

XML Treatment for Austrelatus lopintolensis

XML Treatment for Austrelatus luteomaculatus

XML Treatment for Austrelatus madangensis

XML Treatment for Austrelatus maindai

XML Treatment for Austrelatus mamberamo

XML Treatment for Austrelatus mianminensis

XML Treatment for Austrelatus miltokarenos

XML Treatment for Austrelatus noiadi

XML Treatment for Austrelatus normanbyensis

XML Treatment for Austrelatus ohu

XML Treatment for Austrelatus papuensis

XML Treatment for Austrelatus posmani

XML Treatment for Austrelatus procerus

XML Treatment for Austrelatus pseudogestroi

XML Treatment for Austrelatus pseudomianminensis

XML Treatment for Austrelatus robustus

XML Treatment for Austrelatus sararti

XML Treatment for Austrelatus sumokedi

XML Treatment for Austrelatus wanangensis

XML Treatment for Austrelatus wasiorensis

XML Treatment for Austrelatus wasurensis

XML Treatment for Austrelatus weigeli

XML Treatment for Austrelatus yamurensis

XML Treatment for Austrelatus yeretuar

XML Treatment for Austrelatus xanthocephalus

XML Treatment for Austrelatus xanthocephalus nabirensis

## ﻿﻿Additional information

### Author ORCIDs

Helena Shaverdo https://orcid.org/0000-0001-5034-7342

Lars Hendrich https://orcid.org/0000-0001-8366-0749

Suriani Surbakti https://orcid.org/0000-0003-3984-2187

Michael Balke https://orcid.org/0000-0002-3773-6586

### Data availability

All of the data that support the findings of this study are available in the main text.
